# Modular Enantioselective Synthesis of *cis*-Cyclopropanes through Self-Sensitized Stereoselective Photodecarboxylation with Benzothiazolines

**DOI:** 10.1021/acscatal.1c03949

**Published:** 2021-10-18

**Authors:** Matteo Costantini, Abraham Mendoza

**Affiliations:** Department of Organic Chemistry, Arrhenius Laboratory, Stockholm University, 106 91 Stockholm, Sweden

**Keywords:** redox-active carbene, EDA complex, photochemistry, *cis*-cyclopropanes, stereoselective decarboxylation, benzothiazoline

## Abstract

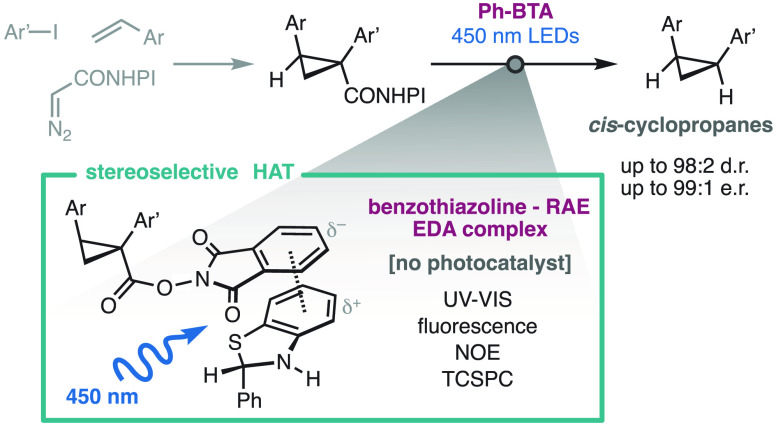

Chiral *cis*-cyclopropanes are strained rigid analogues of alkyl chains, whose study and application are limited by their difficult synthesis. A modular approach from olefin materials is enabled by the discovery of the electron donor–acceptor (EDA) interaction between 2-substituted benzothiazolines and *N*-hydroxyphthalimide esters. These complexes are activated by visible light without photocatalysts, and the benzothiazoline reagent plays a triple role as a photoreductant, a stereoselective hydrogen-atom donor, and a Brønsted acid. Beyond the enantioselective synthesis of *cis*-cyclopropanes, these results introduce benzothiazolines as accessible and easily tunable self-sensitized photoreductants.

Cyclopropanes are central motifs in organic synthesis.^1^ They have been widely used in the field of medicinal chemistry to improve the properties of potential drug candidates due to their resistance toward metabolic degradation and their structural rigidity (Scheme 1A).^1c,2^ As such, several enantioselective protocols have been developed over the years, mainly targeting the more thermodynamically and kinetically favored *trans*-cyclopropanes.^3^ In contrast, the synthesis of *cis*-cyclopropanes, an important class of stable and conformationally restricted alkyl chain analogues,^1c,2a,4^ remains a synthetic challenge with only a limited number of protocols being reported.^5^

**Scheme 1:**
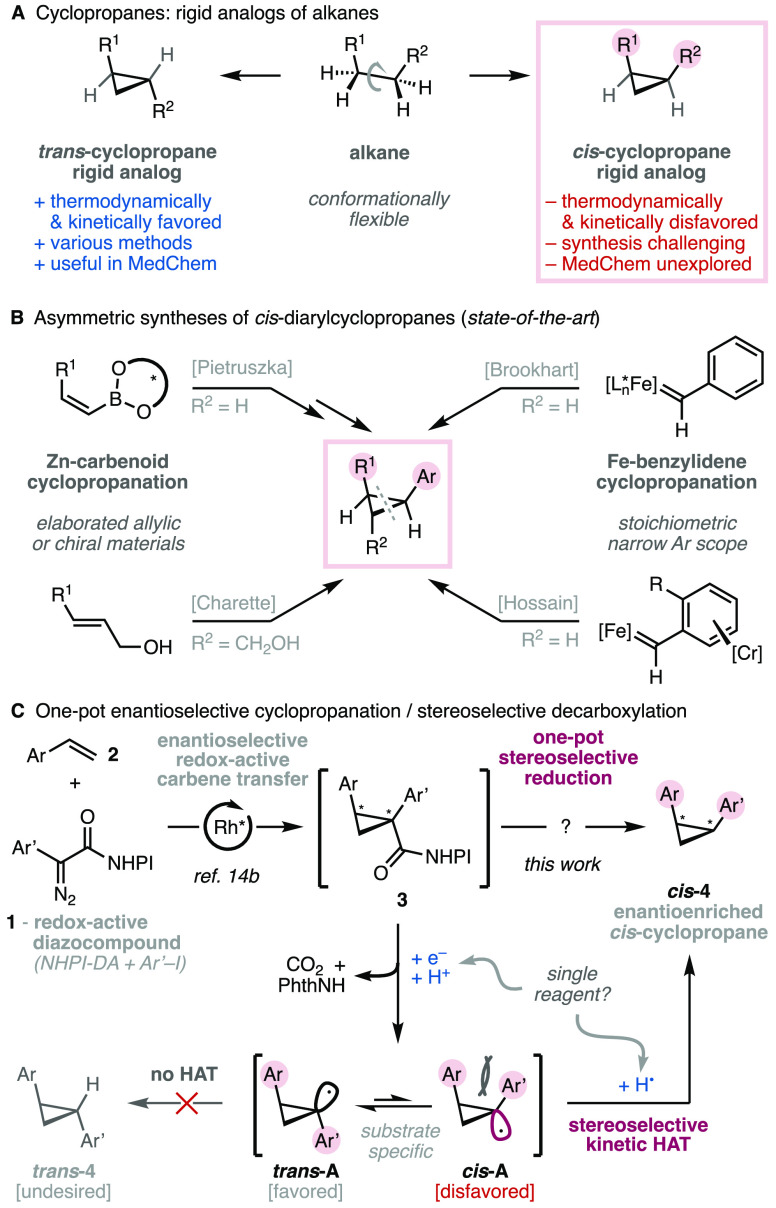
Background and Concept

The asymmetric syntheses of these products require the preparation and derivatization of enantiopure (*Z*)-vinylboronates (Scheme 1B, top left)^6^ or complex catalytic systems employing transition metals^7^ or engineered proteins^8^ to obtain cyclopropyl esters. The complexity of these catalysts^7,8^ highlights the challenge to kinetically favor *cis*-cyclopropanes over their more stable *trans* diasteroisomers. Desirable catalytic approaches only offer limited scope^9^ or low diastereo- and enantioselectivity.^10^ In particular, the *cis*-cyclopropanation of alkenes employing benzylidenes is still problematic, due to the instability of the phenyldiazomethane precursors and the difficult taming of the resulting reactive intermediates. Thus, current methodologies are mostly nonenantioselective,^11^ and the only asymmetric catalytic methods require specific allylic alcohol materials (Scheme 1B, bottom left).^12^ Seminal studies with chiral iron benzylidenes have also been reported but require stoichiometric chiral complexes and are limited in scope (Scheme 1B, right).^13^ Also, a diastereoselective approach from the chiral pool has been demonstrated by a single example^5f^ using the decarboxylation of a Barton ester. Nevertheless, this approach has not found further applications due to the long route to access chiral cyclopropyl Barton esters and the large excess of expensive tris(trimethylsilyl)silane required to trap the *cis* isomer of the cyclopropyl radical intermediate.^5f^

Recently, our group reported the use of redox-active diazoacetate reagents for the general enantioselective synthesis of cyclopropane building blocks from feedstock olefins.^14^ We envisioned that redox-active aryldiazoacetates **1** could be used to convert olefins **2** into *cis*-arylcyclopropanes ***cis*-4**, by means of sequential asymmetric cyclopropanation and stereoselective decarboxylative reduction of the redox-active ester (RAE, **3**; Scheme 1C). The cyclopropyl radical intermediates ***cis*-A** and ***trans*-A** are known to be σ-hybridized (pyramidal) and more electrophilic than conventional alkyl σ-radicals.^15a^ Their stereoinversion is rapid even at extremely low temperatures (*k*_inv_ ≈ 10^8^–10^9^ s^–1^), and this results in thermodynamically controlled stereoselectivities.^15^ Thus, the feasibility of this methodology was contingent upon the design of a suitable hydrogen atom transfer (HAT) reagent that would kinetically control the reaction with the less populated (less stable) *cis*-cyclopropyl radical conformer (*cis***-A**) in the equilibrium. In this respect, the high reactivity of cyclopropyl radicals^15a^ further complicates the challenge to combine chemoselectivity (efficiency) and stereocontrol.

Initially, we evaluated known HAT reagents for the reduction of model substrate **3a** (Table 1). It was found that the known nickel-catalyzed protocol,^16^ although highly diastereoselective, could only provide the desired cyclopropane ***cis*-4a** in low yields (entry 1). In contrast, chloroform^17^ could not afford high stereoselectivity (entry 2). The photoreductions using Hantzsch ester^18^ or *N*-butyl dihydronicotinamide (**5b**)^19^ recently developed by Shang et al.^18a^ and our group^19^ were promising (entries 3 and 4), but further attempts to increase the yield or diastereoselectivity by tuning the structure of the dihydropyridines proved unsuccessful (see the Supporting Information for details). On account of these results, we explored the possibility of employing a reductant with a more sterically hindered hydrogen atom to impose a higher kinetic barrier in the HAT toward the undesired diasteroisomer ***trans*-4a**. 2-Substituted benzothiazolines (BTA, **6**) have been used as alternative hydride sources to Hantzsch esters in transfer hydrogenation reactions.^20^ More recently, these compounds have been used as hydrogen atom donors in photocatalytic reactions^21^ requiring auxiliary thiyl radical carriers^21b^ or metal photocatalysts.^21a^ However, benzothiazolines have never been employed as self-sensitized photoreductants or in reductive decarboxylative reactions, as far as we know. We explored several benzothiazolines in this context, finding promising results (entries 5–10). In particular, phenyl- and *tert*-butylbenzothiazolines **6a**,**b** (entries 5 and 6) provide an optimal performance with the highest diastereoselectivity, whereas other substituents result in either lower yields or lower diastereomeric ratios (entries 7–10). Control experiments with the optimal reagents **6a**,**b** confirmed the need for blue-light irradiation for efficient reduction (entries 11 and 12). These results introduce the benzothiazoline platform for the design of cheap, easy to handle, readily available, and fine-tunable HAT reagents in reductive decarboxylative reactions without any auxiliary light harvesting or chain carrier systems.

**Table 1:**
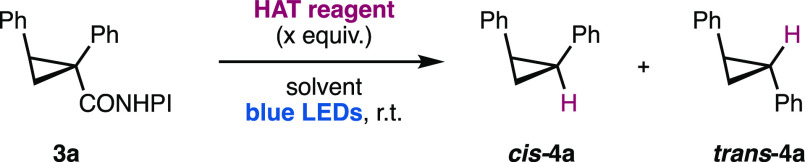
Optimization of the Stereoselective Decarboxylative Reduction of Redox-Active Ester **3a**^a^

entry	HAT reagent	*x* (equiv)	solvent	**4a** (%)^b^	d.r. (*cis*:*trans*)^c^
1^d^^,^^e^	PhSiH_3_	1.5	footnote e	30	90:10
2^f^	CHCl_3_	>100	CHCl_3_	43	77:23
3	**5a**	1.2	DMSO	76	90:10
4	**5b**	1.2	DMSO	60	94:6
5	**6a**	1.2	DMSO	88	95:5
6	**6b**	1.2	DMSO	81	95:5
7	**6c**	1.2	DMSO	nd	
8	**6d**	1.2	DMSO	92	89:11
9	**6e**	1.2	DMSO	54	88:12
10	**6f**	1.2	DMSO	44	90:10
11^d^	**6a**	1.2	DMSO	<10	97:3
12^d^	**6b**	1.2	DMSO	nd	

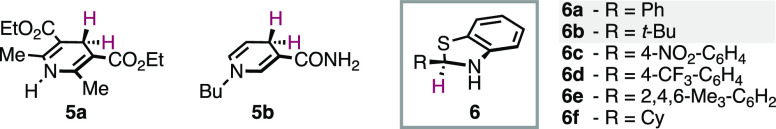

a See the Supporting Information for details.

b Yields measured by ^1^H NMR using 1,1,2,2-tetrachloroethane as an internal standard.

c Determined by GC-MS.

d No light irradiation.

e Reaction conditions: PhSiH_3_ (1.5 equiv), Zn (0.5 equiv), NiCl_2_(H_2_O)_6_ (10 mol %), 4,4′-di-*t-*Bu-2,2′-bipyridyl (20 mol %), THF:DMF:^*i*^PrOH 10:2:1, 40 °C.

f Reaction conditions: Et_3_N (2 equiv), 4CzIPN (2 mol %), CHCl_3_.

The simplicity of the new photoreduction conditions allowed us to telescope the cyclopropanation and stereoselective reduction into a one-pot method that delivers *cis*-cyclopropanes ***cis*-4** from olefins **2** and redox-active diazo compounds **1**. The latter are modularly synthesized from unsubstituted NHPI-DA (**7**) and aryl iodides **8** through a method previously developed by our group.^14b^ The scope of the one-pot synthesis of *cis*-cyclopropanes was explored using the optimal benzothiazoline **6a**, which was easily prepared and stored in multigram amounts.

For the initial cyclopropanation step, we adapted the recently reported conditions by our group^14b^ using strictly stoichiometric amounts of the olefin (1.0 equiv) and a shorter reaction time (5 h). As shown in Scheme 2A, electron-rich and electron-poor styrenes were tolerated in this transformation, furnishing *cis*-diarylcyclopropanes **4b**–**l** in good yields and high enantio- and diastereoselectivities. Substitutions in various positions in the aromatic ring were tolerated. Interesting naphthyl (**4i**) and indolyl (**4j**) cyclopropanes could also be generated with this protocol. The slightly lower stereoselectivity observed in the tricyclic indene derivative **4k** may be explained by a slower stereoinversion equilibrium or the particular instability of the corresponding trisubstituted *cis*-cyclopropyl radical intermediate. Divinylbenzene undergoes double *cis*-cyclopropanation to afford the *C*_2_-symmetric product **4l** as a single enantiomer in 43% yield over the four reactions performed in one pot. It is important to note that negligible erosion of stereoselectivity was observed for all products relative to the intermediate cyclopropanes,^14b^ indicating that the stereochemical information is conserved throughout the photochemical reduction step. The photodecarboxylation can also proceed with aliphatic substrates, albeit with lower stereoselectivity (82:18)^22^ likely due to lower facial discrimination in the key HAT process. Further optimization of the benzothiazoline structure to address the limitation in this substrate class is ongoing in our laboratories. The modular nature of the NHPI-aryldiazoacetates allows for the asymmetric transfer of a variety of aromatic fragments. This way, olefin **2a** can be transformed into a number of *cis*-cyclopropane products decorated with different functionalities (**4m**–**u**), which include pendant alkyne (**4p**), nitrile (**4r**), and ketone (**4t**) moieties. To further explore the synthetic potential of this system, we obtained a *cis*-cyclopropane-modified phenylalanine amino acid (**4u**) in two steps from commercially available 4-iodophenylalanine. Moreover, the asymmetric total synthesis of the combretastatin A4 analogue **4v**(6a) was achieved in three steps starting from isovanillin (**9**) in 39% overall yield (Scheme 2B). To put these results in perspective, twice as many steps (including a resolution) were previously required to obtain this product in <10% overall yield from comparable materials.^6a^

**Scheme 2:**
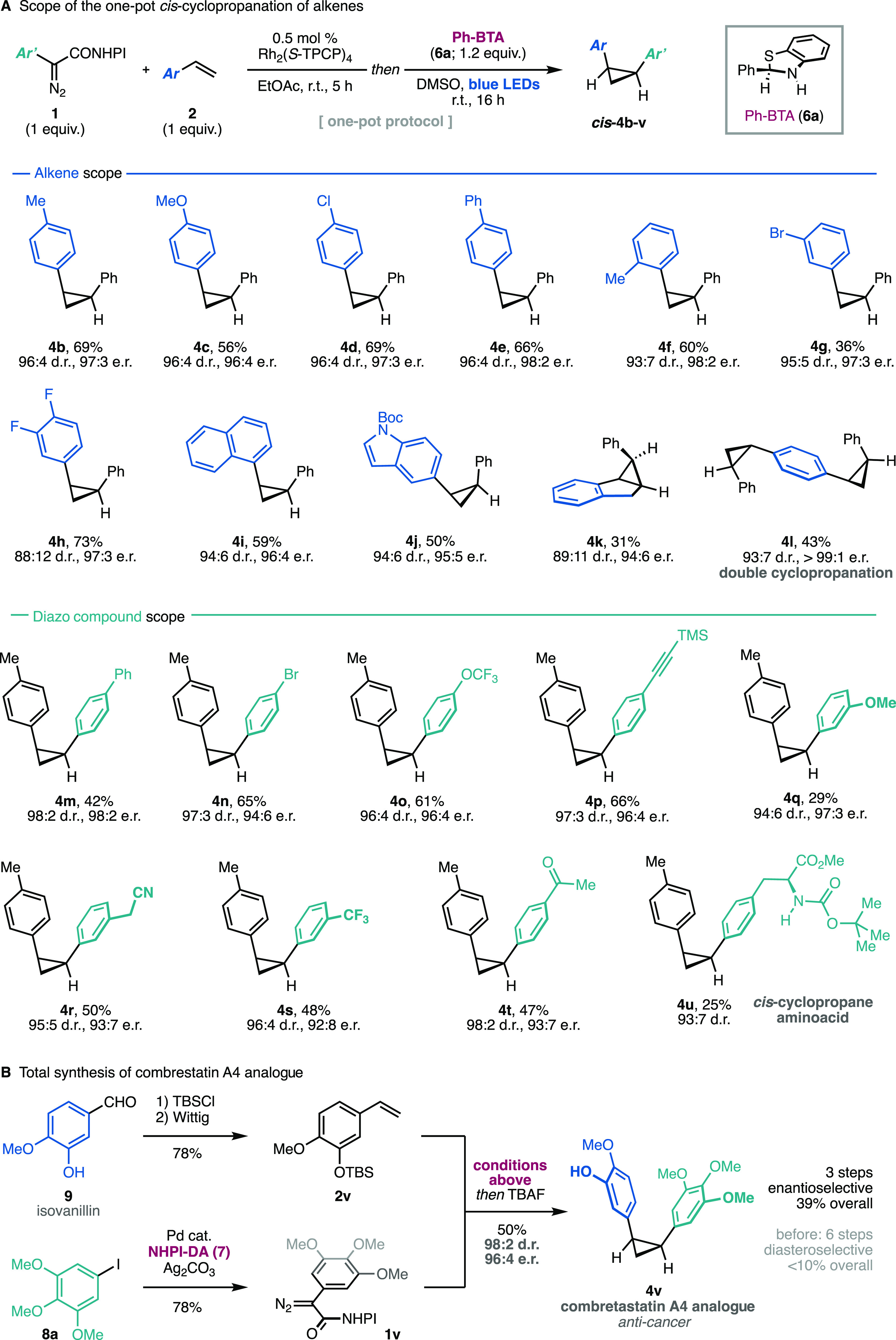
Scope Studies and Synthetic Applications Reaction conditions: **1** (1 equiv), **2** (1 equiv), Rh_2_(*S*-TPCP)_4_ (0.5 mol %), EtOAc (0.05 M), r.t., 5 h; then **6a** (1.2 equiv), DMSO (0.1 M), 450 nm LEDs, rt, 16 h. Isolated yields are given. Diasteromeric ratios were determined by HPLC. The photodecarboxylation of aliphatic substrates proceeds with lower stereoselectivity.^22^

The autonomous photoactivation of benzothiazoline **6a** was unexpected on the basis of the previously known reactivity of these systems based on HAT followed by proaromatic radical reduction with auxiliary photosensitization or chain carriers.^21^ Thus, photochemical studies were performed to investigate the mechanism of the photoreduction. UV–visible spectroscopy revealed that neither 2-phenylbenzothiazoline **6a** nor NHPI-ester **3a** absorb light effectively in the visible range (Figure 1A). Upon mixing, enhanced absorption in the visible range (450 nm) is observed, and a Job plot (Figure 1B) revealed that it is at a maximum when **3a** and **6a** are mixed in a 1:1 stoichiometry, suggesting that a bimolecular EDA complex^23^ absorbing at the LED irradiation wavelength is the dominant species in solution. Clearly defined excitation and emission features (λ_max_ = 435 nm; λ_em_ = 490 nm) of the new EDA complex can also be detected by fluorescence (Figure 1C). The formation of this species is further confirmed by time-correlated single photon counting (TCSPC), which allowed us to identify different fluorescence lifetimes for the benzothiazoline **6a** (τ_0_ = 1.7 ns) and the EDA complex (τ = 1.4 ns). Stern–Volmer quenching studies performed by increasing the concentration of redox-active ester **3a** revealed an unconventional increase in the steady-state fluorescence intensity (see the Supporting Information), while the corresponding fluorescence lifetime remained constant (Figure 1D). This feature strongly supports a static quenching scenario through the formation of a more emissive bimolecular EDA complex, and it rules out dynamic processes involving the excited state of free benzothiazoline (6a*) that would instead result in a concentration-dependent decrease in the observed fluorescence lifetime.

**Figure 1:**
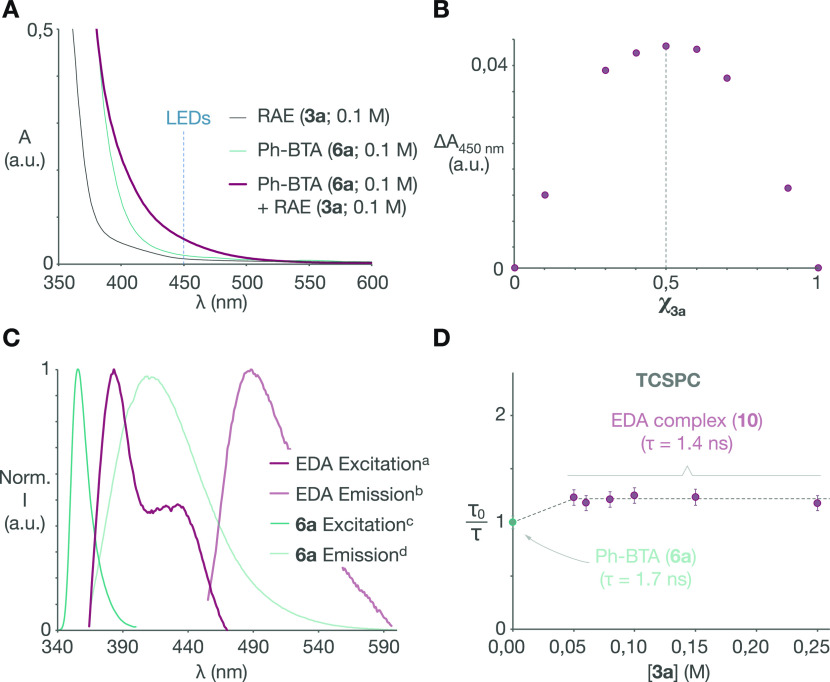
Photophysical characterization of the stereoselective photodecarboxylation: (A) UV–visible spectra of NHPI-ester **3a**, Ph-BTA (**6a**), and their 1:1 mixture; (B) Job plot of mixtures **3a/6a** measured at 450 nm (*c*_tot_ = 0.1 M); (C) Normalized excitation and emission spectra of Ph-BTA (**6a**; 0.02 M) and its EDA complex (0.1 M) with NHPI-ester **3a** (EDA emission profile recorded at the excitation wavelength of TCSPC measurements); (D) lifetime Stern–Volmer plot of Ph-BTA (**6a**; 0.1 M) with NHPI-ester **3a** (λ_ex_ = 450 nm) determined by TCSPC. Legend: (a) λ_em_ = 490 nm; (b) λ_ex_ = 450 nm; (c) λ_em_ = 410 nm; (d) λ_ex_ = 355 nm.

Despite our initial hypothesis, our results could also be explained by a fast stereoretentive hydrogen atom transfer (HAT). To explore this possibility, the diastereoisomer of the redox-active cyclopropane ***diast*-3a** was independently synthesized and subjected to the reaction conditions (Scheme 3A). A similar yield and stereoselectivity for the product ***cis*-4a** is observed, demonstrating that the stereoinversion equilibrium is faster than the HAT process and that the latter is kinetically controlled. This result opens the door for future stereoconvergent applications. In principle, benzothiazoline radical cations have two hydrogen atoms susceptible to undergo the key HAT transfer. To assess their relative contribution, several deuterium incorporation experiments were carried out (Scheme 3B). A first control experiment with DMSO-*d*_6_ ruled out any relevant contribution from the solvent. The monodeuterated benzothiazoline at the benzylic carbon **6a**-*d*_**1**_ resulted in 70% deuterium incorporation (56% yield), while the analogue deuterated in the N–H moiety **6a**-*d*_**1**_**′** led to <5% isotopic labeling and higher efficiency (77% yield). These observations indicate that the benzylic C–H bond is the main hydrogen atom donor but HAT from either the N–H moiety or the imine tautomer^24^ of **6a** may have a secondary role. Indeed, the use of benzothiazoline **6a**-*d*_**2**_ increased the degree of deuteration to >90%, thus accounting for the most relevant HAT processes. These results are consistent with the variable diastereoselectivities observed in the benzothiazoline screening (Table 1) with aliphatic (entries 6 and 10) and aromatic substituents (entries 5, 8, and 9) of different sizes at the C2 position, which affect the relative barriers of the HAT step. Furthermore, the quantum yield of the reaction was determined to be 0.09 ± 0.03 (Scheme 3C), disfavoring the possibility of a radical-chain mechanism. This behavior is fundamentally distinct from that of the previously known dihydronicotinamide system **5b**, operating through a radical chain reaction.^19,25^ The formation of the EDA complex was also directly observed by ^1^H NMR NOE experiments (see the Supporting Information)^26^ that clearly evidence the spatial proximity of **3a** and **6a** in their equimolar mixture in DMSO (Scheme 3C).

**Scheme 3:**
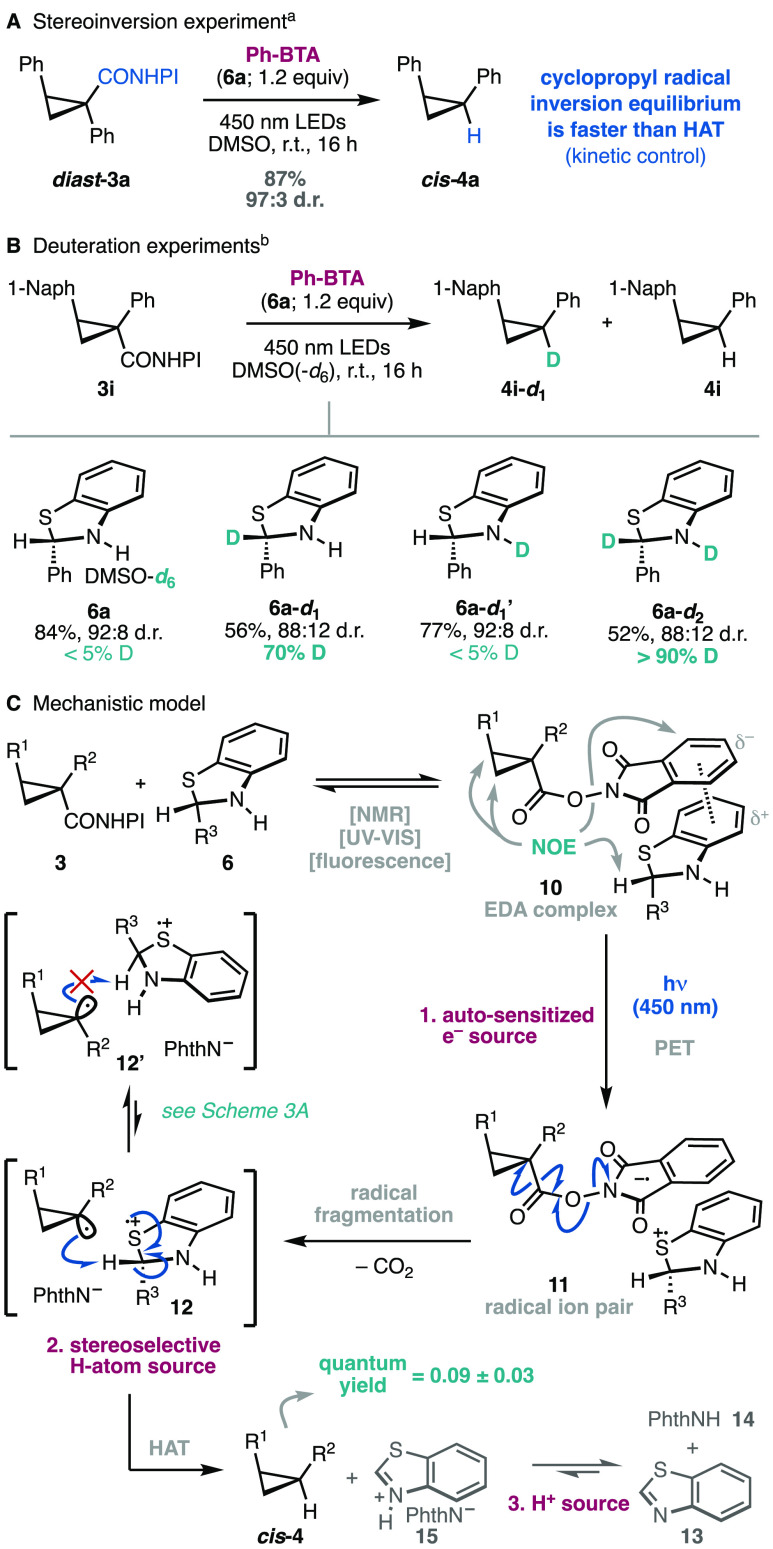
Mechanistic Experiments and Model See the Supporting Information for details. Diastereomeric ratios were determined by (a) GC-MS or (b) ^1^H NMR.

The data presented above supports the mechanism presented in Scheme 3C. Redox-active esters **3** and benzothiazoline **6** associate in solution to form the EDA complex **10**, which undergoes photoinduced electron transfer (PET) in the excited state to form the radical ion pair **11**. After fragmentation of the NHPI moiety with loss of CO_2_, the resulting cyclopropyl radical abstracts a hydrogen atom primarily from the benzylic C–H bond in the benzothiazoline radical cation (intermediate **12**). The alternative HAT process through the N–H bond seems to have a secondary role. Either way, the *cis*-cyclopropane product **4** is kinetically preferred despite the higher energy of the *cis*-cyclopropyl radical in comparison to that of the alternative *trans* conformer (intermediate **12′**). The HAT produces benzothiazole (**13**) and phthalimide (**14**) after an acid–base reaction of the phthalimidate salt **15**. The alternative possibility of the cyclopropyl radical undergoing HAT directly with the benzothiazoline **6** would result in radical chain reactions that can be ruled out on the basis of the quantum yield measurements. Remarkably, the benzothiazoline **6a** has a triple role in this system as a self-sensitized single-electron photoreductant to promote the fragmentation of the redox-active ester, a sterically tuned hydrogen atom source to enhance stereoselectivity, and a proton source to neutralize the phthalimidate anion byproduct.

In summary, a general and highly enantioselective method to obtain *cis*-diarylcyclopropanes from olefins and redox-active carbenes has been developed. This protocol allows for quick and modular access to ring-strained and conformationally strained compounds from available olefin materials, ultimately facilitating the synthesis of interesting bioactive molecules. These advances are bestowed by a new, efficient, and stereoselective photodecarboxylation driven by a novel EDA complex between redox-active esters and benzothiazoline reagents. The photophysical properties of the newly discovered system have been investigated, disclosing a new reactivity manifold of benzothiazolines as single-electron transfer reagents. Beyond enantiopure *cis*-cyclopropanes, these discoveries open the door for further progress in reductive decarboxylative reactions driven by benzothiazolines as a new platform to develop fine-tuned autonomous photoreductants.

## Supporting Information Available

Experimental procedures, characterization data, and mechanistic experiments (PDF)

## Notes

NMR, HRMS and HPLC raw data for this article can be downloaded from Zenodo, DOI: 10.5281/zenodo.5575492.
