# Supplementary material for: Modular Enantioselective Synthesis of cis-Cyclopropanes through Self-Sensitized Stereoselective Photodecarboxylation with Benzothiazolines
Source: ACS Catal. 2021 Oct 18;11(21):13312–9. doi: 10.1021/acscatal.1c03949 (PMC8576787; doi:10.1021/acscatal.1c03949)
Supplement: Supplementary file 1 — cs1c03949_si_001.pdf [file cs1c03949_si_001.pdf]

Supporting Information

Modular Enantioselective Synthesis of *cis*-Cyclopropanes through Self-Sensitized Stereoselective Photo-Decarboxylation with Benzothiazolines

Matteo Costantini, Abraham Mendoza*

Dept. of Organic Chemistry, Arrhenius laboratory, Stockholm University, 106 91 Stockholm (Sweden)

Corresponding author email: abraham.mendoza@su.se

Raw data for this article can be downloaded from Zenodo at:
https://dx.doi.org/10.5281/zenodo.5575493

Contents

General experimental 1
Synthesis of starting materials: 3
Synthesis of 1,3-dioxoisoindolin-2-yl 2-diazo-2-(3,4,5-trimethoxyphenyl)acetate (1v): 3
Optimization of the decarboxylative reduction reaction 4
General procedure A: 4
Synthesis of *cis*-diarylcyclopropanes (4): 7
General procedure B: 7
Synthesis of 1-methyl-4-((1*R*,2*S*)-2-phenylcyclopropyl)benzene (4b): 7
Synthesis of 1-methoxy-4-((1*R*,2*S*)-2-phenylcyclopropyl)benzene (4c): 8
Synthesis of 1-chloro-4-((1*R*,2*S*)-2-phenylcyclopropyl)benzene (4d): 9
Synthesis of 4-((1*R*,2*S*)-2-phenylcyclopropyl)-1,1'-biphenyl (4e): 9
Synthesis of 1-methyl-2-((1*R*,2*S*)-2-phenylcyclopropyl)benzene (4f): 10
Synthesis of 1-bromo-3-((1*R*,2*S*)-2-phenylcyclopropyl)benzene (4g): 11
Synthesis of 1,2-difluoro-4-((1*R*,2*S*)-2-phenylcyclopropyl)benzene (4h): 11
Synthesis of 1-((1*R*,2*S*)-2-phenylcyclopropyl)naphthalene (4i): 12
Synthesis of *tert*-butyl 5-((1*R*,2*S*)-2-phenylcyclopropyl)-1H-indole-1-carboxylate (4j): 13
Synthesis of (1*S*,1a*R*,6a*R*)-1-phenyl-1,1a,6,6a-tetrahydrocyclopropa[1]indene (4k): 13
Synthesis of 1,4-bis((1*R*,2*S*)-2-phenylcyclopropyl)benzene (4l): 14
Synthesis of 4-((1*S*,2*R*)-2-(*p*-tolyl)cyclopropyl)-1,1'-biphenyl (4m): 15
Synthesis of 1-bromo-4-((1*S*,2*R*)-2-(*p*-tolyl)cyclopropyl)benzene (4n): 16
Synthesis of 1-methyl-4-((1*R*,2*S*)-2-(4-(trifluoromethoxy)phenyl)cyclopropyl)benzene (4o): 16
Synthesis of trimethyl((4-((1*S*,2*R*)-2-(*p*-tolyl)cyclopropyl)phenyl)ethynyl)silane (4p): 17
Synthesis of 1-methoxy-3-((1*S*,2*R*)-2-(*p*-tolyl)cyclopropyl)benzene (4q): 18
Synthesis of 2-(3-((1*S*,2*R*)-2-(*p*-tolyl)cyclopropyl)phenyl)acetonitrile (4r): 18
Synthesis of 1-((1*S*,2*R*)-2-(*p*-tolyl)cyclopropyl)-3-(trifluoromethyl)benzene (4s): 19
Synthesis of 1-(4-((1*S*,2*R*)-2-(*p*-tolyl)cyclopropyl)phenyl)ethan-1-one (4t): 20
Synthesis of phenylalanine derivative (4u): 20
Synthesis of 2-methoxy-5-((1*R*,2*S*)-2-(3,4,5-trimethoxyphenyl)cyclopropyl)phenol (4v): 21
Photochemical studies 23
UV-Vis study 23
Job plot 23
Excitation and emission profiles 24
Stern-Volmer quenching experiment 25
Quantum yield measurements 28

Preparation of buffered phenanthroline solution (Solution A) 28
Preparation of potassium ferrioxalate solution (Solution B) 29
Determination of the quantum yield 30
1D-NOE study of the EDA-complex 32
^{1}H NMR of redox-active ester **3a** in DMSO-d_6 32
^{1}H NMR of benzothiazoline **6a** in DMSO-d_6 33
^{1}H NMR of a 1:1 mixture of **6a** and redox-active ester **3a** in DMSO-d_6 34
^{1}H NMR overlay of redox-active ester **3a** (red), **6a** (blue) and their 1:1 mixture (black) in DMSO-d_6 35
^{1}H NMR overlay of redox-active ester **3a** (red), **6a** (blue) and their 1:1 mixture (black) in DMSO-d_6 (superimposed) 36
^{1}H 1D-NOESY of a 1:1 mixture of **6a** and redox-active ester **3a** in DMSO-d_6 (irradiation at 2.21 ppm, 750 ms mixing time) 37
^{1}H 1D-NOESY of a 1:1 mixture of **6a** and redox-active ester **3a** in DMSO-d_6 (irradiation at 6.44 ppm, 750 ms mixing time) 38
^{1}H 1D-NOESY of a 1:1 mixture of **6a** and redox-active ester **3a** in DMSO-d_6 (irradiation at 7.95 ppm, 750 ms mixing time) 39
^{1}H 1D-NOESY of a 1:1 mixture of **6a** and redox-active ester **3a** in DMSO-d_6 (irradiation at 3.35 ppm, 750 ms mixing time) 40
^{1}H 1D-NOESY of a 1:1 mixture of **6a** and redox-active ester **3a** in DMSO-d_6 (irradiation at 6.60 ppm, 500 ms mixing time) 41
Mechanistic experiments 42
Synthesis of 2-phenyl-2,3-dihydrobenzo[5]thiazole-2-d (6a-d_1) 42
N-deuteration of 2-phenylbenzothiazolines 6a-d_2 and 6a-d_1' 42
Synthesis of 1,3-dioxoisoindolin-2-yl 1,2-diphenyl-2-(trimethylsilyl)cyclopropane-1-carboxylate (S3b) 44
Synthesis of 1,3-dioxoisoindolin-2-yl-1,2-diphenylcyclopropane-1-carboxylate (*diast*-3a) 44
Stereo-inversion experiment 45
Deuteration experiments 45
References: 49
NMR spectra of synthesized compounds 50
^{1}H-NMR (400 MHz, $CDCl_3$) for **1v**: 50
^{13}C-NMR (101 MHz, $CDCl_3$) for **1v**: 51
^{1}H-NMR (400 MHz, $CDCl_3$) for **S3b**: 52
^{13}C-NMR (101 MHz, $CDCl_3$) for **S3b**: 53
^{1}H-NMR (400 MHz, $CDCl_3$) for ***diast*-3a**: 54

^{13}C-NMR (101 MHz, $CDCl_3$) for ***diast*-3a**: 55
^{1}H-NMR (400 MHz, $CDCl_3$) for **4b**: 56
^{13}C-NMR (101 MHz, $CDCl_3$) for **4b**: 57
^{1}H-NMR (400 MHz, $CDCl_3$) for **4c**: 58
^{13}C-NMR (101 MHz, $CDCl_3$) for **4c**: 59
^{1}H-NMR (400 MHz, $CDCl_3$) for **4d**: 60
^{13}C-NMR (101 MHz, $CDCl_3$) for **4d**: 61
^{1}H-NMR (400 MHz, $CDCl_3$) for **4e**: 62
^{13}C-NMR (101 MHz, $CDCl_3$) for **4e**: 63
^{1}H-NMR (400 MHz, $CDCl_3$) for **4f**: 64
^{13}C-NMR (101 MHz, $CDCl_3$) for **4f**: 65
^{1}H-NMR (400 MHz, $CDCl_3$) for **4g**: 66
^{13}C-NMR (101 MHz, $CDCl_3$) for **4g**: 67
^{1}H-NMR (400 MHz, $CDCl_3$) for **4h**: 68
^{13}C-NMR (101 MHz, $CDCl_3$) for **4h**: 69
^{19}F-NMR (377 MHz, $CDCl_3$) for **4h**: 70
^{1}H-NMR (400 MHz, $CDCl_3$) for **4i**: 71
^{13}C-NMR (101 MHz, $CDCl_3$) for **4i**: 72
^{1}H-NMR (400 MHz, $CDCl_3$) for **4j**: 73
^{13}C-NMR (101 MHz, $CDCl_3$) for **4j**: 74
^{1}H-NMR (400 MHz, $CDCl_3$) for **4k**: 75
^{13}C-NMR (101 MHz, $CDCl_3$) for **4k**: 76
^{1}H-NMR (400 MHz, $CDCl_3$) for **4l**: 77
^{13}C-NMR (101 MHz, $CDCl_3$) for **4l**: 78
^{1}H-NMR (400 MHz, $CDCl_3$) for **4m**: 79
^{13}C-NMR (101 MHz, $CDCl_3$) for **4m**: 80
^{1}H-NMR (400 MHz, $CDCl_3$) for **4n**: 81
^{13}C-NMR (101 MHz, $CDCl_3$) for **4n**: 82
^{1}H-NMR (400 MHz, $CDCl_3$) for **4o**: 83
^{13}C-NMR (101 MHz, $CDCl_3$) for **4o**: 84
^{19}F-NMR (377 MHz, $CDCl_3$) for **4o**: 85
^{1}H-NMR (400 MHz, $CDCl_3$) for **4p**: 86
^{13}C-NMR (101 MHz, $CDCl_3$) for **4p**: 87
^{1}H-NMR (400 MHz, $CDCl_3$) for **4q**: 88
^{13}C-NMR (101 MHz, $CDCl_3$) for **4q**: 89
^{1}H-NMR (400 MHz, $CDCl_3$) for **4r**: 90
^{13}C-NMR (101 MHz, $CDCl_3$) for **4r**: 91

^{1}H-NMR (400 MHz, $CDCl_3$) for **4s**: 92
^{13}C-NMR (101 MHz, $CDCl_3$) for **4s**: 93
^{19}F-NMR (377 MHz, $CDCl_3$) for **4s**: 94
^{1}H-NMR (400 MHz, $CDCl_3$) for **4t**: 95
^{13}C-NMR (101 MHz, $CDCl_3$) for **4t**: 96
^{1}H-NMR (400 MHz, $CDCl_3$) for **4u**: 97
^{13}C-NMR (101 MHz, $CDCl_3$) for **4u**: 98
^{1}H-NMR (400 MHz, $CDCl_3$) for **4v**: 99
^{13}C-NMR (101 MHz, $CDCl_3$) for **4v**: 100
Chromatography data for enantioenriched compounds 101
SFC trace for **4b**: 101
SFC trace for **4c**: 102
HPLC trace for **4d** 103
HPLC trace for **4e** 104
HPLC trace for **4f**: 105
HPLC trace for **4g**: 106
HPLC trace for **4h**: 107
HPLC trace for **4i**: 108
SFC trace for **4j**: 109
HPLC trace for **4k**: 110
HPLC trace for **4l**: 111
HPLC trace for **4m**: 112
HPLC trace for **4n**: 113
HPLC trace for **4o**: 114
SFC trace for **4p**: 115
SFC trace for **4q**: 116
HPLC trace for **4r**: 117
SFC trace for **4s**: 118
HPLC trace for **4t**: 119
HPLC trace for **4v**: 120

General experimental

Materials. All reactions were carried out using oven-dried glassware under an atmosphere of argon (99.999%). Dry solvents were obtained using a solvent purifier equipped with activated alumina columns. All solvents used in work-up procedures, silica gel column chromatography, HPLC analysis and/or purification were obtained from commercial suppliers and used without further purification. When appropriate, degassing of anhydrous solvent was achieved through three freeze-pump-thaw cycles or by bubbling argon for 30 minutes under sonication. Reagents were obtained from commercially available sources and used as received unless otherwise noted. Diazocompounds **1**, NHPI-DA **7**, olefins **2**, benzothiazolines **6** and catalyst Rh_2(*S*-TPCP)$_4$ were prepared according to literature procedures[1,2]. Racemic cyclopropane products **4** were prepared using $Rh_2(TFA)_4$ as the catalyst and $CHCl_3$ as the reductant (see **Table S1**). Commercially available alkenes were purchased from Sigma-Aldrich, Fluorochem or Acros Organics. Liquid alkenes were distilled, using a Hickman head, prior to use.

Chromatography. Thin layer chromatography (TLC) was carried out on 0.25 mm E. Merck silica plates (60 F_{254}), using UV light as visualizing agent and a vanillin or $KMnO_4$ solution and heat as developing agent. Flash silica gel chromatography was performed using Normasil 60 VWR Chemicals (60 Å, particle size 0.040-0.063 mm). Gas chromatography was performed on a Shimadzu GC-2010 Plus Gas Chromatograph coupled with a GCMS-QP2020 Mass Spectrometer equipped with an FID detector (FID-2010Plus) and an Agilent HP-5MS, 30 m x 0.25 mm fused silica (film = 0.25 µm) capillary column. Determination of Enantiomeric Purity: HPLC analysis on chiral stationary phase was performed on an Agilent 1200-series instrument, employing Daicel Chiralpak columns IA, IB, IF, IG, and OJ-H, or SFC analysis on chiral stationary phase was performed on an Agilent 1260-series instrument, employing Daicel Chiralpak columns IB and IF. The exact conditions for the analyses are specified within the characterization section. Racemic compounds were prepared employing $CHCl_3$ as the hydrogen atom donor.

Characterization. NMR spectra for the characterization of compounds were recorded at room temperature on Bruker instruments: 400 MHz (^{1}H) and at 100 MHz (^{13}C) or 500 MHz (^{1}H) and at 125 MHz (^{13}C). Chemical shifts (δ) are reported in ppm, using the residual solvent peak in $CDCl_3$ (δ_H = 7.26 and δ_C = 77.16 ppm) as internal reference, and coupling constants (*J*) are given in hertz (Hz). Data are reported as follows: chemical shift, multiplicity (s: singlet, d: doublet, t: triplet, q: quartet, br: broad, m: multiplet), coupling constants (*J* in Hz) and integration. Carbon multiplicities were assigned by DEPT and edited HSQC techniques. High-resolution mass spectra (HRMS) were determined with a Bruker Daltonics microTOF Mass Spectrometer with an ESI ionization source, or with a Thermo Scientific Q-Exactive GC-Orbitrap analyzer with an EI ionization source, connected to a Trace 1310 GC (column: Agilent J&W DB-5MS UI, 30 m x 0.25 mm internal diameter, 0.25 µm film) equipped with a PTV injector and a Thermo Triplus RSH autosampler. Specific optical rotation was measured on a

Rudolph Research Analytical Autopol IV polarimeter and is reported as follows: $[\alpha]_D^T$ (concentration in g per 100 mL, solvent).

Experimental details. Reactions were performed in common pyrex round bottom flasks, microwave vials 0.5 - 8 ml (VWR), or 5 - 20 ml flat bottom vials (Cronus, SMI-LabHut Ltd.) crimped on top with 20 mm Sil/PTFE Septa. Reactions were individually irradiated from the bottom using blue LEDs (450 nm) with passive aluminum heat-sink cooling. Reaction temperatures were maintained using an electric fan or an Immersion Cooler (Julabo-FT902) equipped with temperature controller.

Spectrophotometry: UV-vis measurements were carried out on a Varian Cary 50 Bio UV-Visible spectrophotometer equipped with silicon diode detectors and Xe pulse lamp single source. Emission, fluorescence measurements were done using an Edinburgh Instruments FS5 Spectrofluorometer equipped with a 150 W CW Ozone-free Xe arc lamp, Czerny Turner excitation and emission monochromators, and an UV-enhanced silicon diode detector. TCSPC measurements were carried out using an Endinburgh Instruments FS5 Spectrofluorometer equipped with a picosecond pulsed laser (375 nm or 450 nm), Czerny Turner emission monochromator, and a temperature-stabilized high-speed photomultiplier. Raw data was processed with the Fluoracle software.

Synthesis of starting materials:

Synthesis of 1,3-dioxoisoindolin-2-yl 2-diazo-2-(3,4,5-trimethoxyphenyl)acetate (1v):

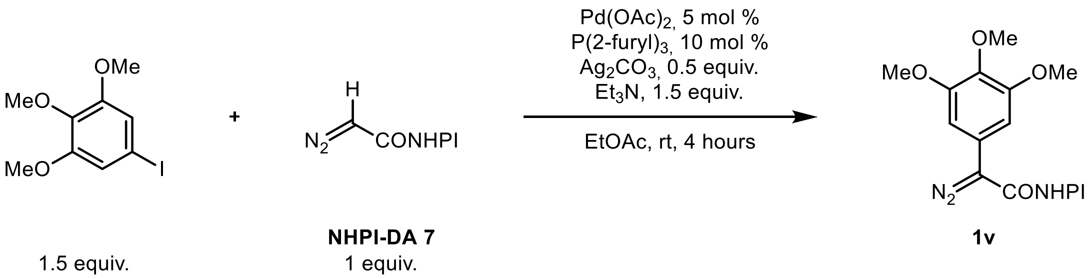

The procedure was adapted from the method by Mendoza and co-workers[1]. To a stirred solution of 5-iodo-1,2,3-trimethoxybenzene **8a**[3] (397.0 mg, 1.35 mmol, 1.50 equiv.), palladium (II) acetate (10.1 mg, 0.045 mmol, 0.05 equiv.), tris(furan-2-yl)phosphane (20.9 mg, 0.09 mmol, 0.10 equiv.), silver carbonate (124.1 mg, 0.45 mmol, 0.50 equiv.) and triethylamine (188 µL, 1.35 mmol, 1.50 equiv.) was added NHPI-DA (208.1 mg, 0.90 mmol, 1.00 equiv.). The mixture was stirred at room temperature for 4 hours, then filtered through a plug of silica. The plug was washed with ethyl acetate and the filtrate concentrated under vacuum. The crude product was purified by column chromatography (pentane:ethyl acetate 7:3) to afford pure product **1v** as an orange solid (264.0 mg, 78%).

Appearance: orange solid.

TLC: R_f: 0.17 (pentane:ethyl acetate 8:2, UV active, stains in vanillin).

^{1}H-NMR: (400 MHz, $CDCl_3$) δ 8.02 – 7.86 (m, 2H), 7.86 – 7.74 (m, 2H), 6.70 (s, 2H), 3.86 (s, 6H), 3.85 (s, 3H).

^{13}C-NMR: (101 MHz, $CDCl_3$) δ 162.3, 161.6, 154.0, 137.2, 135.1, 129.0, 124.2, 118.5, 102.3, 61.1, 56.4 (*Diazo C_q not visible*).

HRMS (ESI): calc'd for $[C_{19}H_{15}N_3O_7+Na]^+$: 420.0802; found: 420.0809.

Optimization of the decarboxylative reduction reaction

General procedure A:

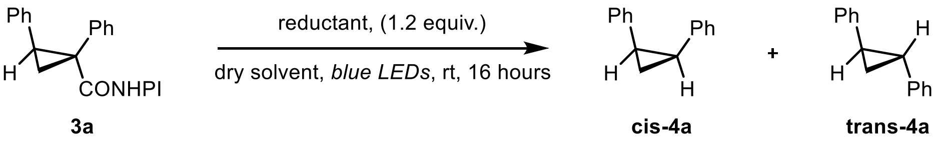

A flask was loaded with redox-active ester **3a**[1] (38.3 mg, 0.10 mmol, 1 equiv.), the indicated reductant (0.12 mmol, 1.2 equiv.) and the indicated dry solvent (1 mL). The mixture was stirred for 16 hours under blue LEDs irradiation with fan cooling. The crude mixture was poured in a separation funnel, diluted with water (15 mL) and ethyl acetate (15 mL). The organic phase was washed with water (3x15 mL) and the water phase was back extracted with ethyl acetate (15 mL). The collected organic phases were dried over Na_2SO_4 and concentrated under vacuum. The yield of the final product **4a** was measured by ^{1}H NMR using 1,1,2,2,-tetrachloroethane as an internal standard. The diastereomeric ratio was determined by GC-MS after filtration of the crude through a plug of silica, eluting with pentane. The results are summarized below in **Table S1**.

***Table S1**: Summary of the optimization of the decarboxylative reduction reaction.*

Entry	*Reductant*	*Solvent*	*Yield (%)*	*d.r. (cis:trans)*	*Conversion (%)*
1	$PhSiH_3$[a]	THF:DMF:*i*-PrOH	30	90:10	> 95
2	$CHCl_3$[b]	$CHCl_3$	43	77:23	> 95
3	**5a**	CH_2Cl_2	41	88:12	90
4	**S5c**	CH_2Cl_2	-	-	20
5	**S5d**	CH_2Cl_2	-	-	< 5
6	**S5e**	CH_2Cl_2	-	-	< 5
7	**S5f**	CH_2Cl_2	22	88:12	40
8	**S5g**	CH_2Cl_2	40	86:14	90
9	**5a**	H_2O	24	89:11	25
10	**5a**	MeCN	60	91:9	> 95
11	**5a**	DMF	74	91:9	> 95
12	**5a**	DMSO	76	90:10	90
13	**5a**	HFIP	60	81:19	> 95
14	**5a**	Acetone	69	91:10	> 95
15	**5a**	$PhCF_3$	29	86:14	50
16	**5a**	EtOAc	53	90:10	> 95
17	**5a**	$MeNO_2$	-	-	25
18	**5b**	DMSO	64	94:6	> 95
19	**6a**	CH_2Cl_2	24	91:9	95

20	**6a**	Acetone	73	92:8	> 95
21	**6a**	MeCN	68	88:12	> 95
22	**6a**	DMF	90	93:6	> 95
23	**6a**	DMSO	88	95:5	> 95
24	**6a**	HFIP	53	84:16	94
25	**6b**	DMSO	81	95:5	> 95
26	**6c**	DMSO	-	-	< 5
27	**6d**	DMSO	92	89:11	> 95
27	**6e**	DMSO	54	88:12	75
29	**6f**	DMSO	44	91:9	50
30	**S6g**	DMSO	-	-	< 5
31	**6a**[c]	DMSO	89	94:6	> 95
32	**6b**[c]	DMSO	81	95:5	> 95
33	**6a**[d]	DMSO	10	97:3	15
34	**6b**[d]	DMSO	-	-	< 5
35	**6a**[e]	DMSO	85	93:7	> 95
36	**6a**[e,f]	DMSO	89	95:5	> 95
37	**6a**[g]	DMSO	58	96:4	> 95
38	**6a**[f,g]	DMSO	59	81:19	> 95

[a]: Performed without light irradiation. Reaction conditions: $PhSiH_3$ (1.5 equiv.), Zn (0.5 equiv.), $NiCl_2(H_2O)_6$ (10 mol%), 4,4'-di-*tert*-butyl-2,2'-bipyridyl (20 mol%), THF:DMF:*i*-PrOH 10:2:1, 40 °C.; [b]: Reaction conditions: Et_3N (2 equiv.), 4CzIPN (2 mol%), $CHCl_3$. [c]: Reaction performed at -10 °C. [d]: Reaction performed in the absence of light. [e]: Performed using DMSO degassed using the freeze-thaw method. [f]: Performed with wet solvent. [g]: Open-flask reaction.

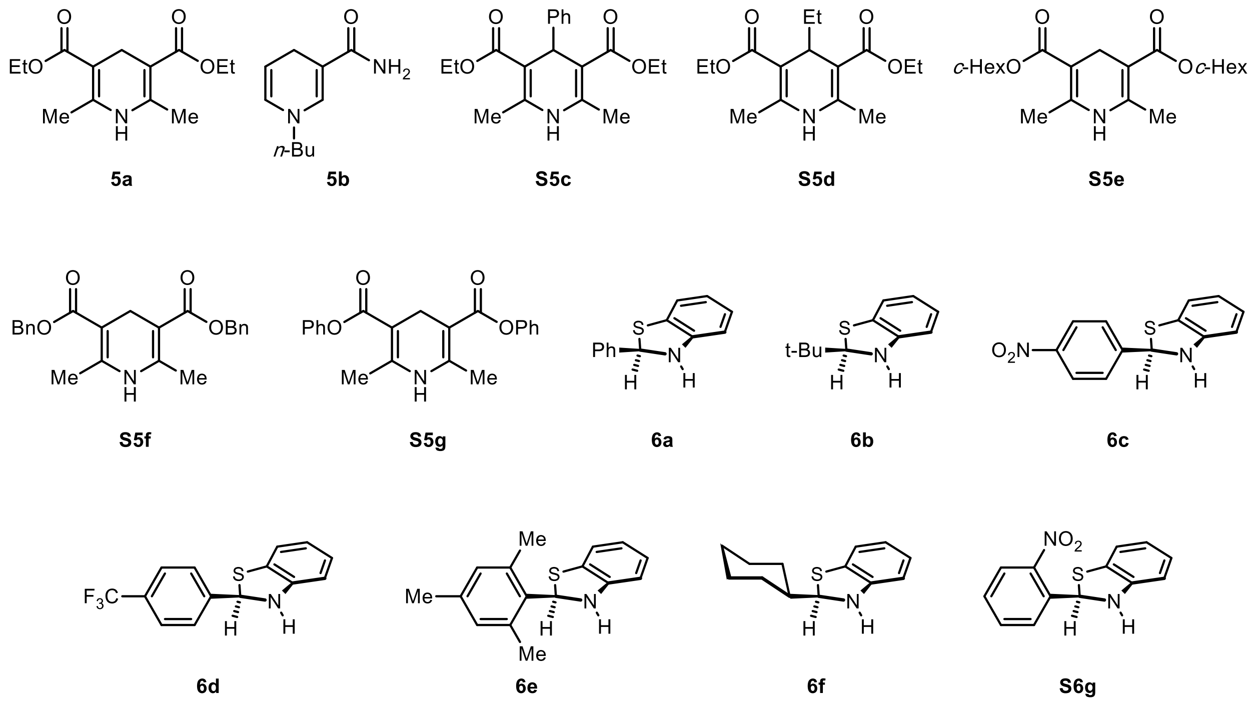

Figure S1: Organic reductants used in the optimization.

Synthesis of *cis*-diarylcyclopropanes (4):

General procedure B:

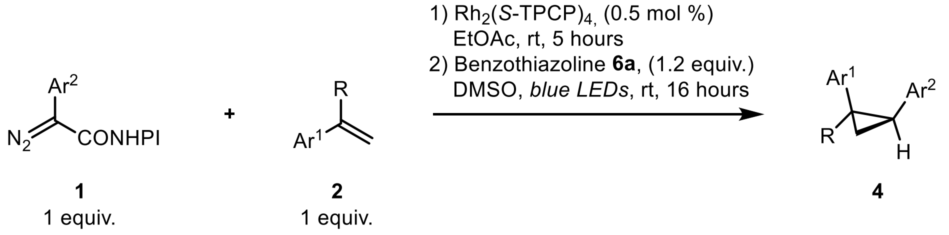

A flask was loaded with Rh_2(*S*-TPCP)$_4$ (2.6 mg, 0.002 mmol, 0.5 mol %) and the olefin substrate **2** (0.350 mmol, 1.0 equiv.). Dry ethyl acetate (7 mL) was added, followed by the NHPI aryldiazoester **1** (0.350 mmol, 1.0 equiv.). The mixture was stirred at room temperature for 5 hours, then the solvent was removed under vacuum. Under inert atmosphere, the crude mixture was loaded with the benzothiazoline reductant **6a** (90 mg, 0.420 mmol, 1.2 equiv.). Dry DMSO (3.5 mL) was then added, and the mixture was stirred for 16 hours under blue LED irradiation with fan cooling. The crude mixture was poured in a separation funnel, diluted with water (15 mL) and ethyl acetate (15 mL). The organic phase was washed with water (3x15 mL) and the water phase was back extracted with ethyl acetate (15 mL). The collected organic phases were dried over Na_2SO_4 and concentrated under vacuum. The final product **4** was purified by flash chromatography.

Synthesis of 1-methyl-4-((1*R*,2*S*)-2-phenylcyclopropyl)benzene (4b):

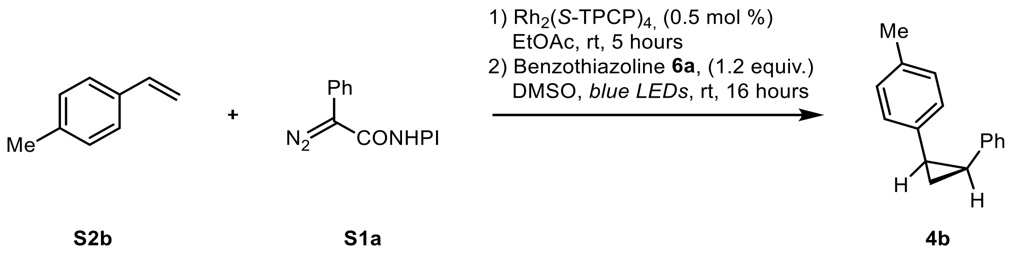

General procedure B was applied employing **S2b** (46 µL, 0.35 mmol, 1.0 equiv.), **S1a** (108 mg, 0.35 mmol, 1.0 equiv.), and **6a** (90 mg, 0.42 mmol, 1.2 equiv.) in the second step. The crude mixture was purified by column chromatography (pentane:diethyl ether, 200:1) to obtain **4b** as a colorless oil (50 mg, 69%; 96:4 dr). Compound (*R*,*S*)-**4b** was obtained with a 96.9:3.1 enantiomeric ratio determined by chiral SFC using a Chiralpak-IF column (MeOH 2%, 1 mL/min): τ_{major}= 10.2 min, τ_{minor}= 9.8 min. Data are in agreement with literature data[4].

Appearance: colorless oil.

TLC: R_f: 0.33 (pentane:diethyl ether 200:1, UV-active).

$[\alpha]_D^{25}$: –25.1 (*c* = 1.0, $CHCl_3$).

^{1}H-NMR: (400 MHz, $CDCl_3$) δ 7.15 – 7.09 (m, 2H), 7.09 – 7.04 (m, 1H), 7.00 – 6.95 (m, 2H), 6.95 – 6.89 (m, 2H), 6.88 – 6.83 (m, 2H), 2.50 – 2.42 (m, 2H), 2.24 (s, 3H), 1.50 – 1.42 (m, 1H), 1.38 – 1.31 (m, 1H).

^{13}C-NMR: (101 MHz, $CDCl_3$) δ 138.7, 135.3, 135.1, 129.1, 129.0, 128.5, 127.8, 125.6, 24.3, 24.2, 21.1, 11.6.

Full characterization can be found at: W. J. Seitz, M. M. Hossain, *Tetrahedron Lett.* **1994**, *35*, 7561-7564.

Synthesis of 1-methoxy-4-((1*R*,2*S*)-2-phenylcyclopropyl)benzene (4c):

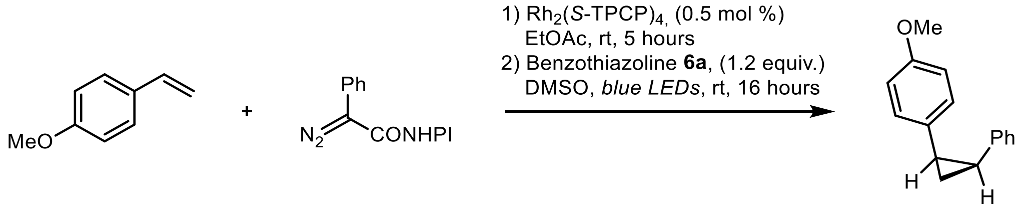

S2c **S1b** **4c**

General procedure B was applied employing **S2c** (47 µL, 0.35 mmol, 1.0 equiv.), **S1b** (108 mg, 0.35 mmol, 1.0 equiv.), and **6a** (90 mg, 0.42 mmol, 1.2 equiv.) in the second step. The crude mixture was purified by column chromatography (pentane:diethyl ether, 200:1) to obtain **4c** as a colorless oil (44 mg, 56%; 96:4 dr). Compound (*R*,*S*)-**4c** was obtained with a 95.5:4.5 enantiomeric ratio determined by chiral SFC using a Chiralpak-IF column (MeOH 10%, 1 mL/min): τ_{major}= 6.0 min, τ_{minor}= 5.7 min. Data are in agreement with literature data[5,6].

Appearance: colorless oil.

TLC: R_f: 0.14 (pentane:diethyl ether 200:1, UV active, weakly stains in vanillin).

$[\alpha]_D^{25}$: –37.5 (*c* = 1.0, $CHCl_3$).

^{1}H-NMR: (400 MHz, $CDCl_3$) δ 7.14 – 7.01 (m, 2H), 6.97 – 6.85 (m, 3H), 6.69 – 6.63 (m, 2H), 3.71 (s, 3H), 2.47 – 2.40 (m, 2H), 1.49 – 1.41 (m, 1H), 1.33 – 1.28 (m, 1H).

^{13}C-NMR: (101 MHz, $CDCl_3$) δ 157.7, 138.8, 130.4, 130.2, 128.9, 127.8, 125.6, 113.3, 55.2, 24.0, 23.9, 11.6.

Full characterization can be found at: É. Lévesque, S. R. Goudreau, A. B. Charette, *Org. Lett.* **2014**, *16*, 1490-1493; M. Verdecchia, C. Tubaro, A. Biffis, *Tetrahedron Lett.* **2011**, *52*, 1136-1139.

Synthesis of 1-chloro-4-((1*R*,2*S*)-2-phenylcyclopropyl)benzene (4d):

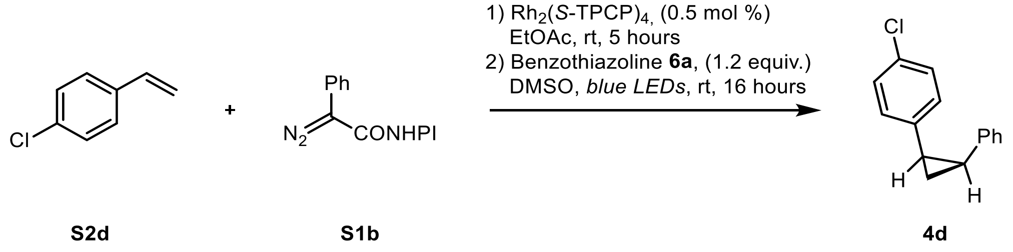

General procedure B was applied employing **S2d** (42 µL, 0.35 mmol, 1.0 equiv.), **S1b** (108 mg, 0.35 mmol, 1.0 equiv.), and **6a** (90 mg, 0.42 mmol, 1.2 equiv.) in the second step. The crude mixture was purified by column chromatography (pentane:diethyl ether, 200:1) to obtain **4d** as a colorless oil (55 mg, 69%; 96:4 dr). Compound (*R*,*S*)-**4d** was obtained with a 96.6:3.4 enantiomeric ratio determined by chiral HPLC using a Chiralpak-IB column (Hexane 100%, 1 mL/min): τ_{major}= 11.7 min, τ_{minor}= 10.9 min. Data are in agreement with literature data[6].

Appearance: colorless oil.

TLC: R_f: 0.36 (pentane:diethyl ether 200:1, UV active).

$[\alpha]_D^{25}$: –22.8 (*c* = 1.0, $CHCl_3$).

^{1}H-NMR: (400 MHz, $CDCl_3$) δ 7.16 – 7.03 (m, 4H), 6.97 – 6.93 (m, 2H), 6.90 – 6.83 (m, 2H), 2.57 – 2.39 (m, 2H), 1.52 – 1.45 (m, 1H), 1.38 – 1.31 (m, 1H).

^{13}C-NMR: (101 MHz, $CDCl_3$) δ 138.0, 137.2, 131.4, 130.3, 129.1, 127.9, 127.9, 125.9, 24.6, 23.8, 11.6.

Full characterization can be found at: M. Verdecchia, C. Tubaro, A. Biffis, *Tetrahedron Lett.* **2011**, *52*, 1136-1139.

Synthesis of 4-((1*R*,2*S*)-2-phenylcyclopropyl)-1,1'-biphenyl (4e):

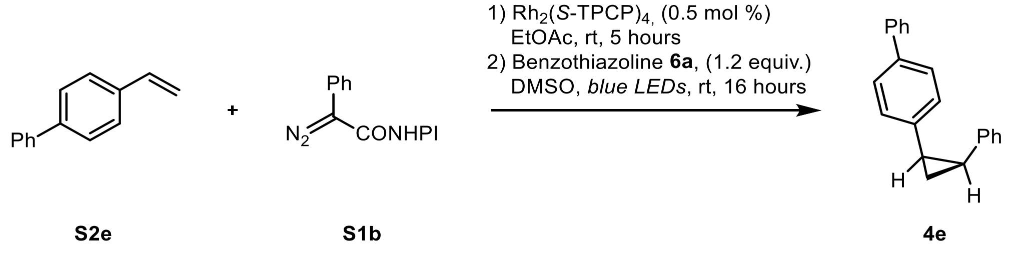

General procedure B was applied employing **S2e** (63 mg, 0.35 mmol, 1.0 equiv.), **S1b** (108 mg, 0.35 mmol, 1.0 equiv.), and **6a** (90 mg, 0.42 mmol, 1.2 equiv.) in the second step. The crude mixture was purified by column chromatography (pentane:diethyl ether, 150:1) to obtain **4e** as a white solid (62 mg, 66%; 96:4 dr). Compound (*R*,*S*)-**4e** was obtained with a 97.6:2.4 enantiomeric ratio determined by chiral HPLC using a Chiralpak-OJ-H column

(Hex:*i*-PrOH 80:20, 0.5 mL/min): τ_{major}= 23.3 min, τ_{minor}= 25.1 min. Data are in agreement with literature data[7].

Appearance: white solid.

TLC: R_f: 0.21 (pentane:diethyl ether 200:1, UV active).

$[\alpha]_D^{25}$: –29.2 (*c* = 1.0, $CHCl_3$).

^{1}H-NMR: (400 MHz, $CDCl_3$*d*) δ 7.59 – 7.55 (m, 2H), 7.46 – 7.31 (m, 3H), 7.21 – 7.02 (m, 5H), 2.63 – 2.52 (m, 2H), 1.60 – 1.51 (m, 1H), 1.50 – 1.42 (m, 1H).

^{13}C-NMR: (101 MHz, $CDCl_3$) δ 141.0, 138.4, 138.4, 137.8, 129.4, 129.2, 128.7, 127.9, 127.0, 126.9, 126.4, 125.8, 24.7, 24.1, 11.9.

Full characterization can be found at: G.-H. Fang, Z.-J. Yan, M.-Z. Deng, *Org. Lett.* **2004**, *6*, 357-360.

Synthesis of 1-methyl-2-((1*R*,2*S*)-2-phenylcyclopropyl)benzene (4f):

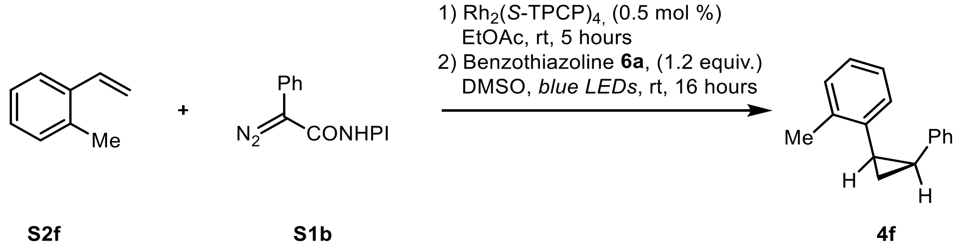

General procedure B was applied employing **S2f** (45 μL, 0.35 mmol, 1.0 equiv.), **S1b** (108 mg, 0.35 mmol, 1.0 equiv.), and **6a** (90 mg, 0.42 mmol, 1.2 equiv.) in the second step. The crude mixture was purified by column chromatography (pentane:diethyl ether, 200:1) to obtain **4f** as a colorless oil (44 mg, 60%; 94:6 dr). Compound (*R*,*S*)-**4f** was obtained with a 97.2:2.8 enantiomeric ratio determined by chiral HPLC using a Chiralpak-OJ-H column (Hexane 100%, 1 mL/min): τ_{major}= 12.9 min, τ_{minor}= 11.5 min. Data are in agreement with literature data[8].

Appearance: colorless oil.

TLC: R_f: 0.35 (pentane:diethyl ether 200:1, UV active).

$[\alpha]_D^{25}$: +12.3 (*c* = 1.0, $CHCl_3$).

^{1}H-NMR: (400 MHz, $CDCl_3$) δ 7.25 – 7.16 (m, 1H), 7.13 – 6.97 (m, 6H), 6.86 – 6.81 (m, 2H), 2.55 – 2.42 (m, 2H), 2.18 (s, 3H), 1.57 – 1.44 (m, 2H).

^{13}C-NMR: (101 MHz, $CDCl_3$) δ 139.2, 138.9, 136.0, 129.5, 129.2, 127.5, 127.4, 126.3, 125.4, 125.4, 24.7, 23.5, 19.8, 11.6.

Full characterization can be found at: Q. Wang, F. H. Försterling, M. M. Hossain, *J. Organomet. Chem.* **2005**, *690*, 6238-6246.

Synthesis of 1-bromo-3-((1*R*,2*S*)-2-phenylcyclopropyl)benzene (4g):

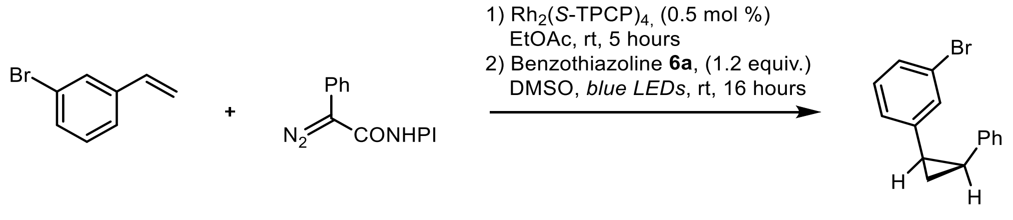

S2g **S1b** **4g**

General procedure B was applied employing **S2g** (45 µL, 0.35 mmol, 1.0 equiv.), **S1b** (108 mg, 0.35 mmol, 1.0 equiv.), and **6a** (90 mg, 0.42 mmol, 1.2 equiv.) in the second step. The crude mixture was purified by column chromatography (pentane:diethyl ether, 200:1) to obtain **4g** as a colorless oil (34 mg, 36%; 95:5 dr). Compound (*R*,*S*)-**4g** was obtained with a 97.0:3.0 enantiomeric ratio determined by chiral HPLC using a Chiralpak-IA column (Hexane 100%, 1 mL/min): τ_{major}= 6.6 min, τ_{minor}= 7.2 min.

Appearance: colorless oil.

TLC: R_f: 0.30 (pentane:diethyl ether 200:1, UV active, weakly stains in vanillin).

$[\alpha]_D^{25}$: –25.6 (*c* = 1.0, $CHCl_3$).

^{1}H-NMR: (400 MHz, $CDCl_3$) δ 7.19 – 7.04 (m, 5H), 6.99 – 6.89 (m, 3H), 6.81 – 6.76 (m, 1H), 2.57 – 2.49 (m, 1H), 2.47 – 2.39 (m, 1H), 1.52 – 1.44 (m, 1H), 1.41 – 1.34 (m, 1H).

^{13}C-NMR: (101 MHz, $CDCl_3$) δ 141.2, 137.7, 132.1, 129.2, 129.1, 128.8, 127.9, 127.5, 126.0, 121.9, 24.8, 24.0, 11.5.

HRMS (EI): calc'd for $[C_{15}H_{13}Br]^{+\cdot}$: 272.0195; found: 272.0195.

Synthesis of 1,2-difluoro-4-((1*R*,2*S*)-2-phenylcyclopropyl)benzene (4h):

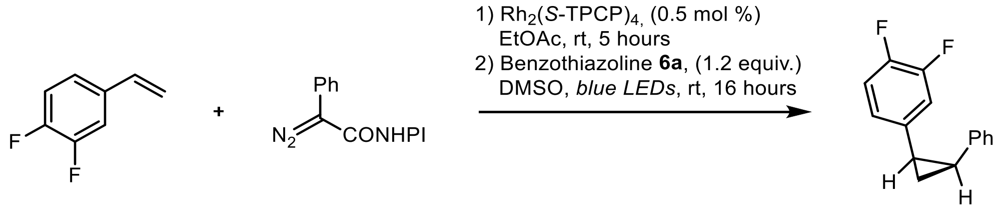

S2h **S1b** **4h**

General procedure B was applied employing **S2h** (49 mg, 0.35 mmol, 1.0 equiv.), **S1b** (108 mg, 0.35 mmol, 1.0 equiv.), and **6a** (90 mg, 0.42 mmol, 1.2 equiv.) in the second step. The crude mixture was purified by column chromatography (pentane:diethyl ether, 200:1) to obtain **4h** as a colorless oil (59 mg, 73%; 88:12 dr). Compound (*R*,*S*)-**4h** was obtained with a

96.6:3.4 enantiomeric ratio determined by chiral HPLC using a Chiralpak-IB column (Hexane 100%, 1 mL/min): τ_{major}= 11.3 min, τ_{minor}= 10.3 min.

Appearance: colorless oil.

TLC: R_f: 0.46 (pentane:diethyl ether 200:1, UV active, weakly stains in vanillin).

$[\alpha]_D^{25}$: –20.6 (*c* = 1.0, $CHCl_3$).

^{1}H-NMR: (400 MHz, $CDCl_3$) δ 7.18 – 7.04 (m, 3H), 6.97 – 6.92 (m, 2H), 6.87 (dt, *J* = 10.4, 8.4 Hz, 1H), 6.77 – 6.69 (m, 1H), 6.69 – 6.62 (m, 1H), 2.54 – 2.46 (m, 1H), 2.47 – 2.38 (m, 1H), 1.51 – 1.43 (m, 1H), 1.36 – 1.30 (m, 1H).

^{13}C-NMR: (101 MHz, $CDCl_3$) δ 150.5 (dd, *J* = 114.4, 12.8 Hz), 148.0 (dd, *J* = 113.2, 12.7 Hz), 137.7, 135.7 (dd, *J* = 6.0, 3.7 Hz), 129.0, 128.0, 126.1, 124.9 (dd, *J* = 6.1, 3.4 Hz), 117.7 (d, *J* = 17.2 Hz), 116.4 (d, *J* = 17.1 Hz), 24.5, 23.6, 11.5.

^{19}F-NMR: (377 MHz, $CDCl_3$) δ –139.09 (d, *J* = 21.6 Hz), –142.21 (d, *J* = 21.3 Hz).

HRMS (EI): calc'd for $[C_{15}H_{12}F_2]^{+\cdot}$: 230.0902; found: 230.0906.

Synthesis of 1-((1*R*,2*S*)-2-phenylcyclopropyl)naphthalene (4i):

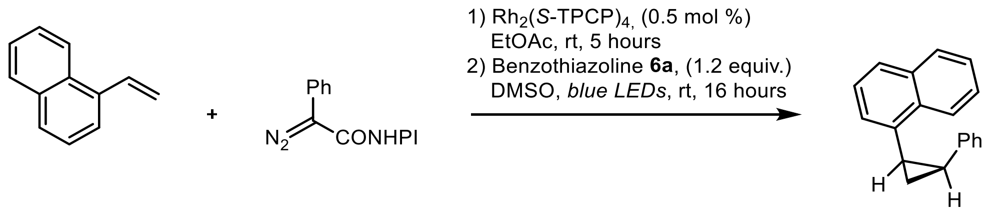

S1i **S1b** **4i**

General procedure B was applied employing **S2i** (54 mg, 0.35 mmol, 1.0 equiv.), **S1b** (108 mg, 0.35 mmol, 1.0 equiv.), and **6a** (90 mg, 0.42 mmol, 1.2 equiv.) in the second step. The crude mixture was purified by column chromatography (pentane:diethyl ether, 150:1) to obtain **4i** as a white solid (50 mg, 59%; 94:6 dr). Compound (*R*,*S*)-**4i** was obtained with a 96.9:3.1 enantiomeric ratio determined by chiral HPLC using a Chiralpak-OJ-H column (Hex:*i*-PrOH 90:10, 1 mL/min): τ_{major}= 13.6 min, τ_{minor}= 10.2 min.

Appearance: white solid.

TLC: R_f: 0.19 (pentane:diethyl ether 200:1, UV active).

$[\alpha]_D^{25}$: +316.0 (*c* = 1.0, $CHCl_3$).

^{1}H-NMR: (400 MHz, $CDCl_3$) δ 8.32 – 8.27 (m, 1H), 7.78 – 7.73 (m, 1H), 7.68 – 7.61 (m, 1H), 7.49 – 7.37 (m, 2H), 7.37 – 7.28 (m, 2H), 6.96 – 6.84 (m, 5H), 2.95 – 2.86 (m, 1H), 2.77 – 2.67 (m, 1H), 1.72 – 1.62 (m, 2H).

^{13}C-NMR: (101 MHz, $CDCl_3$) δ 138.7, 134.2, 133.7, 133.5, 128.3, 127.6, 127.4, 126.9, 126.7, 125.5, 125.5, 125.4, 125.1, 124.7, 24.1, 23.5, 11.0.

HRMS (EI): calc'd for $[C_{19}H_{16}]^{+\cdot}$: 244.1247; found: 244.1250.

Synthesis of *tert*-butyl 5-((1*R*,2*S*)-2-phenylcyclopropyl)-1H-indole-1-carboxylate (4j):

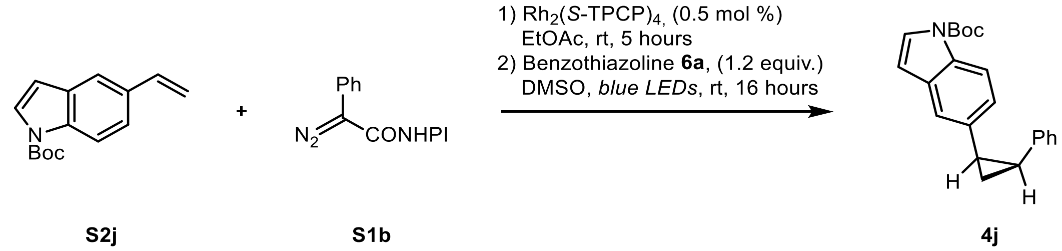

General procedure B was applied employing **S2j** (103 mg, 0.35 mmol, 1.0 equiv.), **S1b** (108 mg, 0.35 mmol, 1.0 equiv.), and **6a** (90 mg, 0.42 mmol, 1.2 equiv.) in the second step. The crude mixture was purified by column chromatography (pentane:ethyl acetate, 100:1) to obtain **4j** as a colorless oil (58 mg, 50%; 94:6 dr). Compound (*R*,*S*)-**4j** was obtained with a 95.4:4.6 enantiomeric ratio determined by chiral SFC using a Chiralpak-OJ-H column (MeOH 20%, 1 mL/min): τ_{major}= 8.7 min, τ_{minor}= 8.1 min.

Appearance: white solid.

TLC: R_f: 0.29 (pentane:ethyl acetate 100:1, UV active, stains in vanillin).

$[\alpha]_D^{25}$: –36.6 (c = 1.0, $CHCl_3$).

^{1}H-NMR: (400 MHz, $CDCl_3$) δ 7.85 (d, J = 8.6 Hz, 1H), 7.49 (d, J = 3.7 Hz, 1H), 7.20 – 7.16 (m, 1H), 7.09 – 7.03 (m, 2H), 7.03 – 6.97 (m, 1H), 6.97 – 6.91 (m, 3H), 6.40 (dd, J = 3.7, 0.7 Hz, 1H), 2.64 – 2.55 (m, 1H), 2.52 – 2.44 (m, 1H), 1.64 (s, 9H), 1.54 – 1.46 (m, 1H), 1.45 – 1.38 (m, 1H).

^{13}C-NMR: (101 MHz, $CDCl_3$) δ 149.9, 138.8, 132.6, 130.4, 128.8, 128.5, 127.8, 126.0, 125.8, 125.5, 121.3, 114.4, 107.3, 83.5, 28.3, 24.7, 24.3, 11.8.

HRMS (ESI): calc'd for $[C_{22}H_{23}NO_2+Na]^+$: 356.1621; found: 256.1628.

Synthesis of (1*S*,1a*R*,6a*R*)-1-phenyl-1,1a,6,6a-tetrahydrocyclopropa[1]indene (4k):

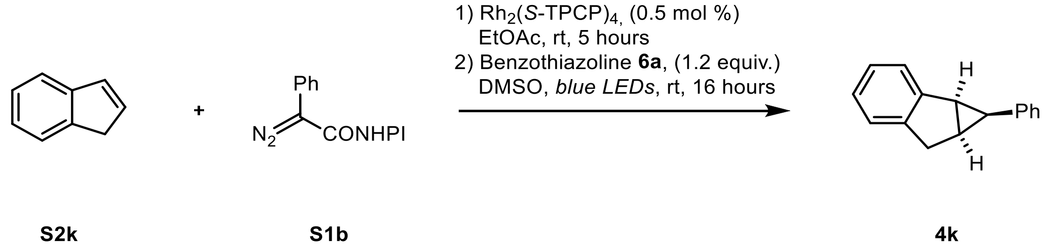

General procedure B was applied employing **S2k** (103 mg, 0.35 mmol, 1.0 equiv.), **S1b** (108 mg, 0.35 mmol, 1.0 equiv.), and **6a** (90 mg, 0.42 mmol, 1.2 equiv.) in the second step. The crude mixture was purified by column chromatography (pentane:diethyl ether, 150:1) to obtain **4k** as a colorless oil (22 mg, 31%; 89:11 dr), together with an inseparable impurity. Compound (*S*,*R*,*R*)-**4k** was obtained with a 94.1:5.9 enantiomeric ratio determined by chiral HPLC using a Chiralpak-IB column (Hexane 100%, 1 mL/min): τ_{major}= 10.2 min, τ_{minor}= 9.7 min. Data are in agreement with literature data[5].

Appearance: colorless oil.

TLC: R_f: 0.38 (pentane:ethyl acetate 100:1, UV active, weakly stains in vanillin).

$[\alpha]_D^{25}$: +6.4 (c = 0.5, $CHCl_3$).

^{1}H-NMR: (400 MHz, $CDCl_3$) δ 7.40 – 7.36 (m, 1H), 7.12 – 6.94 (m, 6H), 6.92 (td, *J* = 7.5, 1.2 Hz, 1H), 6.77 (d, *J* = 7.5 Hz, 1H), 3.17 – 3.09 (m, 1H), 2.97 – 2.91 (m, 1H), 2.75 – 2.67 (m, 1H), 2.46 (t, *J* = 8.3 Hz, 1H), 2.31 – 2.24 (m, 1H).

^{13}C-NMR: (101 MHz, $CDCl_3$) δ 143.3, 143.2, 131.1, 129.0, 127.6, 125.9, 125.7, 125.6, 124.7, 124.4, 32.0, 30.0, 27.2, 22.3.

Full characterization can be found at: É. Lévesque, S. R. Goudreau, A. B. Charette, *Org. Lett.* **2014**, *16*, 1490-1493.

Synthesis of 1,4-bis((1*R*,2*S*)-2-phenylcyclopropyl)benzene (4l):

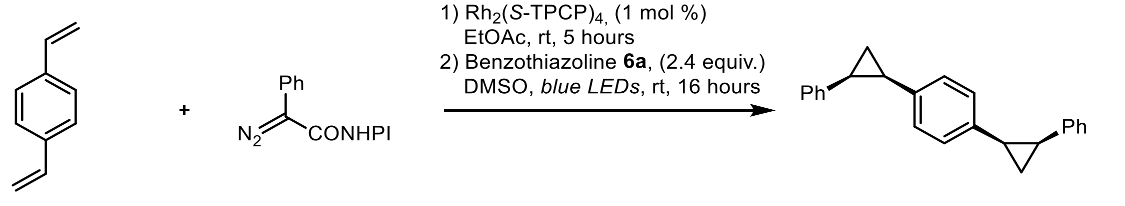

S2l **S1b** **4l**

General procedure B was applied employing **S2l** (23 mg, 0.175 mmol, 1.0 equiv.), **S1b** (108 mg, 0.35 mmol, 2.0 equiv.), Rh_2(*S*-TPCP) (2.6 mg, 0.002 mmol, 0.01 equiv.), and **6a** (90 mg, 0.42 mmol, 2.4 equiv.) in the second step. The crude mixture was purified by column chromatography (pentane:dichloromethane, 15:1) to obtain **4l** as a white crystal (22 mg, 40%; 93:7 dr). Compound (*R*,*S*,*R*,*S*)-**4l** was obtained in a 99.4:0.6 enantiomeric ratio determined by chiral HPLC using a Chiralpak-IA column (Hexane:*i*-PrOH 90:10, 1 mL/min): τ_{major}= 3.8 min, τ_{minor}= 4.1 min.

Appearance: White crystal.

TLC: R_f: 0.35 (pentane:dichloromethane 15:1, UV active).

$[\alpha]_D^{25}$: +30.5 (c = 0.6, $CHCl_3$).

^{1}H-NMR: (400 MHz, $CDCl_3$) δ 7.10 – 7.00 (m, 6H), 6.88 – 6.79 (m, 4H), 6.71 (s, 4H), 2.43 – 2.34 (m, 4H), 1.43 – 1.35 (m, 2H), 1.29 – 1.22 (m, 2H).

^{13}C-NMR: (101 MHz, $CDCl_3$) δ 138.7, 135.6, 128.9, 128.6, 127.7, 125.5, 24.2, 24.2, 11.6.

HRMS (EI): calc'd for $[C_{24}H_{22}]^{+\cdot}$: 310.1716; found: 310.1716.

Synthesis of 4-((1*S*,2*R*)-2-(*p*-tolyl)cyclopropyl)-1,1'-biphenyl (4m):

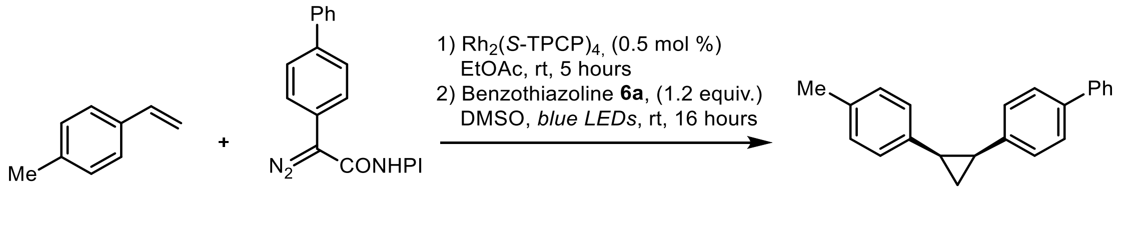

S2b **S1c** **4m**

General procedure B was applied employing **S2b** (46 µL, 0.35 mmol, 1.0 equiv.), **S1c** (134 mg, 0.35 mmol, 1.0 equiv.), and **6a** (90 mg, 0.42 mmol, 1.2 equiv.) in the second step. The crude mixture was purified by column chromatography (pentane:dichloromethane, 15:1) to obtain **4m** as a white solid (42 mg, 42%; 98:2 dr). Compound (*S,R*)-**4m** was obtained in a 97.8:2.2 enantiomeric ratio determined by chiral HPLC using a Chiralpak-IB column (Hexane:*i*-PrOH 95:5, 1 mL/min): τ_{major}= 5.4 min, τ_{minor}= 7.5 min.

Appearance: White solid.

TLC: R_f: 0.32 (pentane:dichloromethane 15:1, UV active).

$[\alpha]_D^{25}$: –11.0 (*c* = 1.0, $CHCl_3$).

^{1}H-NMR: (400 MHz, $CDCl_3$) δ 7.59 – 7.51 (m, 2H), 7.45 – 7.37 (m, 4H), 7.36 – 7.28 (m, 1H), 7.05 (d, *J* = 8.3 Hz, 2H), 6.95 (q, *J* = 8.2 Hz, 4H), 2.56 – 2.46 (m, 2H), 2.26 (s, 3H), 1.56 – 1.47 (m, 1H), 1.44 – 1.37 (m, 1H).

^{13}C-NMR: (101 MHz, $CDCl_3$) δ 141.1, 138.2, 138.1, 135.2, 135.2, 129.4, 129.1, 128.8, 128.6, 127.0, 126.9, 126.4, 24.4, 24.0, 21.1, 11.9.

HRMS (EI): calc'd for $[C_{22}H_{20}]^{+\cdot}$: 284.1560; found: 284.1561.

Synthesis of 1-bromo-4-((1*S*,2*R*)-2-(*p*-tolyl)cyclopropyl)benzene (4n):

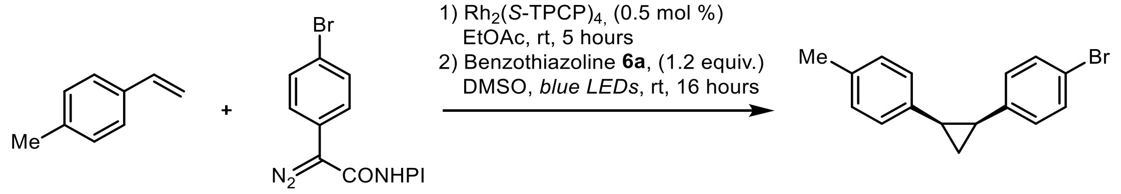

S2b **S1d** **4n**

General procedure B was applied employing **S2b** (46 µL, 0.35 mmol, 1.0 equiv.), **S1d** (135 mg, 0.35 mmol, 1 equiv.), and **6a** (90 mg, 0.42 mmol, 1.2 equiv.) in the second step. The crude mixture was purified by column chromatography (pentane:dichloromethane, 15:1) to obtain **4n** as a colorless oil (65 mg, 65%; 97:3 dr). Compound (*S,R*)-**4n** was obtained in a 93.9:6.1 enantiomeric ratio determined by chiral HPLC using a Chiralpak-IB column (Hexane 100%, 1 mL/min): τ_{major}= 12.1 min, τ_{minor}= 13.4 min.

Appearance: White solid.

TLC: R_f: 0.45 (pentane:dichloromethane 15:1, UV active, weakly stains in vanillin).

$[\alpha]_D^{25}$: −12.7 (*c* = 1.0, $CHCl_3$).

^{1}H-NMR: (400 MHz, $CDCl_3$) δ 7.23 – 7.16 (m, 2H), 6.92 (d, *J* = 7.9 Hz, 2H), 6.86 – 6.77 (m, 4H), 2.50 – 2.42 (m, 1H), 2.41 – 2.33 (m, 1H), 2.24 (s, 3H), 1.49 – 1.41 (m, 1H), 1.33 – 1.25 (m, 1H).

^{13}C-NMR: (101 MHz, $CDCl_3$) δ 138.0, 135.4, 134.7, 130.8, 130.7, 129.0, 128.7, 119.4, 24.3, 23.7, 21.1, 11.7.

HRMS (EI): calc'd for $[C_{16}H_{15}Br]^{+\cdot}$: 286.0352; found: 286.0353.

Synthesis of 1-methyl-4-((1*R*,2*S*)-2-(4-(trifluoromethoxy)phenyl)cyclopropyl)benzene (4o):

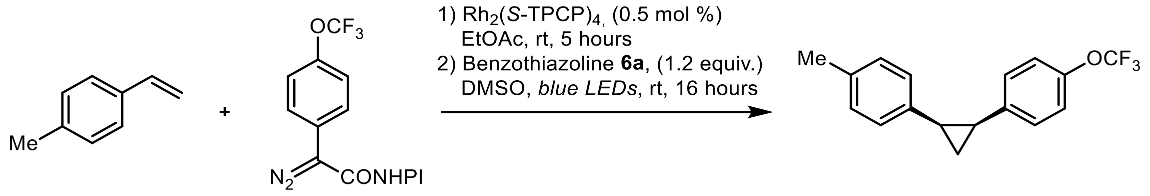

S2b **S1e** **4o**

General procedure B was applied employing **S2b** (46 µL, 0.35 mmol, 1.0 equiv.), **S1e** (137 mg, 0.35 mmol, 1.0 equiv.), and **6a** (90 mg, 0.42 mmol, 1.2 equiv.) in the second step. The crude mixture was purified by column chromatography (pentane:ethyl acetate, 15:1) to obtain **4o** as a colorless oil (62 mg, 61%; 96:4 dr). Compound (*R,S*)-**4o** was obtained in a 96.3:3.7 enantiomeric ratio determined by chiral HPLC using a Chiralpak-IA column (Hexane 100%, 1 mL/min): τ_{major}= 5.4 min, τ_{minor}= 5.2 min.

Appearance: Colorless oil.

TLC: R_f: 0.63 (pentane:ethyl acetate 15:1, UV active, stains in vanillin).

$[\alpha]_D^{25}$: –15.9 (c = 1.0, $CHCl_3$).

^{1}H-NMR: (400 MHz, $CDCl_3$) δ 7.10 – 7.02 (m, 6H), 6.95 (d, J = 8.0 Hz, 2H), 2.64 – 2.48 (m, 2H), 2.36 (s, 3H), 1.64 – 1.55 (m, 1H), 1.46 – 1.39 (m, 1H).

^{13}C-NMR: (101 MHz, $CDCl_3$) δ 147.3, 147.3, 147.3, 147.3, 137.7, 135.4, 134.8, 130.2, 129.0, 128.7, 124.4, 120.6 (q, J = 256.5 Hz), 24.2, 23.5, 21.1, 11.8.

^{19}F-NMR: (377 MHz, $CDCl_3$) δ –57.92.

HRMS (EI): calc'd for $[C_{17}H_{15}OF_3]^{+\cdot}$: 292.1070; found: 292.1069.

Synthesis of trimethyl((4-((1*S*,2*R*)-2-(*p*-tolyl)cyclopropyl)phenyl)ethynyl)silane (4p):

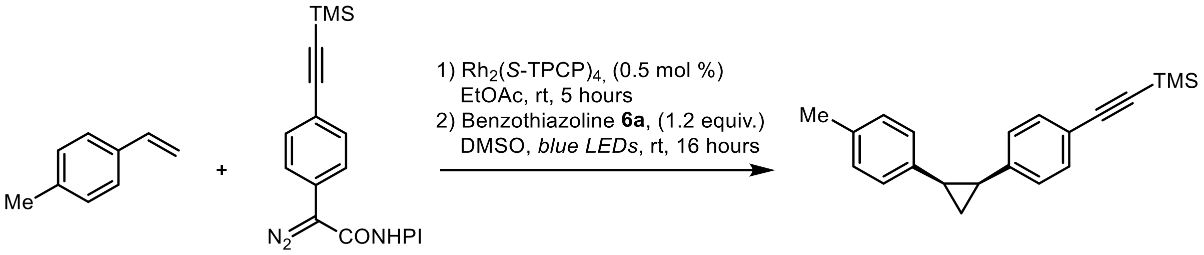

S2b **S1f** **4p**

General procedure B was applied employing **S2b** (46 µL, 0.35 mmol, 1.0 equiv.), **S1f** (141 mg, 0.35 mmol, 1 equiv.), and **6a** (90 mg, 0.42 mmol, 1.2 equiv.) in the second step. The crude mixture was purified by column chromatography (pentane:dichloromethane, 15:1) to obtain **4p** as a colorless oil (70 mg, 66%; 97:3 dr). Compound (*S,R*)-**4p** was obtained in a 95.5:4.5 enantiomeric ratio determined by chiral SFC using a Chiralpak-IB column (MeOH 5%, 1 mL/min): τ_{major}= 6.7 min, τ_{minor}= 7.2 min.

Appearance: Colorless oil.

TLC: R_f: 0.37 (pentane:dichloromethane 15:1, UV active, stains in vanillin).

$[\alpha]_D^{25}$: –21.4 (c = 1.0, $CHCl_3$).

^{1}H-NMR: (400 MHz, $CDCl_3$) δ 7.25 – 7.19 (m, 2H), 6.92 (d, J = 7.8 Hz, 2H), 6.90 – 6.81 (m, 4H), 2.55 – 2.37 (m, 2H), 2.24 (s, 3H), 1.51 – 1.42 (m, 1H), 1.40 – 1.33 (m, 1H), 0.24 (s, 9H).

^{13}C-NMR: (101 MHz, $CDCl_3$) δ 139.8, 135.3, 134.7, 131.5, 129.1, 128.7, 128.7, 120.0, 105.6, 93.5, 24.7, 24.2, 21.1, 11.6, 0.1.

HRMS (EI): calc'd for $[C_{21}H_{24}Si]^{+\cdot}$: 304.1642; found: 304.1641.

Synthesis of 1-methoxy-3-((1*S*,2*R*)-2-(*p*-tolyl)cyclopropyl)benzene (4q):

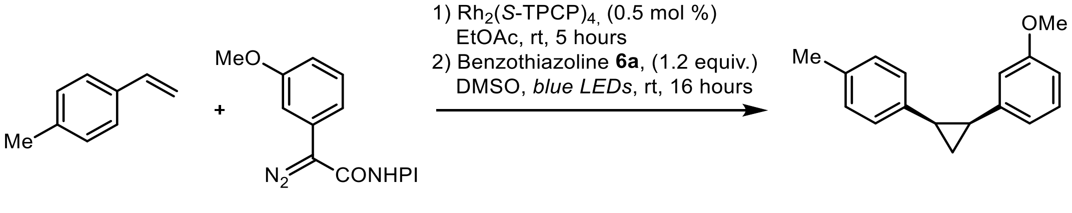

General procedure B was applied employing **S2b** (46 µL, 0.35 mmol, 1.0 equiv.), **S1g** (118 mg, 0.35 mmol, 1.0 equiv.), and **6a** (90 mg, 0.42 mmol, 1.2 equiv.) in the second step. The crude mixture was purified by column chromatography (pentane:dichloromethane, 8:2) to obtain **4q** as a colorless oil (24 mg, 29%; 94:6 dr). Compound (*S,R*)-**4q** was obtained in a 97.4:2.6 enantiomeric ratio determined by chiral SFC using a Chiralpak-OJ-H column (MeOH 5%, 1 mL/min): τ_{major}= 16.6 min, τ_{minor}= 16.0 min.

Appearance: Colorless oil.

TLC: R_f: 0.28 (pentane:dichloromethane 8:2, UV active, stains in vanillin).

$[\alpha]_D^{25}$: –19.5 (*c* = 0.8, $CHCl_3$).

^{1}H-NMR: (400 MHz, $CDCl_3$) δ 7.05 – 6.99 (m, 1H), 6.96 – 6.91 (m, 2H), 6.91 – 6.86 (m, 2H), 6.64 – 6.56 (m, 2H), 6.48 – 6.43 (m, 1H), 3.63 (s, 3H), 2.52 – 2.38 (m, 2H), 2.24 (s, 3H), 1.50 – 1.42 (m, 1H), 1.36 – 1.30 (m, 1H).

^{13}C-NMR: (101 MHz, $CDCl_3$) δ 159.1, 140.5, 135.3, 135.2, 129.1, 128.6, 128.6, 121.7, 114.3, 111.5, 55.1, 24.3, 24.3, 21.1, 11.8.

HRMS (EI): calc'd for $[C_{17}H_{18}O]^{+\cdot}$: 238.1352; found: 238.1352.

Synthesis of 2-(3-((1*S*,2*R*)-2-(*p*-tolyl)cyclopropyl)phenyl)acetonitrile (4r):

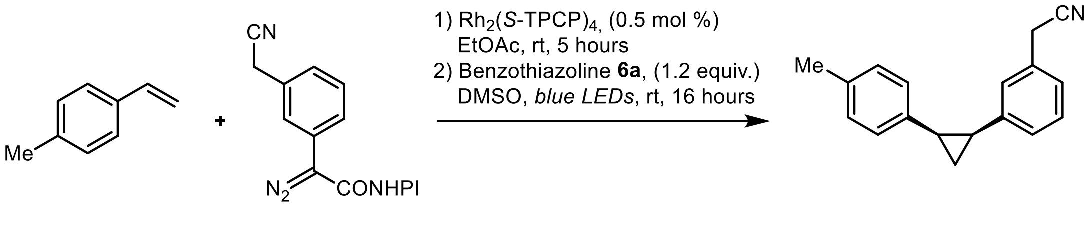

General procedure B was applied employing **S2b** (46 µL, 0.35 mmol, 1.0 equiv.), **S1h** (121 mg, 0.35 mmol, 1.0 equiv.), and **6a** (90 mg, 0.42 mmol, 1.2 equiv.) in the second step. The crude mixture was purified by column chromatography (pentane:ethyl acetate, 15:1) to obtain **6a** as a colorless oil (43 mg, 50%; 95:5 dr). Compound (*S,R*)-**6a** was obtained in a 92.7:7.3

enantiomeric ratio determined by chiral HPLC using a Chiralpak-AS-H column (Hexane:*i*-PrOH 80:20, 1 mL/min): τ_{major}= 7.5 min, τ_{minor}= 7.9 min.

Appearance: Colorless oil.

TLC: R_f: 0.33 (pentane:ethyl acetate 15:1; UV active, stains in vanillin).

$[\alpha]_D^{25}$: –24.2 (*c* = 1.0, $CHCl_3$).

^{1}H-NMR: (400 MHz, $CDCl_3$) δ 7.11 – 7.06 (m, 1H), 7.01 – 6.96 (m, 1H), 6.95 – 6.81 (m, 6H), 3.57 (s, 3H), 2.53 – 2.39 (m, 2H), 2.22 (s, 3H), 1.51 – 1.43 (m, 1H), 1.38 – 1.31 (m, 1H).

^{13}C-NMR: (101 MHz, $CDCl_3$) δ 140.1, 135.3, 134.7, 129.2, 129.0, 128.6, 128.5, 128.5, 128.4, 125.1, 118.0, 24.4, 23.9, 23.5, 21.1, 11.5.

HRMS (ESI): calc'd for $[C_{18}H_{17}N+Na]^+$: 270.1253; found: 270.1252.

Synthesis of 1-((1*S*,2*R*)-2-(*p*-tolyl)cyclopropyl)-3-(trifluoromethyl)benzene (4s):

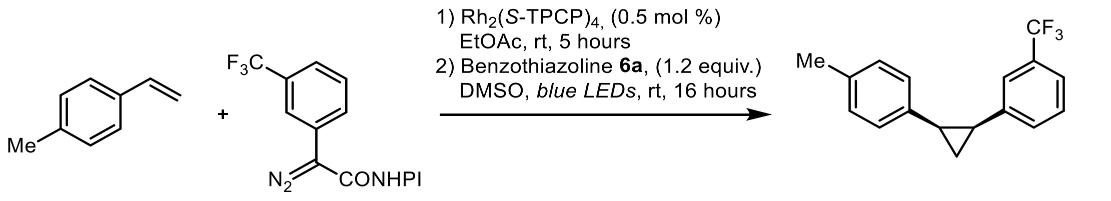

S2b **S1i** **4s**

General procedure B was applied employing **S2b** (46 µL, 0.35 mmol, 1.0 equiv.), **S1i** (131 mg, 0.35 mmol, 1.0 equiv.), and **6a** (90 mg, 0.42 mmol, 1.2 equiv.) in the second step. The crude mixture was purified by column chromatography (pentane:dichloromethane, 15:1) to obtain **4s** as a colorless oil (46 mg, 48%; 96:4 dr). Compound (*S,R*)-**4s** was obtained in a 92.1:7.9 enantiomeric ratio determined by chiral SFC using a Chiralpak-OJ-H column (MeOH 5%, 1 mL/min): τ_{major}= 5.0 min, τ_{minor}= 4.6 min.

Appearance: Colorless oil.

TLC: R_f: 0.60 (pentane:dichloromethane 15:1, UV active, weakly stains in vanillin).

$[\alpha]_D^{25}$: –16.2 (*c* = 1.0, $CHCl_3$).

^{1}H-NMR: (400 MHz, $CDCl_3$) δ 7.34 – 7.28 (m, 1H), 7.25 – 7.13 (m, 2H), 7.09 – 7.03 (m, 1H), 6.97 – 6.91 (m, 2H), 6.89 – 6.84 (m, 2H), 2.60 – 2.44 (m, 2H), 2.24 (s, 3H), 1.56 – 1.48 (m, 1H), 1.44 – 1.37 (m, 1H).

^{13}C-NMR: (101 MHz, $CDCl_3$) δ 140.0, 135.6, 134.3, 132.08 (d, J = 1.8 Hz), 130.0 (q, *J* = 31.9 Hz), 129.1, 128.7, 128.0, 125.7 (q, *J* = 3.8 Hz), 124.4 (q, *J* = 272.2 Hz), 122.4 (q, *J* = 3.8 Hz), 24.6, 23.8, 21.1, 11.6.

^{19}F-NMR: (377 MHz, $CDCl_3$) δ –62.73.

HRMS (EI): calc'd for $[C_{17}H_{15}F_3]^{+\cdot}$: 276.1120; found: 276.1120.

Synthesis of 1-(4-((1*S*,2*R*)-2-(*p*-tolyl)cyclopropyl)phenyl)ethan-1-one (4t):

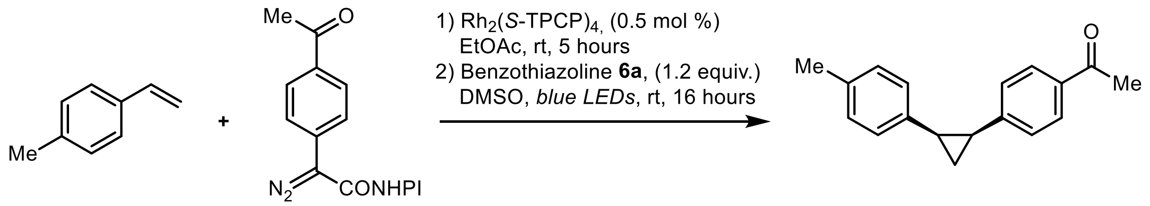

S2b **S1j** **4t**

General procedure B was applied employing **S2b** (46 μL, 0.35 mmol, 1.0 equiv.), **S1j** (122 mg, 0.35 mmol, 1.0 equiv.), and **6a** (90 mg, 0.42 mmol, 1.2 equiv.) in the second step. The crude mixture was purified by column chromatography (pentane:ethyl acetate, 18:1) to obtain **4t** as a colorless oil (41 mg, 47%; 98:2 dr). Compound (*S,R*)-**4t** was obtained in a 93.2:6.8 enantiomeric ratio determined by chiral HPLC using a Chiralpak-OZ-H column (Hexane:*i*-PrOH 90:10, 1 mL/min): τ_{major}= 6.5 min, τ_{minor}= 6.1 min.

Appearance: Colorless oil.

TLC: R_f: 0.36 (pentane:ethyl acetate 18:1, UV active, stains in vanillin).

$[\alpha]_D^{25}$: –44.8 (*c* = 1.0, $CHCl_3$).

^{1}H-NMR: (400 MHz, $CDCl_3$) δ 7.72 – 7.64 (m, 2H), 7.01 – 6.95 (m, 2H), 6.95 – 6.89 (m, 2H), 6.89 – 6.83 (m, 2H), 2.61 – 2.53 (m, 1H), 2.52 – 2.43 (m, 4H), 2.23 (s, 3H), 1.57 – 1.48 (m, 1H), 1.46 – 1.38 (m, 1H).

^{13}C-NMR: (101 MHz, $CDCl_3$) δ 198.0, 145.2, 135.6, 134.6, 134.4, 129.2, 128.7, 128.7, 127.9, 26.6, 25.3, 24.2, 21.1, 12.1.

HRMS (EI): calc'd for $[C_{18}H_{18}O]^{+\cdot}$: 250.1352; found: 250.1350.

Synthesis of phenylalanine derivative (4u):

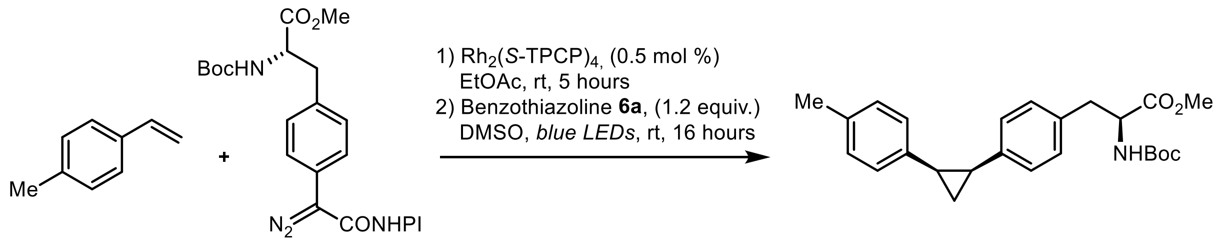

S2b **S1k** **4u**

General procedure B was applied employing **S2b** (46 µL, 0.35 mmol, 1.0 equiv.), **S1k** (178 mg, 0.35 mmol, 1.0 equiv.), and **6a** (90 mg, 0.42 mmol, 1.2 equiv.) in the second step. The crude mixture was purified by column chromatography (pentane:ethyl acetate, 8:2) to obtain **4u** as a colorless oil (36 mg, 25%; 93% (*S,S,R*)-diastereomer).

Appearance: Colorless oil.

TLC: R_f: 0.59 (pentane:ethyl acetate 8:2, UV active, stains in vanillin).

$[\alpha]_D^{25}$: +34.2 (c = 1.0, $CHCl_3$).

^{1}H-NMR: (400 MHz, $CDCl_3$) δ 6.97 – 6.75 (m, 8H), 4.89 (bd, J = 8.3 Hz, 1H), 4.49 (bq, J = 6.7 Hz, 1H), 3.59 (s, 3H), 2.96 (d, J = 6.0 Hz, 2H), 2.46 – 2.36 (m, 2H), 2.22 (s, 3H), 1.49 – 1.36 (m, 10H), 1.32 – 1.26 (m, 1H).

^{13}C-NMR: (101 MHz, $CDCl_3$) δ 172.5, 155.2, 137.6, 135.3, 135.1, 133.1, 129.2, 129.0, 128.6, 128.5, 79.9, 54.5, 52.1, 38.1, 28.4, 24.1, 23.9, 21.1, 11.6.

HRMS (ESI): calc'd for $[C_{25}H_{31}NO_4+Na]^+$: 432.2145; found: 432.2144.

Synthesis of 2-methoxy-5-((1*R*,2*S*)-2-(3,4,5-trimethoxyphenyl)cyclopropyl)phenol (4v):

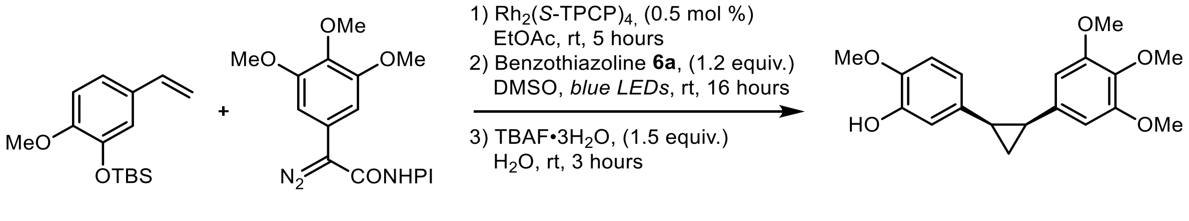

2v **1a** **4v**

General procedure B was applied employing **2v**[9] (93 mg, 0.35 mmol, 1.0 equiv.), **1a** (139 mg, 0.35 mmol, 1.0 equiv.), and **6a** (90 mg, 0.42 mmol, 1.2 equiv.) in the second step. To the reaction mixture was then added tetrabutylammonium fluoride trihydrate (166 mg, 0.525 mmol, 1.5 equiv.) and water (350 µL), then the mixture was stirred at room temperature for 3 hours. The mixture was poured in a separation funnel, diluted with water (15 mL) and ethyl acetate (15 mL). The organic phase was washed with water (3x15 mL) and the water phase was back-extracted with ethyl acetate (15 mL). The crude was purified by column chromatography (pentane:ethyl acetate, 7:3) to obtain **4v** as a colorless oil (58 mg, 50%; 98:2 dr). Compound (*R,S*)-**4v** was obtained in a 96.4:3.6 enantiomeric ratio determined by chiral HPLC using a Chiralpak-IB column (Hexane:*i*-PrOH 70:30, 1 mL/min): τ_{major}= 10.2 min, τ_{minor}= 11.3 min. Data are in agreement with literature data[10].

Appearance: Colorless oil.

TLC: R_f: 0.27 (pentane:ethyl acetate 6:4, UV active, stains in vanillin).

$[\alpha]_D^{25}$: –7.4 (c = 1.0, $CHCl_3$).

^{1}H-NMR: (400 MHz, $CDCl_3$) δ 6.66 (dd, *J* = 2.1, 0.6 Hz, 1H), 6.61 (d, *J* = 8.3 Hz, 1H), 6.47 (ddd, *J* = 8.3, 2.2, 0.7 Hz, 1H), 6.15 – 6.09 (m, 2H), 5.49 (s, 1H), 3.78 (s, 3H), 3.75 (s, 3H), 3.65 (s, 6H), 2.45 – 2.36 (m, 1H), 2.36 – 2.26 (m, 1H), 1.46 – 1.37 (m, 1H), 1.24 – 1.17 (m, 1H).

^{13}C-NMR: (101 MHz, $CDCl_3$) δ 152.5, 145.1, 144.8, 135.9, 134.7, 131.6, 120.7, 115.8, 110.3, 105.9, 60.9, 56.0, 56.0, 24.2, 24.1, 12.1.

Full characterization can be found at: N. Ty, R. Pontikis, G. G. Chabot, E. Devillers, L. Quentin, S. Bourg, J.-C. Florent, *Bioorg. Med. Chem.* **2013**, *21*, 1357-1366.

Photochemical studies

UV-Vis study

UV-Vis absorption spectra in DMSO of 2-phenylbenzothiazoline **6a**, redox-active ester **3a** and an equimolar mixture of both were measured to provide insights on the formation of an EDA complex. From these experiments, it is evident that the absorbance increases considerably in the 425-550 nm range (see **Figure S2**). These results suggest the formation of an EDA complex between **3a** and **6a**.

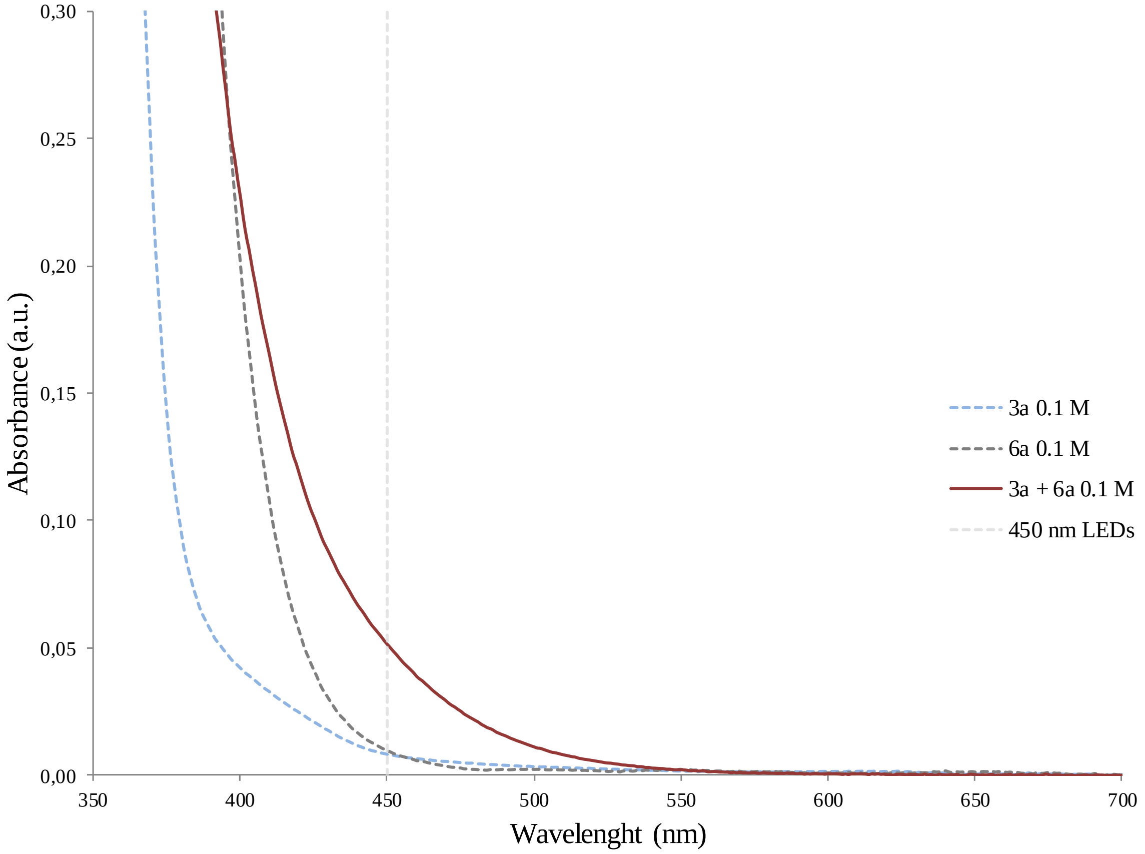

Figure S2: UV-Vis spectra of **3a**, **6a**, and their 1:1 mixture in DMSO at 0.1 M concentration.

Job plot

A Job plot was generated by measuring UV-Vis absorption spectra in DMSO of benzothiazoline **6a** and redox-active ester **3a** at different ratios, maintaining total concentration [**6a**] + [**3a**] = 0.1 M. The increase in absorbance was plotted against the molar fraction of redox-active ester **3a**. The resulting Job plot (**Figure S3**) indicated that the stoichiometry of the EDA-complex is 1:1.

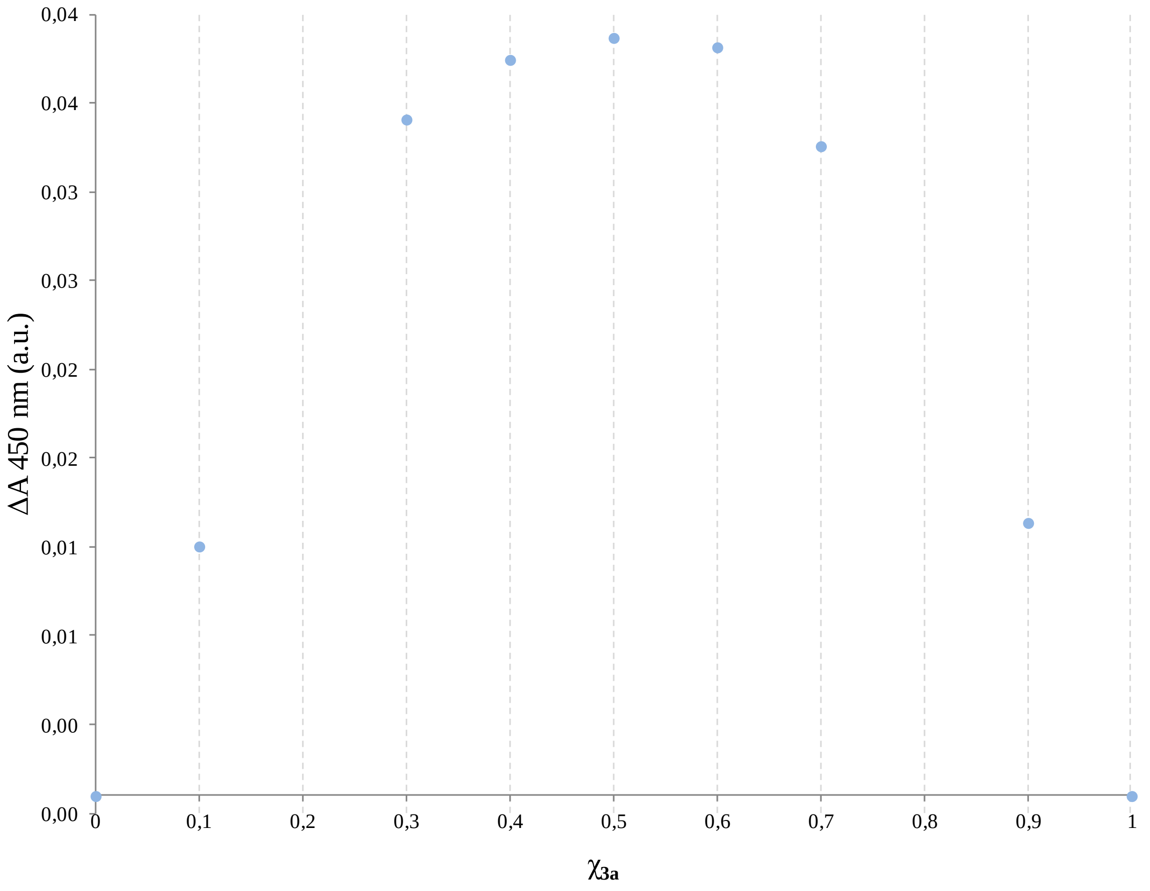

Figure S3: Job plot for benzothiazoline **6a** and redox-active ester **3a** in DMSO. [**6a**] + [**3a**] = 0.1 M.

Excitation and emission profiles

The excitation and emission spectra of 2-phenylbenzothiazoline **6a** were recorded at 410 nm and 355 nm respectively (**Figure S4**, yellow and gray dotted traces), at 0.02 M concentration in DMSO. The same was done for an equimolar mixture of **6a** and redox-active ester **3a**, measuring emission at 490 nm and exciting at 450 nm (**Figure S4**, red and blue traces) at 0.1 M concentration in DMSO. It is possible to observe a new spectroscopical feature emerged between 410 nm and 470 nm, which can be attributed to the formation of an EDA-complex.

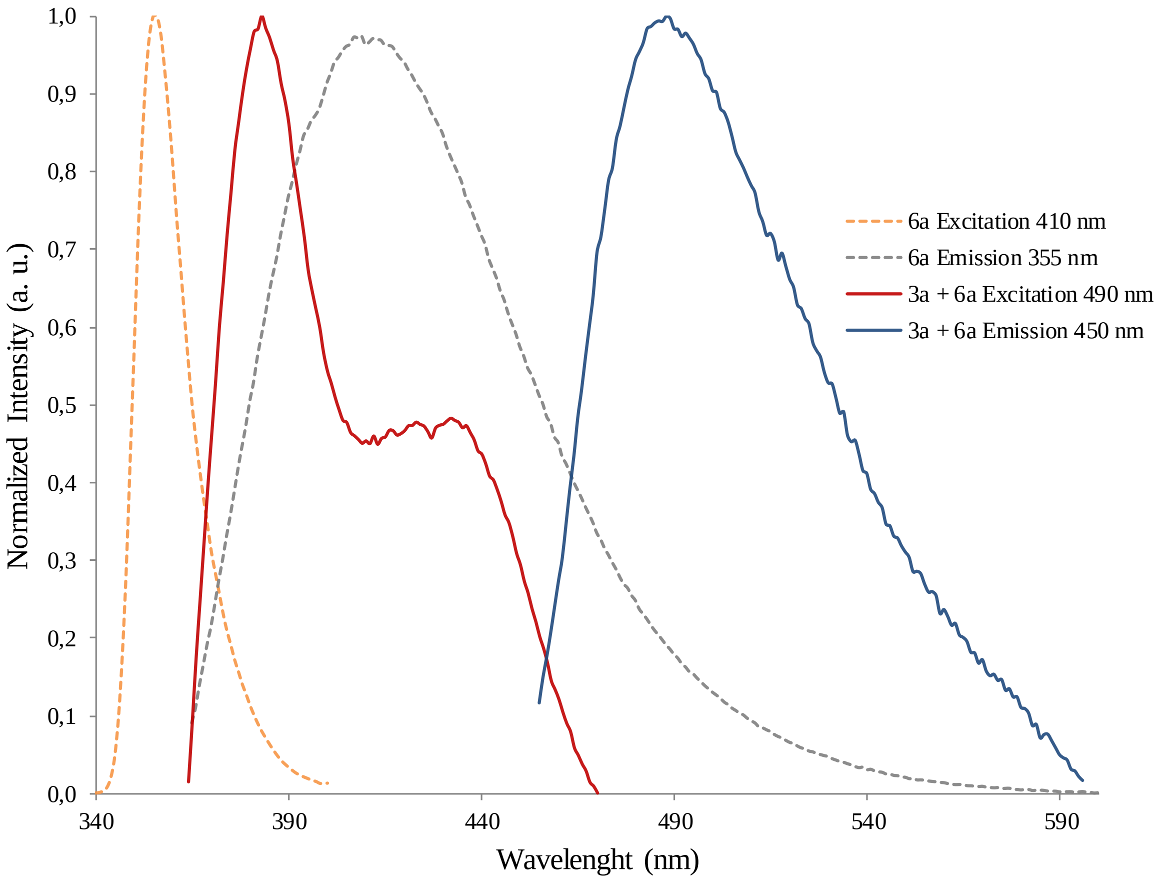

Figure S4: Excitation (yellow dotted) and emission spectra (gray dotted) of **6a**, overlapped with the excitation (red) and emission (blue) spectra of a 1:1 mixture of **3a** and **6a**. All spectra recorded in DMSO at 0.1 M concentration for **3a** + **6a**, 0.02 M for **6a**.

Stern-Volmer quenching experiment

A steady-state Stern-Volmer quenching experiment was performed by measuring the fluorescence intensity (excitation at 375 nm, emission measured at 440 nm) of a DMSO solution of benzothiazoline **6a** and redox-active ester **3a**, according to the indicated amounts in **Table S2**. The intensity was corrected by a concentration factor as follows:

$$I_{C_1} = I_{C_2}\frac{C_1}{C_2}$$

Where I_{C1} is the fluorescence intensity at concentration C_1 and I_{C2} is the fluorescence intensity at concentration C_2.

***Table S2**: Data of the fluorescence-based Stern-Volmer quenching experiment of 2-phenylbenzothiazoline **6a** and redox-active ester **3a**. All experiments were performed in dry, degassed spectroscopy-grade DMSO.*

Entry	*[**6a**] (M)*	*[**3a**] (M)*	*[**3a**]/ [**6a**]*	*Corrected Intensity (Counts 10^4)*	*I_0/I*
1	0.050	0.050	1.0	5.79	1.000
2	0.046	0.054	1.2	7.02	0.825
3	0.043	0.057	1.3	7.85	0.737
4	0.040	0.060	1.5	8.69	0.666
5	0.038	0.063	1.7	9.39	0.617
6	0.033	0.067	2.0	11.01	0.526
7	0.029	0.071	2.5	13.98	0.414
8	0.025	0.075	3.0	16.80	0.345

The data is shown in **Figure S5-6**. An increased emission intensity was observed with increasing concentrations of redox-active ester **3a** (**Figure S5**). This trend resulted in a negative slope in the steady-state Stern-Volmer plot (**Figure S6**).

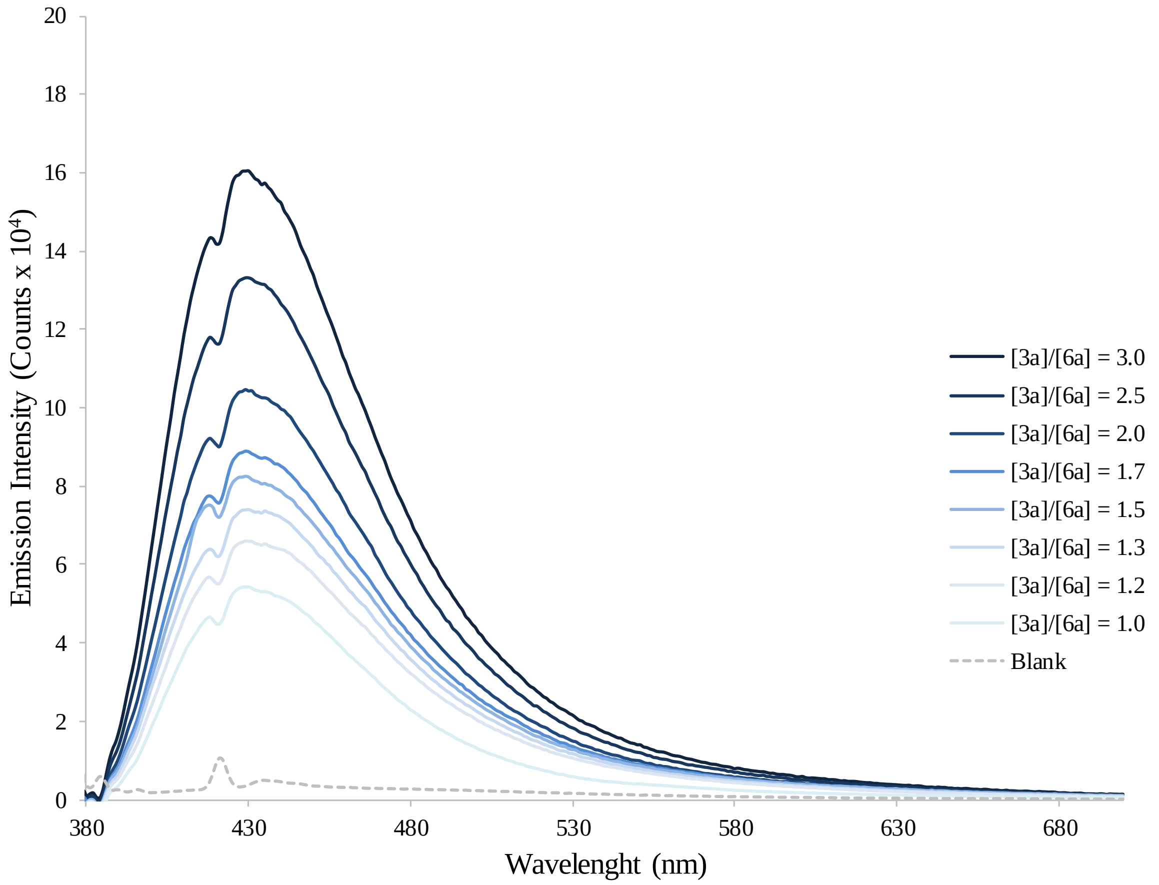

Figure S5: Emission profile of **3a** and **6a** in DMSO at increasing amounts of **3a**. The feature at λ = 422 nm is an artifact originated by subtraction of the blank (dotted gray) from the emission spectra.

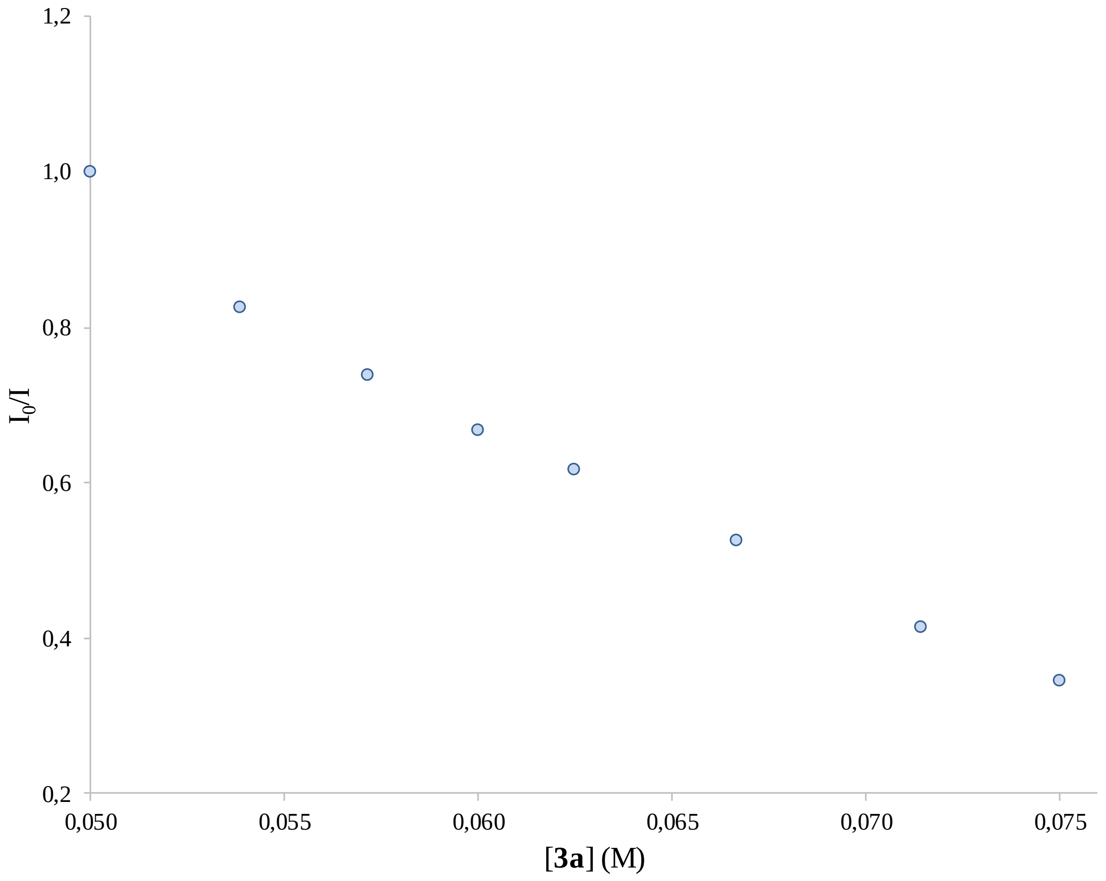

Figure S6: Steady-state Stern-Volmer quenching of **6a** and **3a** in DMSO.

Lifetime measurements were performed by TCSPC exciting at λ = 450 nm (emission bandwidth = 5.00 nm) employing a 50 ns pulsed laser with stop condition 10000 counts per channel. The time domaindecay was fitted using the exponential decay (τ, table S3). LUDOX® SM colloidal silica (30 wt.% suspension in H_2O) was used to measure the instrument response factor (IRF). Inside a glovebox, a dry cuvette was charged with 2-phenylbenzothiazoline **6a** (0.05 mmol) and a variable amount of redox-active ester **3a** (from 0.05 mmol to 0.25 mmol). The volume was then adjusted to 1 mL with dry degassed DMSO (spectroscopy grade). The results are summarized in **Table S3** below. The resulting graph shows how lifetime of the excited species (*ca* 1.43 ns) remains constant independently of the amount of redox-active ester, supporting the hypothesis of an intramolecular photoinduced electron-transfer mechanism (PET, see **Figure S7**).

Table S3: *Data of the lifetime-based Stern-Volmer quenching experiment of 2-phenylbenzothiazoline* ***6a*** *and redox-active ester* ***3a****. All experiments were performed in dry degassed spectroscopy-grade DMSO (V_{tot} = 1 mL).*

Entry	*[**6a**] (M)*	*[**3a**] (M)*	*[**3a**]/ [**6a**]*	*τ (ns)*	*Error τ (ns)*	*$τ_0/τ$*	*Error $τ_0/τ$*
1	0.05	0.00	0.0	1.739894	0.046902	1.00	0.05
2	0.05	0.05	1.0	1.410223	0.056378	1.23	0.06

3	0.05	0.06	1.2	1.472394	0.048958	1.18	0.05
4	0.05	0.08	1.4	1.432890	0.049349	1.21	0.05
5	0.05	0.10	1.6	1.390524	0.055080	1.25	0.06
6	0.05	0.15	1.8	1.407267	0.060774	1.24	0.06
7	0.05	0.25	2.0	1.474024	0.063669	1.18	0.06

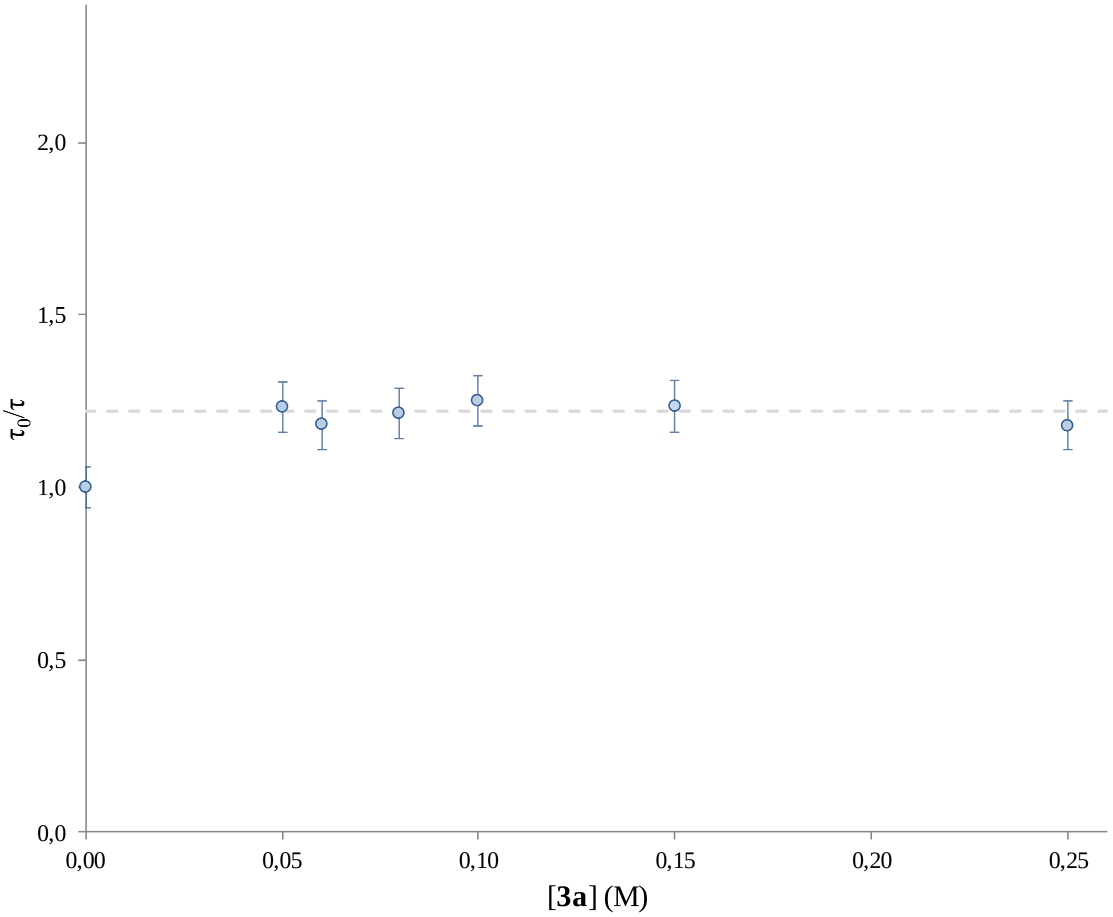

Figure S7: Stern-Volmer quenching experiment of an equimolar amount of **6a** and **3a** in DMSO. Error bars set at 6%.

Quantum yield measurements

The photon flux of the spectrophotometer was determined by standard potassium ferrioxalate actinometry.[11]

Preparation of buffered phenanthroline solution (Solution A)

A buffered solution was made by dissolving phenanthroline (25 mg, 138.0 µmol) and anhydrous sodium acetate (5.63 g, 0.07 mol) in 0.5 M H_2SO_4 (25 mL).

Preparation of potassium ferrioxalate solution (Solution B)

The solution of potassium ferrioxalate trihydrate in 0.05 M H_2SO_4 was prepared in a dark laboratory illuminated with red light. Potassium ferrioxalate trihydrate (736 mg, 1.5 mmol) was dissolved in 0.05 M H_2SO_4 (10 mL) in a volumetric flask to yield a 0.15 M solution.

Four cuvettes were charged with solution B (2 mL). The cuvettes were irradiated at λ = 450 nm with an excitation slit width of 5.0 nm for the time as indicated in **table S4**. After irradiation, phenanthroline solution (0.35 mL solution A) was added to every cuvette (irradiated and non-irradiated). The solutions were then allowed to rest for 1 h in the dark to complete the chelation of ferrous ions to the phenanthroline. After 1 h, absorbance was measured for all four solutions (and a 0.05 M H_2SO_4 blank) at λ = 510 nm. For the calculation of photon flux the following equations were used:

$$\text{mol of } Fe^{2+} = \frac{V * \Delta\text{A}}{\text{l} * \text{Ɛ}}$$

$$Photon\ flux = \frac{mol\ of\ Fe^{2+}}{\Phi * \text{t} * \text{f}}$$

Where V is the total volume of the solution (2.35 mL), ΔA is the difference in absorption between the irradiated and non-irradiated samples, l is the path length (1 cm), and Ɛ is the molar absorptivity at λ = 510 nm (11110 L mol^{-1} cm^{-1}),[11] φ is the quantum yield for the potassium ferrioxalate actinometer (1.01),[11] t is the time of irradiation (s), and f is the fraction of light absorbed at λ = 450 nm by potassium ferrioxalate (0.99833).[11]

***Table S4**: Determination of photon flux by ferrioxalate actinometry.*

Entry	*Irradiation time (s)*	*Abs (A)*	*ΔA*	*V (Lx10^{-3})*	*Fe^{2+} (mol·10^{-8})*	*Photon flux (einstein·s^{-1}·10^{-9})*
1	0.00	0.575	0.000	2.35	-	-
2	10.70	0.769	0.194	2.35	4.11	3.81
3	20.80	0.917	0.342	2.35	7.24	3.45
4	30.90	1.130	0.555	2.35	11.75	3.77
						Average: 3.68
						Std. dev.: 0.22

Considering 95% confidence interval, the average measured photon flux is 3.68 ± 0.22 einstein·s^{-1}·10^{-9}.

Determination of the quantum yield

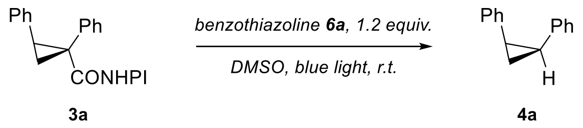

Inside a glovebox, a cuvette was charged with redox-active ester **3a** (68.9 mg, 0.1 mmol, 1.0 equiv.) and 2-phenylbenzothiazoline **6a** (64.9 mg, 0.12 mmol, 1.2 equiv.). Anhydrous DMSO (1 mL) was then added to the mixture. The cuvette was wrapped in an aluminium foil and was brought outside the glovebox. The absorption was measured at λ = 450 nm. The sample was irradiated in the spectrofluorometer for the indicated period of time (**Table S5** at λ = 450 nm with an excitation slit width of 5.0 nm. The yield of the reaction was measured by no-*D* ^{1}H-NMR using 1,2,4,5-tetrachloro-3-nitrobenzene as an internal standard.

$$Quantum\ yield, \Phi = \frac{mol\ of\ \mathbf{4a}}{\text{photon flux} * \text{t} * \text{f}}$$

Where t is the time of the reaction and f is the fraction of the light absorbed at 450 nm by the reaction mixture = $1–10^{–A}$. The initial absorbance (A) of the reaction mixture at λ = 450 nm is 0.25, and the absorbance of the same sample after 20 hours is 0.19. The average value of f is, $(f_0 + f_f)/2$ = 0.3920. The results are summarized in **Table S5**.

Table S5: *Determination of the quantum yield of the photodecarboxylative reduction reaction.*

Entry	*Irradiation time (s)*	*NMR yield **4a** (%)*	***4a** (mol·10^{-5})*	*Quantum yield (φ)*	*Error (Δφ)*
1	57600	6	5.95	0.072	0.06
2	72000	8	7.65	0.074	0.05
3	86400	14	13.51	0.108	0.04

Considering 5% error in measuring the NMR yield, the error in determining the mol of **4a** is 1.20·10^{-5}.

The following equations were used to calculate the error in determining the quantum yield.

$$\Phi = \frac{mol\ of\ 4a}{photon\ flux * t * f}$$

Therefore, the error in measuring the quantum yield (φ) is:

$$\Delta\Phi = \sqrt{[\frac{\partial\Phi}{\partial(mol\ of\ 4a)} * \Delta(mol\ of\ 4a)]^2 + [\frac{\partial\Phi}{\partial(photon\ flux)} * \Delta(photon\ flux)]^2}$$

Or, $\Delta\Phi = \sqrt{\left[\frac{\partial}{\partial(mol\ of\ 4a)}\left(\frac{mol\ of\ 4a}{photon\ flux*t*f}\right)*\Delta(mol\ of\ 4a)\right]^2 + \left[\frac{\partial}{\partial(photon\ flux)}\left(\frac{mol\ of\ 4a}{photon\ flux*t*f}\right)*\Delta(photon\ flux)\right]^2}$

Or, $\Delta\Phi = \sqrt{\left[\frac{1}{photon\ flux*t*f}*\Delta(mol\ of\ 4a)\right]^2 + \left[(-)\frac{mol\ of\ 4a}{(photon\ flux)^2*t*f}*\Delta(photon\ flux)\right]^2}$

Or, $\Delta\Phi = \sqrt{\left[\frac{1}{photon\ flux*t*f}*\Delta(mol\ of\ 4a)\right]^2 + \left[\frac{mol\ of\ 4a}{(photon\ flux)^2*t*f}*\Delta(photon\ flux)\right]^2}$

Thus, $\Delta\Phi_1 = 0.060, \Delta\Phi_2 = 0.048, \Delta\Phi_3 = 0.041.$

Average quantum yield of the photo-ligation reaction was calculated to be

$$\bar{\Phi} = \frac{\Phi_1 + \Phi_2 + \Phi_3}{3} = 0.085.$$

The error in measuring the average quantum yield of the photo-ligation reaction is

$$\Delta\bar{\Phi} = \sqrt{\left(\frac{\delta\bar{\Phi}}{\delta\Phi_1}*\Delta\Phi_1\right)^2 + \left(\frac{\delta\bar{\Phi}}{\delta\Phi_2}*\Delta\Phi_2\right)^2 + \left(\frac{\delta\bar{\Phi}}{\delta\Phi_3}*\Delta\Phi_3\right)^2}$$

Or, $\Delta\bar{\Phi} = \sqrt{\left(\frac{1}{3}*\Delta\Phi_1\right)^2 + \left(\frac{1}{3}*\Delta\Phi_2\right)^2 + \left(\frac{1}{3}*\Delta\Phi_3\right)^2} = 0.029$

After taking into account all the errors, the average quantum yield of the reaction was measured to be $\bar{\Phi} = 0.09 \pm 0.03$.

1D-NOE study of the EDA-complex

^{1}H NMR of redox-active ester 3a in DMSO-d$_6$

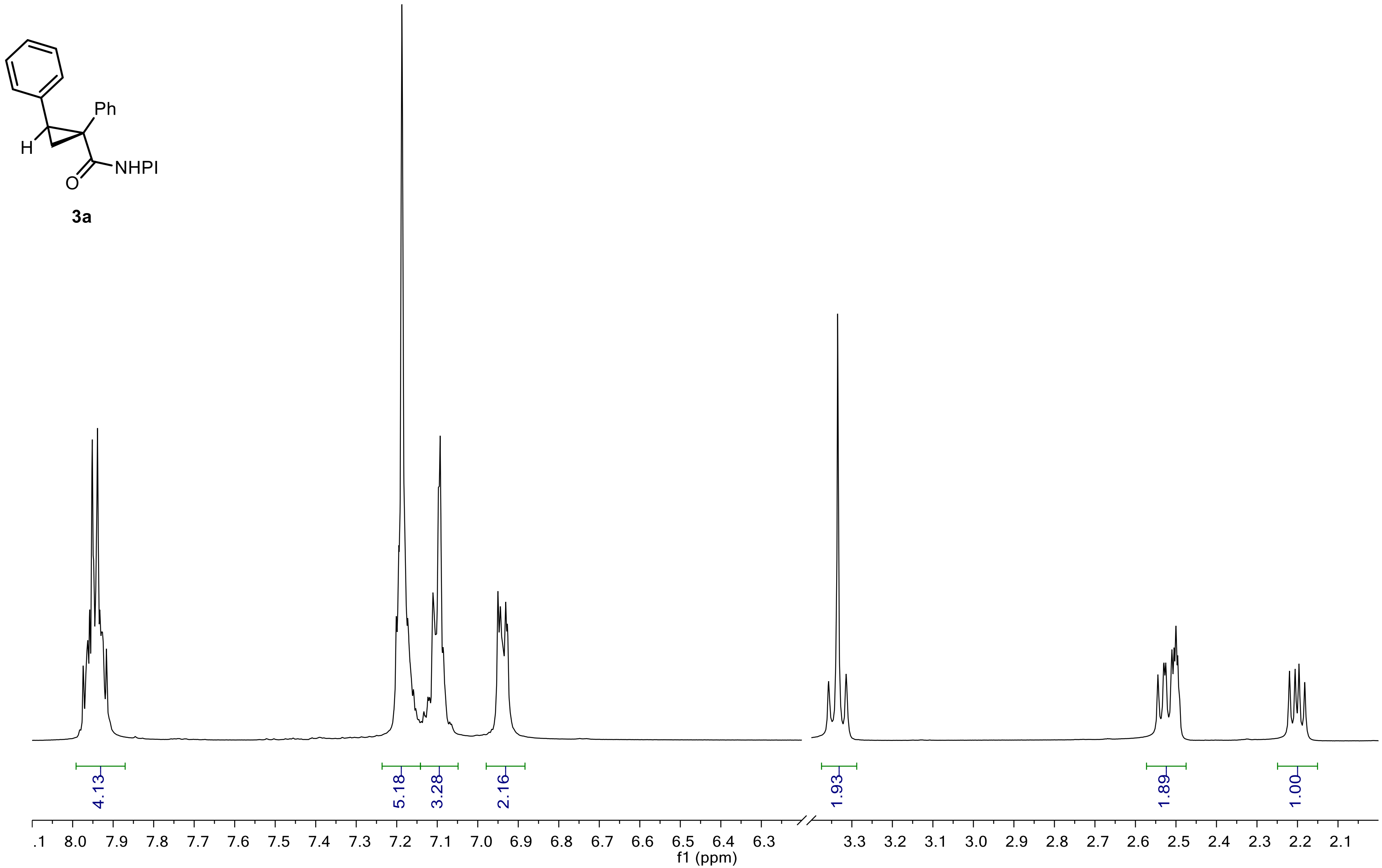

^{1}H NMR of benzothiazoline 6a in DMSO-d_6

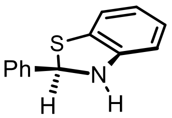

6a

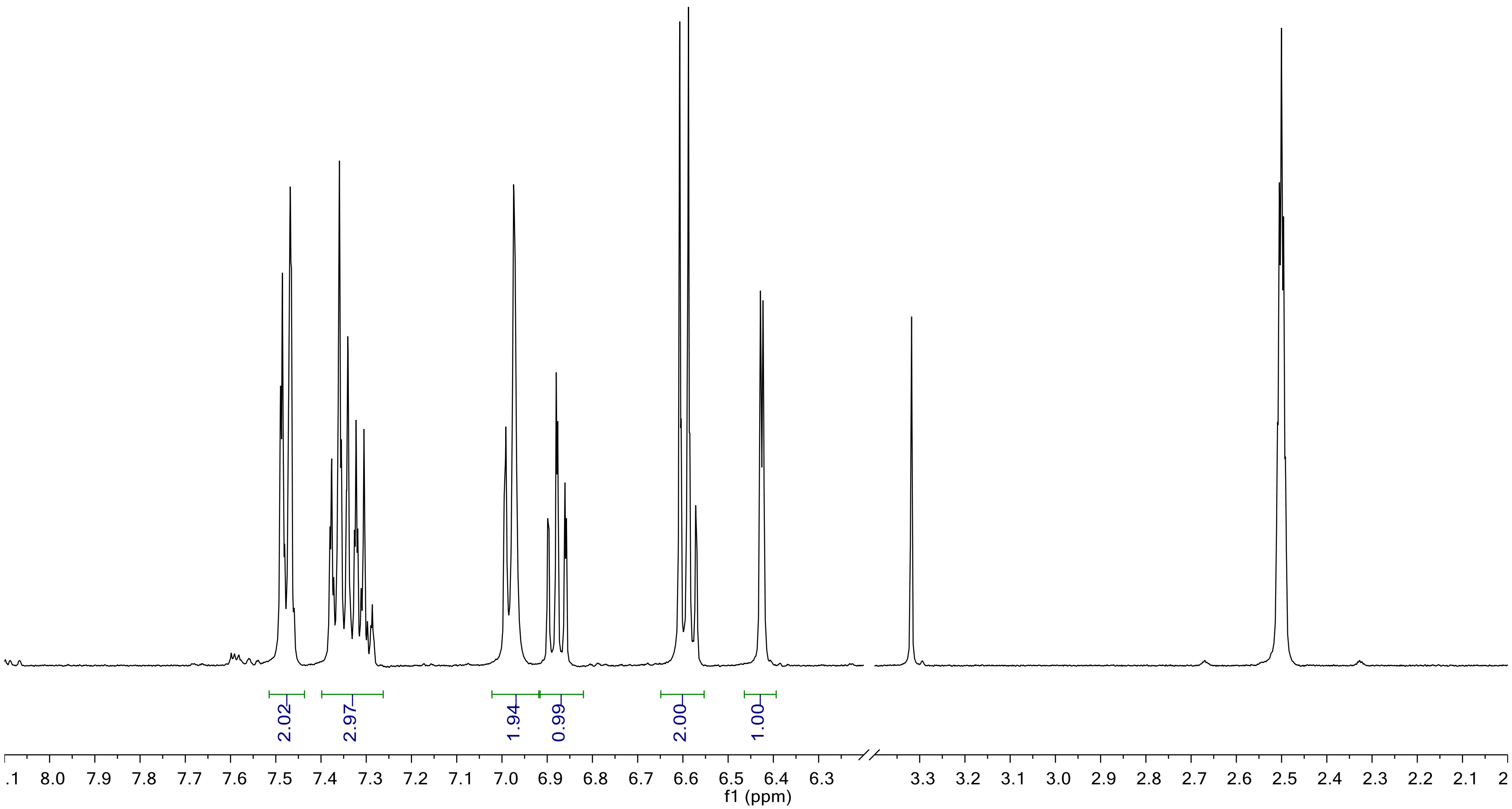

^{1}H NMR of a 1:1 mixture of 6a and redox-active ester 3a in DMSO-d_6

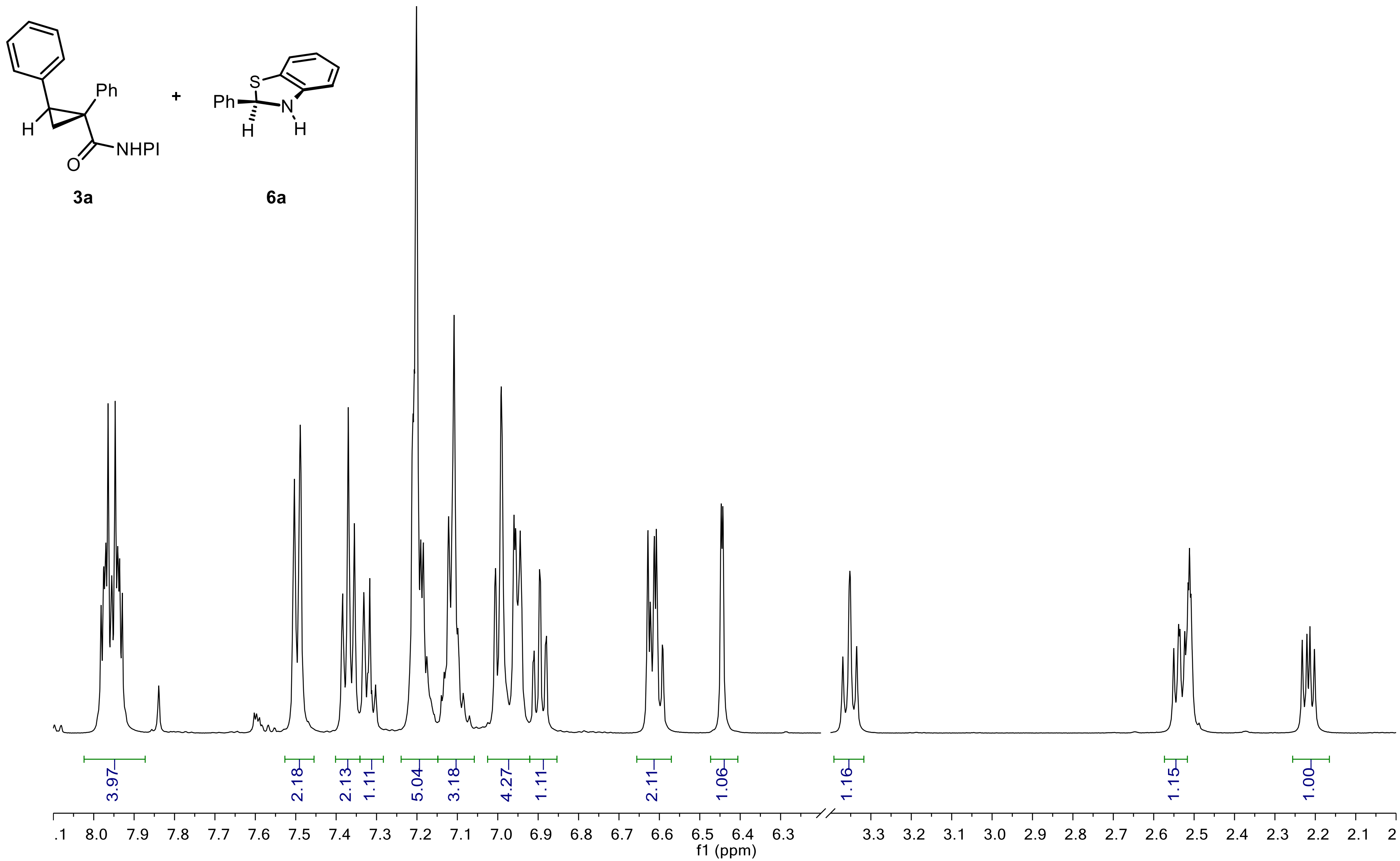

^{1}H NMR overlay of redox-active ester 3a (red), 6a (blue) and their 1:1 mixture (black) in DMSO-d6

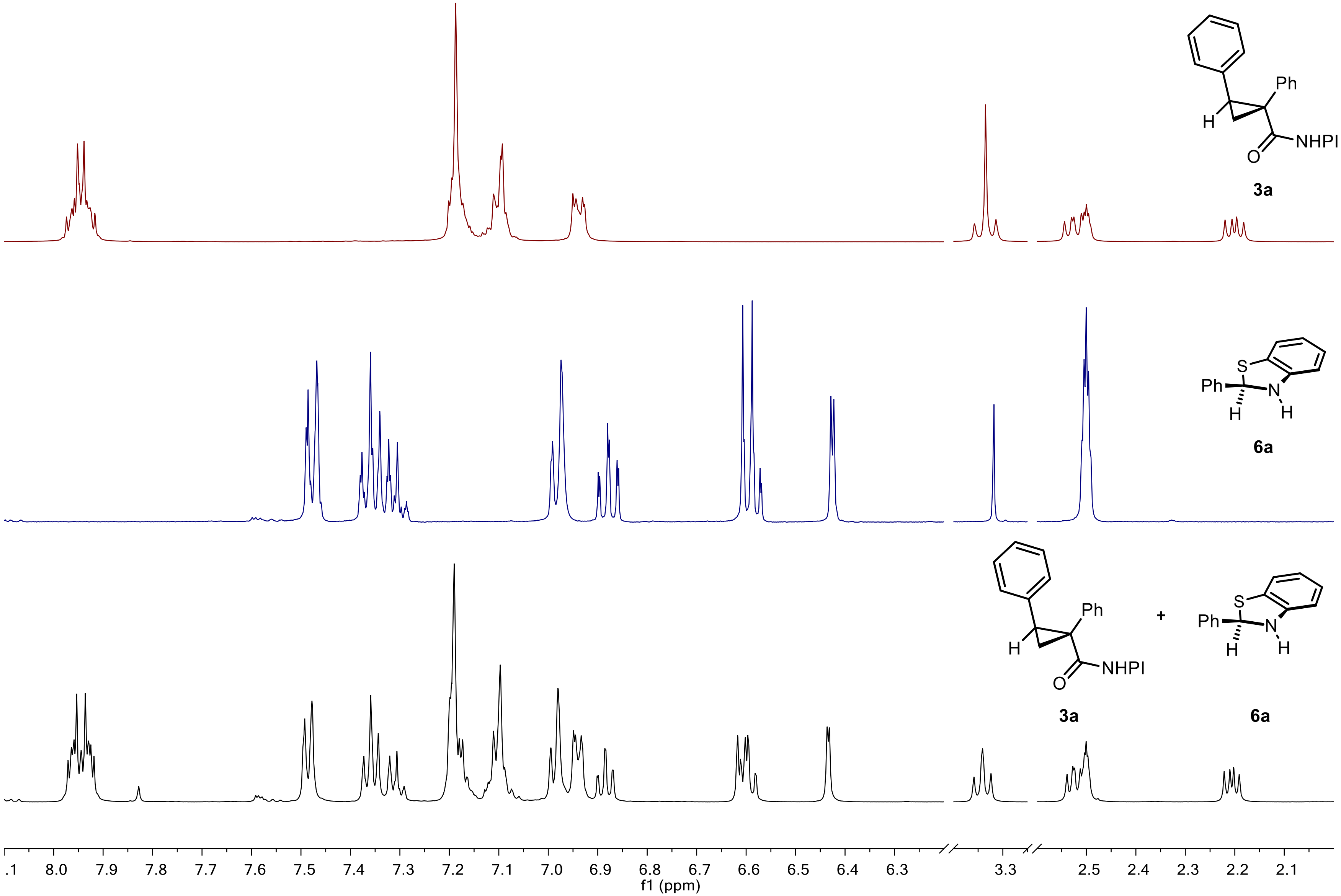

^{1}H NMR overlay of redox-active ester 3a (red), 6a (blue) and their 1:1 mixture (black) in DMSO-d_6 (superimposed)

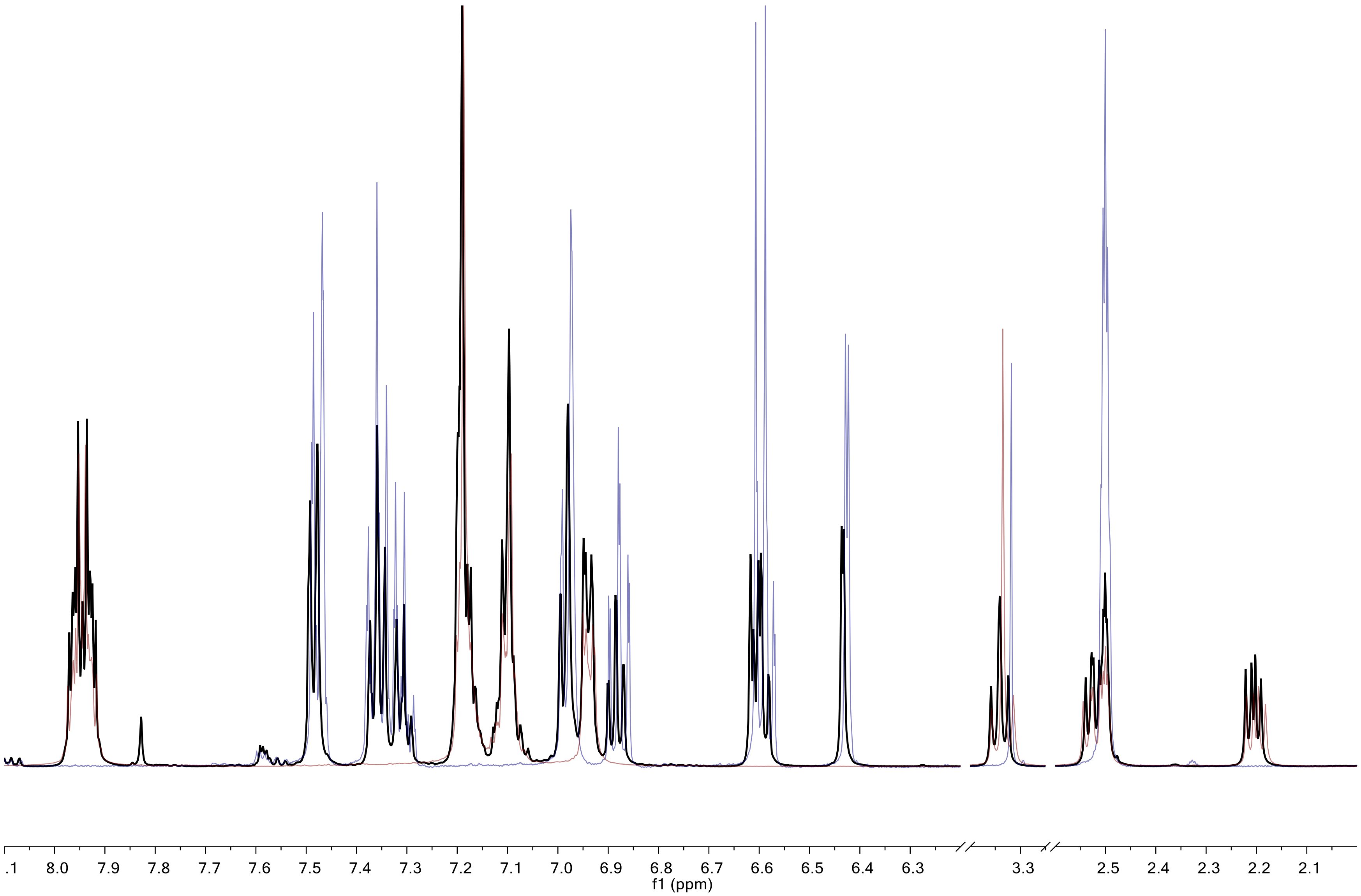

^{1}H 1D-NOESY of a 1:1 mixture of 6a and redox-active ester 3a in DMSO-d_6 (irradiation at 2.21 ppm, 750 ms mixing time)

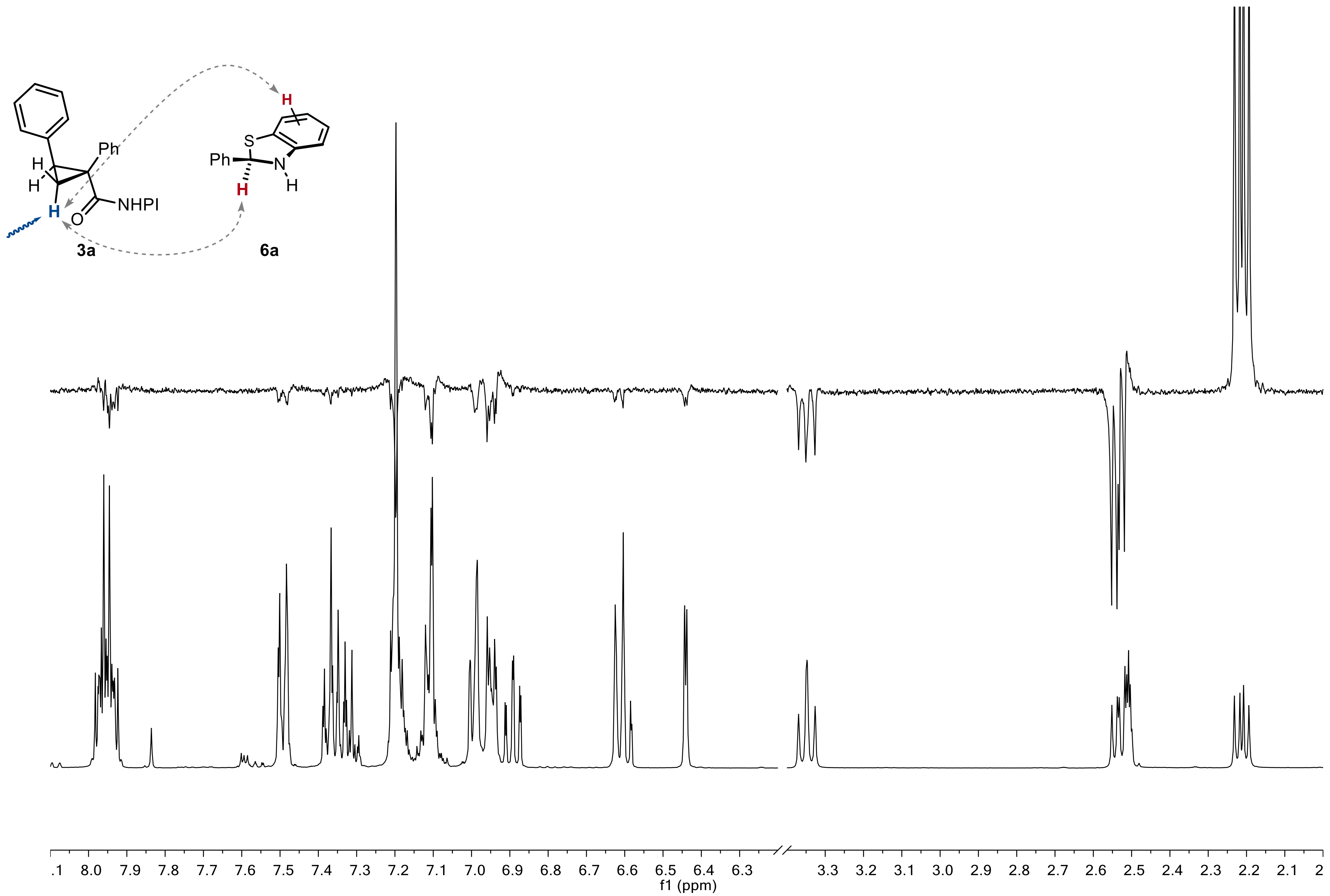

^{1}H 1D-NOESY of a 1:1 mixture of 6a and redox-active ester 3a in DMSO-d_6 (irradiation at 6.44 ppm, 750 ms mixing time)

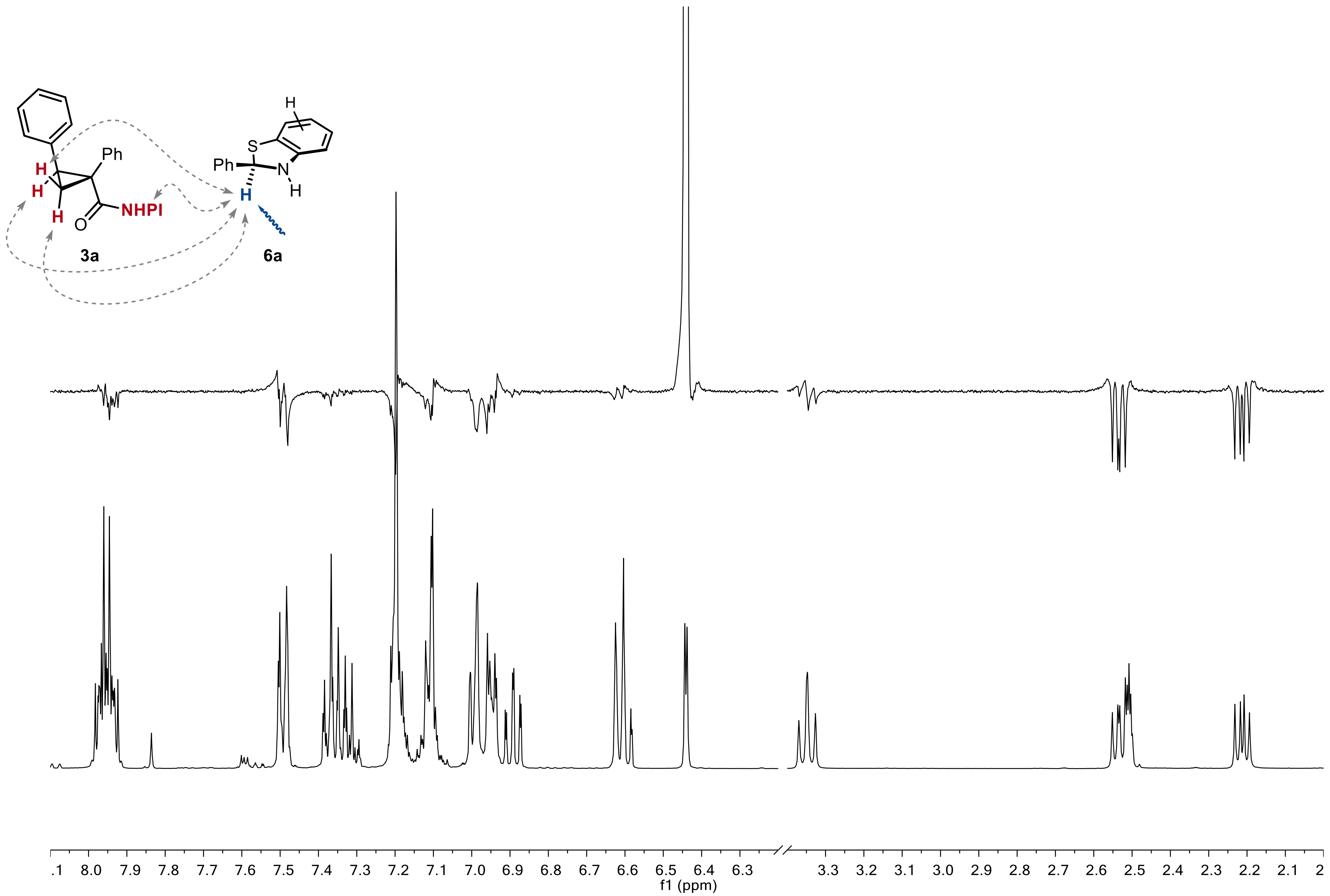

^{1}H 1D-NOESY of a 1:1 mixture of 6a and redox-active ester 3a in DMSO-d$_6$ (irradiation at 7.95 ppm, 750 ms mixing time)

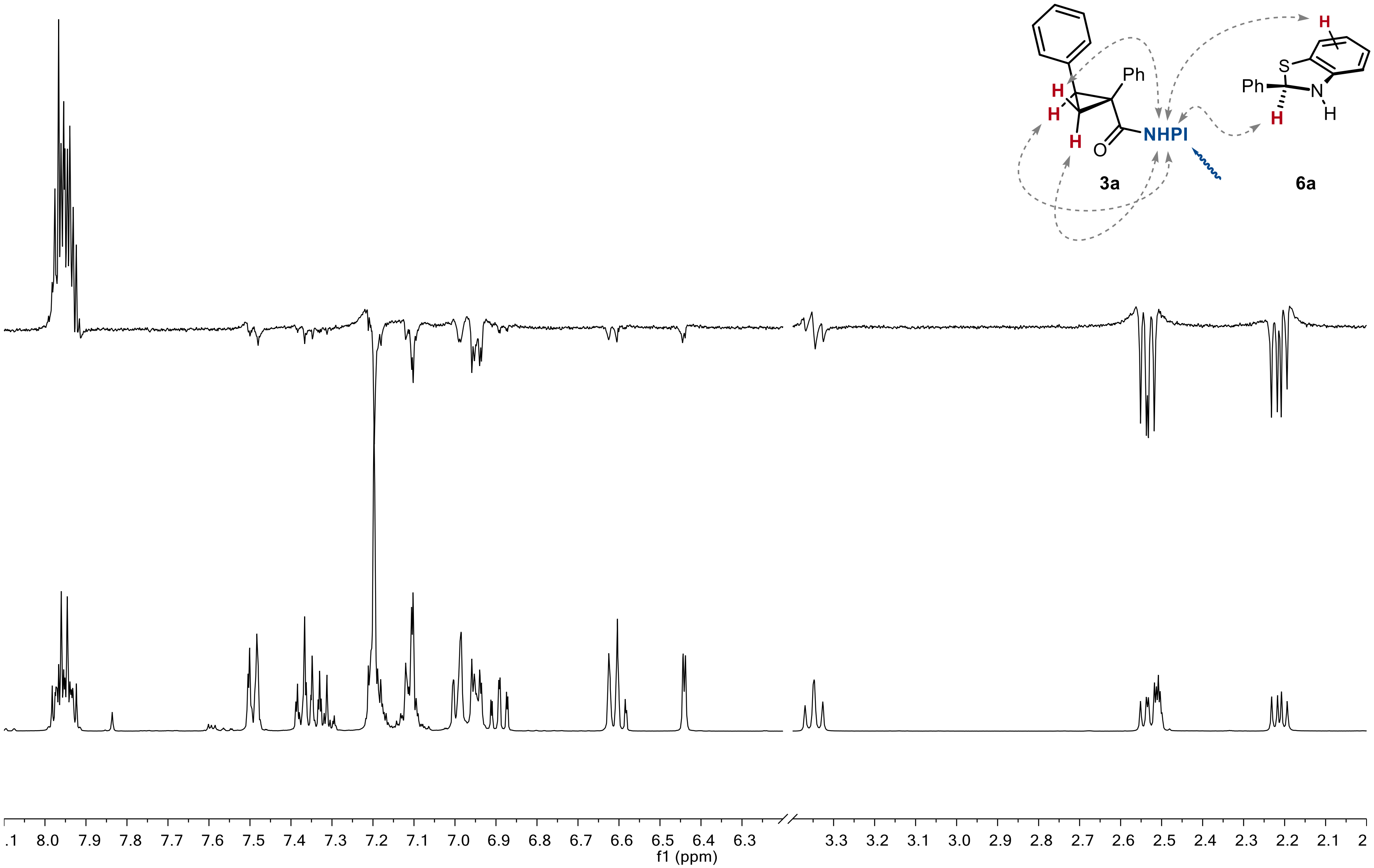

^{1}H 1D-NOESY of a 1:1 mixture of 6a and redox-active ester 3a in DMSO-d6 (irradiation at 3.35 ppm, 750 ms mixing time)

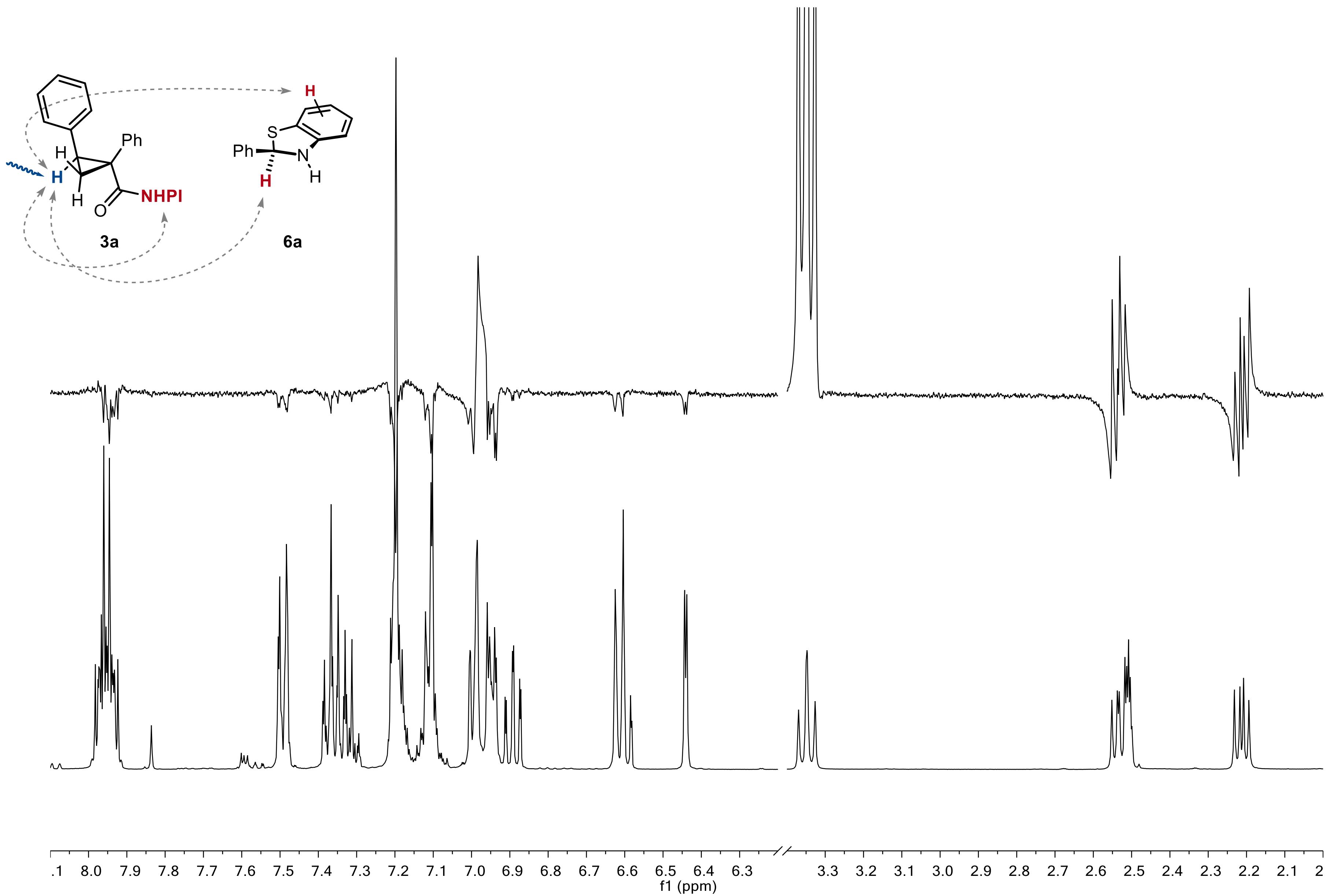

^{1}H 1D-NOESY of a 1:1 mixture of 6a and redox-active ester 3a in DMSO-d_6 (irradiation at 6.60 ppm, 500 ms mixing time)

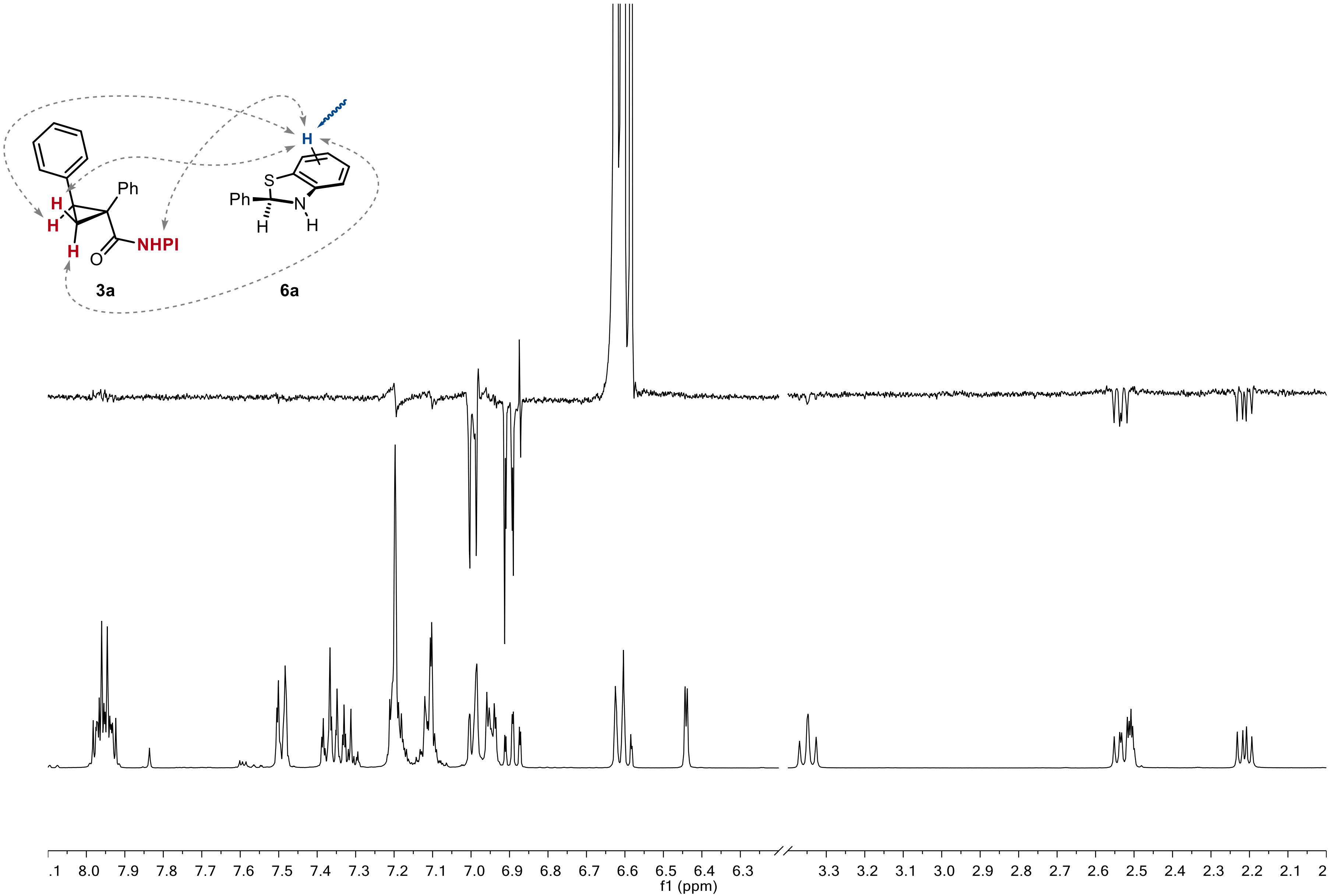

Mechanistic experiments

Synthesis of 2-phenyl-2,3-dihydrobenzo[5]thiazole-2-d (6a-d_1)

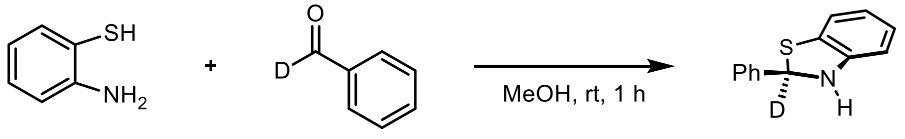

6a-d$_1$

A flask was loaded with 2-aminothiophenol (2.10 g, 0.017 mol, 1.0 equiv.) and benzaldehyde-d_1 (1.80 mL, 0.017 mol, 1.0 equiv.). The solids were dissolved in methanol (1.7 mL) and stirred for one hour at room temperature. The flask was then cooled at 0 °C for 30 minutes, until a precipitate started to form. The solids were filtered and dried under vacuum to afford the pure product **6a-d$_1$** as white solid (3.356 g, 93%; >95% D). Data are in agreement with literature data[12].

Appearance: White solid.

^{1}H-NMR: (400 MHz, $CDCl_3$) δ 7.60 – 7.51 (m, 2H), 7.41 – 7.29 (m, 3H), 7.05 (dd, J = 7.6, 1.3 Hz, 1H), 6.95 (td, J = 7.6, 1.3 Hz, 1H), 6.77 (td, J = 7.5, 1.2 Hz, 1H), 6.67 (dd, J = 7.7, 1.1 Hz, 1H), 4.35 (s, 1H).

N-deuteration of 2-phenylbenzothiazolines 6a-d_2 and 6a-d_1′

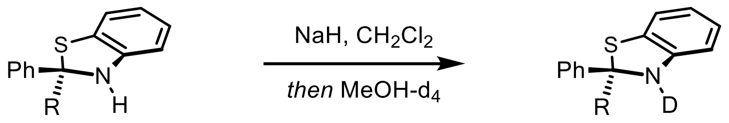

6a, R = H
6a-d$_1$, R = D

6a-d$_1$', R = H
6a-d$_2$, R = D

The *N*-deuteration of 2-phenylbenzothiazolines **6a** and **6a-d$_1$** was performed by dissolving the substrate (0.1 mmol) in CH_2Cl_2 (1 mL), followed by addition of NaH (0.25 mmol). The solution was stirred for 10 minutes before quenching with 1 mL of MeOH-d_4. The solvent was then removed, and the procedure was repeated three times in total. The deuterium incorporation was assessed by ^{1}H NMR analysis of the resulting solid: **6a-d$_1$′** (94% D), **6a-d$_2$** (93% D).

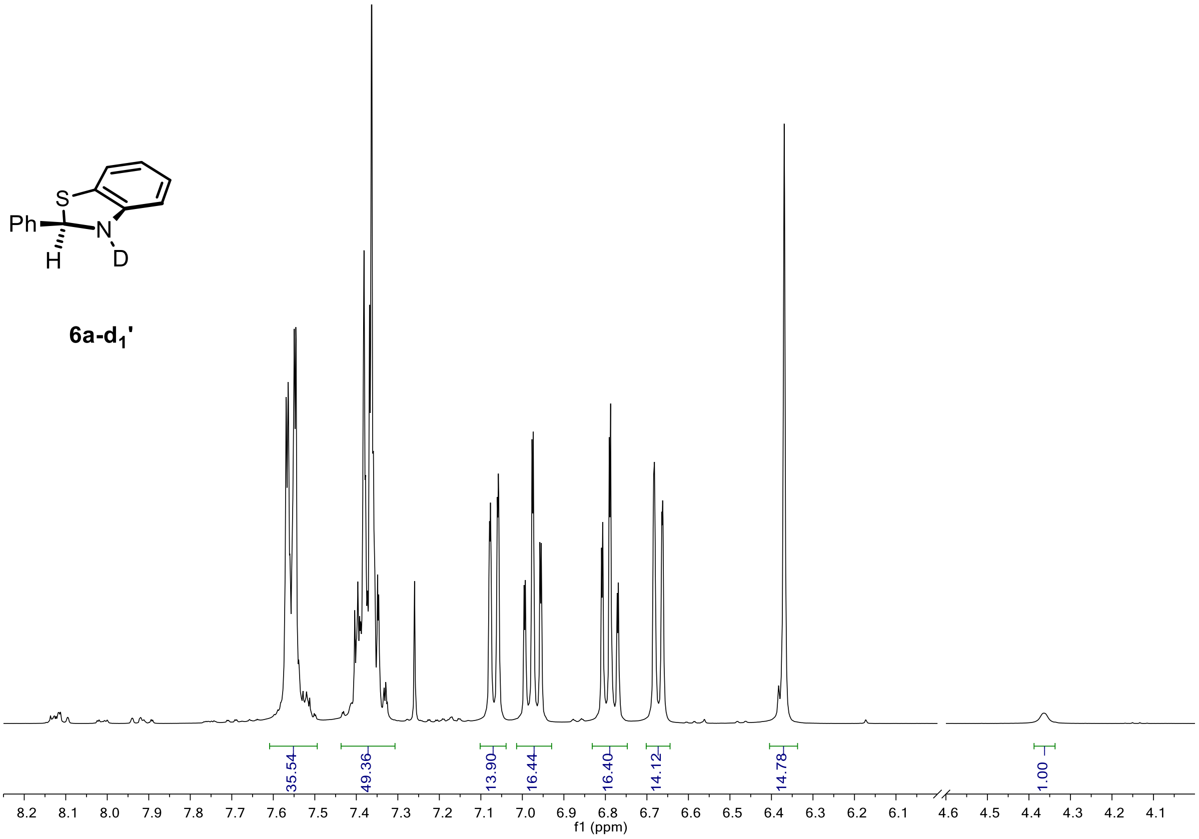

Figure S8: Cropped ^{1}H NMR of deuterated benzothiazoline **6a-d$_1$'**.

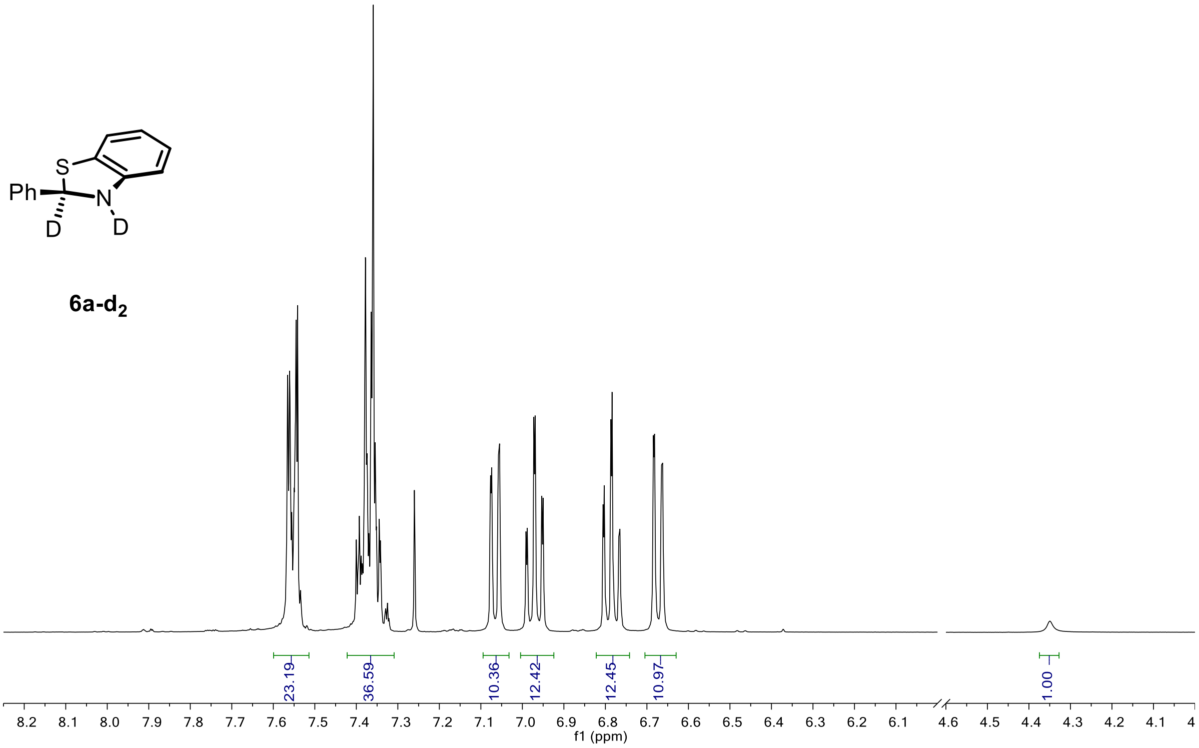

Figure S9: Cropped ^{1}H NMR of deuterated benzothiazoline **6a-d$_2$**.

Synthesis of 1,3-dioxoisoindolin-2-yl 1,2-diphenyl-2-(trimethylsilyl)cyclopropane-1-carboxylate (S3b)

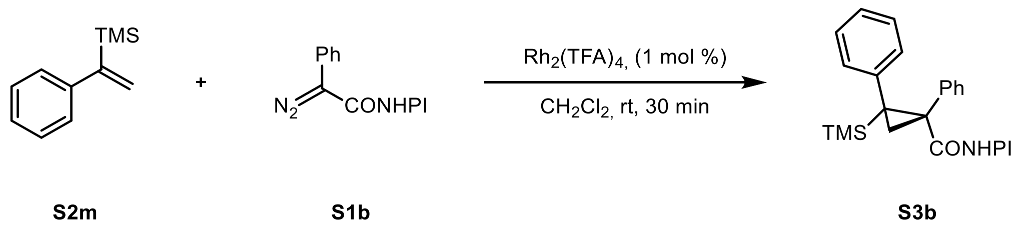

General procedure B was applied employing **S2m**[13] (132.3 mg, 0.750 mmol, 1.5 equiv.), **S1l** (153.6 mg, 0.500 mmol, 1 equiv.), and $Rh_2(TFA)_4$ (3.3 mg, 0.005 mmol, 1 mol %) using dichloromethane (20 mL) as a solvent instead of ethyl acetate. The crude mixture was purified by column chromatography (pentane:dichloromethane, 1:1 to 1:2) to obtain **S3b** as a white solid (105 mg, 46%, > 99:1 dr for the *trans*-isomer).

Appearance: White solid.

TLC: R_f: 0.66 (pentane:ethyl acetate 8:2, UV active, stains in vanillin).

^{1}H-NMR: (400 MHz, $CDCl_3$) δ 7.95 – 7.82 (m, 2H), 7.82 – 7.72 (m, 2H), 7.31 – 7.19 (m, 2H), 7.16 – 6.83 (m, 8H), 2.42 (d, J = 5.1 Hz, 1H), 2.10 (d, J = 5.0 Hz, 1H), 0.05 (s, 9H).

^{13}C-NMR: (101 MHz, $CDCl_3$) δ 170.1, 161.9, 140.2, 134.8, 133.9, 130.0, 129.1, 127.8, 127.3, 125.6, 124.0, 37.8, 34.9, 22.9, -0.6.

HRMS (ESI): calc'd for $[C_{27}H_{25}NO_4Si+Na]^+$: 478.1445; found: 478.1443.

Synthesis of 1,3-dioxoisoindolin-2-yl-1,2-diphenylcyclopropane-1-carboxylate (*diast*-3a)

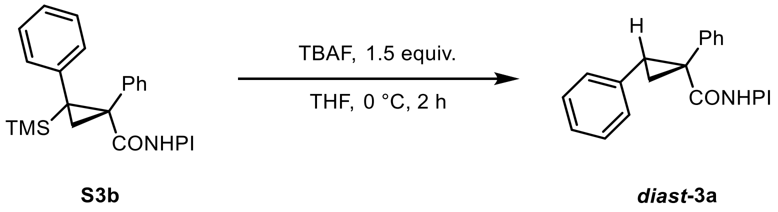

Adapted from a reported procedure,[13] to a solution of cyclopropane **S3b** (100.0 mg, 0.219 mmol, 1.0 equiv.) in dry THF (2.2 mL) at 0 °C, was added a 1 M solution of TBAF in THF (330 µL, 0.330 mmol, 1.5 equiv.). The mixture was stirred at 0 °C for 2 hours, then partitioned between dichloromethane and water. The organic phase was separated and washed successively with sat. aqueous NH_4Cl, water, and brine, then dried over Na_2SO_4. The mixture was concentrated and the residue was purified by column chromatography (pentane:ethyl acetate 6:1) to afford pure ***diast*-3a** as a white solid (42.0 mg, 50%).

Appearance: White solid.

TLC: R_f: 0.45 (pentane:ethyl acetate 8:2, UV active, stains in vanillin).

^{1}H-NMR: (400 MHz, $CDCl_3$) δ 7.79 – 7.72 (m, 2H), 7.72 – 7.63 (m, 4H), 7.50 – 7.41 (m, 4H), 7.41 – 7.33 (m, 3H), 7.33 – 7.21 (m, 1H), 3.25 (t, J = 8.5 Hz, 1H), 2.43 (dd, J = 7.9, 5.0 Hz, 1H), 1.88 (dd, J = 9.0, 5.0 Hz, 1H).

^{13}C-NMR: (101 MHz, $CDCl_3$) δ 167.6, 161.5, 138.1, 134.9, 134.6, 130.6, 129.6, 129.0, 128.8, 128.5, 128.2, 127.6, 123.8, 36.0, 35.5, 21.8.

HRMS (ESI): calc'd for $[C_{24}H_{17}NO_4+Na]^+$: 406.1050; found: 406.1047.

Stereo-inversion experiment

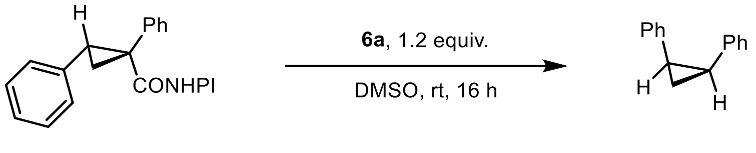

diast-3a **4a**

A vial was loaded with redox-active ester ***diast*-3a** (38.3 mg, 0.10 mmol, 1.0 equiv.) and benzothiazoline **6a** (25.6 mg, 0.12 mmol, 1.2 equiv.), followed by dry DMSO (1 mL). The mixture was stirred under blue LEDs irradiation with fan cooling. After 16 hours, the crude was diluted with ethyl acetate (4 mL) and extracted with water (3x4 mL), then the aqueous phase was back-extracted with ethyl acetate (4 mL). The solvent was removed and the crude analyzed by ^{1}H NMR to determine the yield using 1,1,2,2-tetrachloroethane as an internal standard, followed by filtration over a plug of silica (eluting with pure pentane) before determination of the diastereomeric ratios by GC-MS. Product ***cis*-4a** was obtained in 83% yield and 97.2:2.8 diastereomeric ratio.

Deuteration experiments

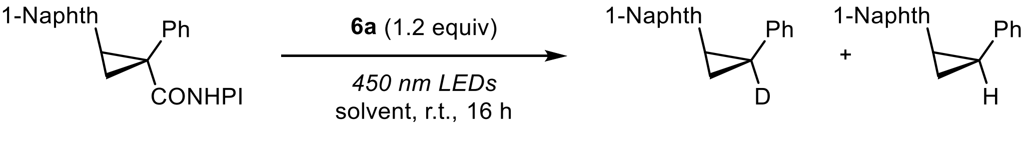

3i **4i-d$_1$** **4i**

A vial was loaded with redox-active ester **3i** (43.0 mg, 0.10 mmol, 1.0 equiv.) and the indicated benzothiazoline **6a** (26.0 mg, 0.12 mmol, 1.2 equiv.). Dry DMSO (1 mL) was added and the mixtures were stirred overnight under blue LEDs irradiation, with fan cooling. The crude was diluted with ethyl acetate (4 mL) and extracted with water (3x4 mL), then the aqueous phase was back-extracted with ethyl acetate (4 mL). The solvent was removed and the crude analyzed by ^{1}H NMR to determine the yield, followed by preparative TLC

(pentane:dichloromethane 15:1) before determination of the diastereomeric ratios. The results are summarized in **Table S6** below.

***Table S6**: Deuterium incorporation experiments. Reported yields are determined by ^{1}H NMR using 1,1,2,2-tetrachloroethane as an internal standard. Ratio of isomers determined by ^{1}H NMR.*

Entry	*Reductant*	*Solvent*	*Yield (%)*	*dr*	***4i-d$_1$:4i***
1	***6a-d$_1$***	DMSO	56	88:12	70:30
2	***6a-d$_2$***	DMSO	52	88:12	> 90:10
3	***6a-d$_1$'***	DMSO	77	92:8	< 5:95
4	***6a***	DMSO-d$_6$	84	92:8	< 5:95

^{1}H NMR spectrum for Entry 1:

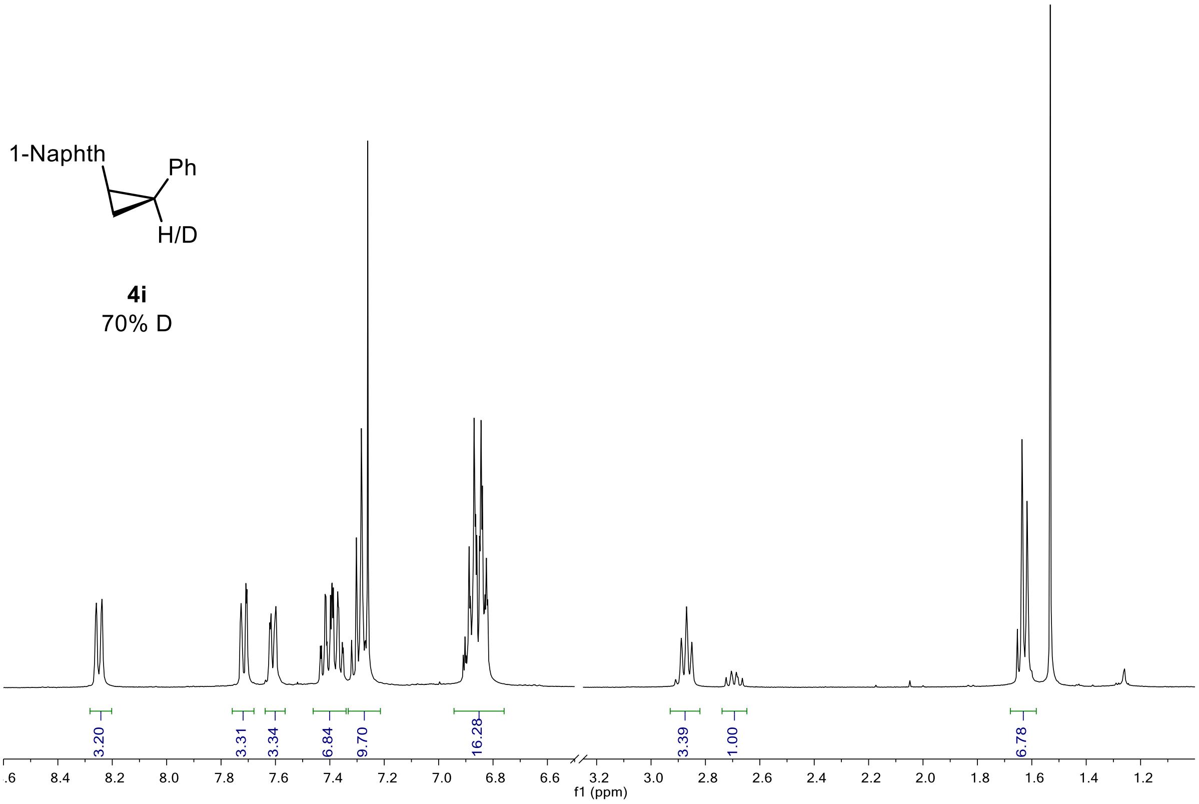

^{1}H NMR spectrum for Entry 2:

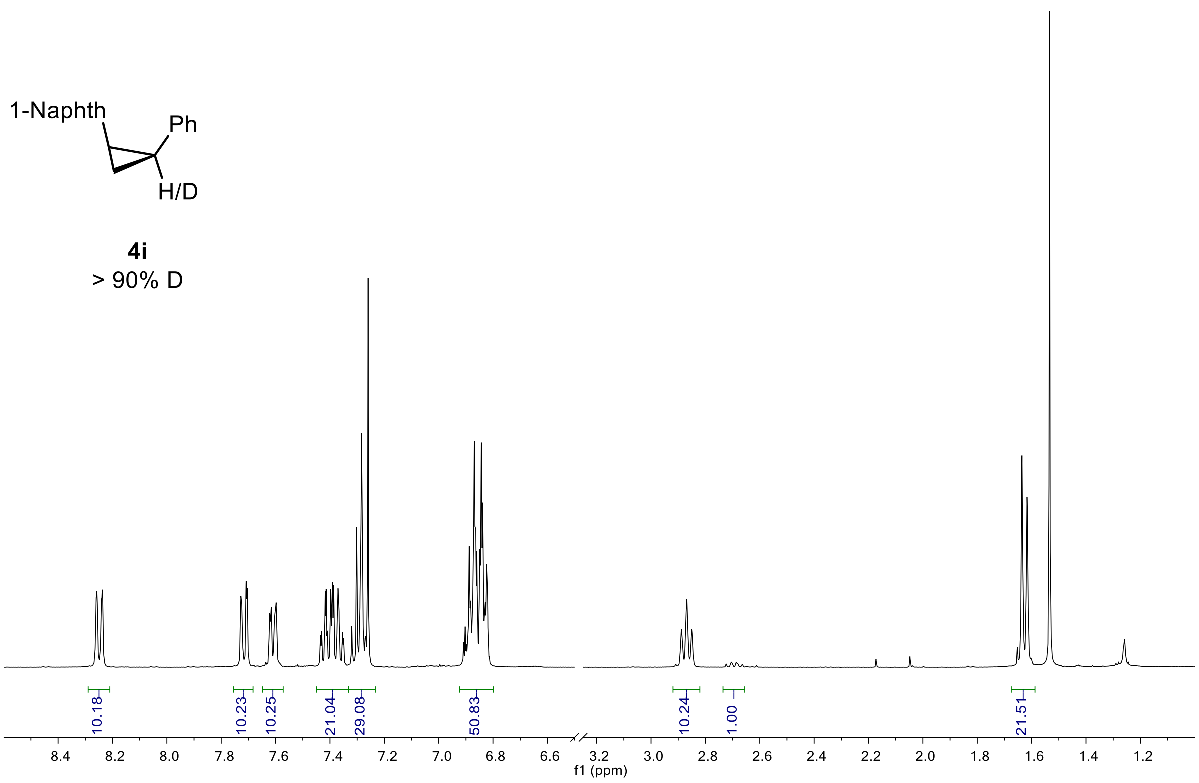

^{1}H NMR spectrum for Entry 3:

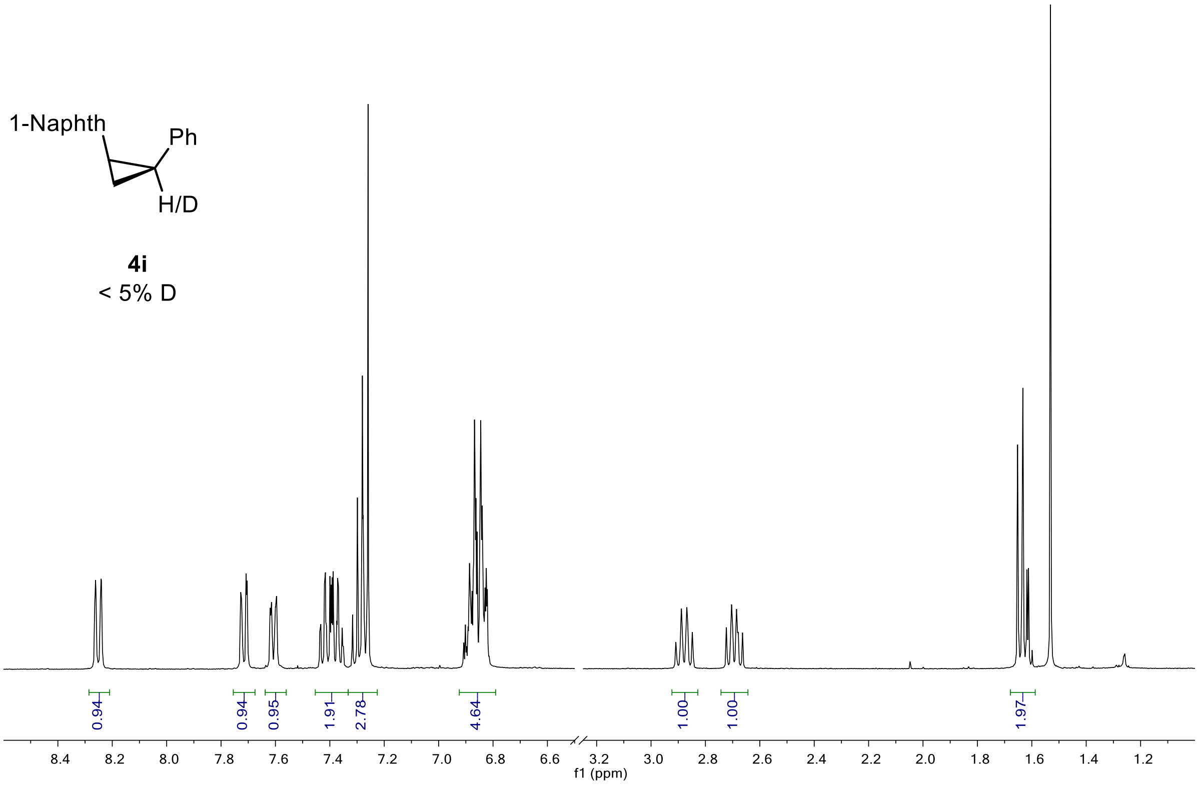

^{1}H NMR spectrum for Entry 4:

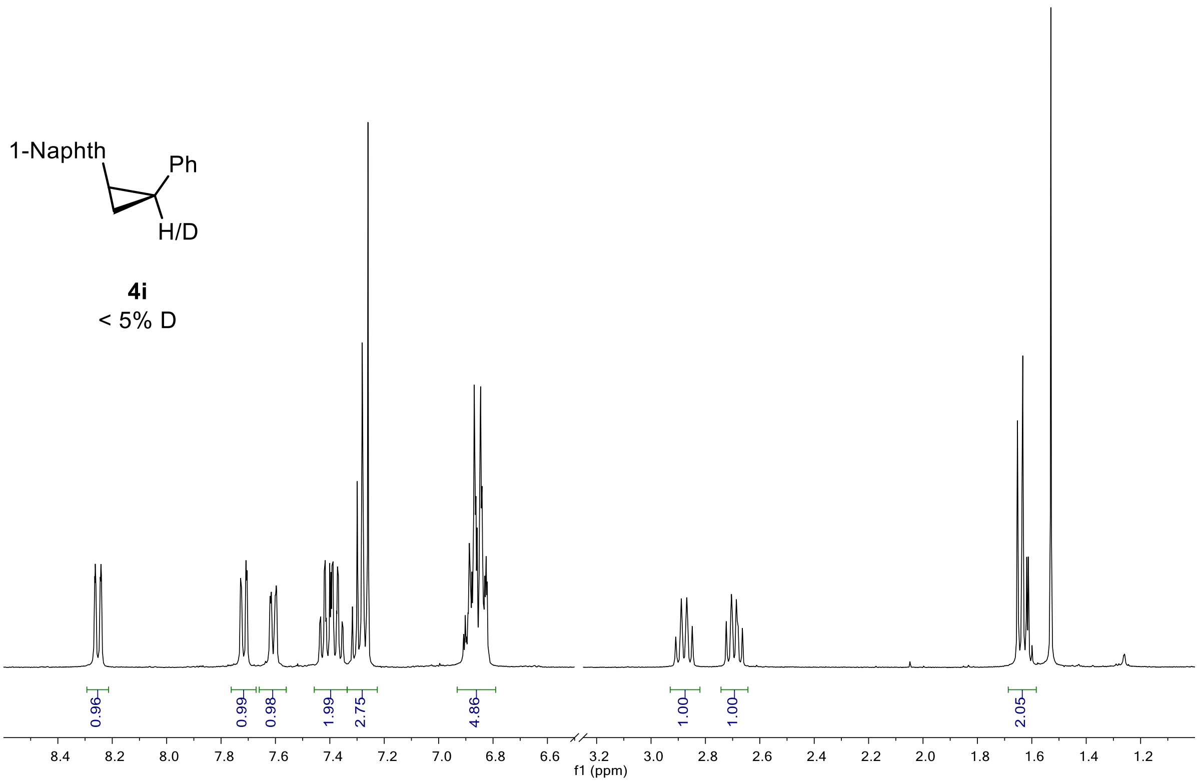

References:

[1] Z. Yu, A. Mendoza, *ACS Catal.* **2019**, *9*, 7870-7875.

[2] H. Chikashita, M. Miyazaki, K. Itoh, *J. Chem. Soc., Perkin Trans*. **1987**, *1*, 699-706.

[3] L. Han, Y. Zhang, W. Chen, X. Cheng, K. Ye, J. Zhang, Y. Wang, *Chem. Commun*. **2015**, *51*, 4477-4480.

[4] W. J. Seitz, M. M. Hossain, *Tetrahedron Lett.* **1994**, *35*, 7561-7564.

[5] É. Lévesque, S. R. Goudreau, A. B. Charette, *Org. Lett.* **2014**, *16*, 1490-1493.

[6] M.Verdecchia, C. Tubaro, A. Biffis, *Tetrahedron Lett.* **2011**, *52*, 1136-1139.

[7] G.-H. Fang, Z.-J. Yan, M.-Z. Deng, *Org. Lett.* **2004**, *6*, 357-360.

[8] Q. Wang, F. H. Försterling, M. M. Hossain, *J. Organomet. Chem*. **2005**, *690*, 6238-6246.

[9] G. D. Kishore Kumar, A. Natarajan, *Tetrahedron Lett.* **2008**, *49*, 2103-2105.

[10] N. Ty, R. Pontikis, G. G. Chabot, E. Devillers, L. Quentin, S. Bourg, J.-C. Florent, *Bioorg. Med. Chem*. **2013**, *21*, 1357-1366.

[11] M. A. Cismesiaa, T. P. Yoon, *Chem. Sci.*, **2015**, 6, 5426-5434.

[12] T. Sakamoto, K. Mori, T. Akiyama, *Org. Lett.* **2012**, *14*, 3312-3315.

[13] Y. Su, Q.-F. Li, Y.-M. Zhao, P. Gu, *Org. Lett.* **2016**, *18*, 4356-4359.

NMR spectra of synthesized compounds

^{1}H-NMR (400 MHz, $CDCl_3$) for 1v:

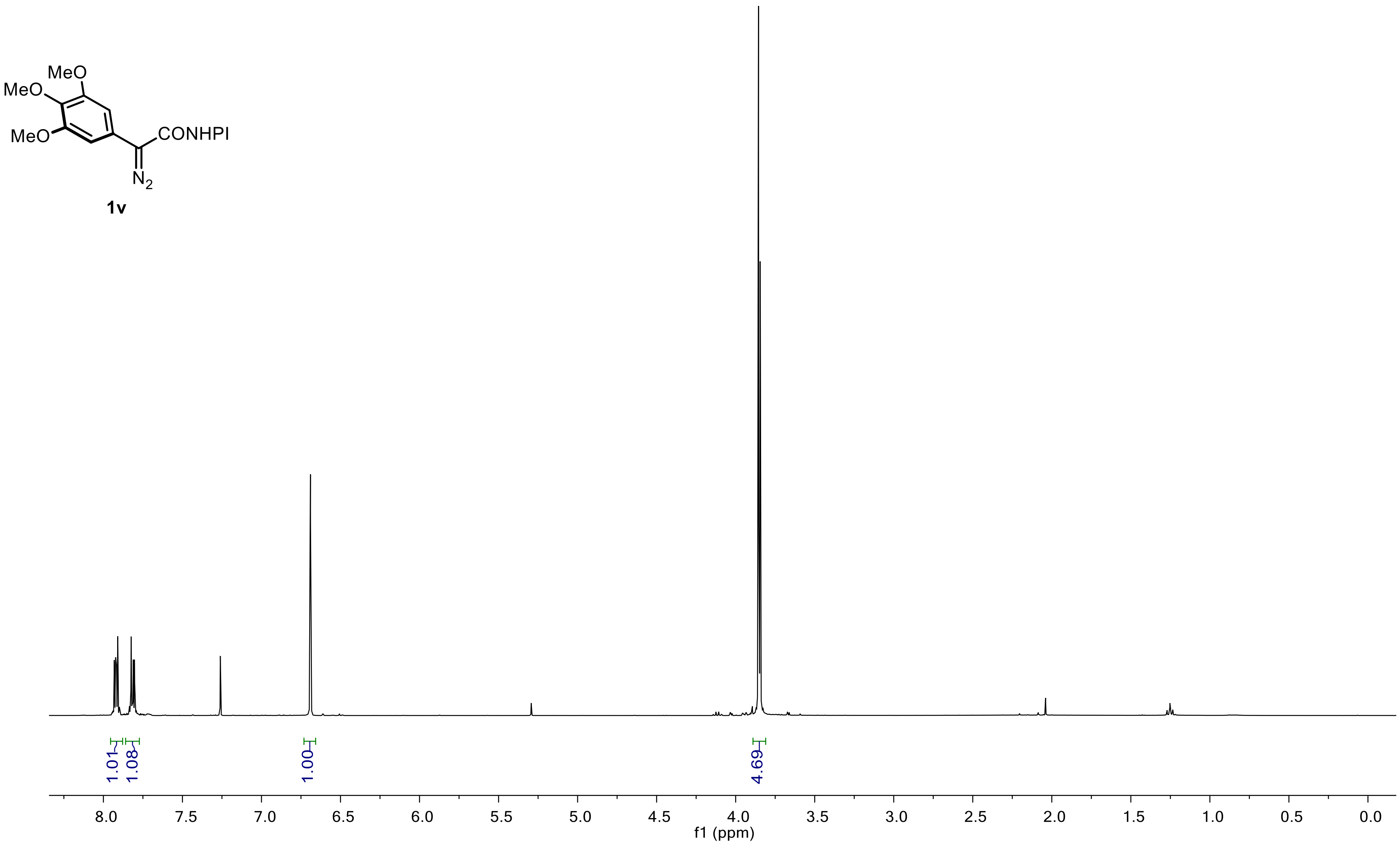

1v

^{13}C-NMR (101 MHz, $CDCl_3$) for 1v:

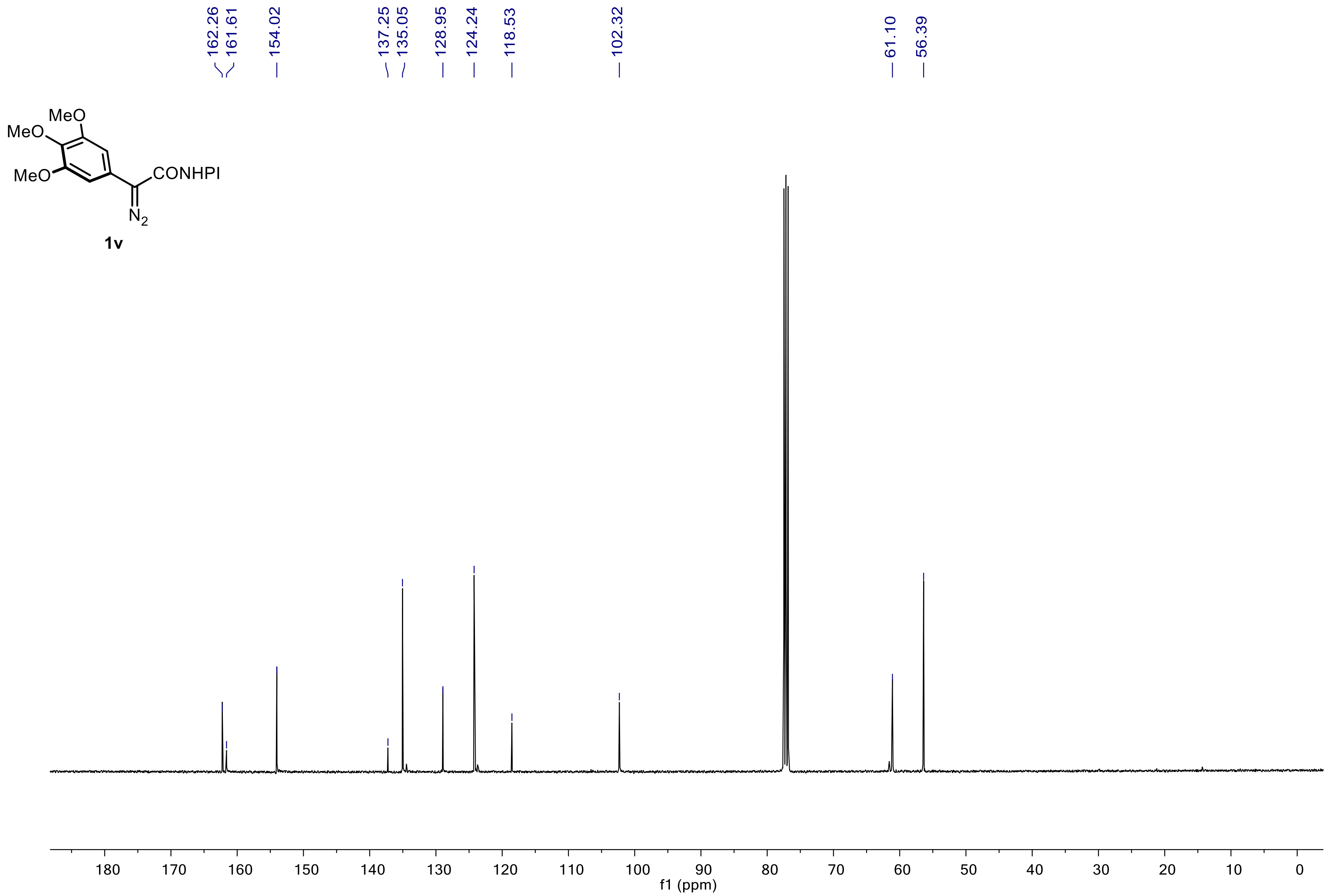

^{1}H-NMR (400 MHz, $CDCl_3$) for S3b:

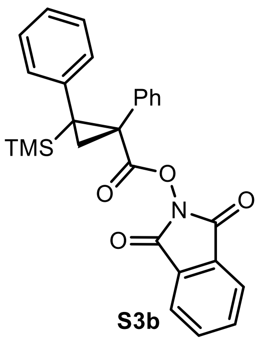

S3b

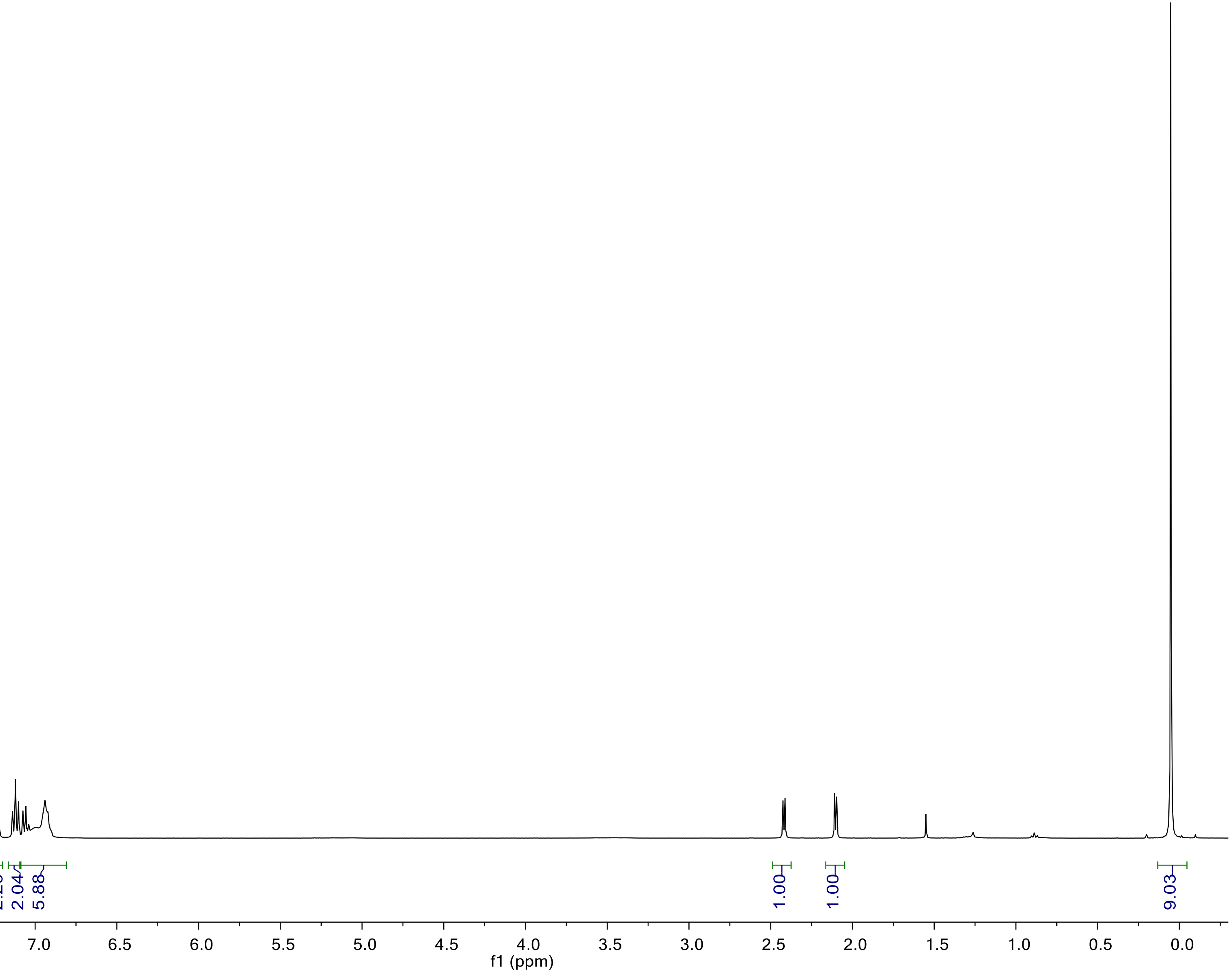

^{13}C-NMR (101 MHz, $CDCl_3$) for S3b:

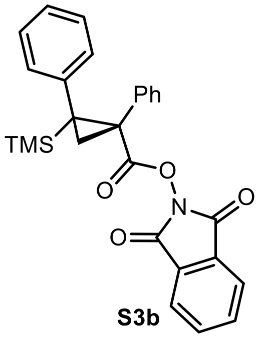

S3b

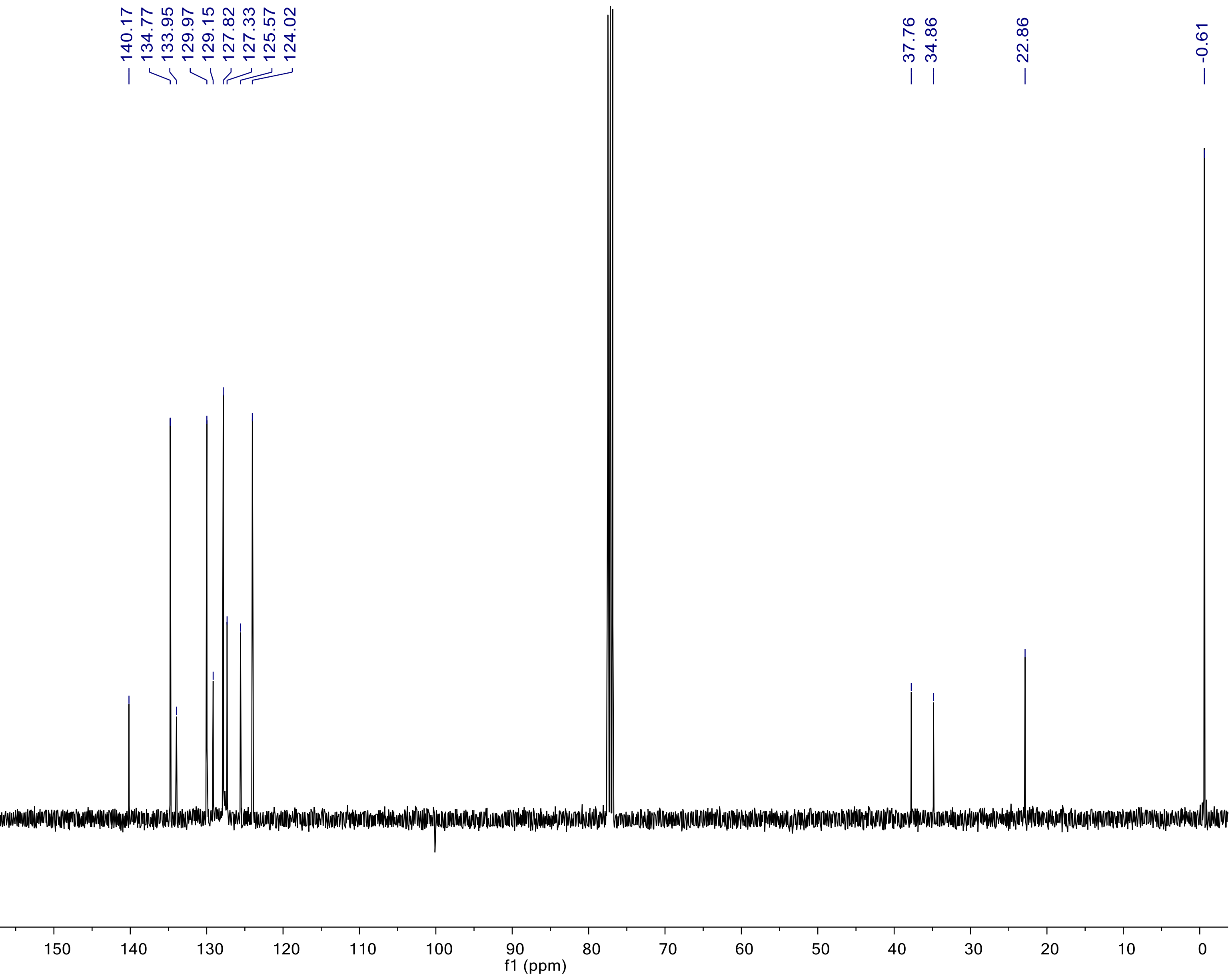

^{1}H-NMR (400 MHz, $CDCl_3$) for *diast*-3a:

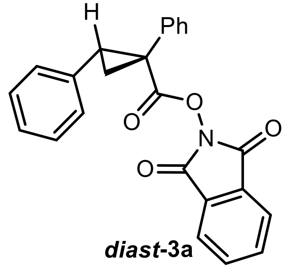

***diast*-3a**

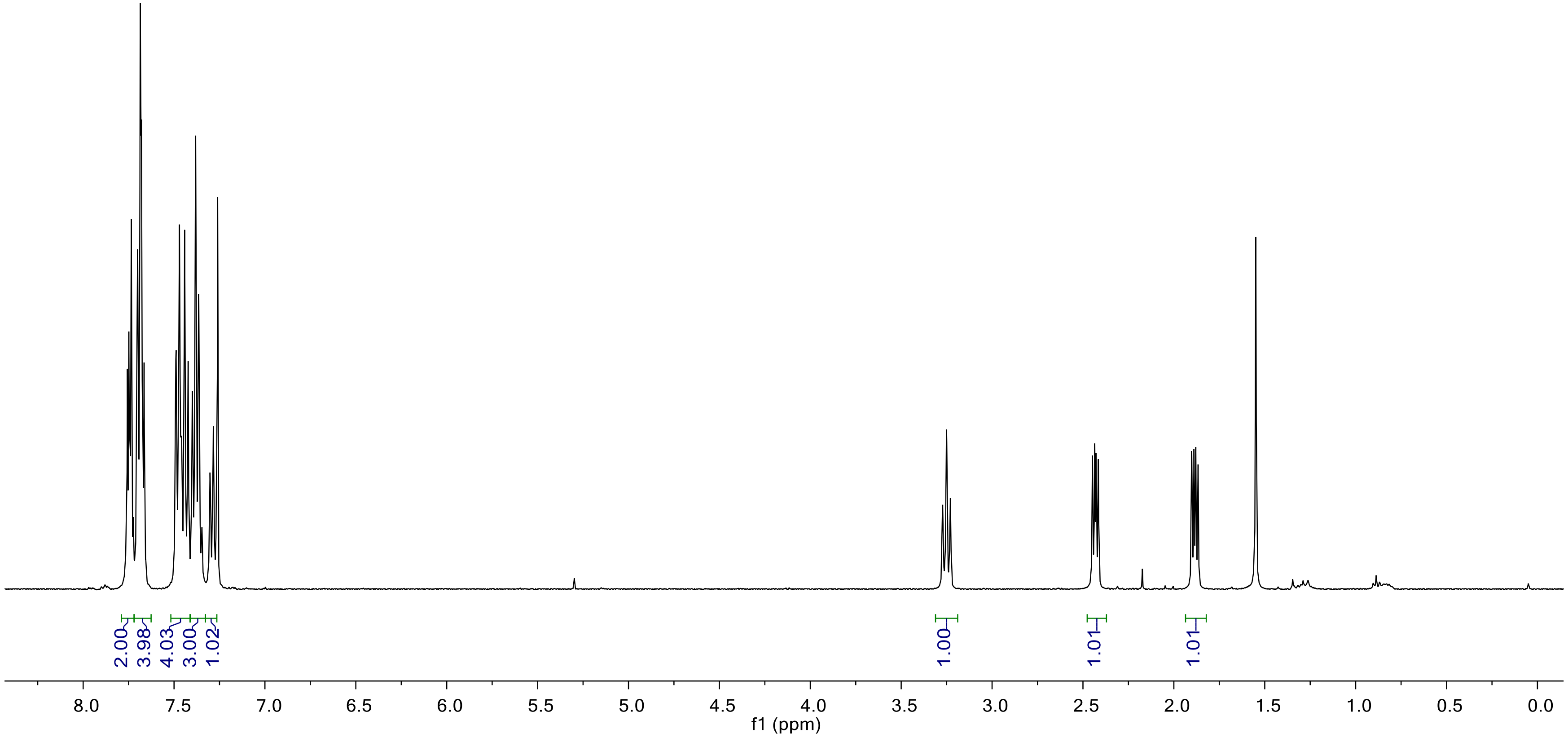

^{13}C-NMR (101 MHz, $CDCl_3$) for *diast*-3a:

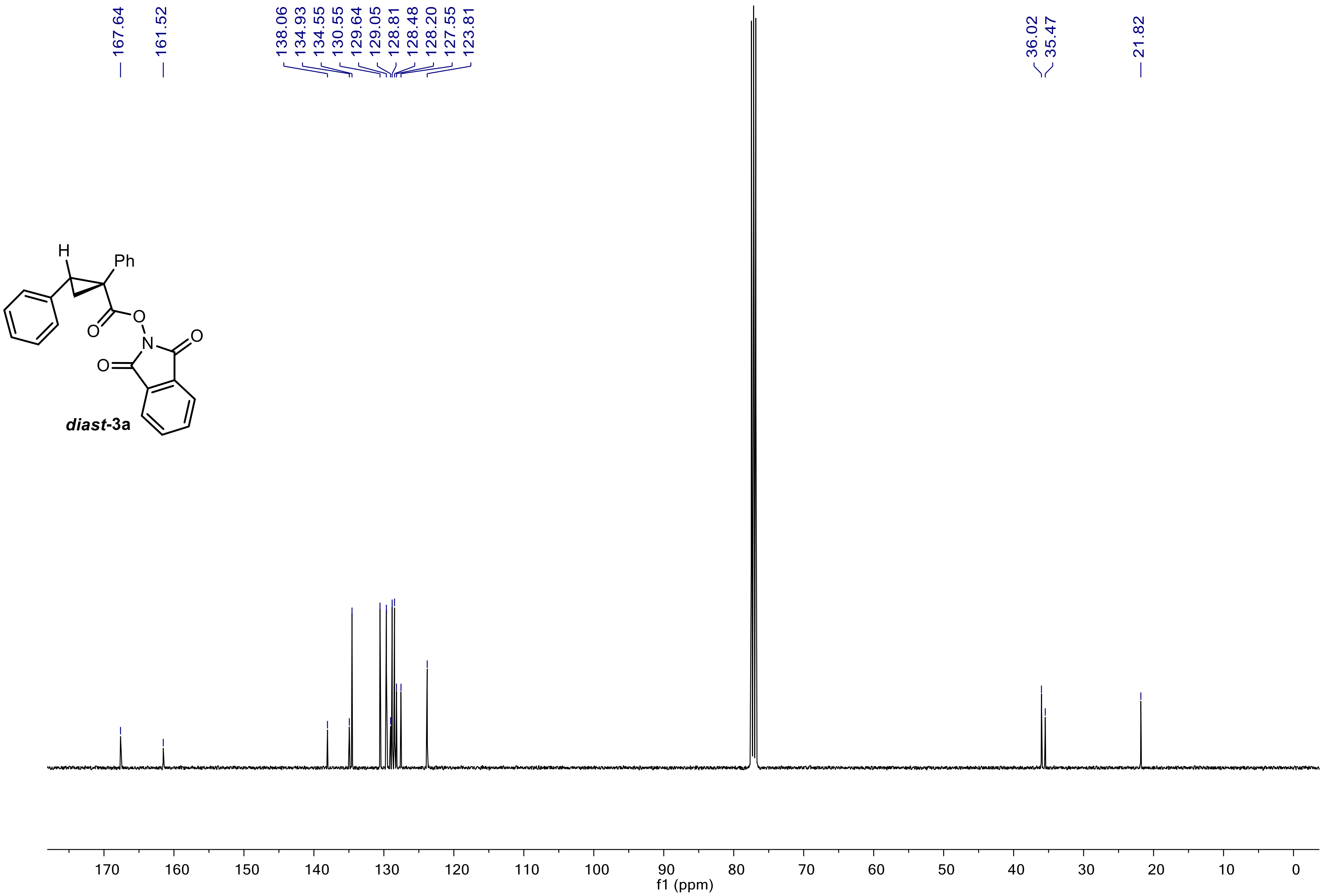

***diast*-3a**

^{1}H-NMR (400 MHz, $CDCl_3$) for 4b:

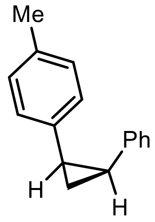

4b

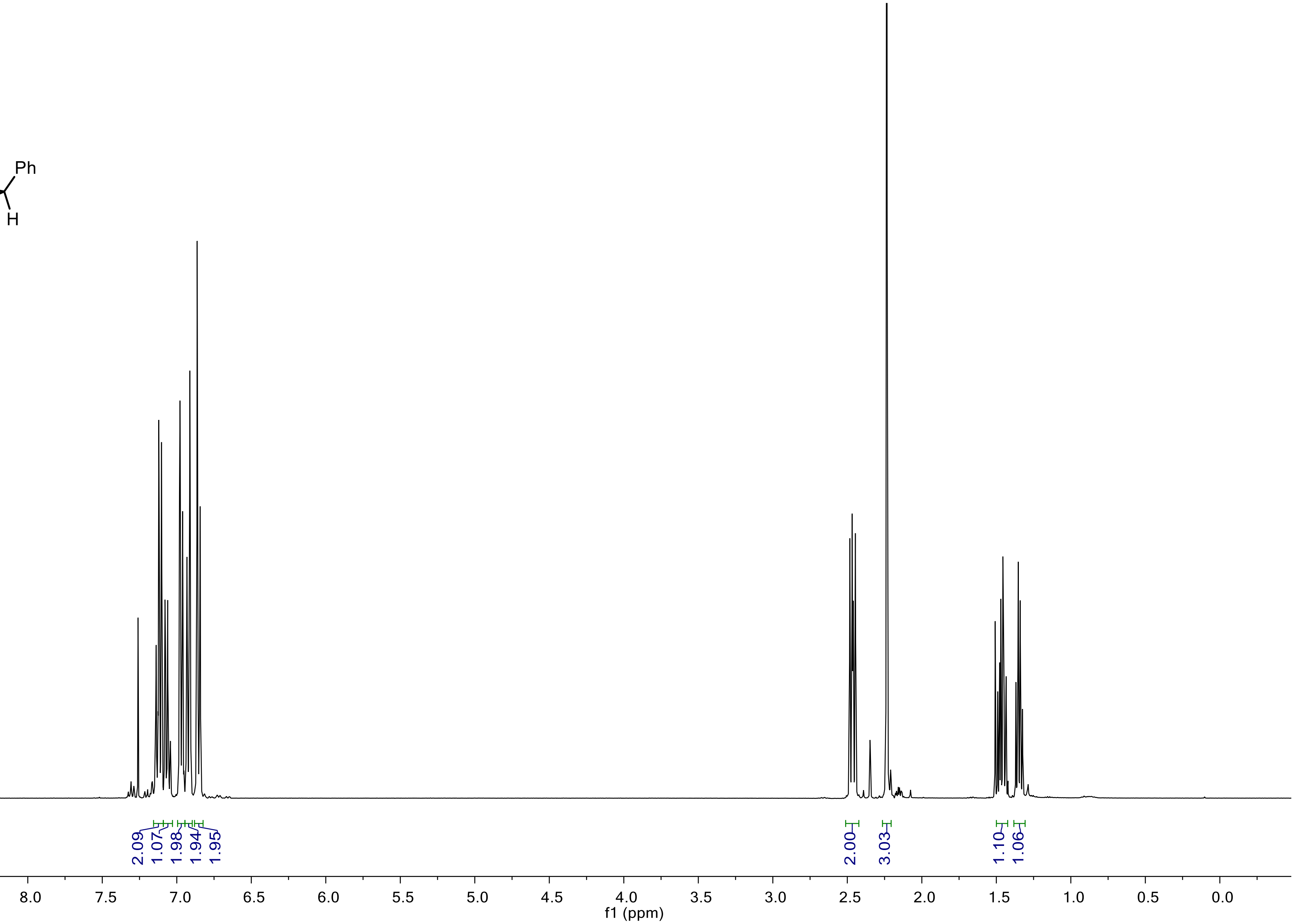

^{13}C-NMR (101 MHz, $CDCl_3$) for 4b:

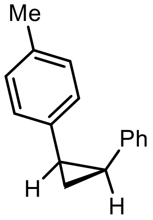

4b

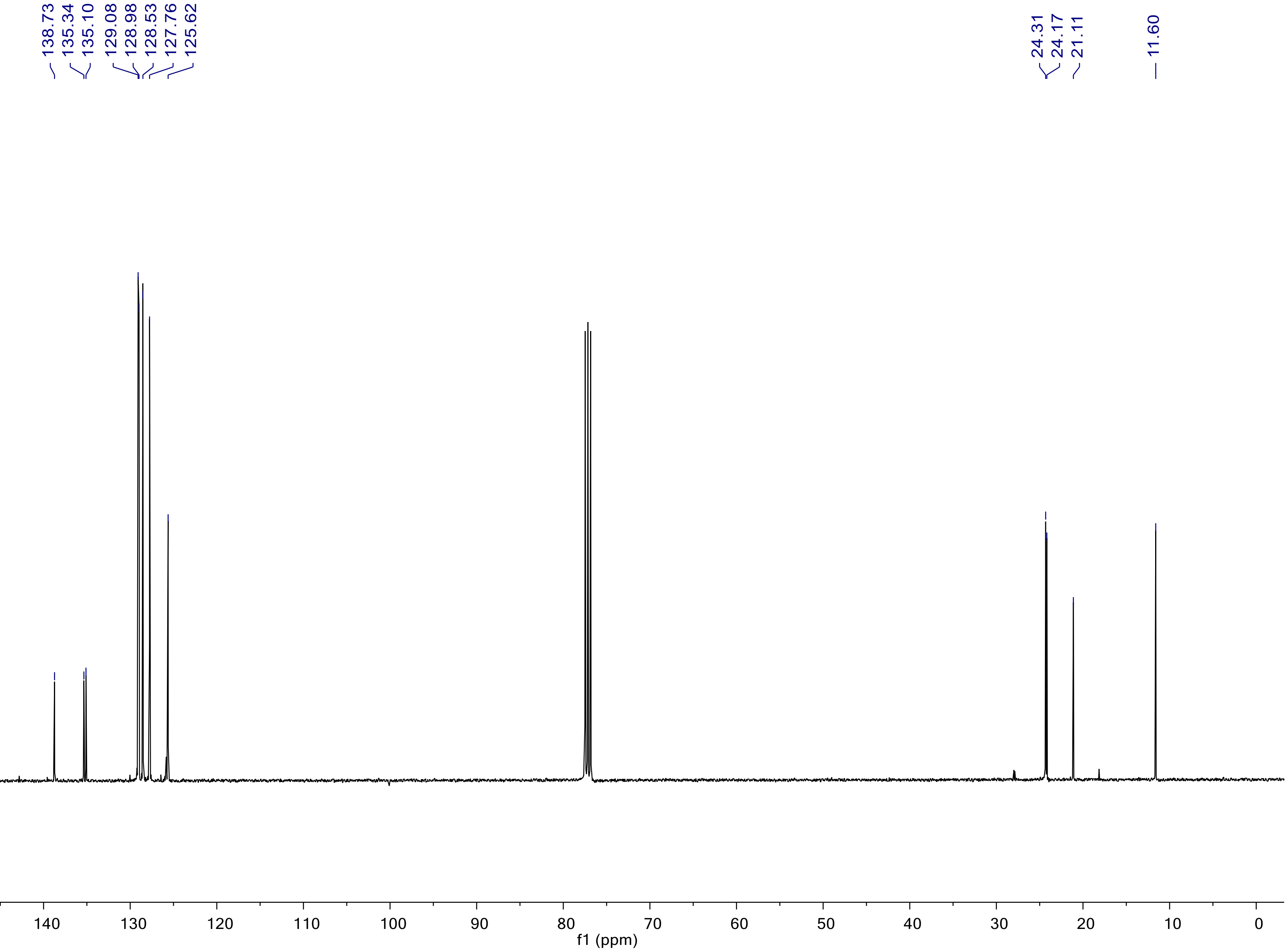

^{1}H-NMR (400 MHz, $CDCl_3$) for 4c:

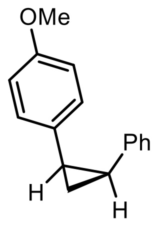

4c

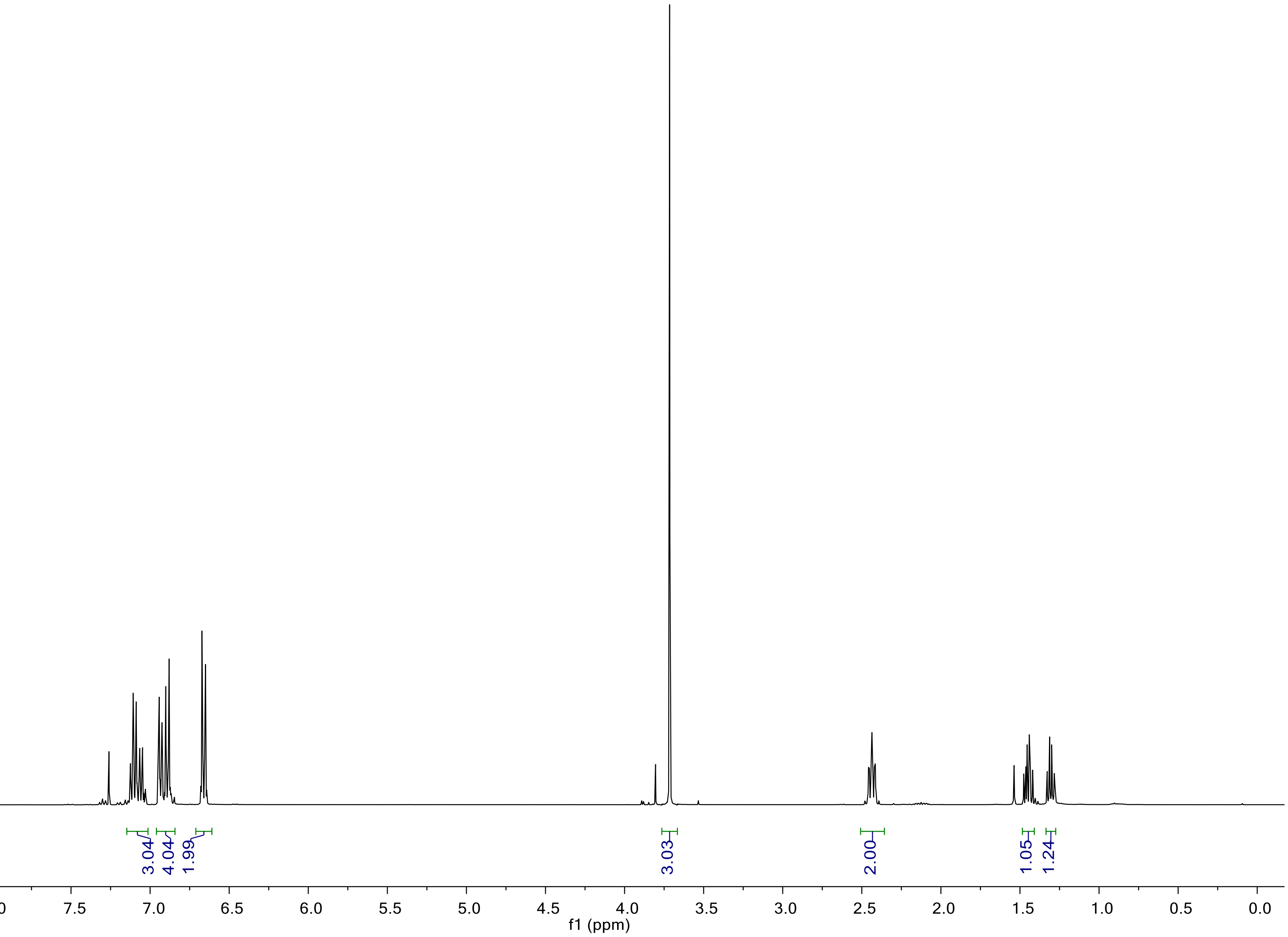

^{13}C-NMR (101 MHz, CDCl$_3$) for 4c:

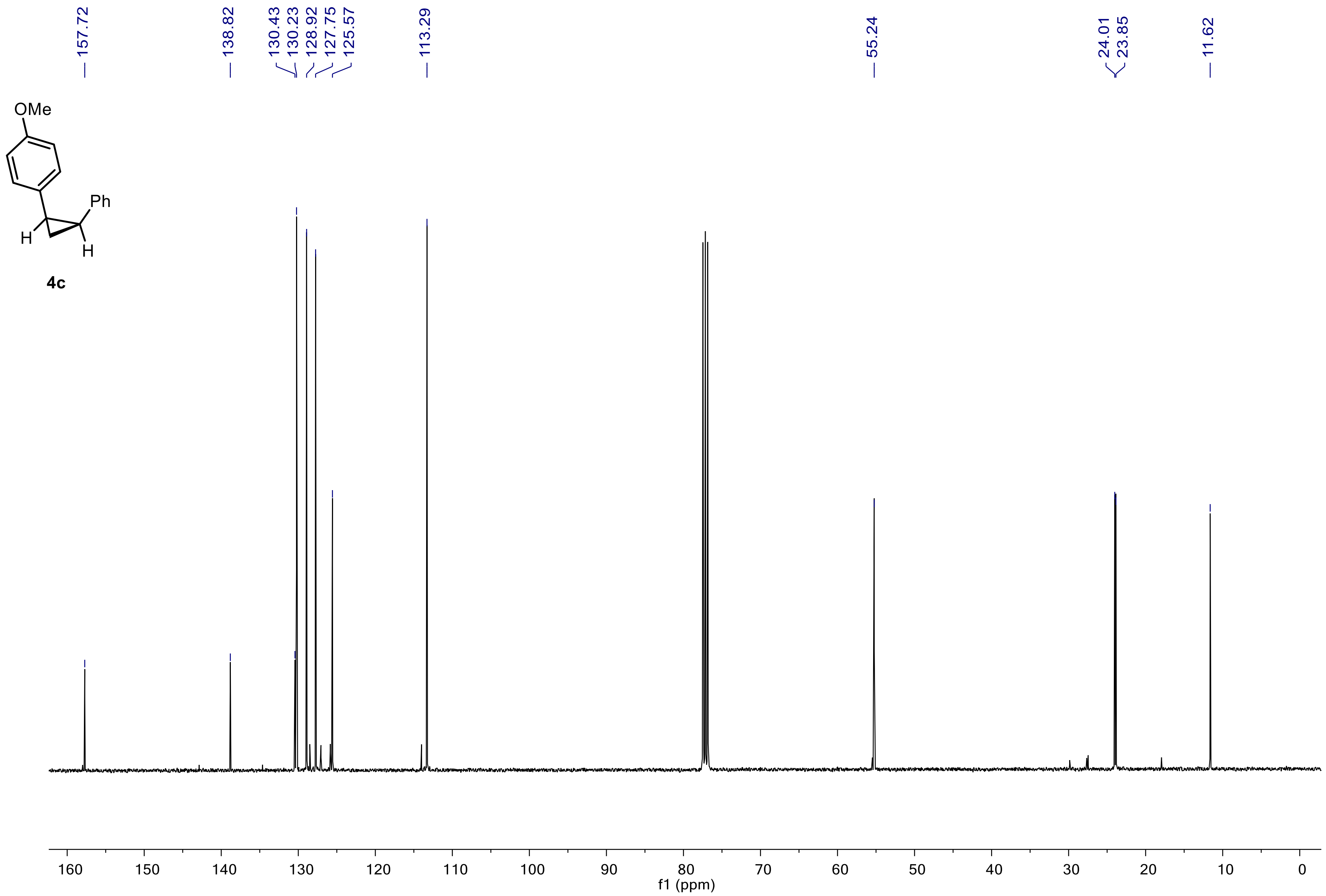

^{1}H-NMR (400 MHz, $CDCl_3$) for 4d:

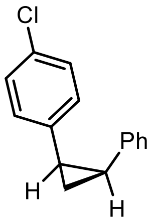

4d

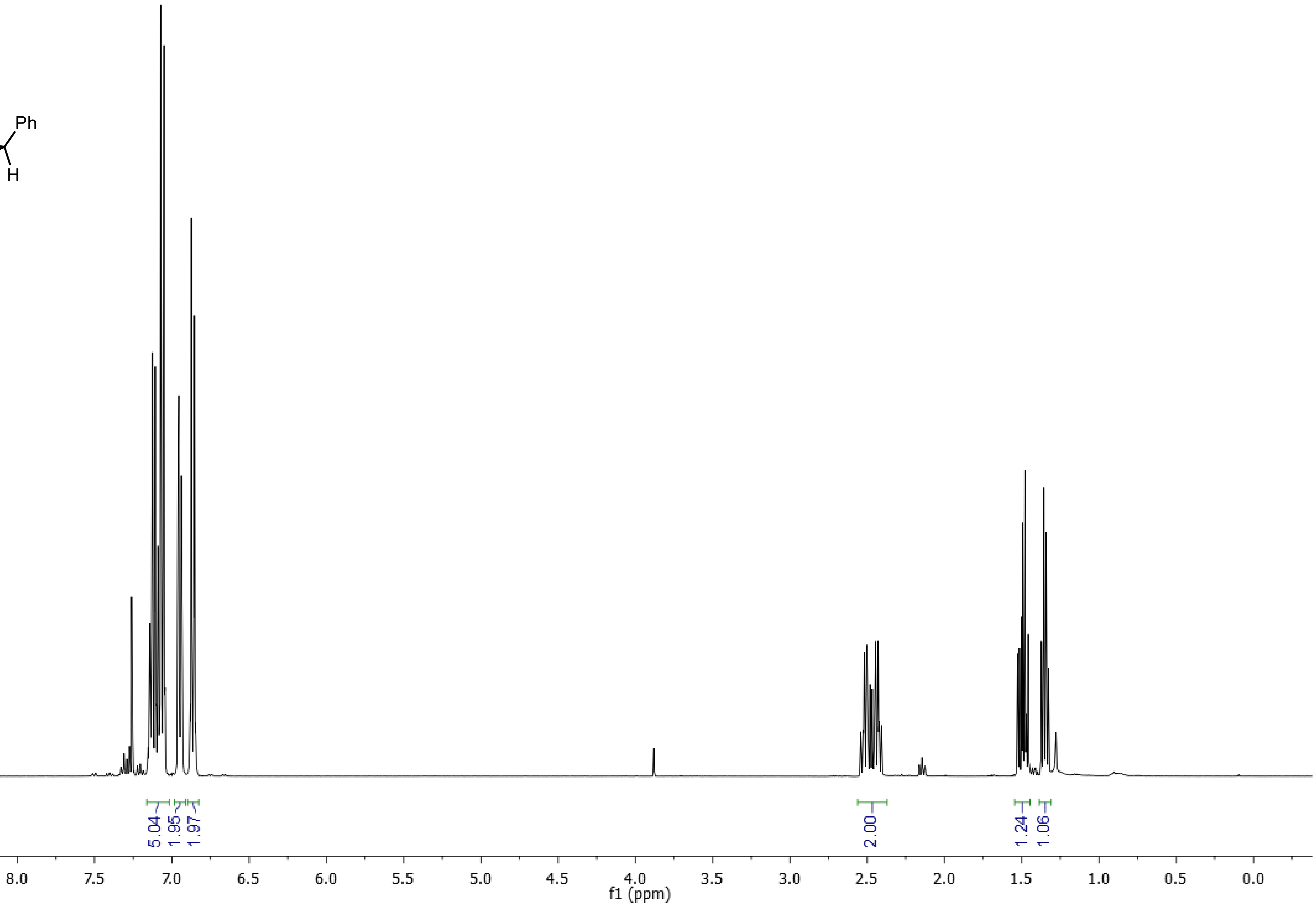

^{13}C-NMR (101 MHz, $CDCl_3$) for 4d:

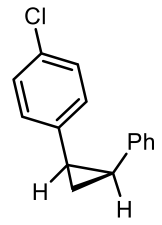

4d

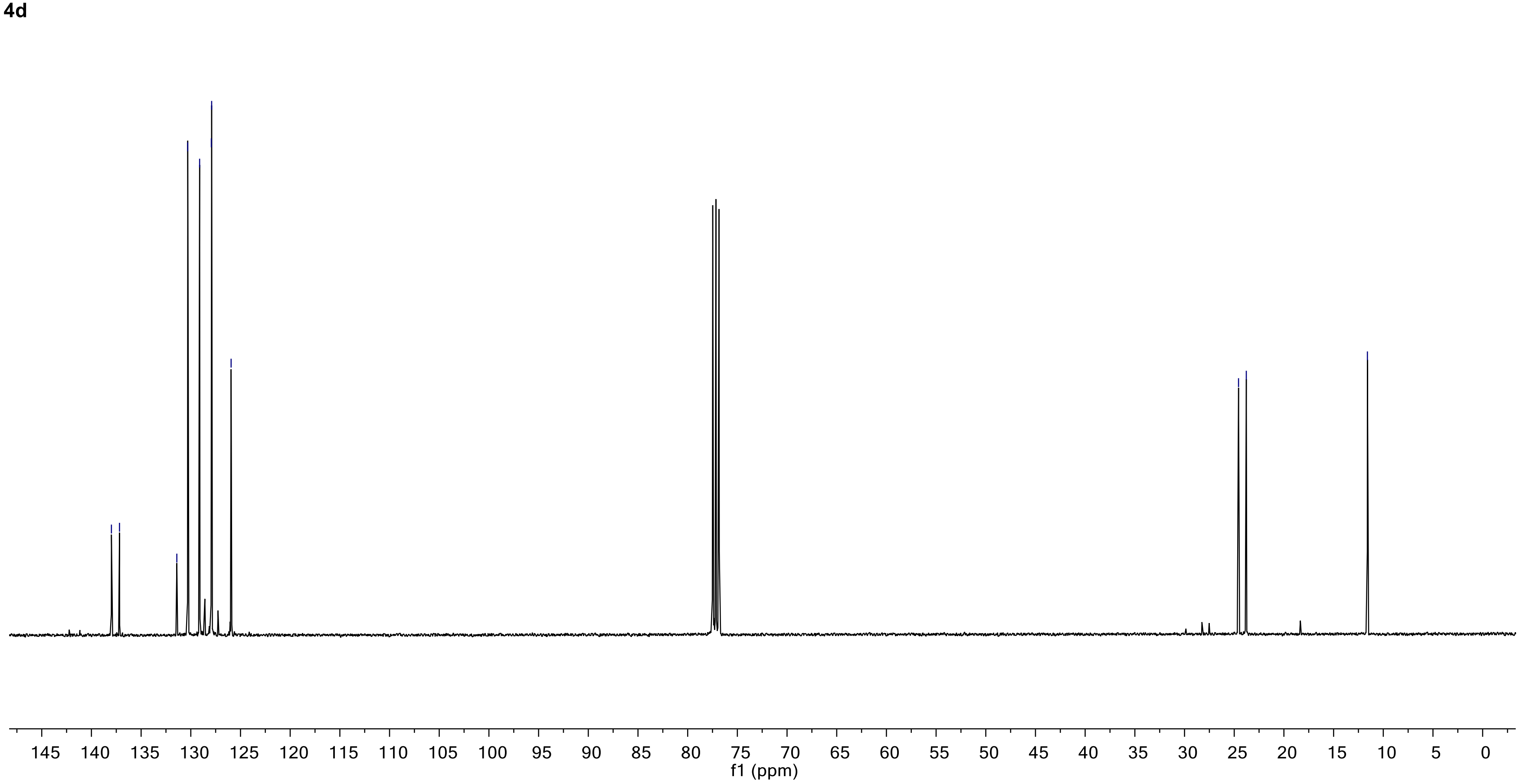

^{1}H-NMR (400 MHz, $CDCl_3$) for 4e:

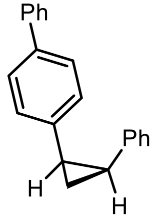

4e

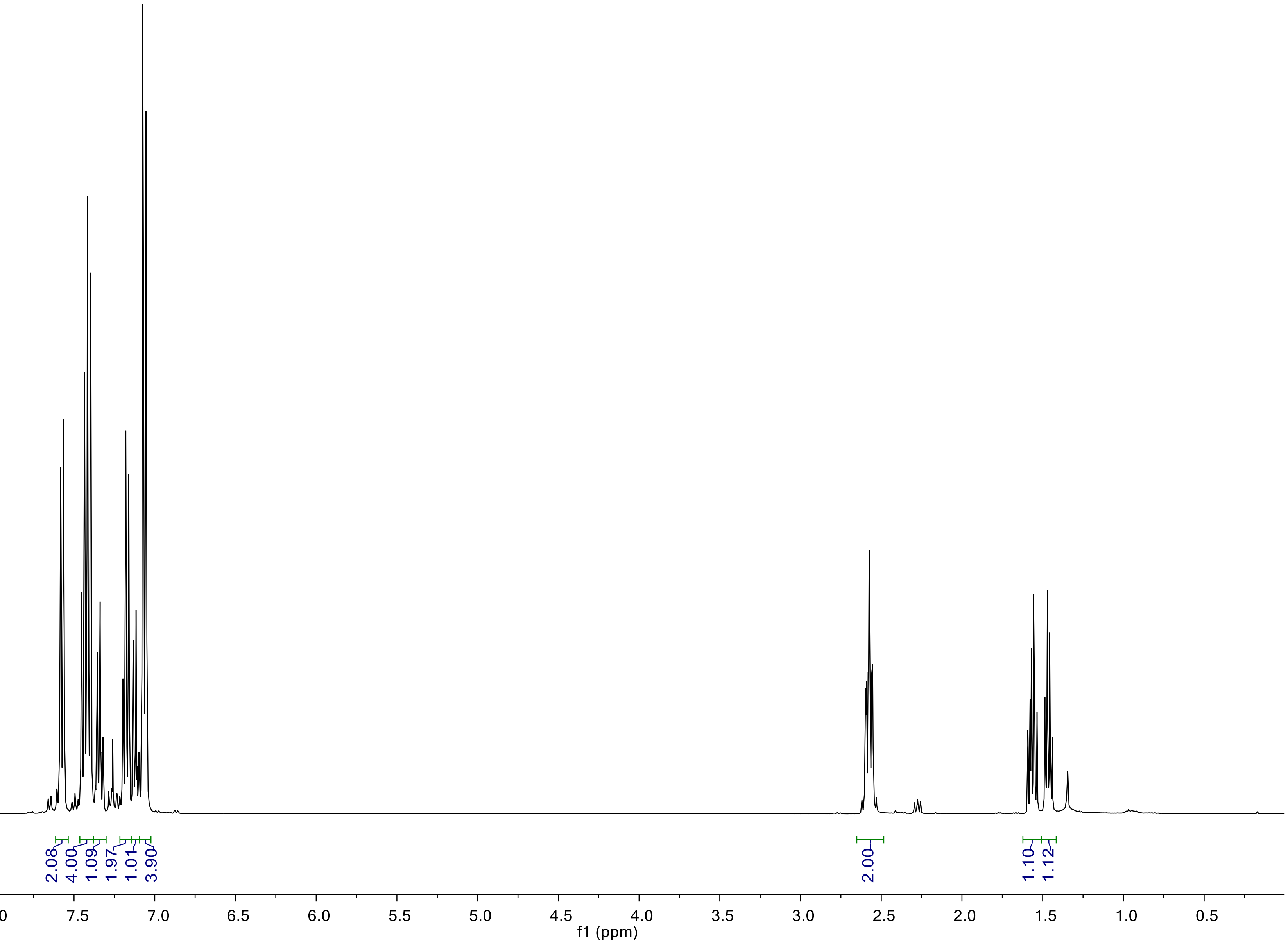

^{13}C-NMR (101 MHz, $CDCl_3$) for 4e:

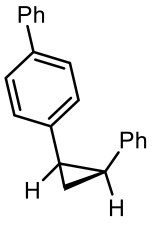

4e

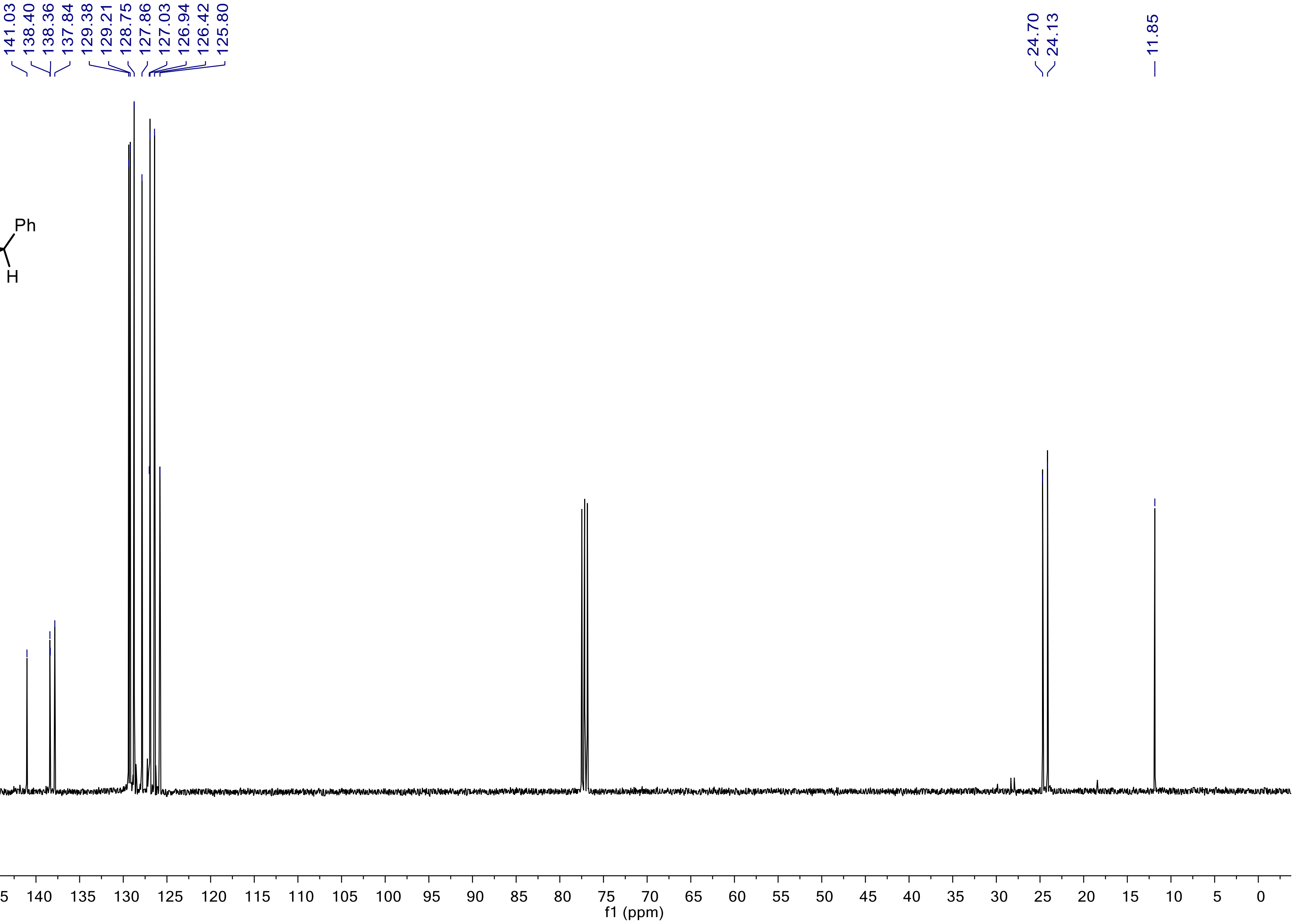

^{1}H-NMR (400 MHz, $CDCl_3$) for 4f:

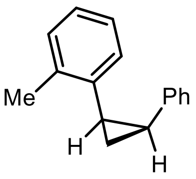

4f

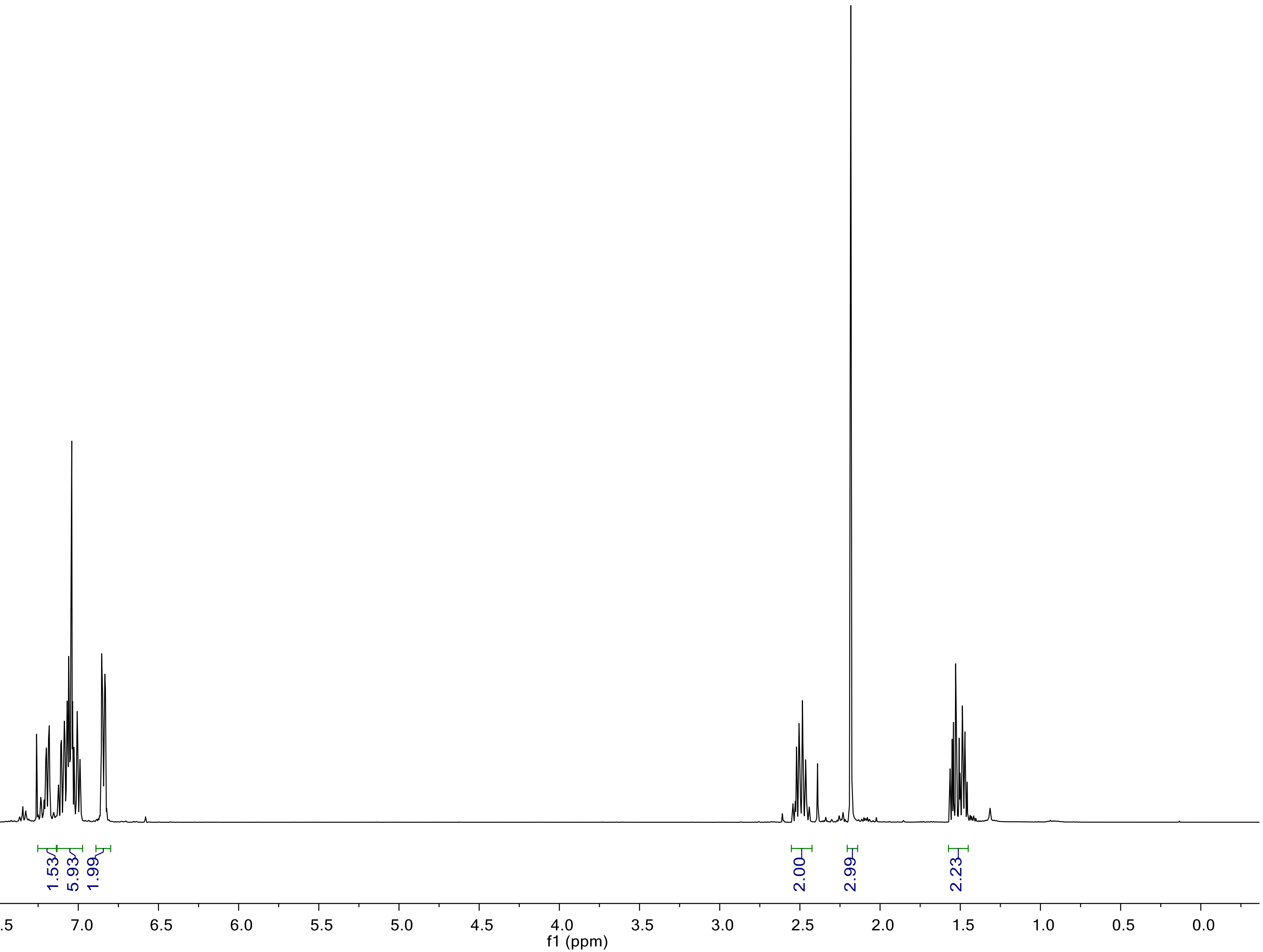

^{13}C-NMR (101 MHz, $CDCl_3$) for 4f:

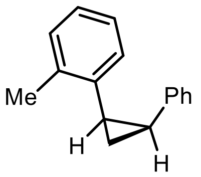

4f

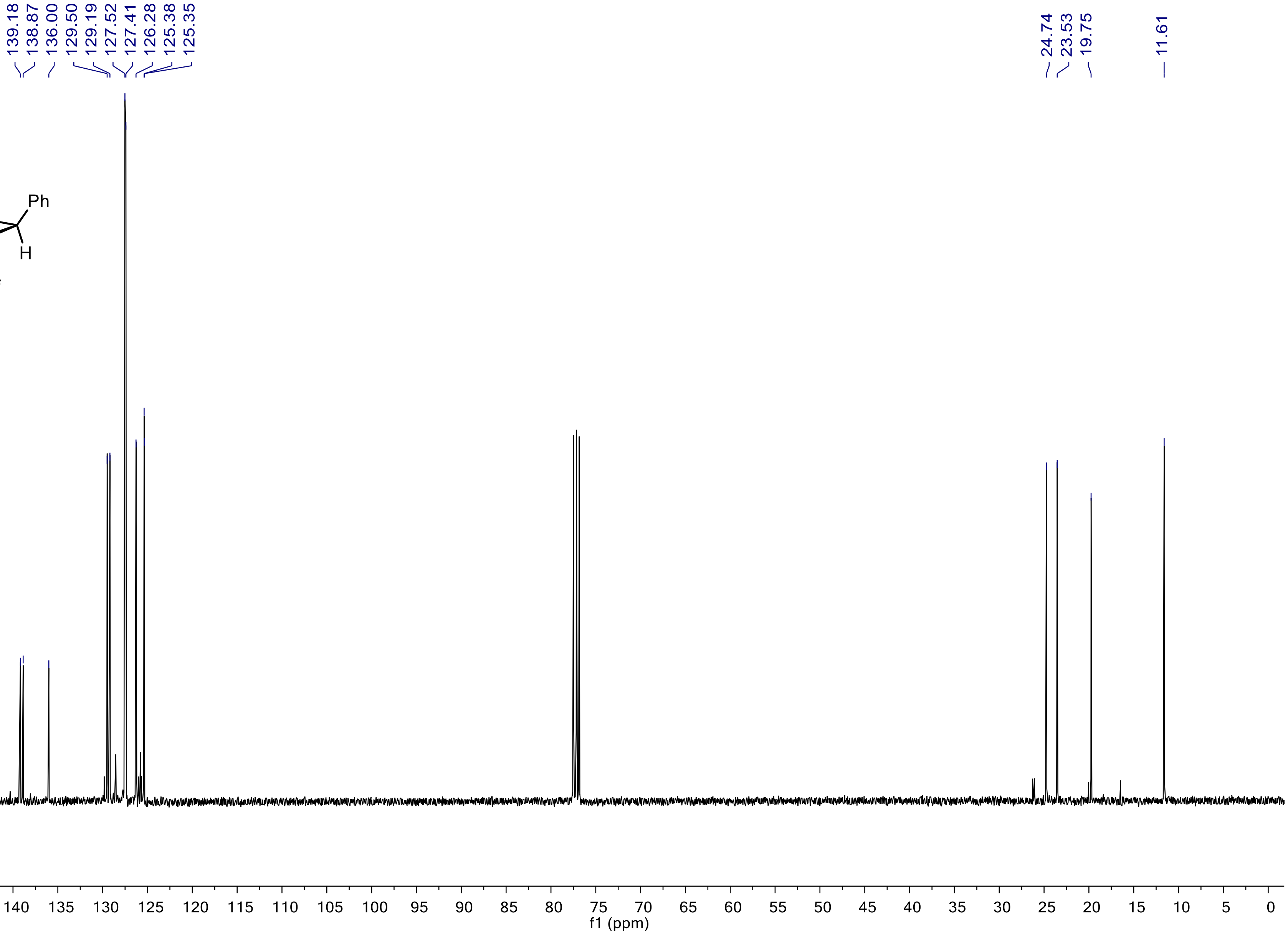

^{1}H-NMR (400 MHz, $CDCl_3$) for 4g:

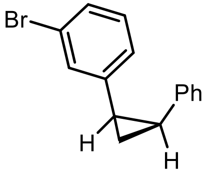

4g

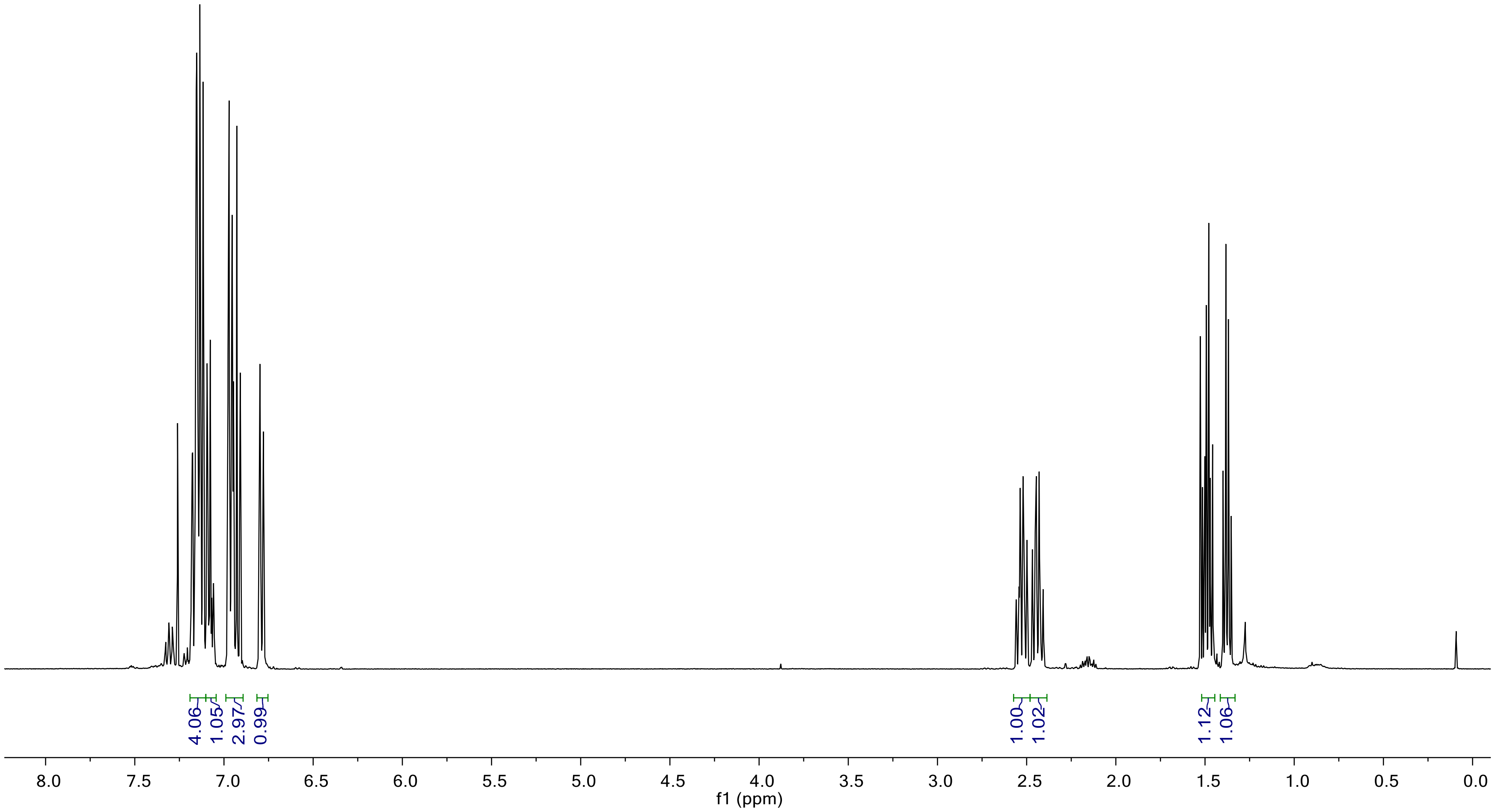

^{13}C-NMR (101 MHz, $CDCl_3$) for 4g:

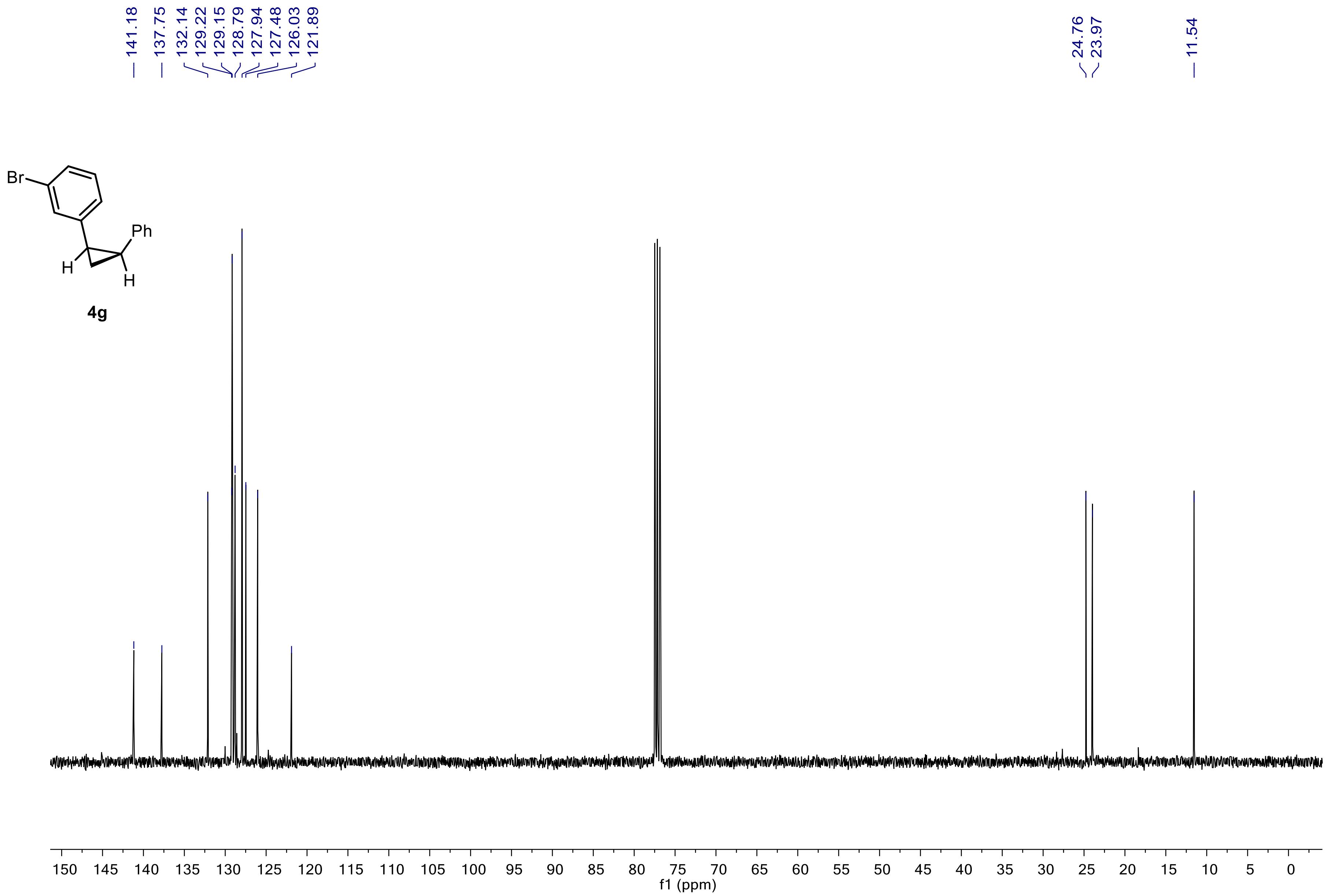

^{1}H-NMR (400 MHz, $CDCl_3$) for 4h:

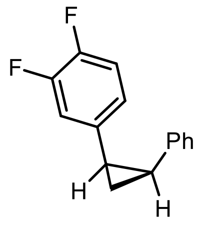

4h

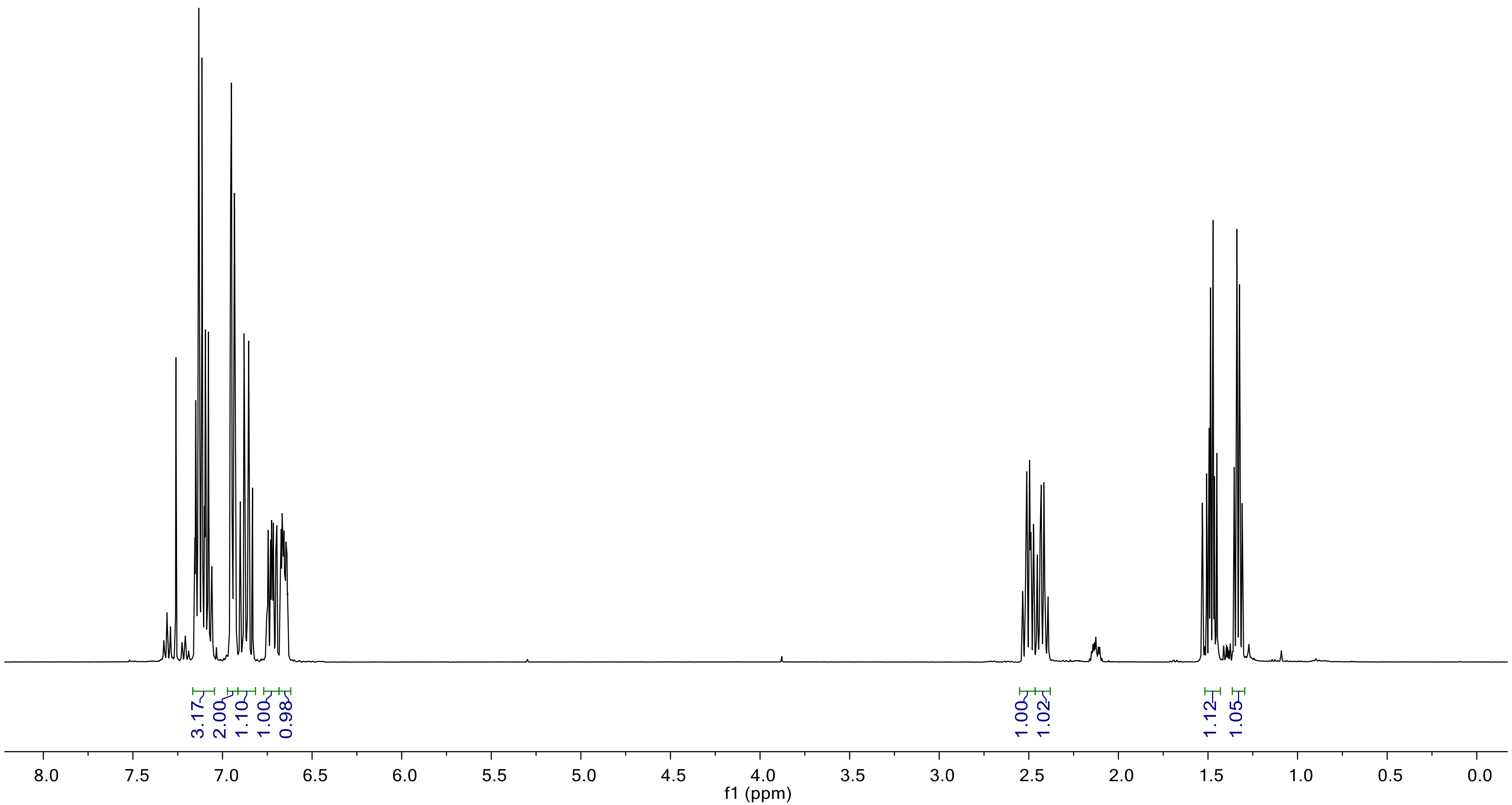

^{13}C-NMR (101 MHz, $CDCl_3$) for 4h:

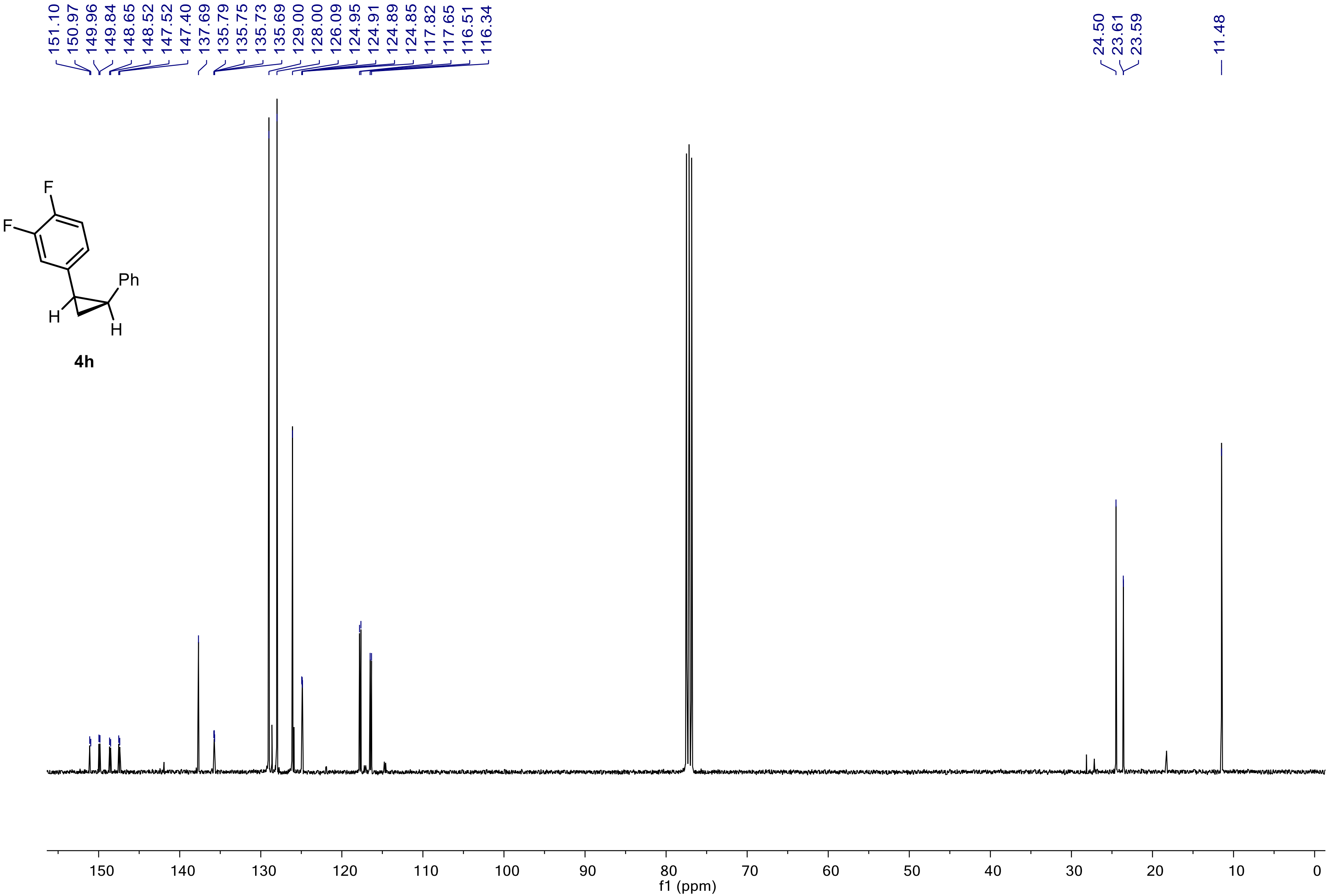

^{19}F-NMR (377 MHz, $CDCl_3$) for 4h:

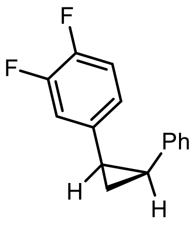

4h

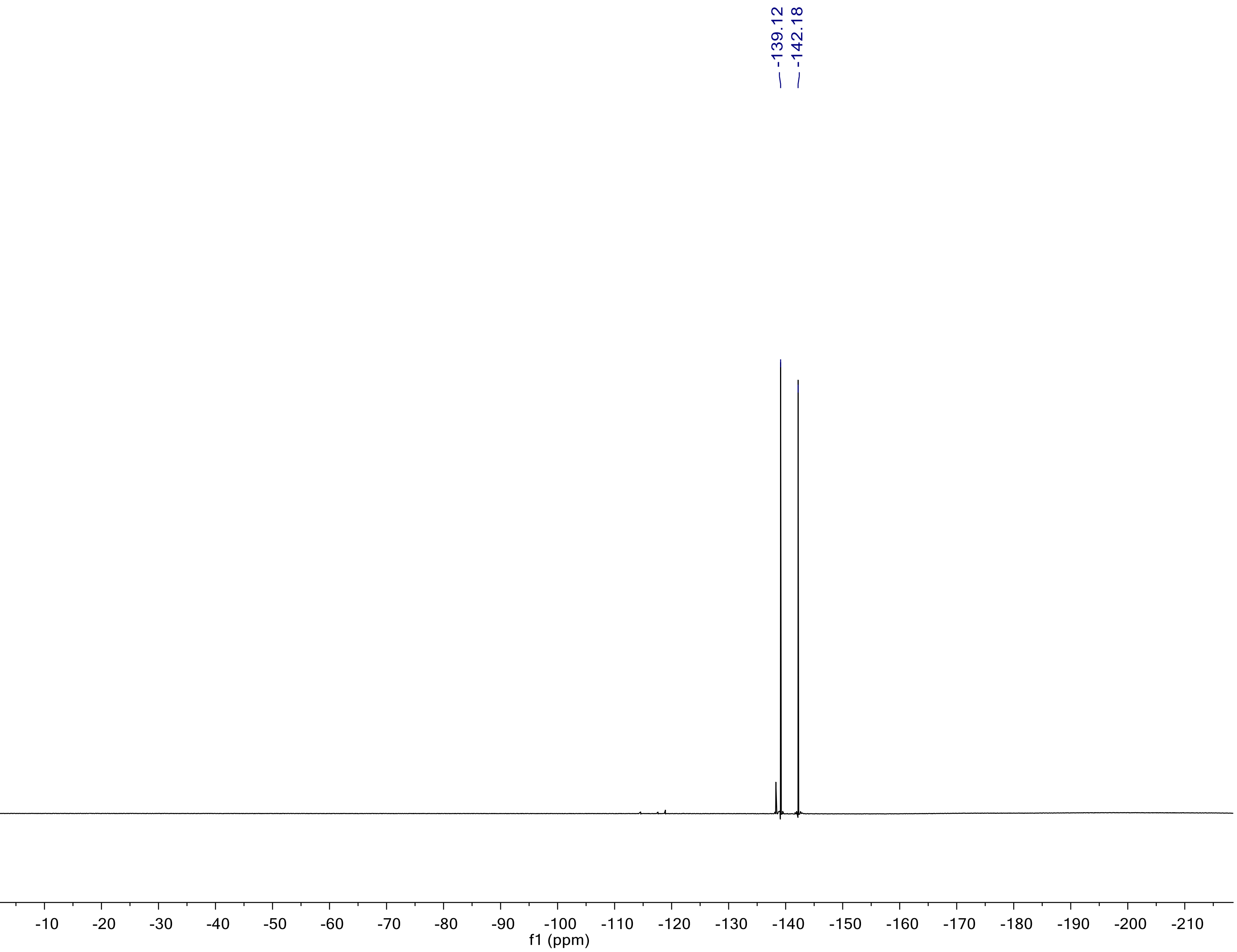

^{1}H-NMR (400 MHz, $CDCl_3$) for 4i:

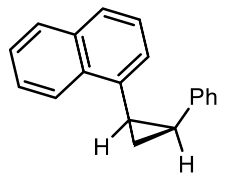

4i

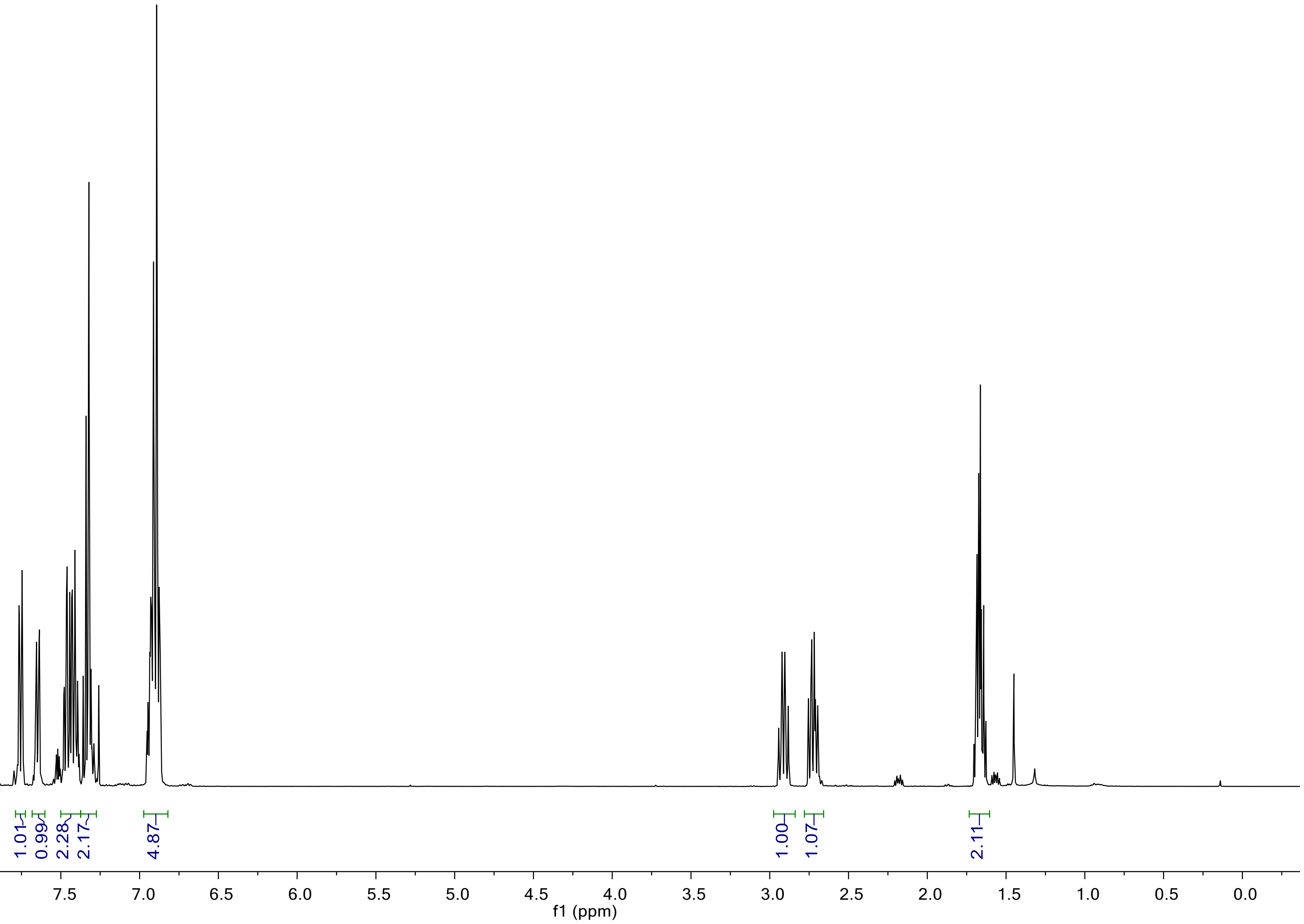

^{13}C-NMR (101 MHz, $CDCl_3$) for 4i:

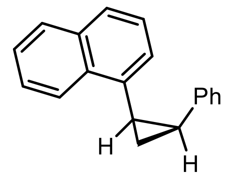

4i

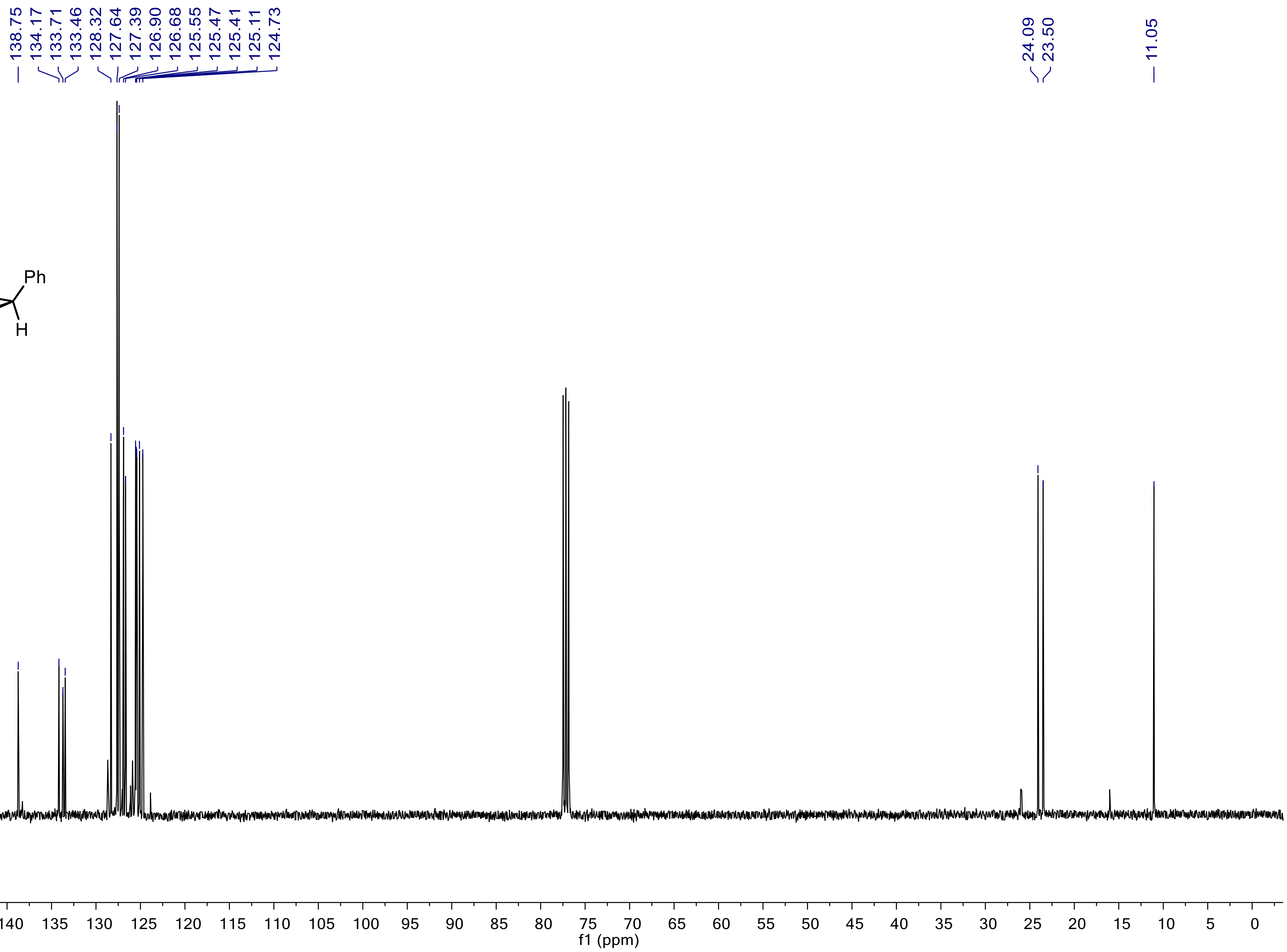

^{1}H-NMR (400 MHz, $CDCl_3$) for 4j:

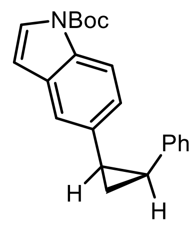

4j

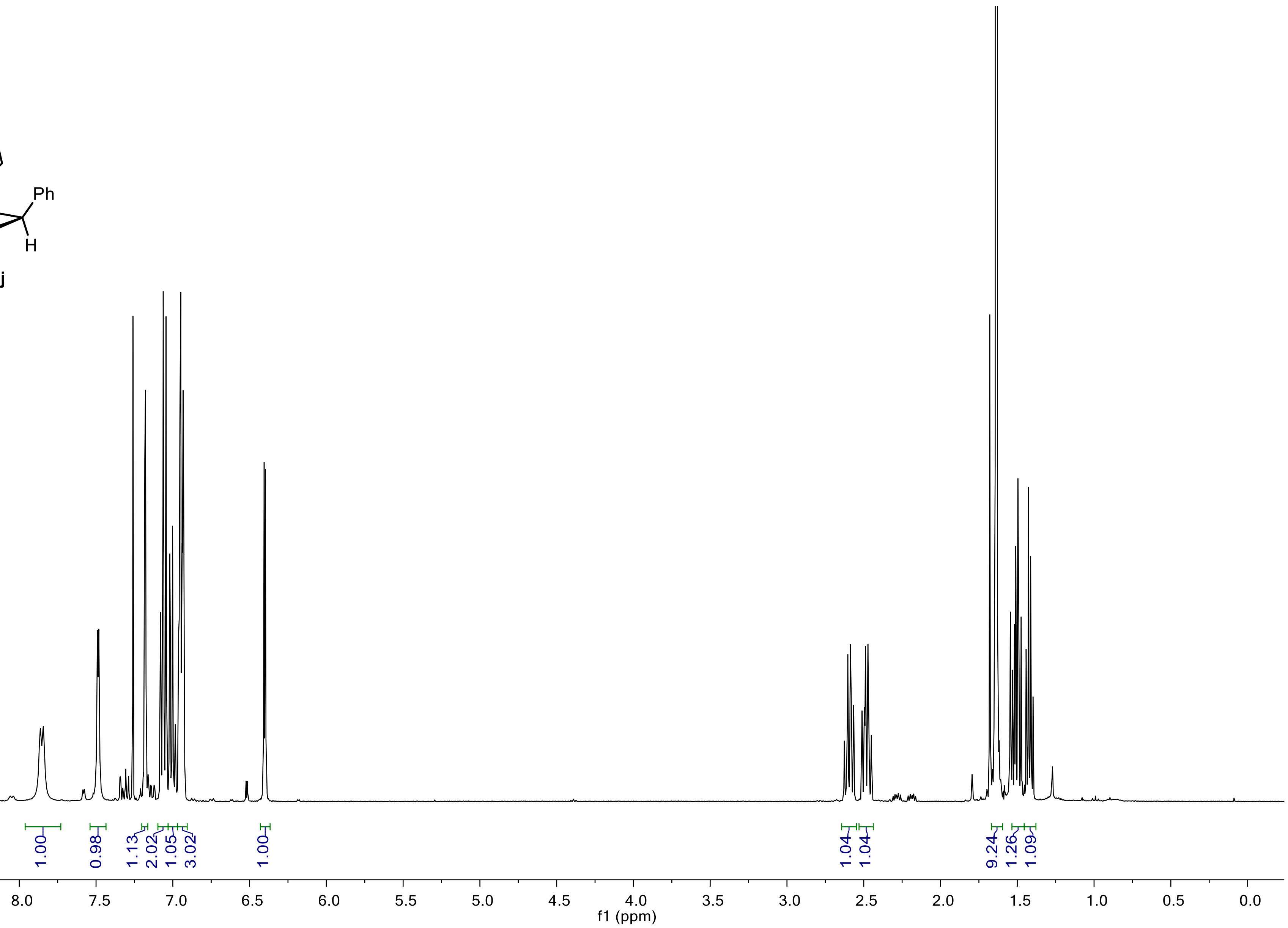

^{13}C-NMR (101 MHz, $CDCl_3$) for 4j:

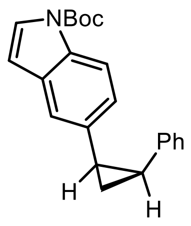

4j

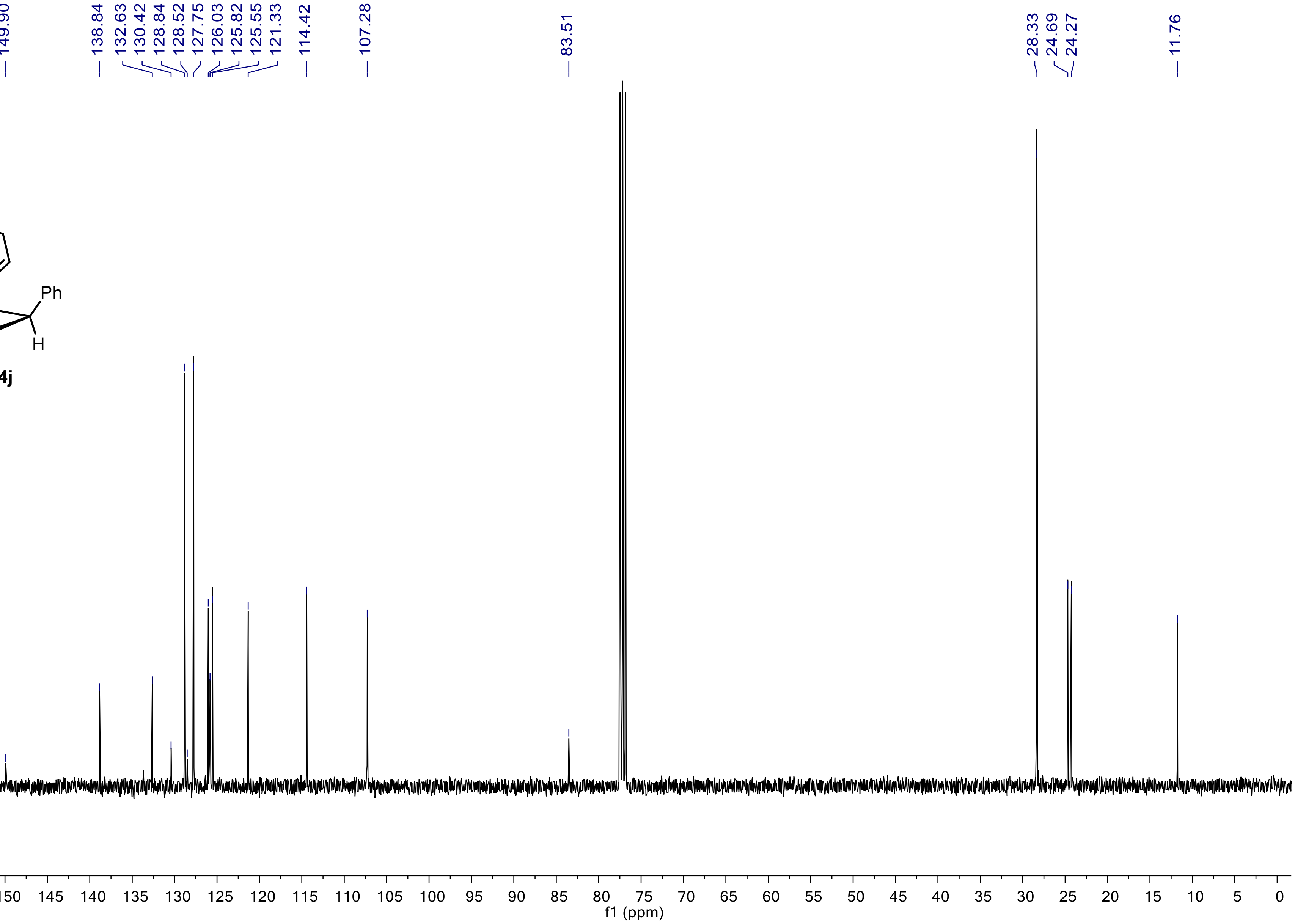

^{1}H-NMR (400 MHz, $CDCl_3$) for 4k:

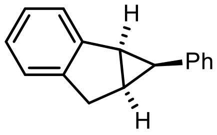

4k

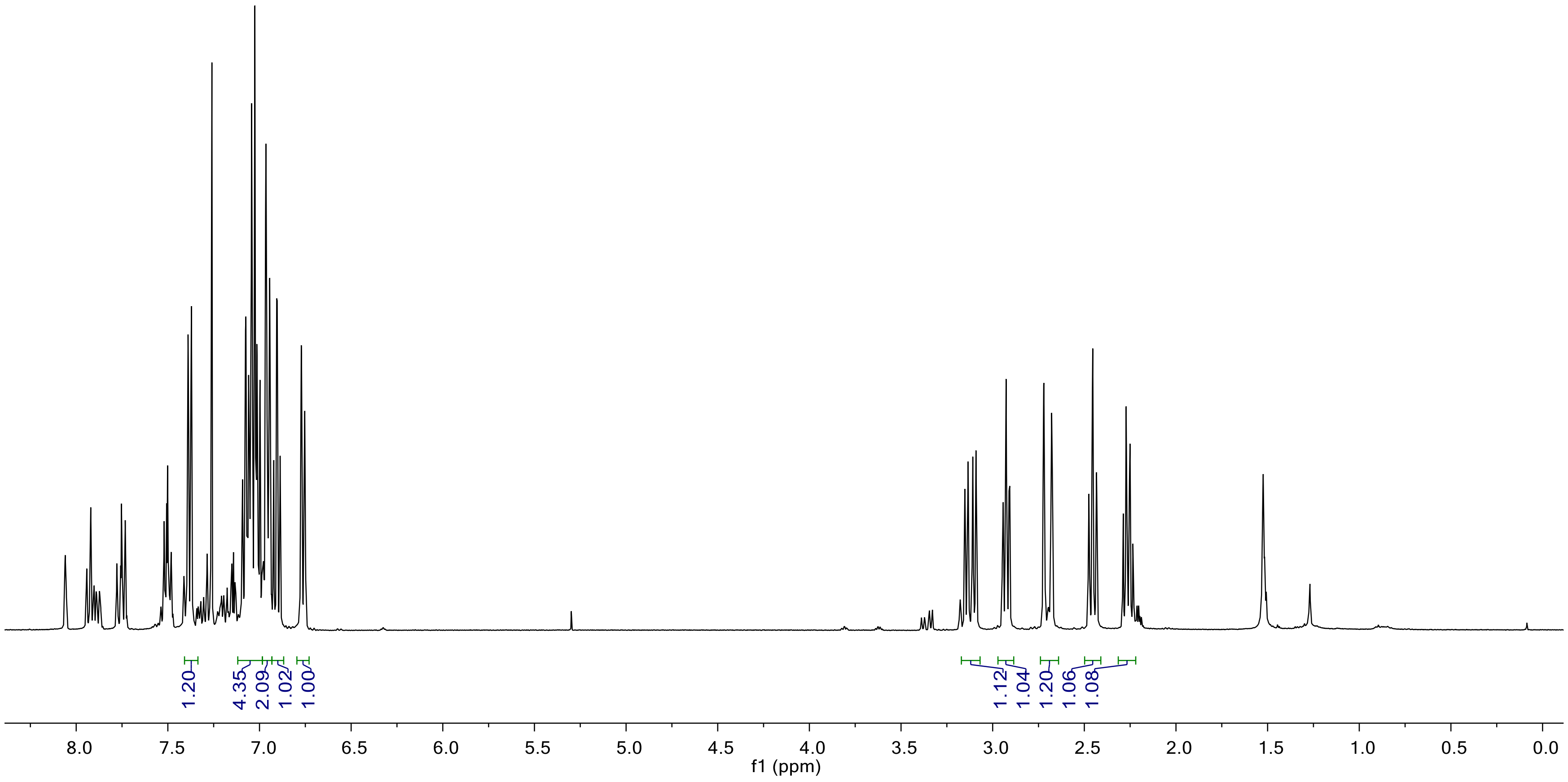

^{13}C-NMR (101 MHz, $CDCl_3$) for 4k:

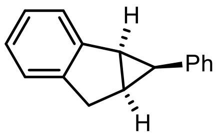

4k

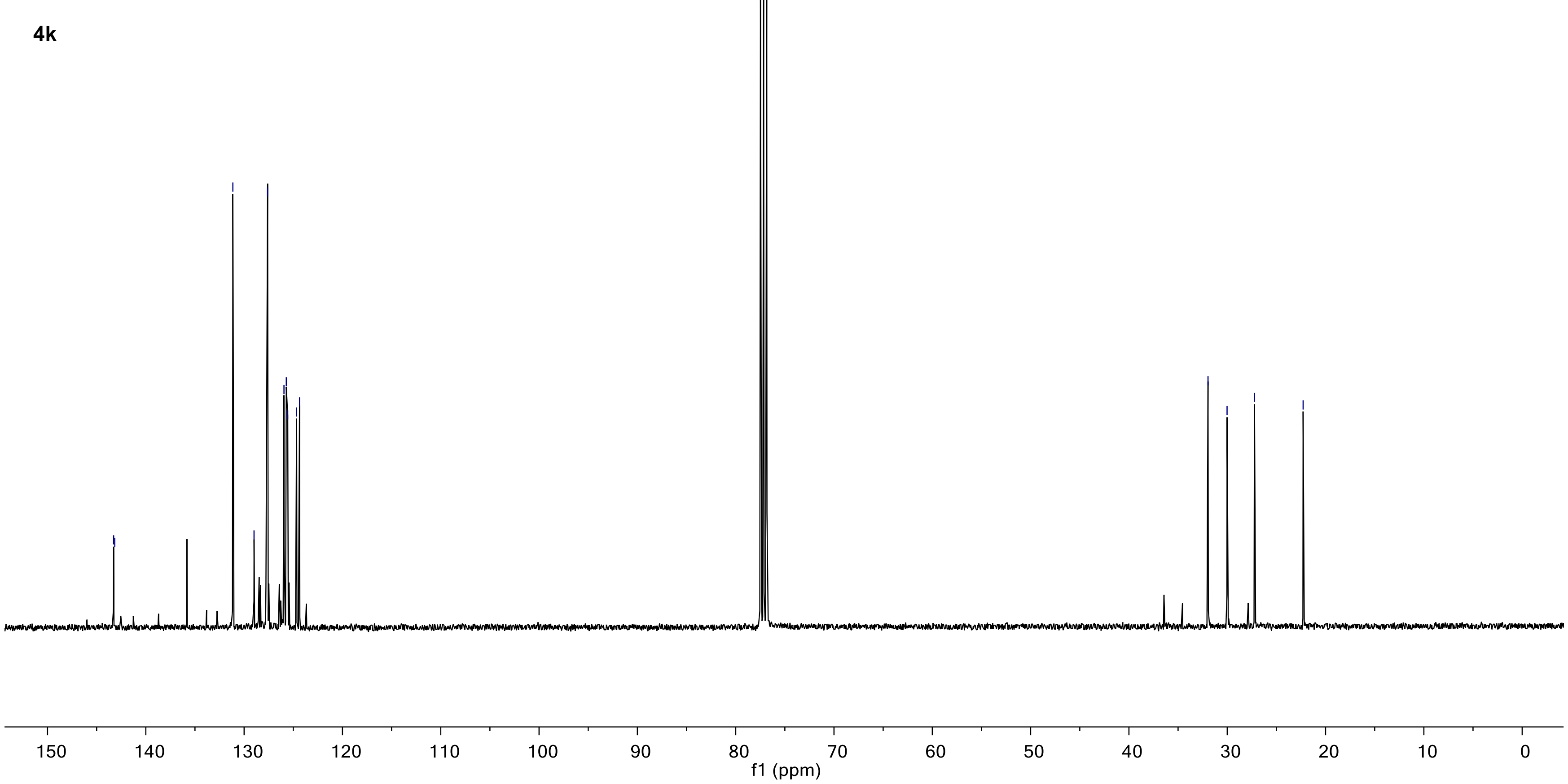

^{1}H-NMR (400 MHz, $CDCl_3$) for 4l:

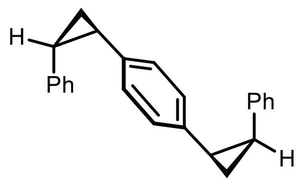

4l

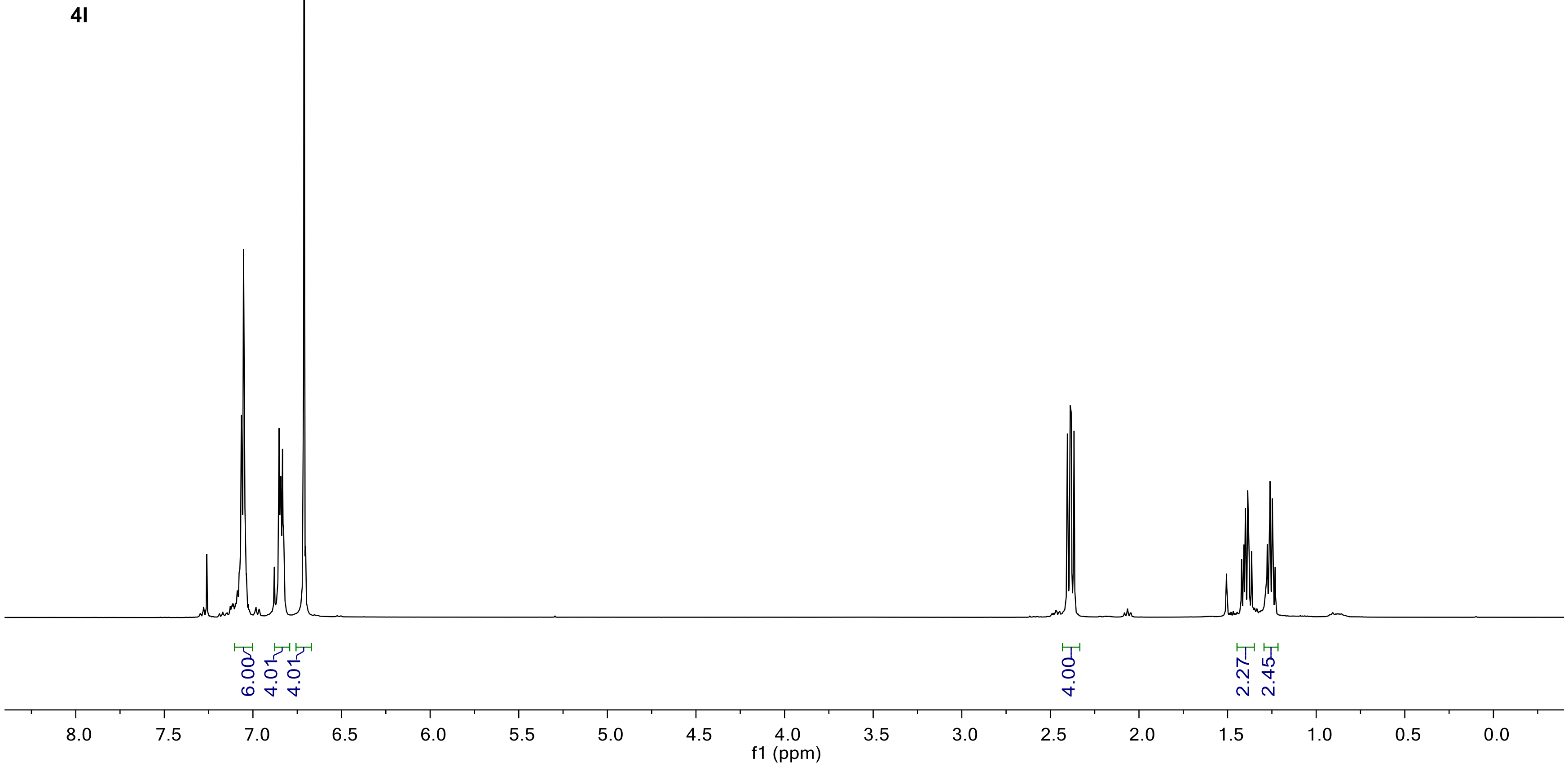

^{13}C-NMR (101 MHz, $CDCl_3$) for 4l:

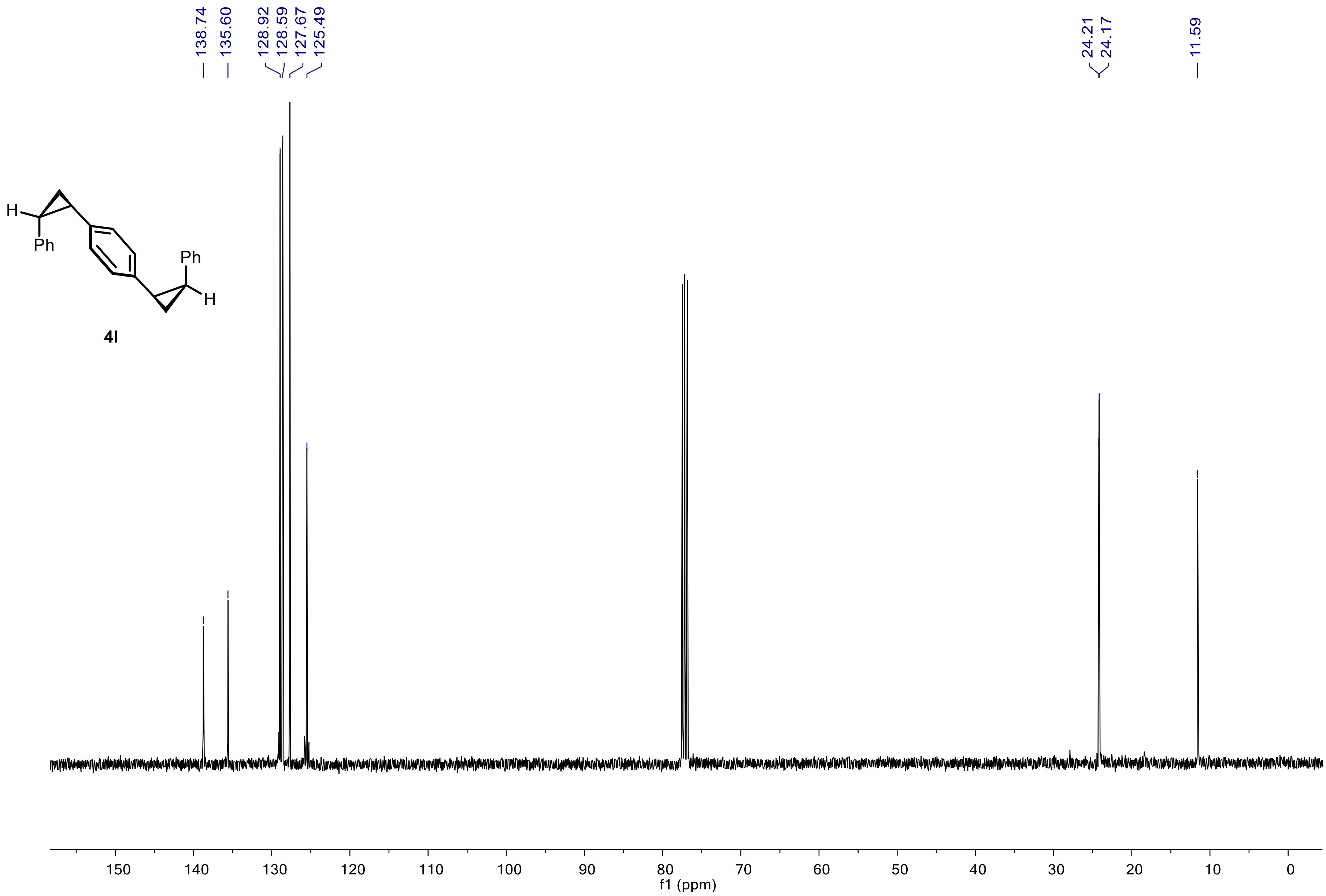

^{1}H-NMR (400 MHz, $CDCl_3$) for 4m:

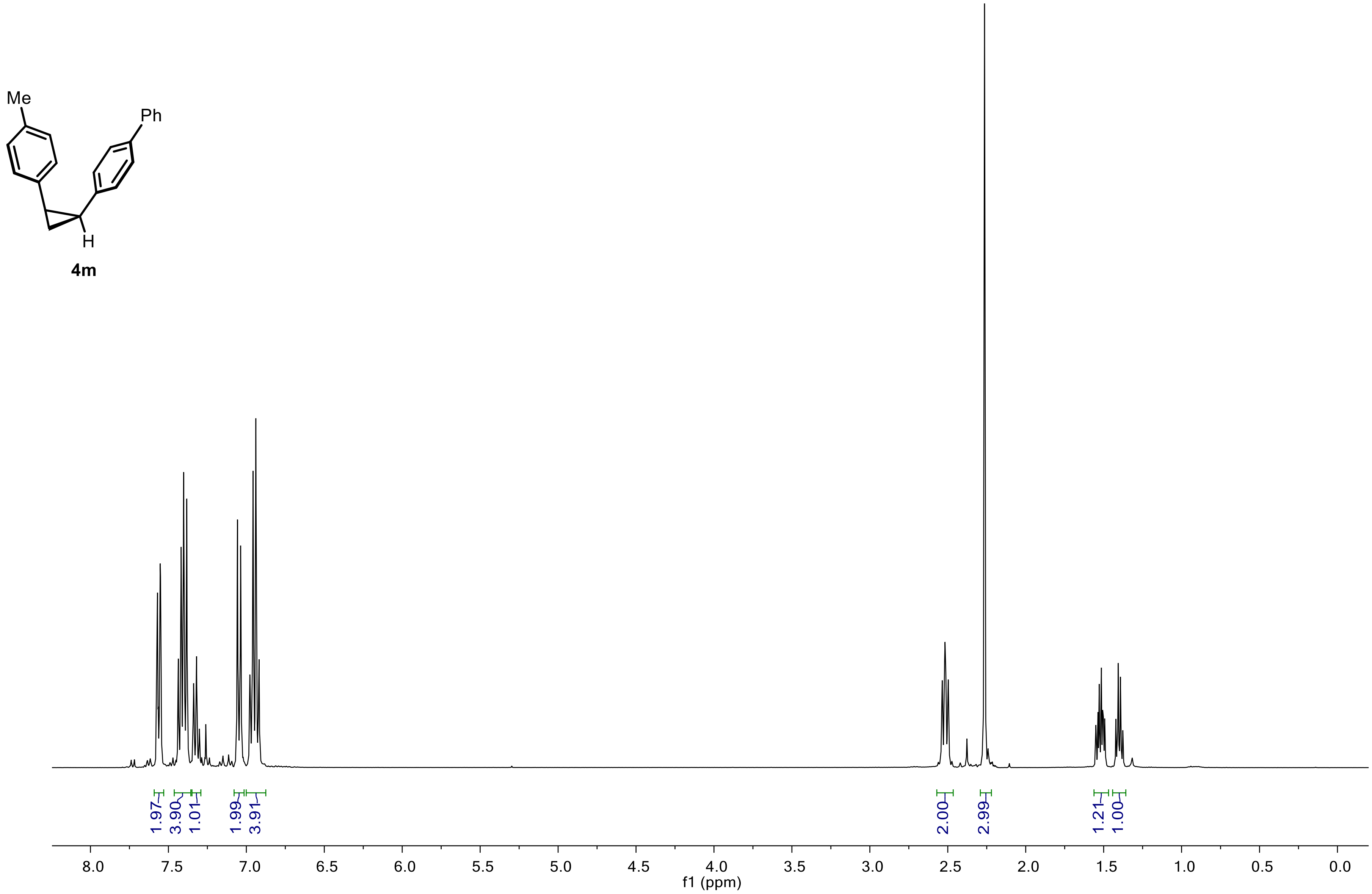

^{13}C-NMR (101 MHz, $CDCl_3$) for 4m:

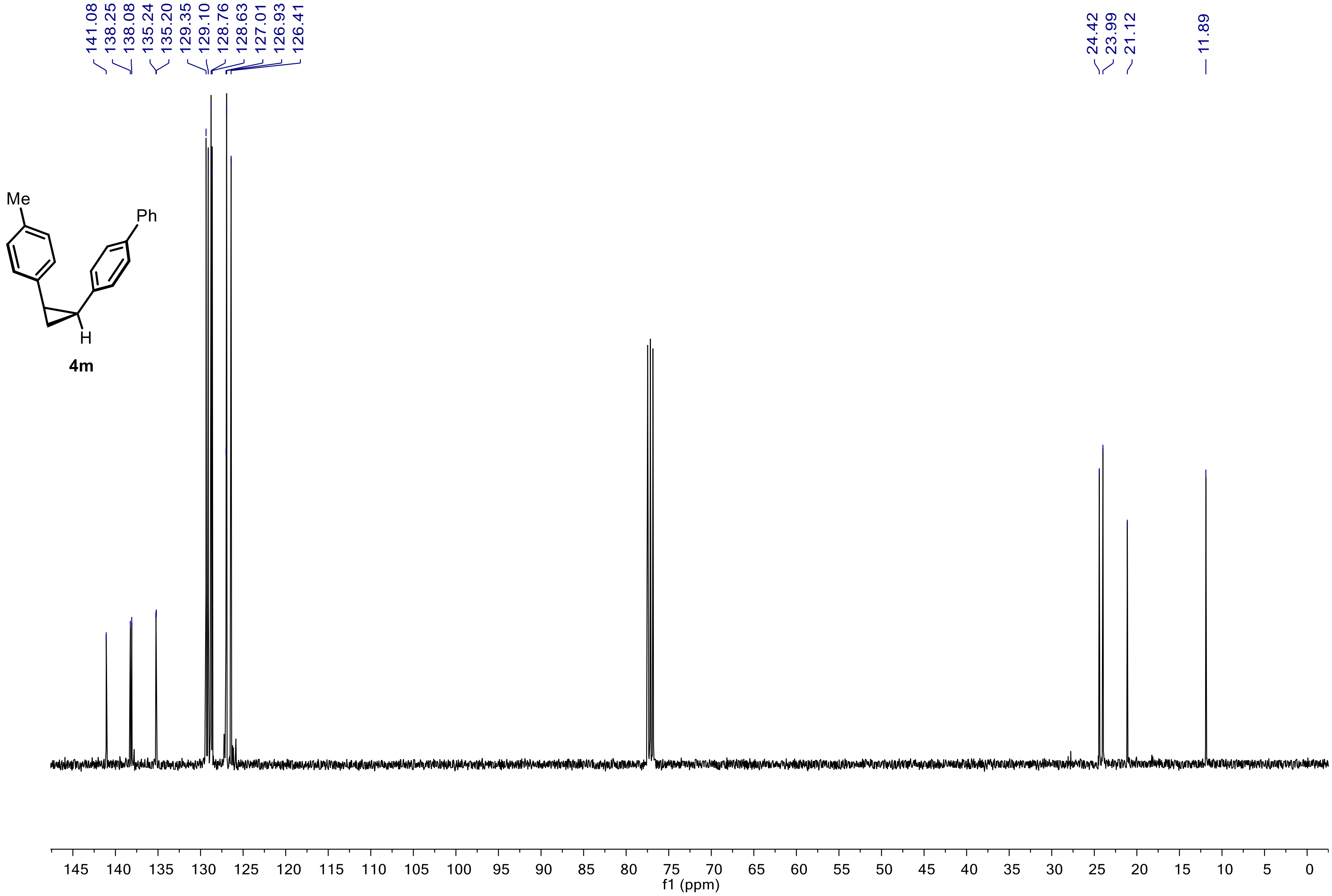

^{1}H-NMR (400 MHz, $CDCl_3$) for 4n:

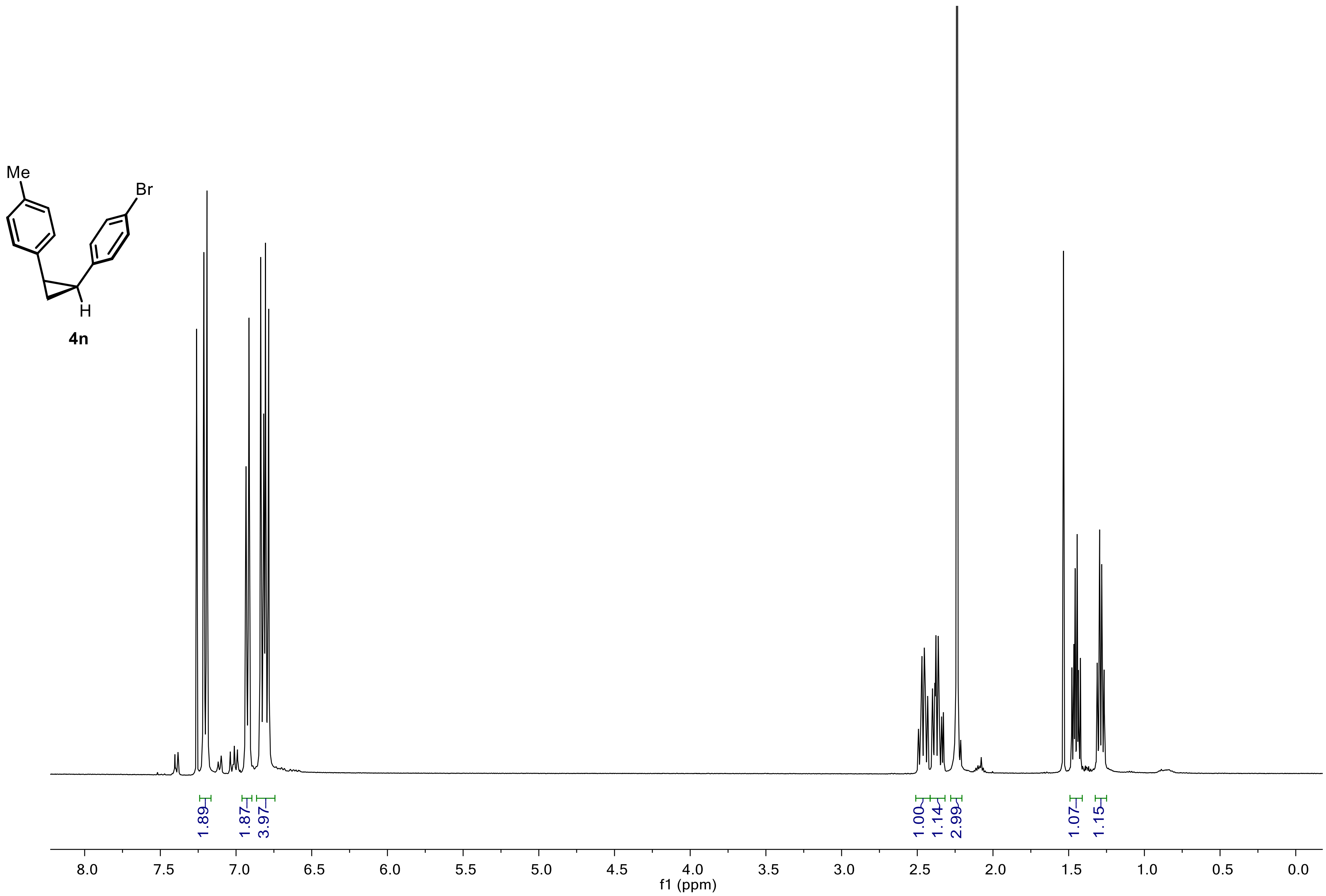

^{13}C-NMR (101 MHz, $CDCl_3$) for 4n:

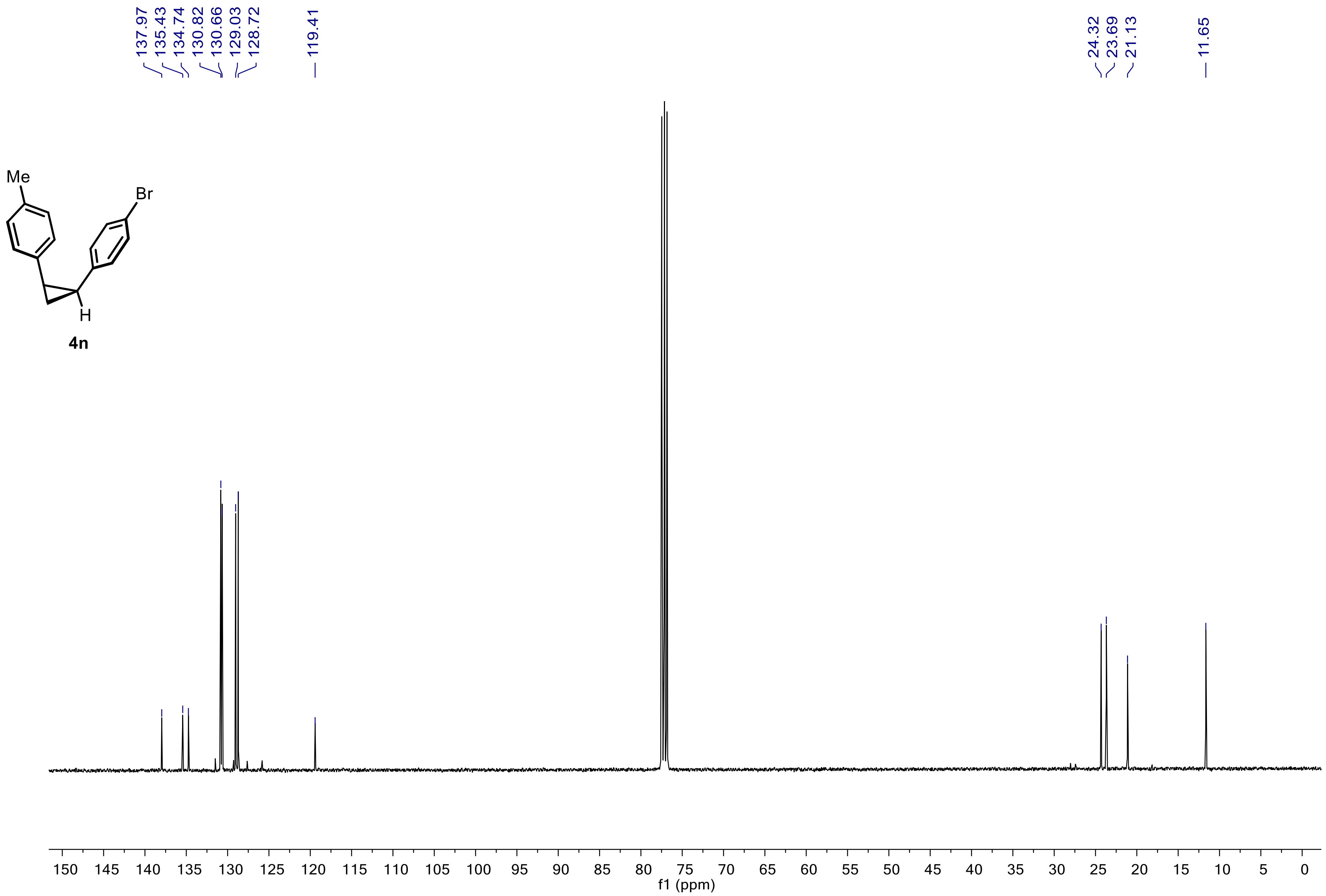

^{1}H-NMR (400 MHz, $CDCl_3$) for 4o:

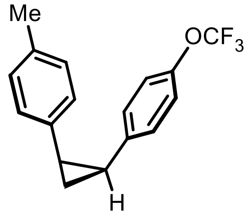

4o

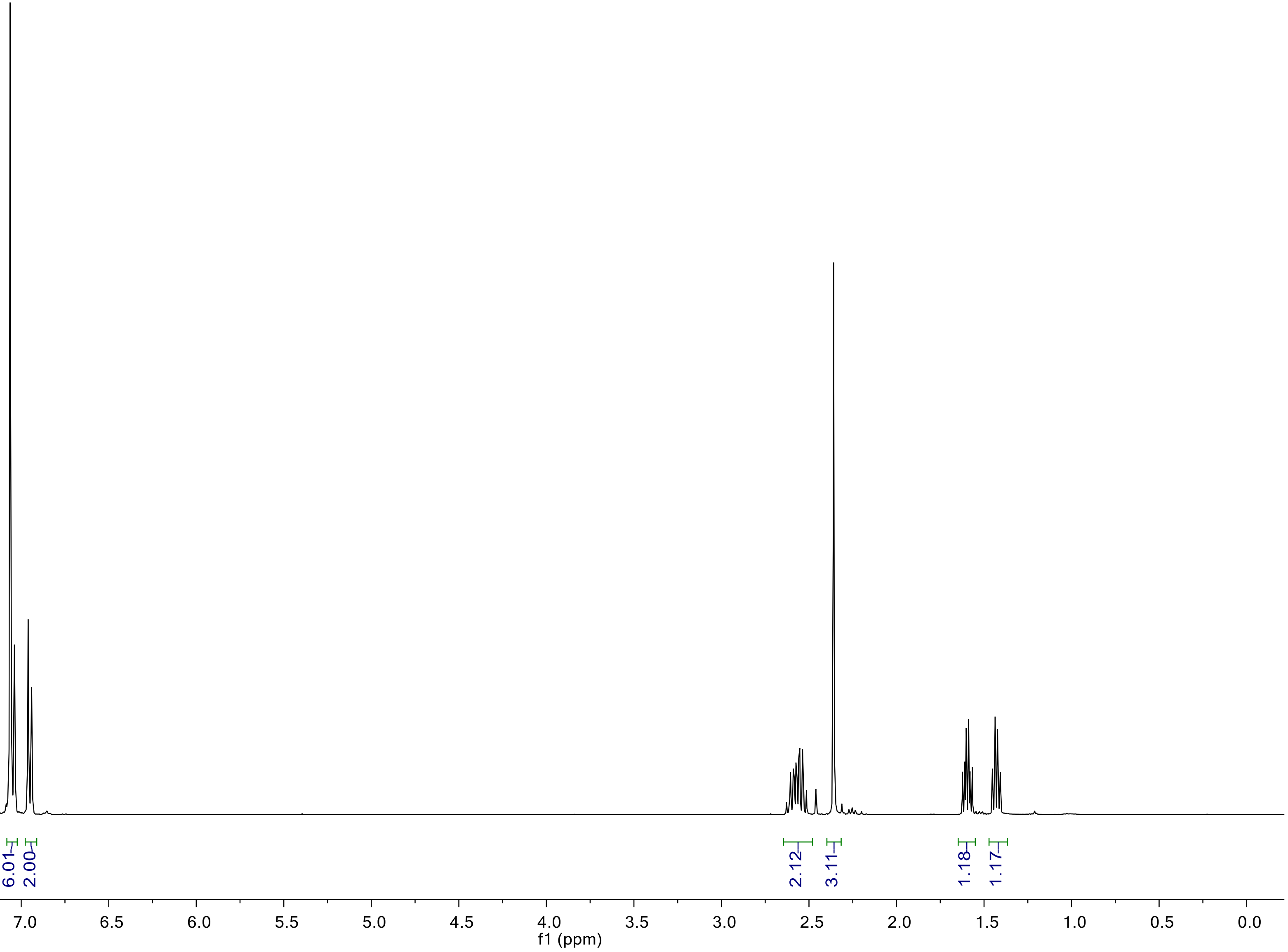

^{13}C-NMR (101 MHz, $CDCl_3$) for 4o:

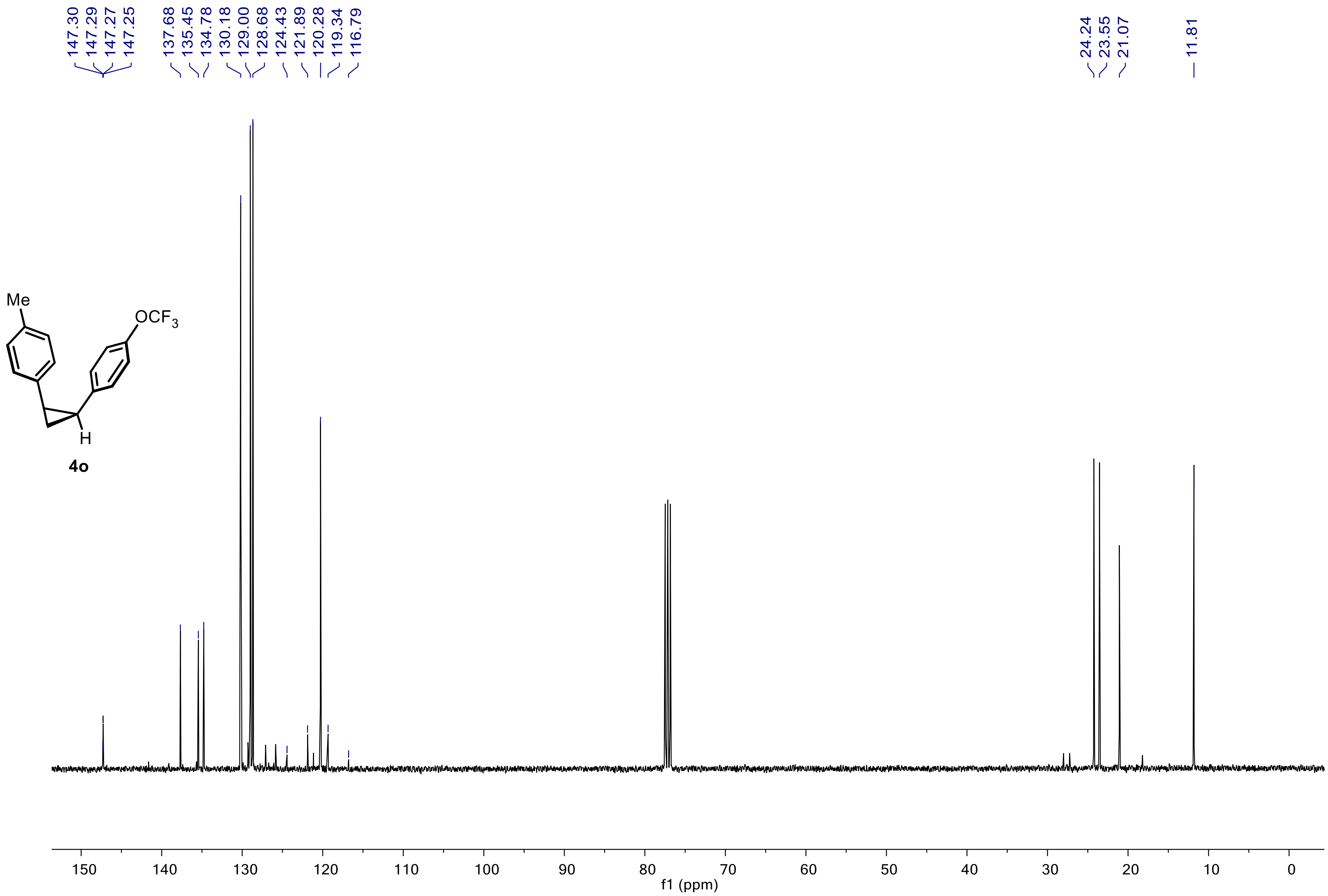

^{19}F-NMR (377 MHz, $CDCl_3$) for 4o:

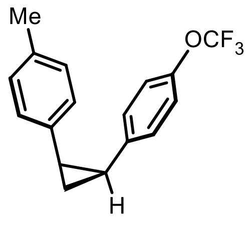

4o

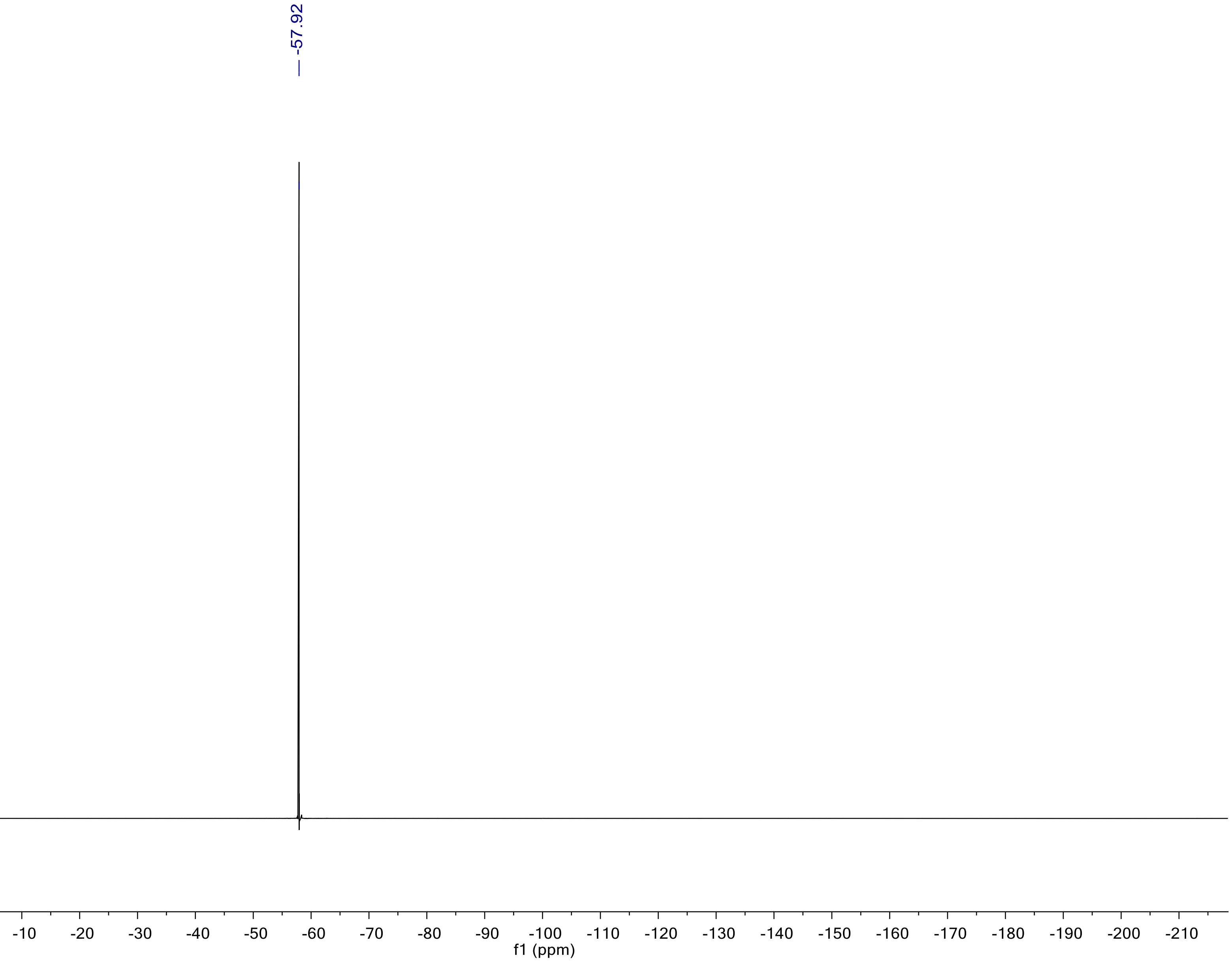

^{1}H-NMR (400 MHz, $CDCl_3$) for 4p:

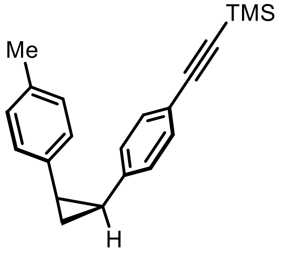

4p

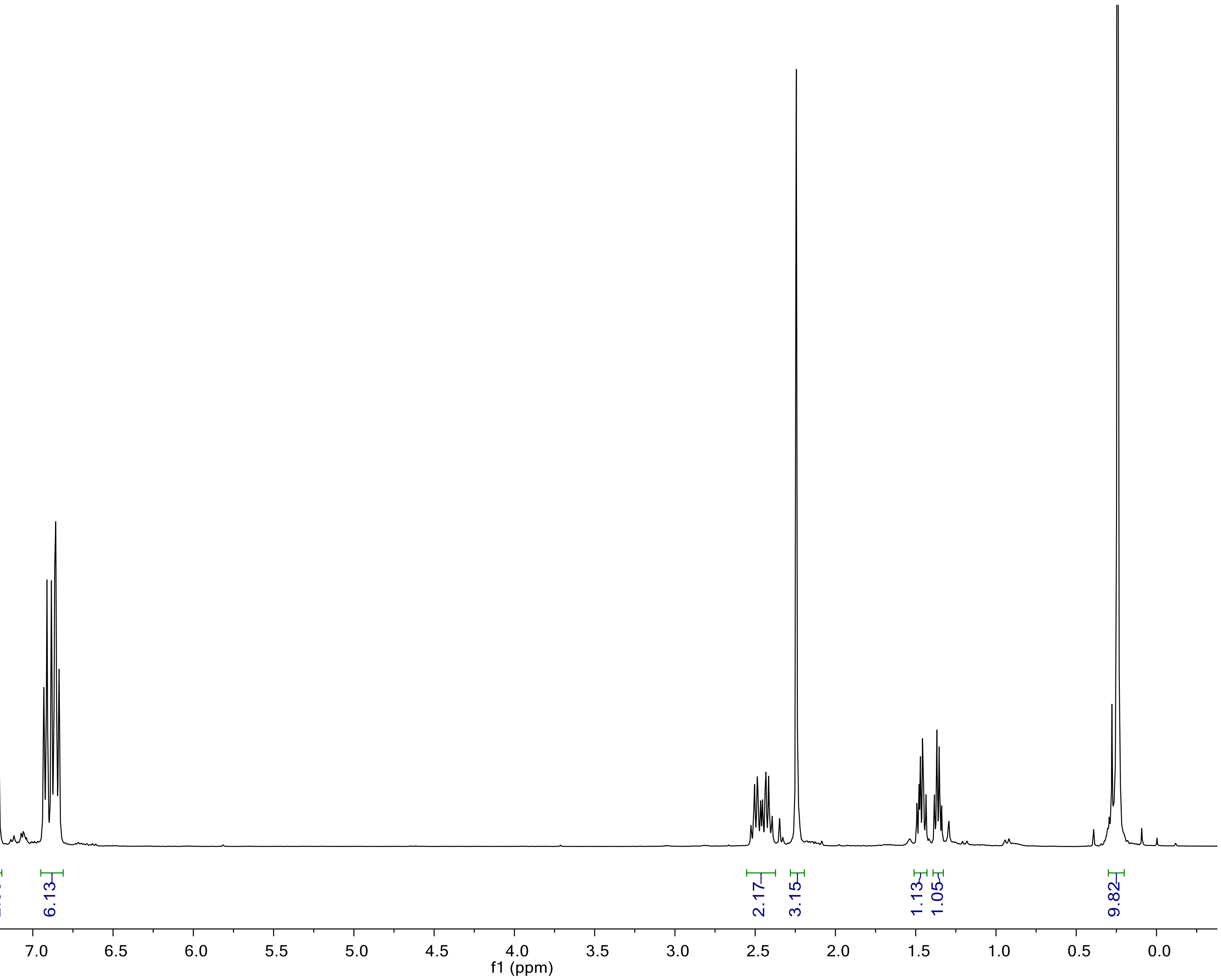

^{13}C-NMR (101 MHz, $CDCl_3$) for 4p:

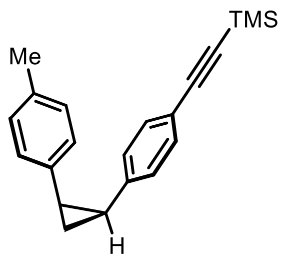

4p

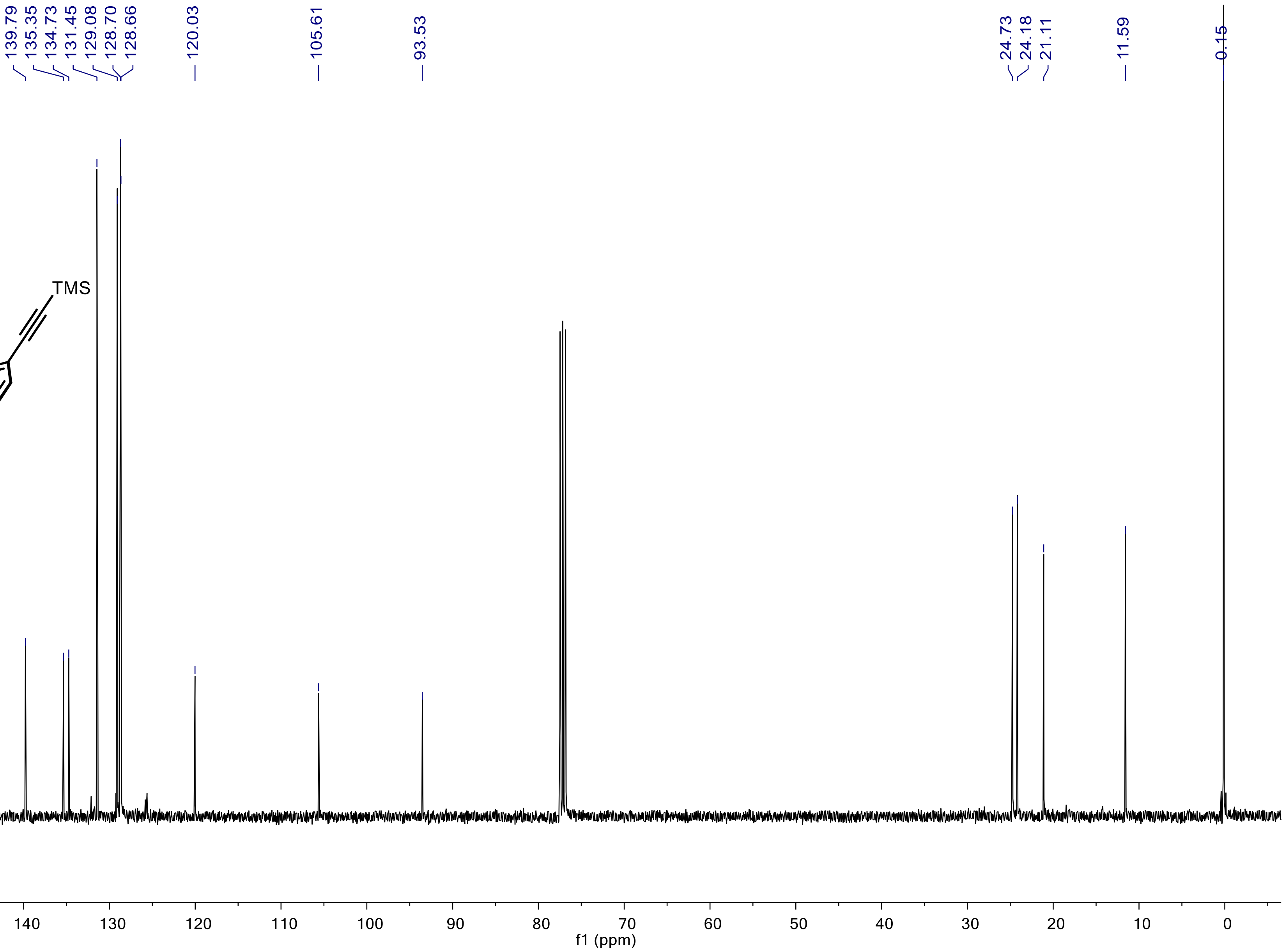

^{1}H-NMR (400 MHz, $CDCl_3$) for 4q:

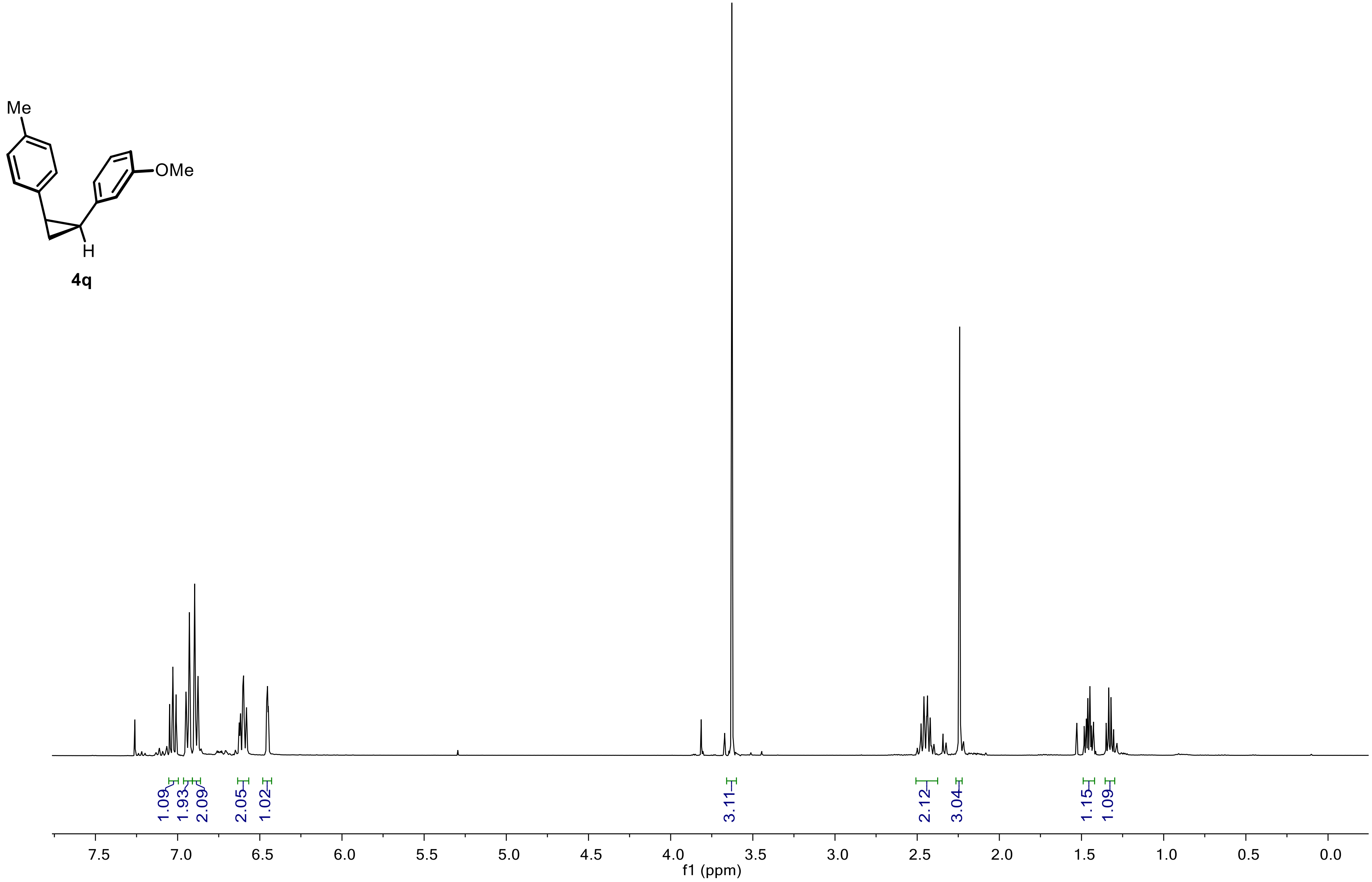

^{13}C-NMR (101 MHz, $CDCl_3$) for 4q:

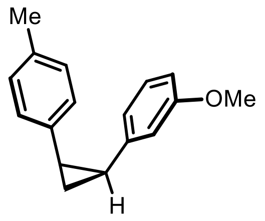

4q

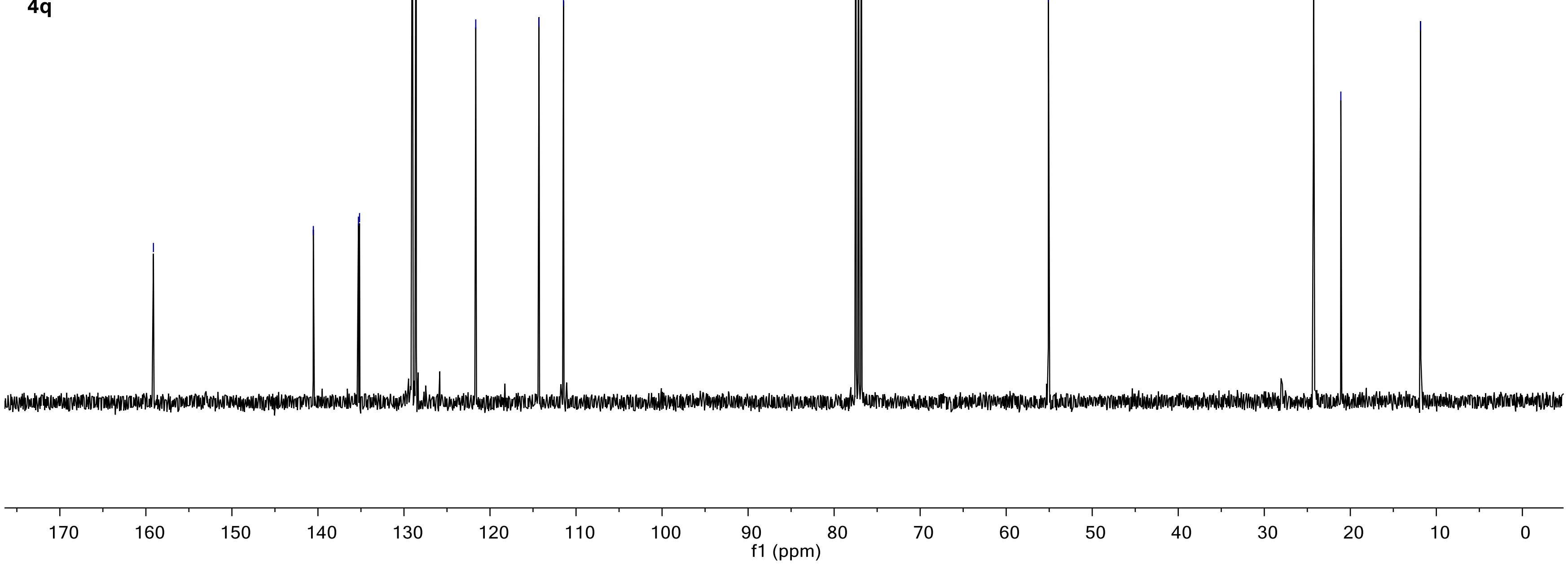

^{1}H-NMR (400 MHz, $CDCl_3$) for 4r:

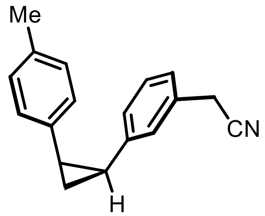

4r

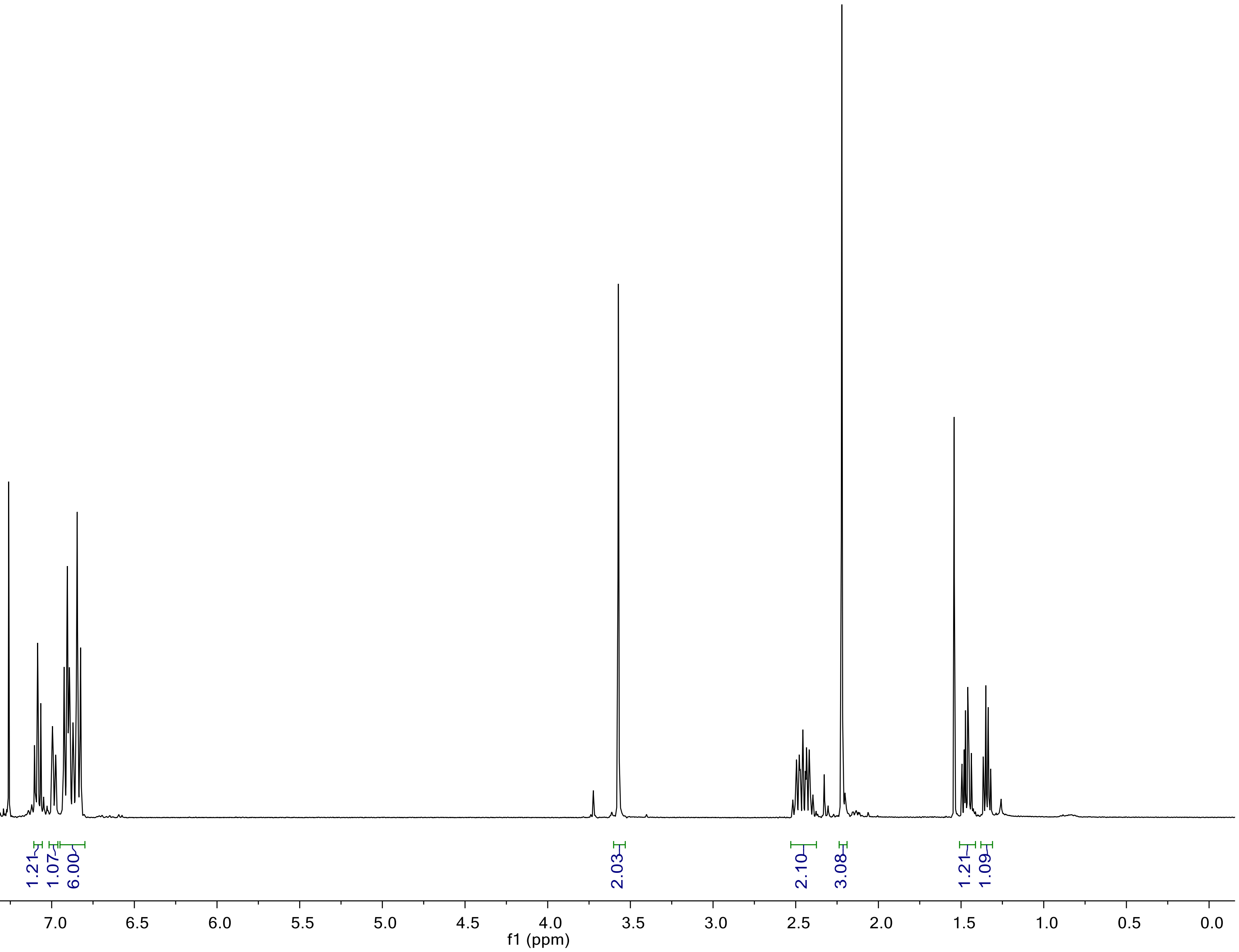

^{13}C-NMR (101 MHz, $CDCl_3$) for 4r:

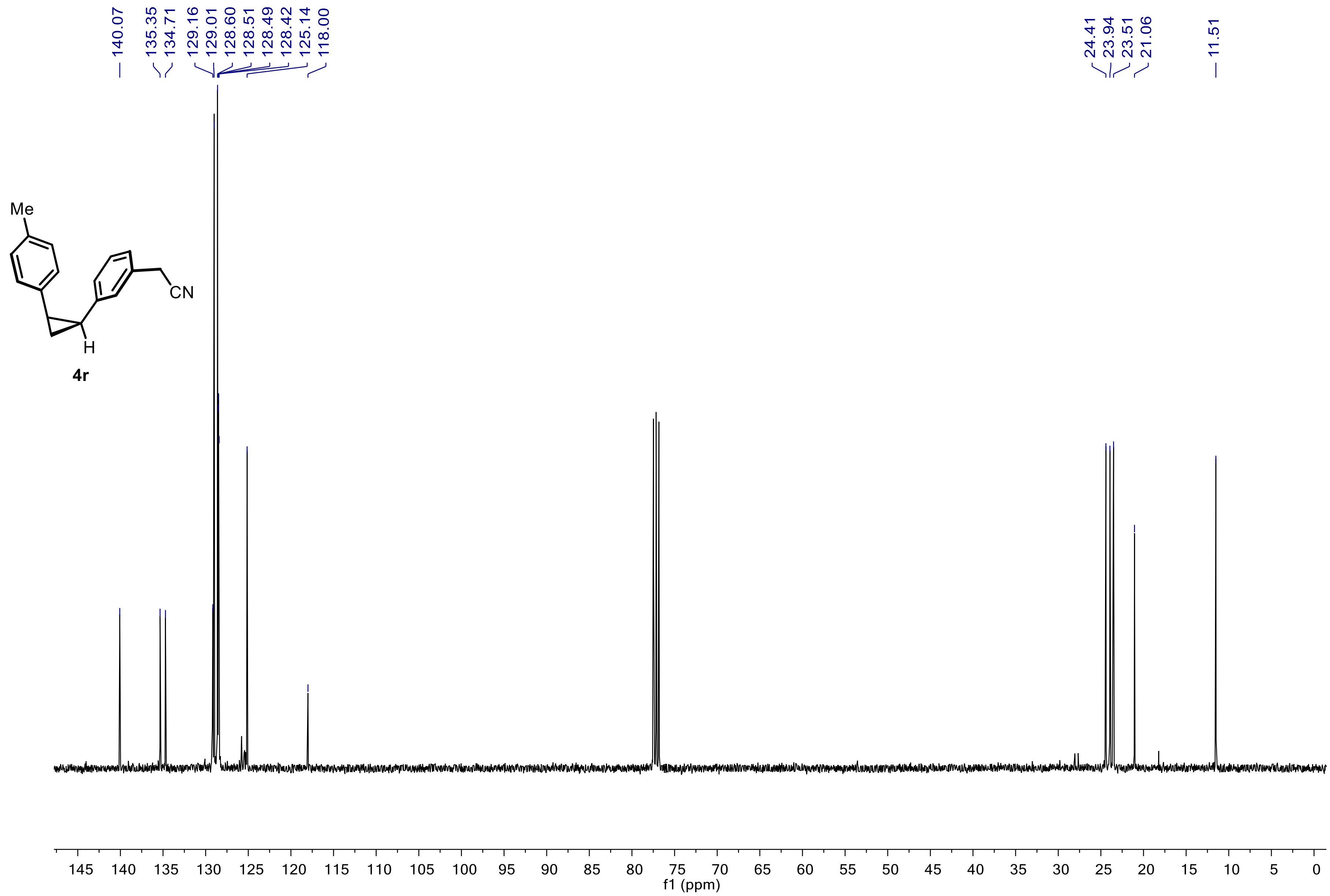

^{1}H-NMR (400 MHz, $CDCl_3$) for 4s:

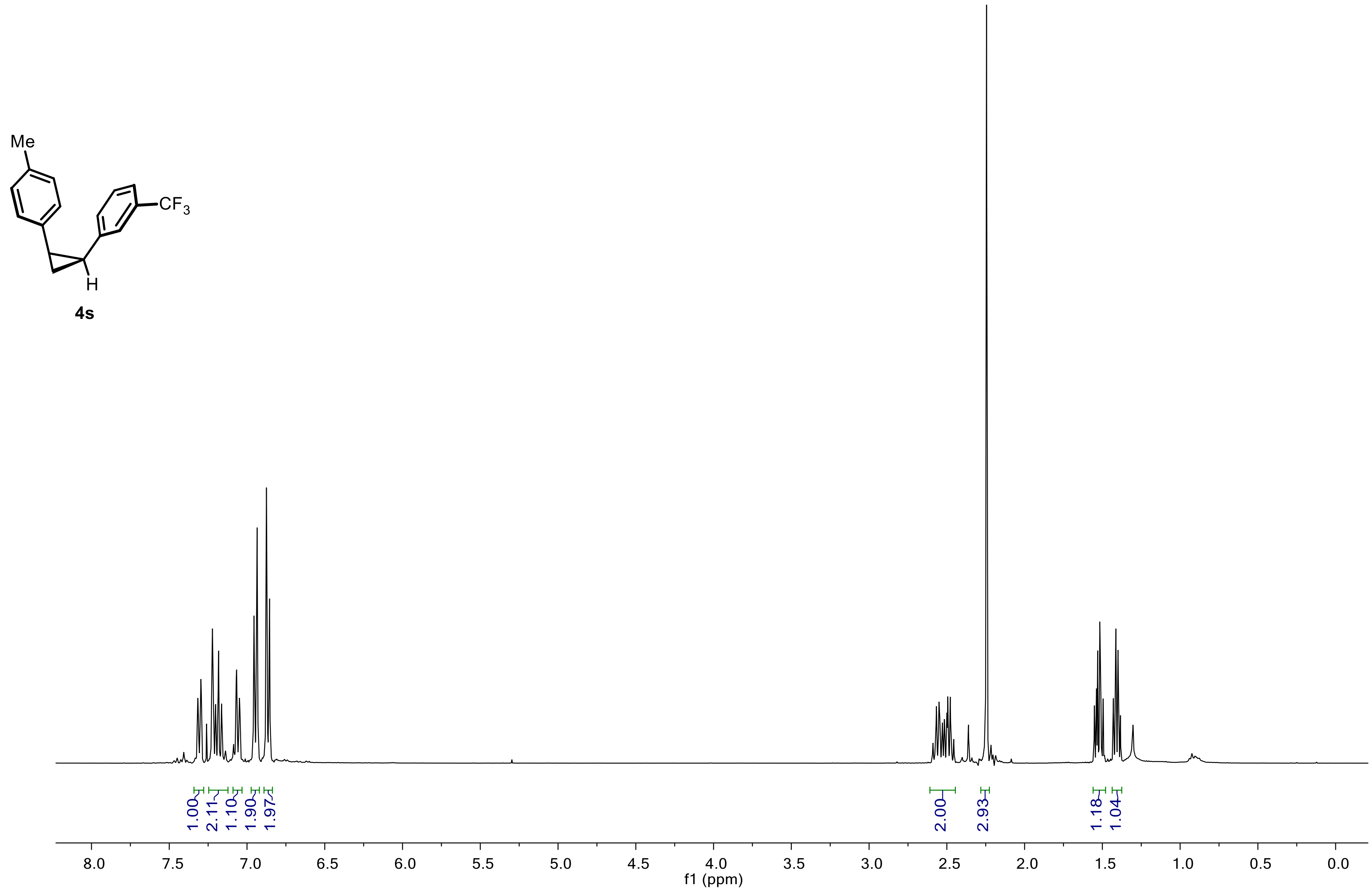

^{13}C-NMR (101 MHz, $CDCl_3$) for 4s:

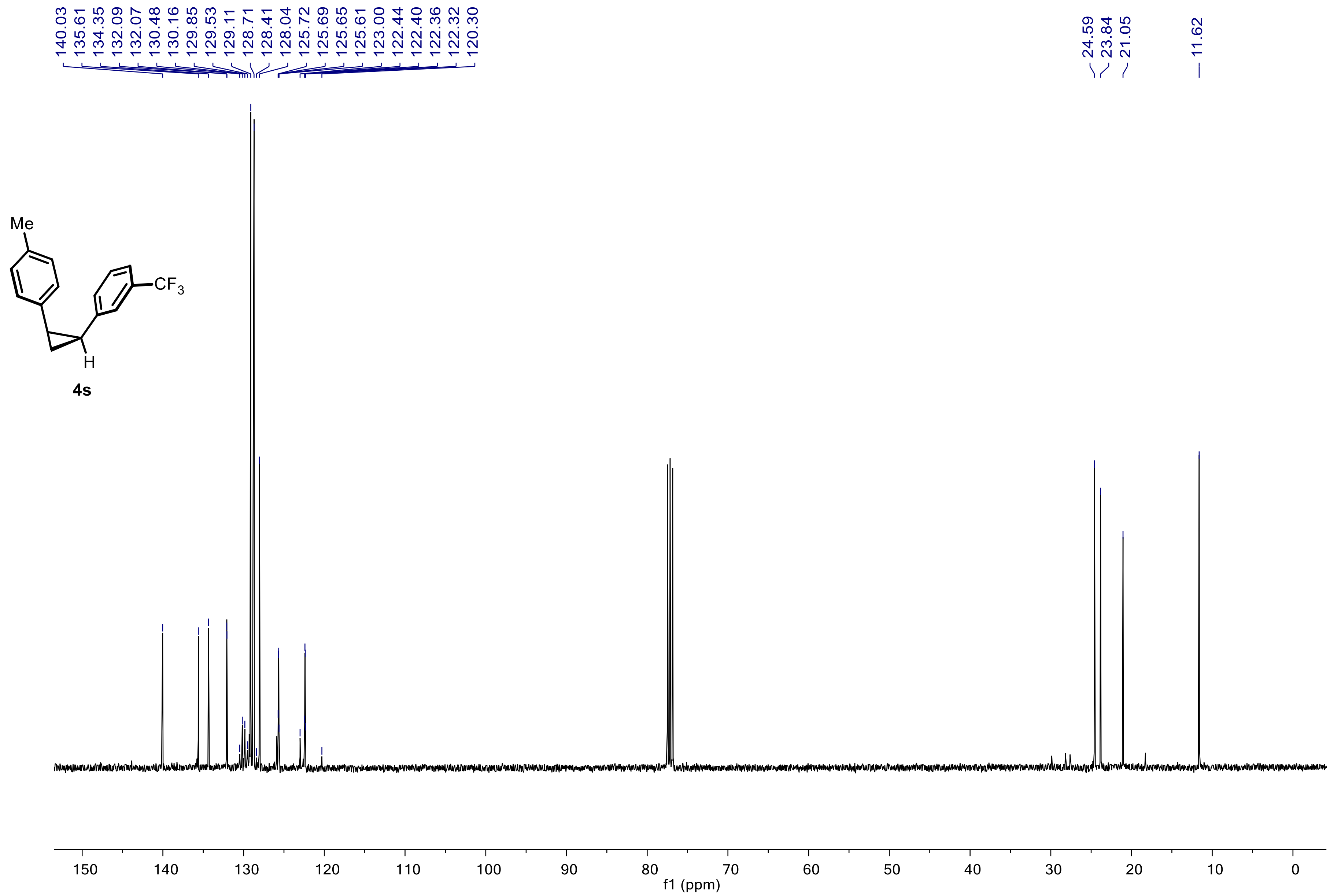

^{19}F-NMR (377 MHz, $CDCl_3$) for 4s:

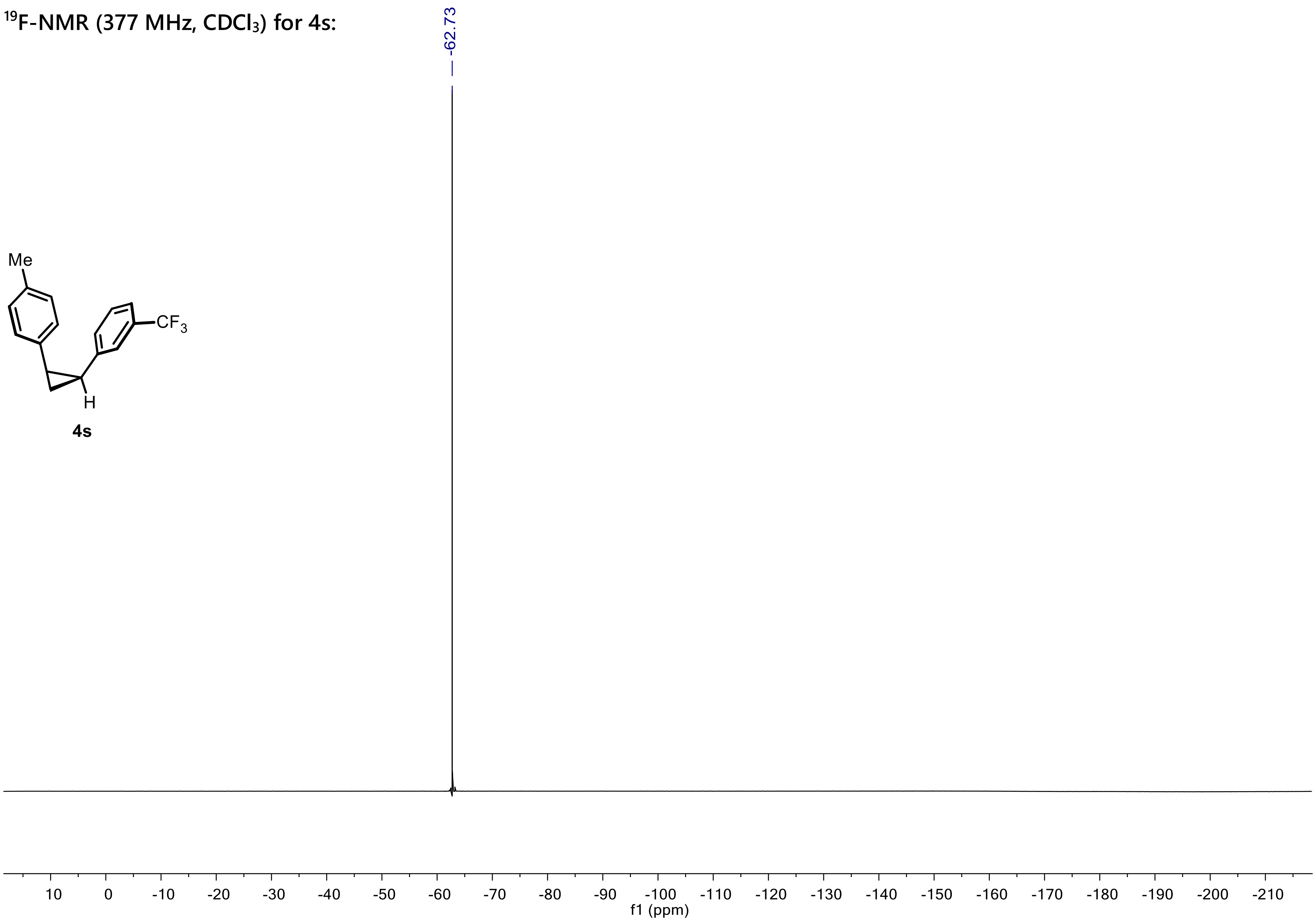

^{1}H-NMR (400 MHz, $CDCl_3$) for 4t:

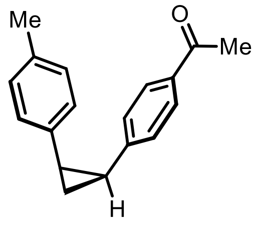

4t

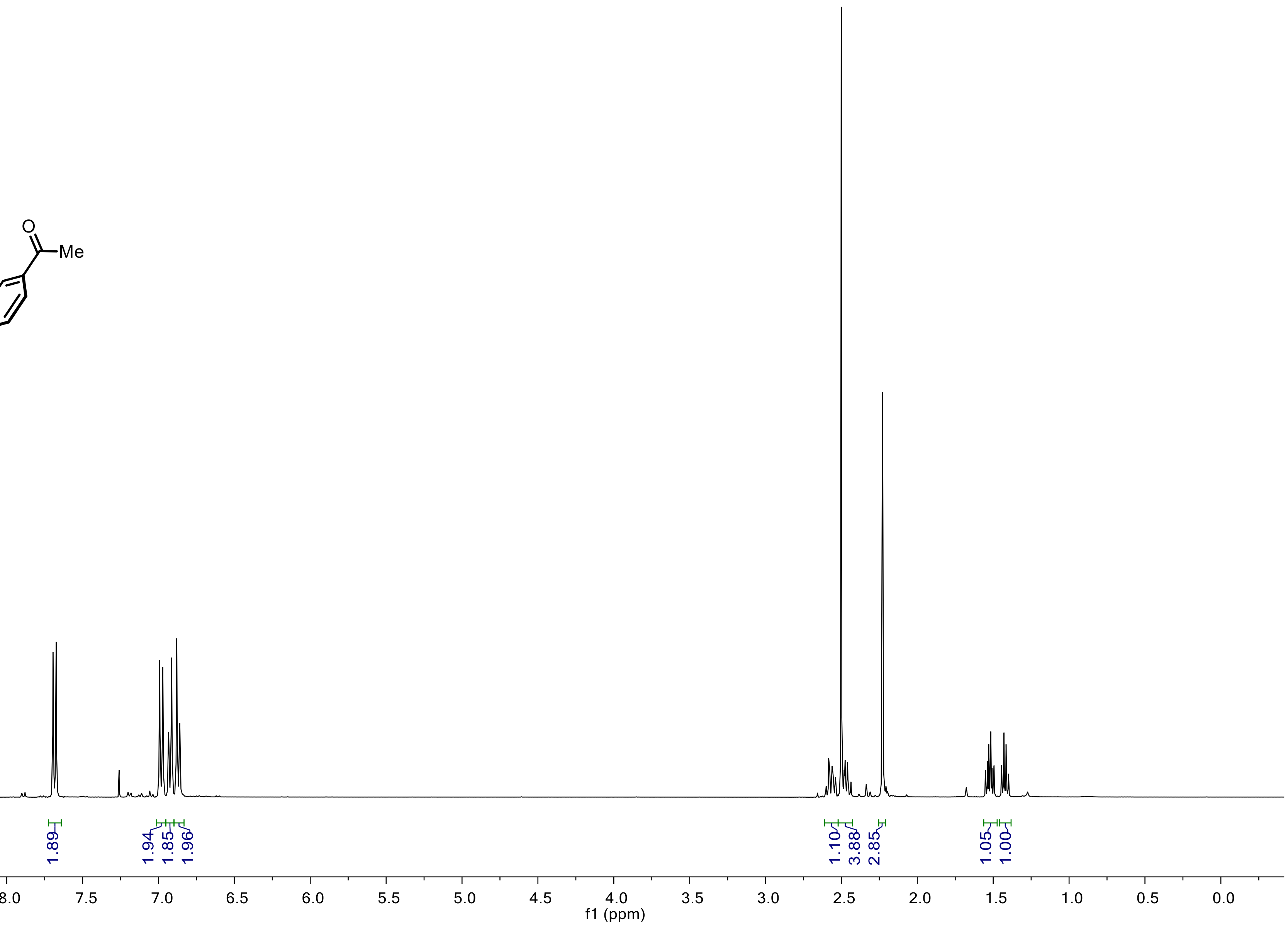

^{13}C-NMR (101 MHz, $CDCl_3$) for 4t:

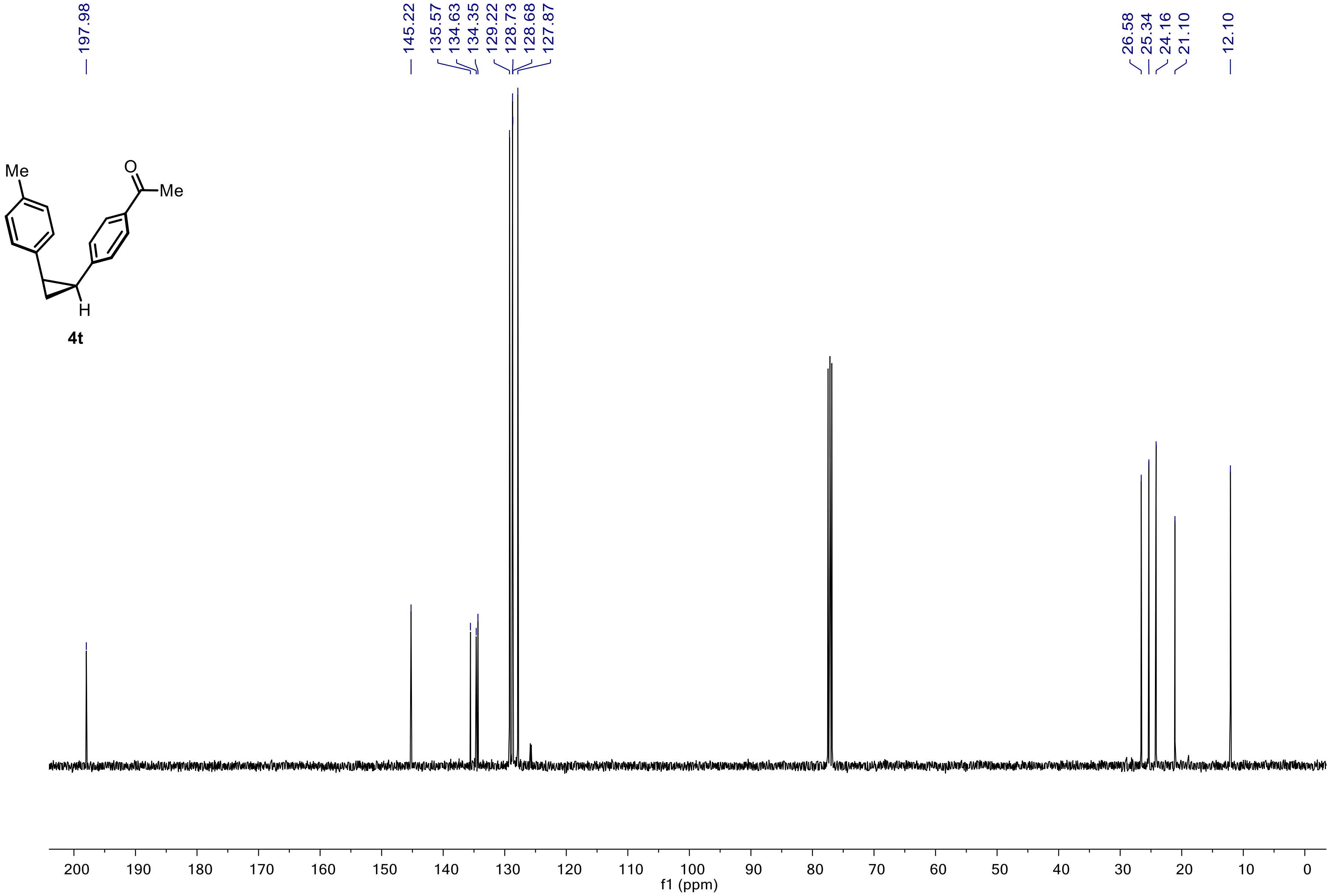

^{1}H-NMR (400 MHz, $CDCl_3$) for 4u:

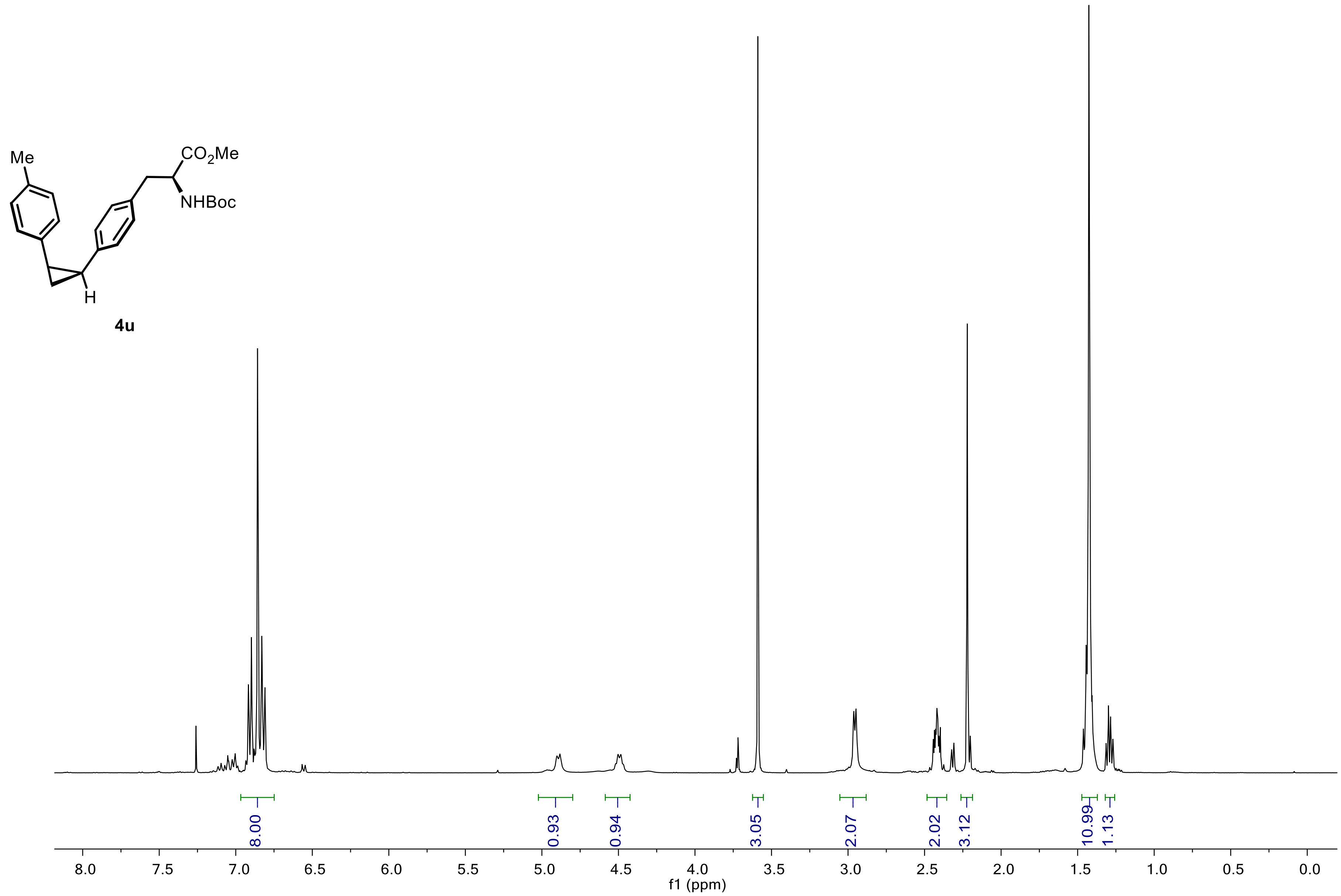

^{13}C-NMR (101 MHz, $CDCl_3$) for 4u:

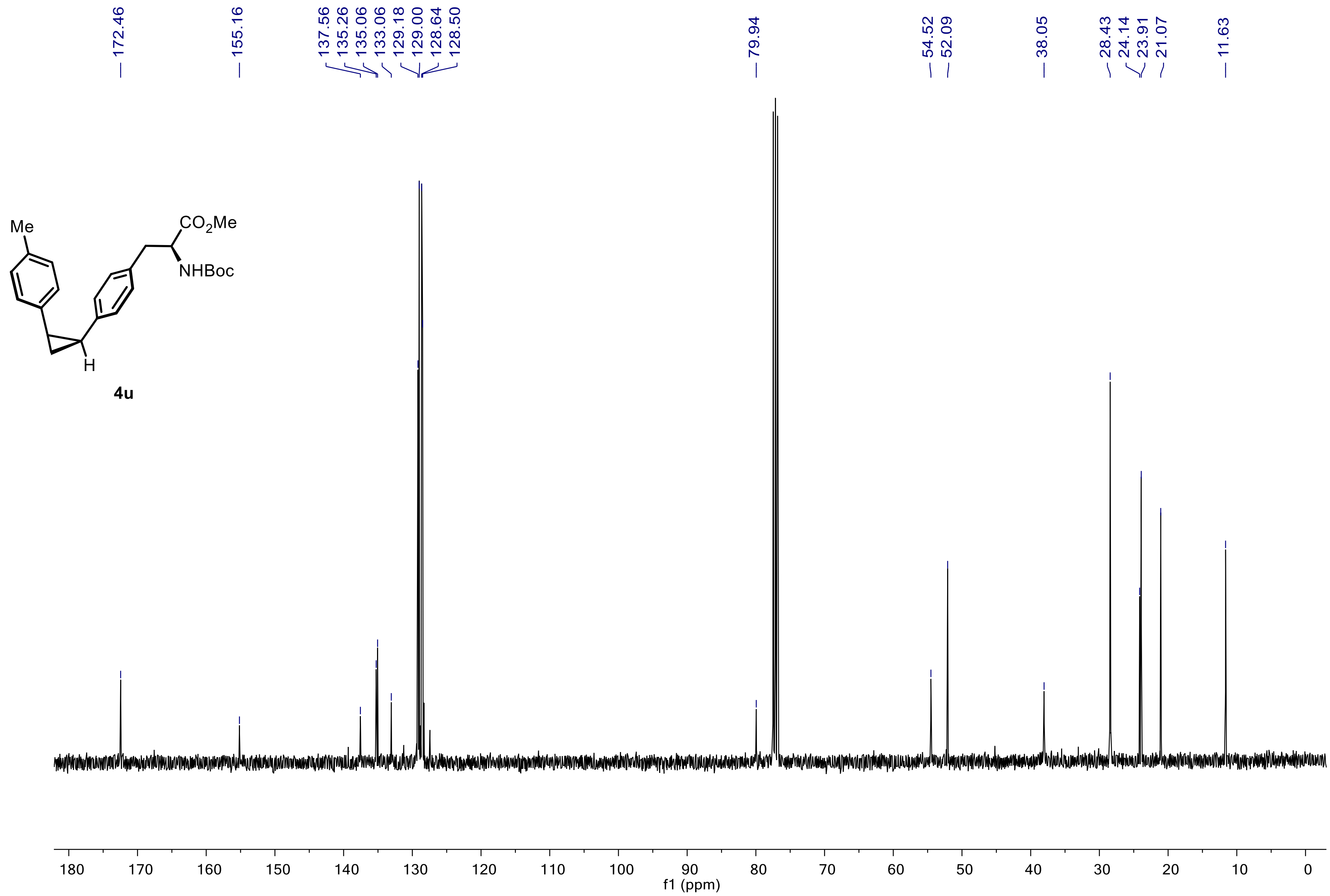

^{1}H-NMR (400 MHz, $CDCl_3$) for 4v:

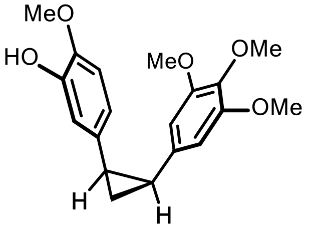

4v

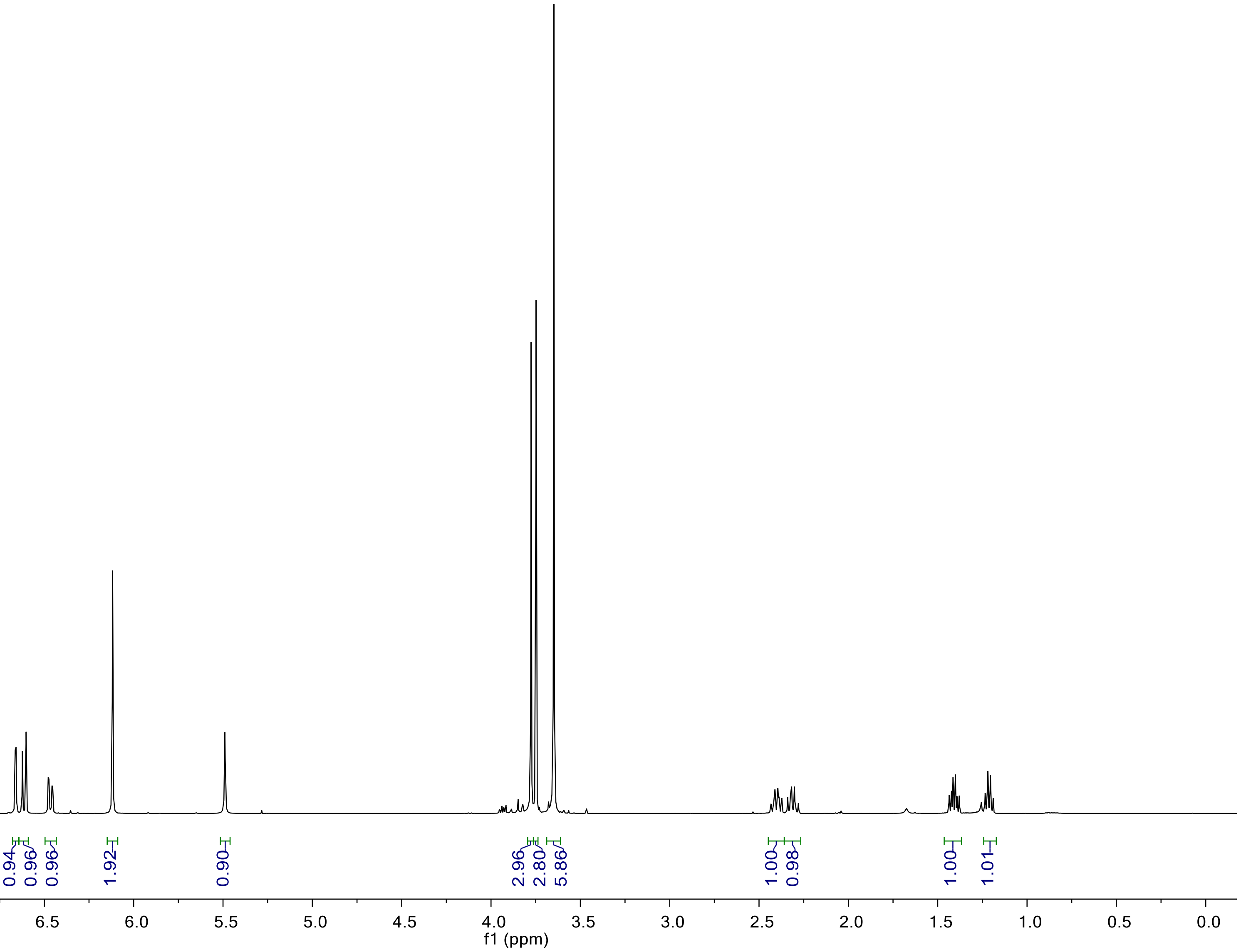

^{13}C-NMR (101 MHz, CDCl$_3$) for 4v:

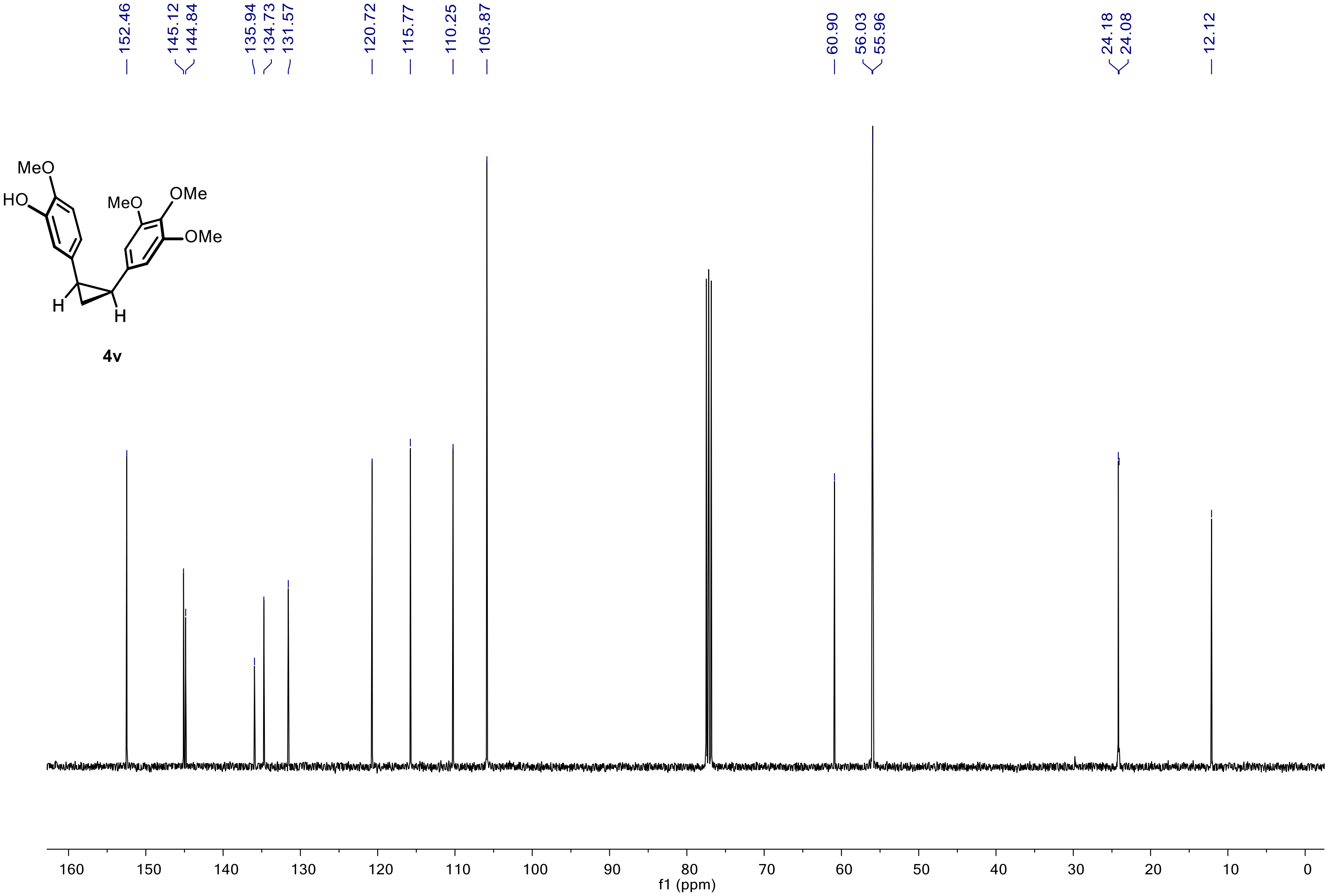

Chromatography data for enantioenriched compounds

SFC trace for 4b:

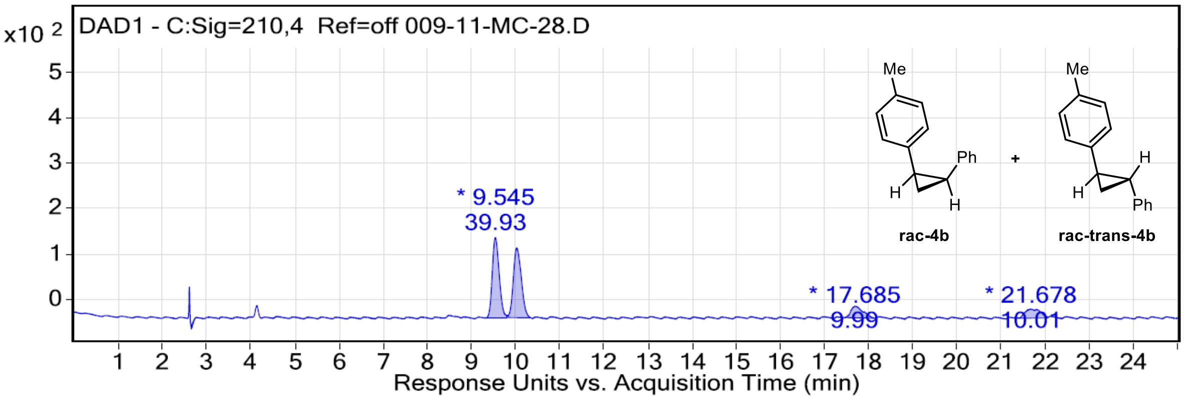

Integration Peak List

Peak	Start	RT	End	Height	Area	AreaSumPercent
1	9.342	9.545	9.803	177.08	1935.19	39.93
2	9.835	10.026	10.342	153.71	1942.18	40.07
3	17.468	17.685	18.138	25.49	484.09	9.99
4	21.399	21.678	22.054	19.97	485.03	10.01

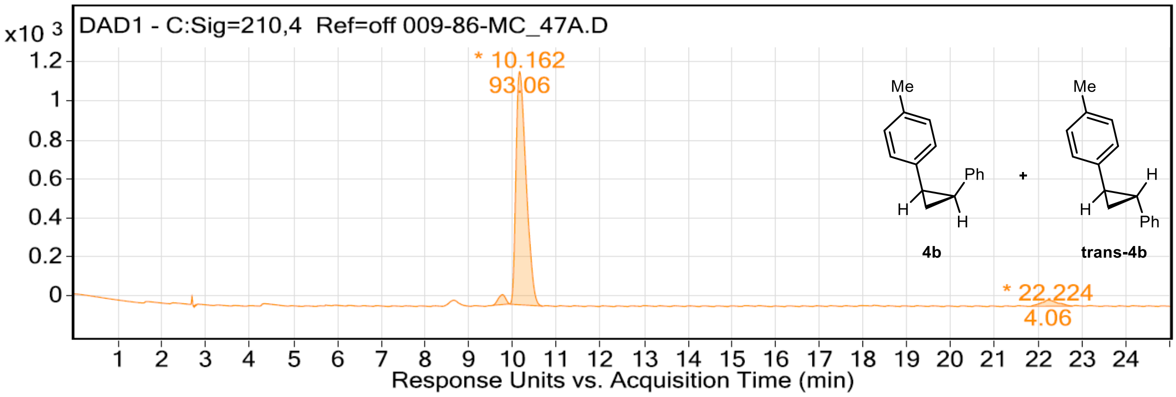

Integration Peak List

Peak	Start	RT	End	Height	Area	AreaSumPercent
1	9.551	9.766	9.935	51.05	596.67	2.88
2	9.948	10.162	10.682	1197.1	19286.28	93.06
3	21.835	22.224	22.779	31.61	842.04	4.06

Analysis Conditions: Daicel Chiralpak-IF column (0.46 cm internal diameter x 25cm), temperature 25°C, eluent MeOH 2%, flow rate 1 mL/min, λ: 210 nm.

SFC trace for 4c:

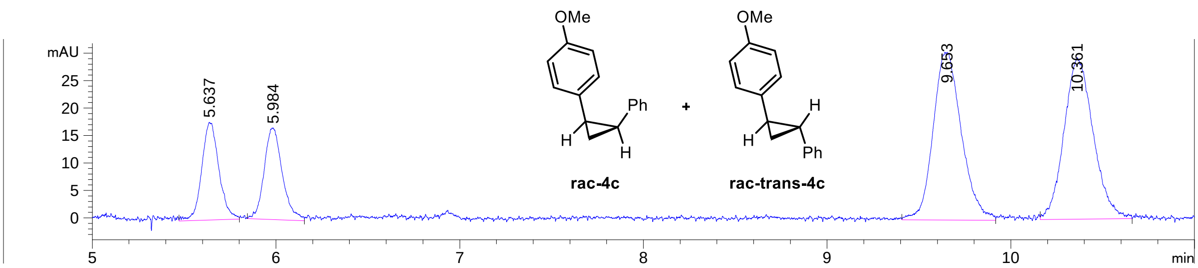

Signal 2: DAD1 B, Sig=230,4 Ref=off

Peak #	RetTime [min]	Type	Width [min]	Area [mAU*s]	Height [mAU]	Area %
1	5.637	VV	0.0786	115.98796	17.79135	13.1514
2	5.984	BV	0.0841	114.96969	16.64931	13.0359
3	9.653	VV	0.1269	327.17523	30.56682	37.0971
4	10.361	VB	0.1304	323.81100	29.42137	36.7156

Totals : 881.94389 94.42884

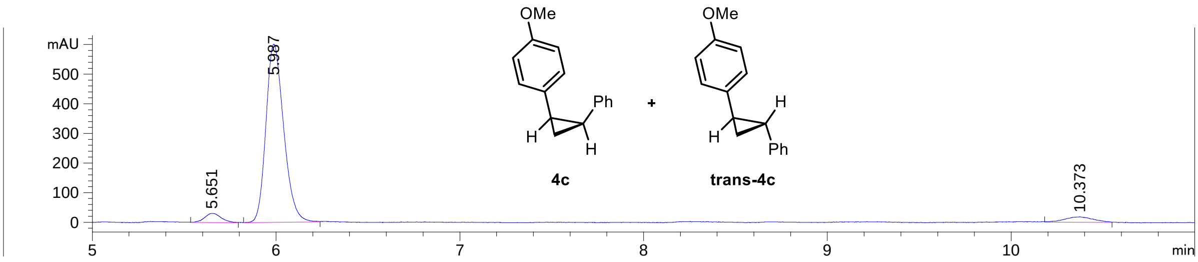

Signal 3: DAD1 C, Sig=210,4 Ref=off

Peak #	RetTime [min]	Type	Width [min]	Area [mAU*s]	Height [mAU]	Area %
1	5.651	BV	0.0744	190.85710	30.75790	4.3094
2	5.987	BV	0.1019	4041.50977	601.78796	91.2548
3	10.373	VV	0.1274	196.45016	18.27879	4.4357

Totals : 4428.81703 650.82465

Analysis Conditions: Daicel Chiralpak-IF column (0.46 cm internal diameter x 25cm), temperature 25°C, eluent MeOH 10%, flow rate 1 mL/min, λ: 210 nm.

HPLC trace for 4d:

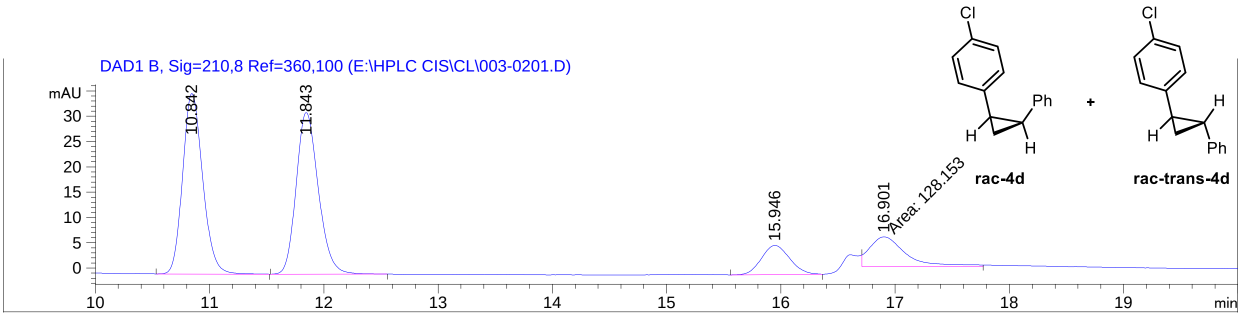

Signal 2: DAD1 B, Sig=210,8 Ref=360,100

Peak #	RetTime [min]	Type	Width [min]	Area [mAU*s]	Height [mAU]	Area %
1	10.842	BB	0.1885	435.91937	35.68105	39.8546
2	11.843	BB	0.2075	430.86081	31.91575	39.3921
3	15.946	BB	0.2603	98.84071	5.75847	9.0367
4	16.901	MM	0.3657	128.15315	5.84084	11.7166
Totals :				1093.77405	79.19611	

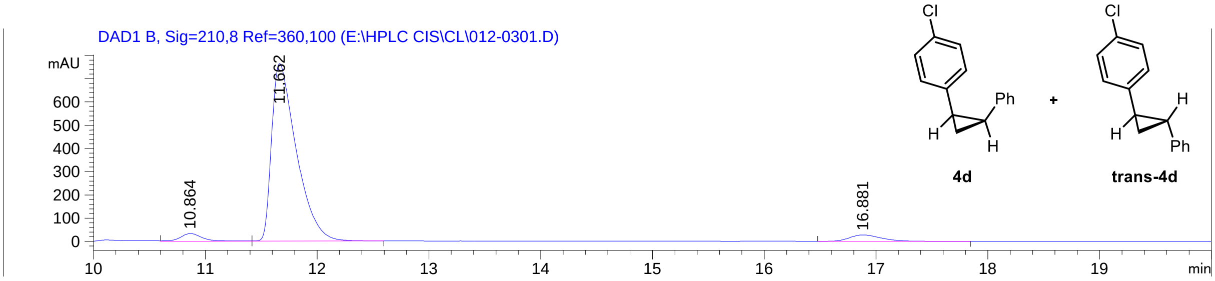

Signal 2: DAD1 B, Sig=210,8 Ref=360,100

Peak #	RetTime [min]	Type	Width [min]	Area [mAU*s]	Height [mAU]	Area %
1	10.864	VV	0.2064	453.90817	33.01723	3.5789
2	11.662	VB	0.2267	1.16959e4	763.62457	92.2188
3	16.881	BB	0.3009	532.97003	27.39898	4.2023
Totals :				1.26828e4	824.04078	

Analysis Conditions: Daicel Chiralpak-IB column (0.46 cm internal diameter x 25cm), temperature 25°C, eluent Hexane, flow rate 1 mL/min, λ: 210 nm.

HPLC trace for 4e:

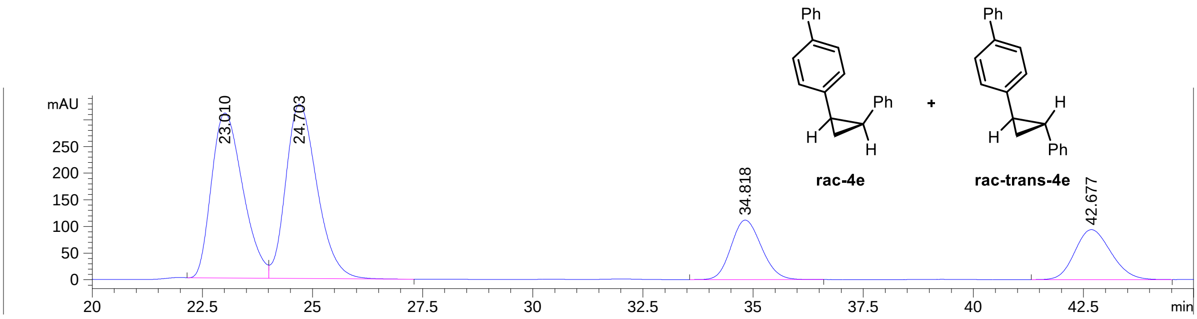

Signal 3: DAD1 C, Sig=210,8 Ref=360,100

Peak #	RetTime [min]	Type	Width [min]	Area [mAU*s]	Height [mAU]	Area %
1	23.010	BV	0.7770	1.53031e4	309.43289	36.3165
2	24.703	VB	0.7644	1.61491e4	326.88589	38.3242
3	34.818	BB	0.7430	5347.33350	111.60691	12.6900
4	42.677	BB	0.8625	5338.56104	93.72369	12.6692

Totals : 4.21381e4 841.64939

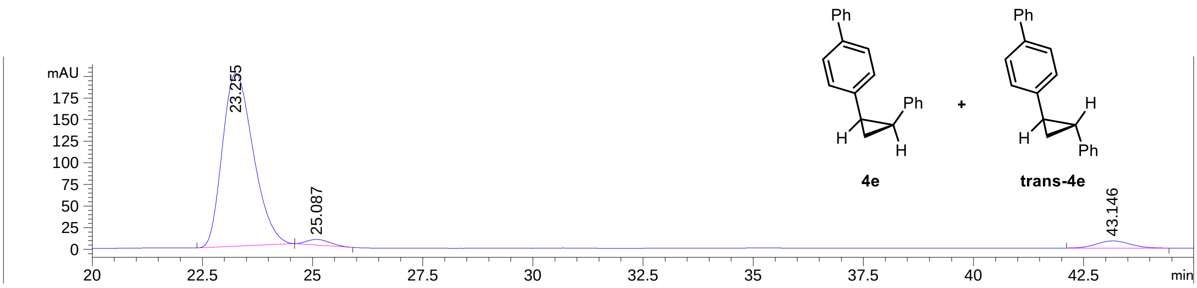

Signal 3: DAD1 C, Sig=210,8 Ref=360,100

Peak #	RetTime [min]	Type	Width [min]	Area [mAU*s]	Height [mAU]	Area %
1	23.255	BB	0.7594	9738.43066	200.23973	93.4359
2	25.087	BB	0.4787	243.04141	6.55362	2.3319
3	43.146	BB	0.6313	441.11194	8.33658	4.2323

Totals : 1.04226e4 215.12993

Analysis Conditions: Daicel Chiralpak-OJ-H column (0.46 cm internal diameter x 25cm), temperature 25°C, eluent 80:20 Hexane:*i*-PrOH, flow rate 0.5 mL/min, λ: 210 nm.

HPLC trace for 4f:

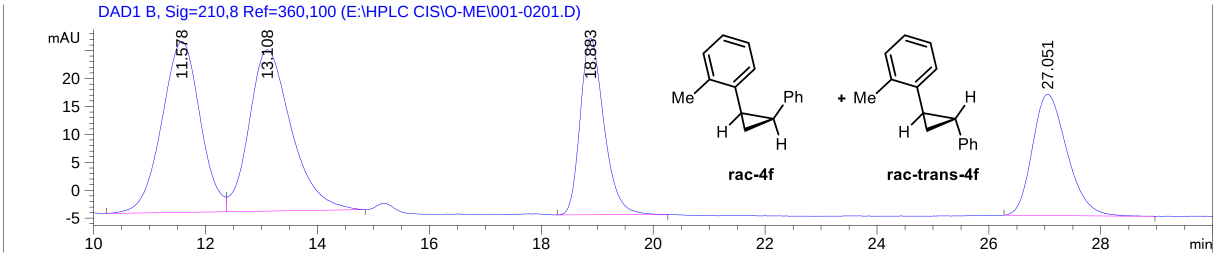

Signal 2: DAD1 B, Sig=210,8 Ref=360,100

Peak #	RetTime [min]	Type	Width [min]	Area [mAU*s]	Height [mAU]	Area %
1	11.578	BV	0.7270	1454.09973	30.56906	30.1226
2	13.108	VB	0.7633	1511.83875	28.85257	31.3187
3	18.883	BB	0.4532	929.70221	31.60043	19.2594
4	27.051	BB	0.6466	931.62994	21.71385	19.2993

Totals : 4827.27063 112.73592

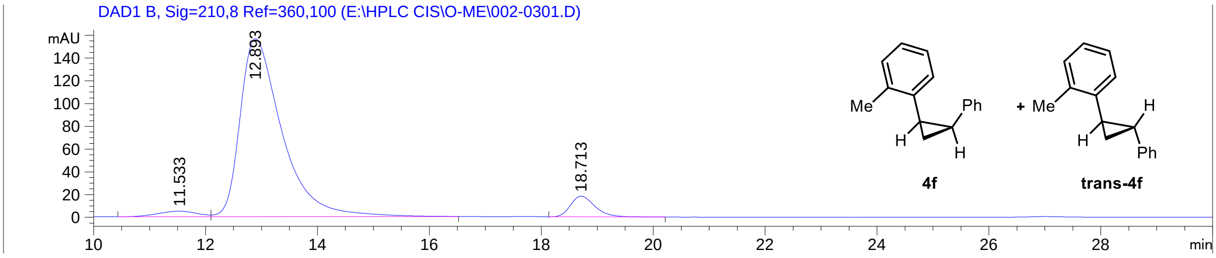

Signal 2: DAD1 B, Sig=210,8 Ref=360,100

Peak #	RetTime [min]	Type	Width [min]	Area [mAU*s]	Height [mAU]	Area %
1	11.533	BV	0.5796	235.46376	4.89126	2.6442
2	12.893	VB	0.7811	8112.91895	156.43448	91.1073
3	18.713	BB	0.4645	556.41467	18.31292	6.2485

Totals : 8904.79738 179.63865

Analysis Conditions: Daicel Chiralpak-OJ-H column (0.46 cm internal diameter x 25cm), temperature 25°C, eluent Hexane, flow rate 1 mL/min, λ: 210 nm.

HPLC trace for 4g:

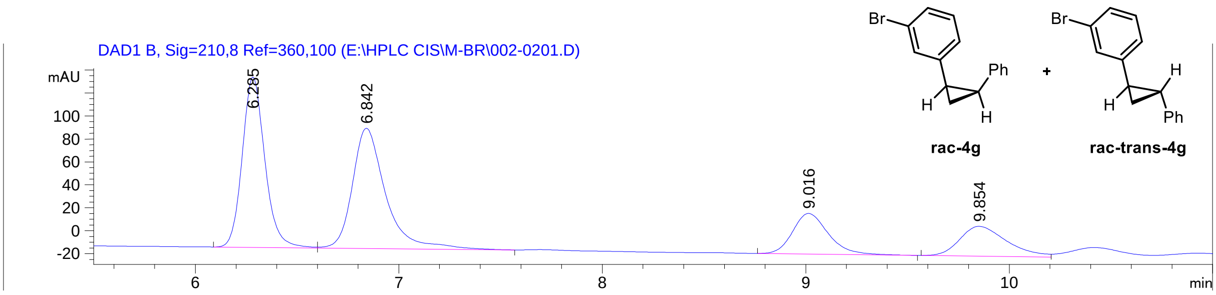

Signal 2: DAD1 B, Sig=210,8 Ref=360,100

Peak #	RetTime [min]	Type	Width [min]	Area [mAU*s]	Height [mAU]	Area %
1	6.285	BV	0.1180	1145.38745	148.51521	35.9662
2	6.842	VB	0.1704	1189.38000	104.80462	37.3476
3	9.016	BB	0.1863	431.87808	35.41222	13.5614
4	9.854	BV	0.2418	417.97867	25.96820	13.1249

Totals : 3184.62421 314.70025

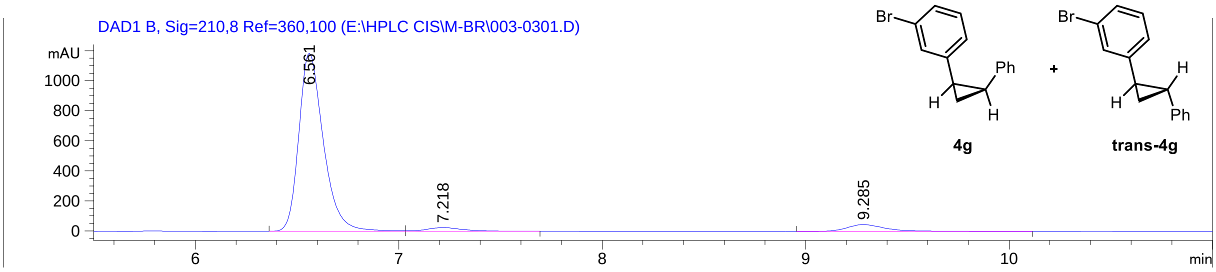

Signal 2: DAD1 B, Sig=210,8 Ref=360,100

Peak #	RetTime [min]	Type	Width [min]	Area [mAU*s]	Height [mAU]	Area %
1	6.561	VV	0.1279	9949.41504	1185.48523	91.6695
2	7.218	VB	0.1856	308.32840	24.70280	2.8408
3	9.285	BB	0.1979	595.82312	45.15876	5.4897

Totals : 1.08536e4 1255.34679

Analysis Conditions: Daicel Chiralpak-IA column (0.46 cm internal diameter x 25cm), temperature 25°C, eluent Hexane, flow rate 1 mL/min, λ: 210 nm.

HPLC trace for 4h:

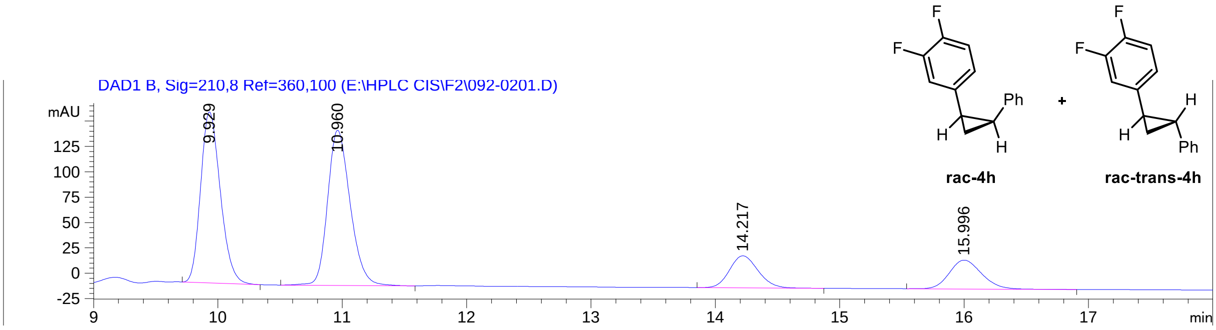

Signal 2: DAD1 B, Sig=210,8 Ref=360,100

Peak #	RetTime [min]	Type	Width [min]	Area [mAU*s]	Height [mAU]	Area %
1	9.929	BB	0.1703	1854.54138	168.60869	38.6385
2	10.960	BB	0.1950	1928.76343	153.07634	40.1849
3	14.217	BB	0.2460	503.55490	31.59138	10.4913
4	15.996	BB	0.2784	512.86395	28.43902	10.6853

Totals : 4799.72366 381.71543

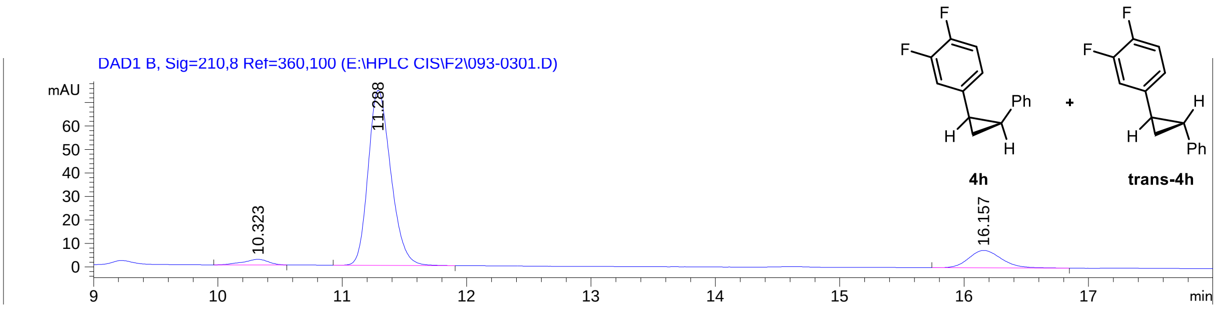

Signal 2: DAD1 B, Sig=210,8 Ref=360,100

Peak #	RetTime [min]	Type	Width [min]	Area [mAU*s]	Height [mAU]	Area %
1	10.323	BB	0.2065	33.92763	2.40582	3.0410
2	11.288	BB	0.1958	945.40253	74.64990	84.7382
3	16.157	BB	0.2838	136.34473	7.43951	12.2208

Totals : 1115.67488 84.49524

Analysis Conditions: Daicel Chiralpak-IF column (0.46 cm internal diameter x 25cm), temperature 25°C, eluent Hexane, flow rate 1 mL/min, λ: 210 nm.

HPLC trace for 4i:

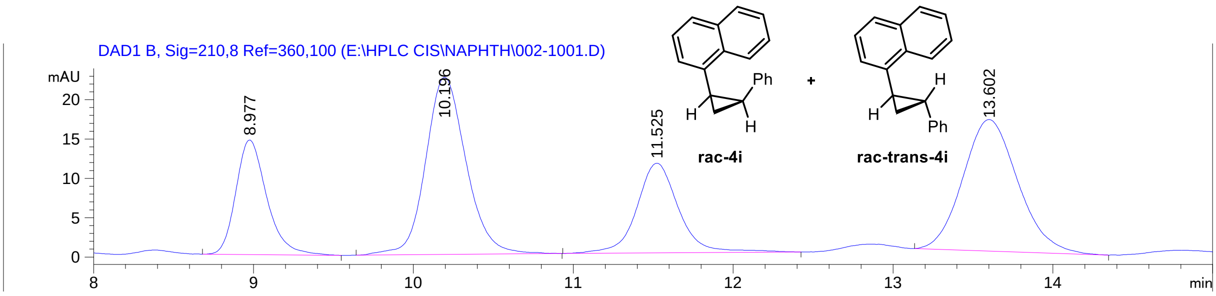

Signal 2: DAD1 B, Sig=210,8 Ref=360,100

Peak #	RetTime [min]	Type	Width [min]	Area [mAU*s]	Height [mAU]	Area %
1	8.977	BB	0.2068	195.98303	14.57788	16.3249
2	10.196	BB	0.2672	396.51782	22.54517	33.0289
3	11.525	BB	0.2768	208.21089	11.41714	17.3434
4	13.602	BB	0.3650	399.80554	16.71866	33.3028
Totals :				1200.51729	65.25885	

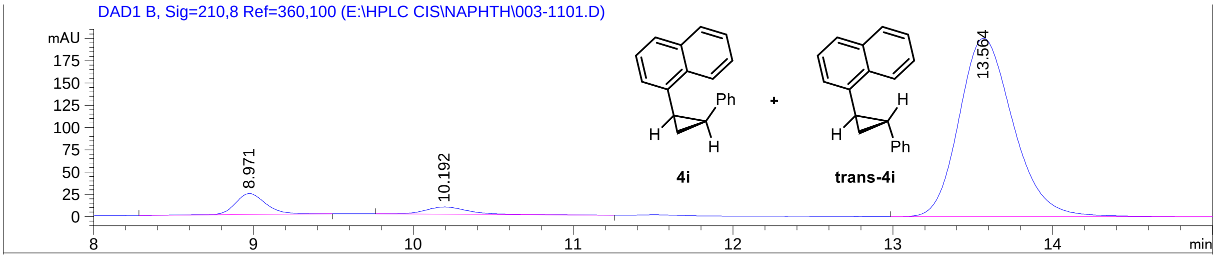

Signal 2: DAD1 B, Sig=210,8 Ref=360,100

Peak #	RetTime [min]	Type	Width [min]	Area [mAU*s]	Height [mAU]	Area %
1	8.971	BB	0.2042	311.20114	23.54065	6.0251
2	10.192	BB	0.2849	152.32506	8.04324	2.9491
3	13.564	BB	0.3618	4701.54395	200.35014	91.0258
Totals :				5165.07014	231.93403	

Analysis Conditions: Daicel Chiralpak-OJ-H column (0.46 cm internal diameter x 25cm), temperature 25°C, eluent 90:10 Hexane:*i*-PrOH, flow rate 1 mL/min, λ: 210 nm.

SFC trace for 4j:

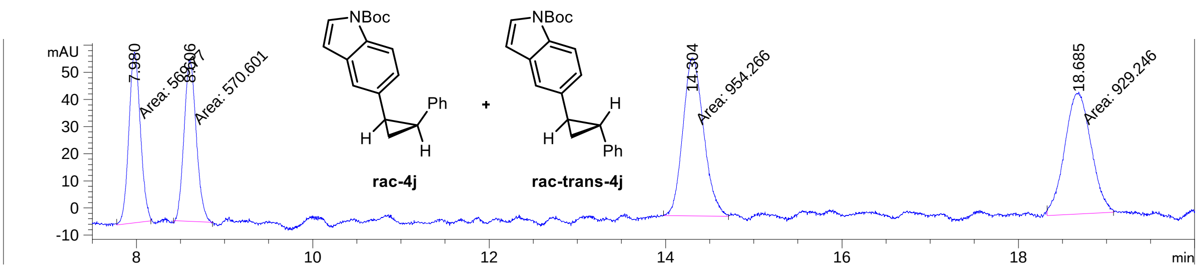

Signal 2: DAD1 B, Sig=230,4 Ref=off

Peak #	RetTime [min]	Type	Width [min]	Area [mAU*s]	Height [mAU]	Area %
1	7.980	MM	0.1508	569.77014	62.99105	18.8423
2	8.606	MM	0.1601	570.60120	59.39034	18.8698
3	14.304	MM	0.2730	954.26563	58.26799	31.5576
4	18.685	MM	0.3446	929.24634	44.94716	30.7302
Totals :				3023.88330	225.59654	

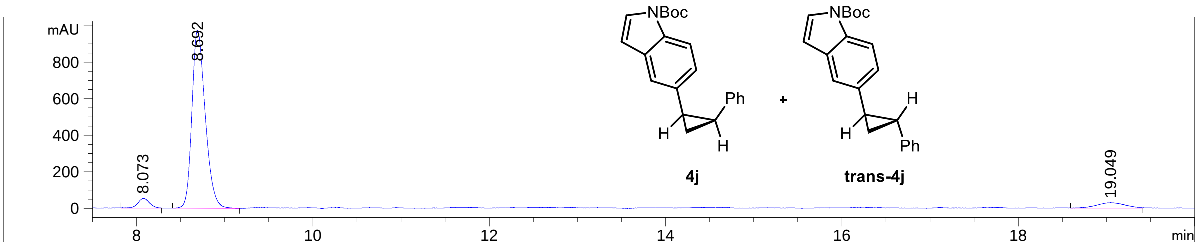

Signal 2: DAD1 B, Sig=230,4 Ref=off

Peak #	RetTime [min]	Type	Width [min]	Area [mAU*s]	Height [mAU]	Area %
1	8.073	VB	0.1125	498.80228	53.90529	4.3847
2	8.692	BB	0.1583	1.02306e4	976.27045	89.9308
3	19.049	BV	0.2585	646.67700	29.64105	5.6845
Totals :				1.13761e4	1059.81679	

Analysis Conditions: Daicel Chiralpak-OJ-H column (0.46 cm internal diameter x 25cm), temperature 25°C, eluent MeOH 20%, flow rate 1 mL/min, λ: 230 nm.

HPLC trace for 4k:

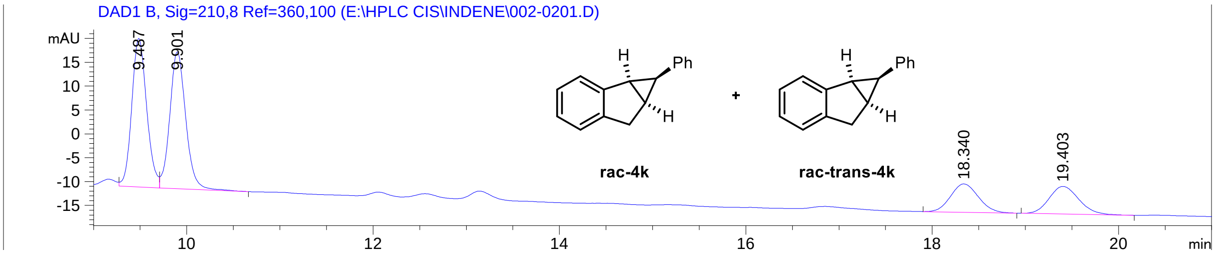

Signal 2: DAD1 B, Sig=210,8 Ref=360,100

Peak #	RetTime [min]	Type	Width [min]	Area [mAU*s]	Height [mAU]	Area %
1	9.487	VV	0.1715	345.79999	31.14869	36.7502
2	9.901	VB	0.1811	342.06989	28.70051	36.3537
3	18.340	BB	0.3117	124.27502	5.95058	13.2074
4	19.403	BB	0.3500	128.80354	5.78015	13.6887

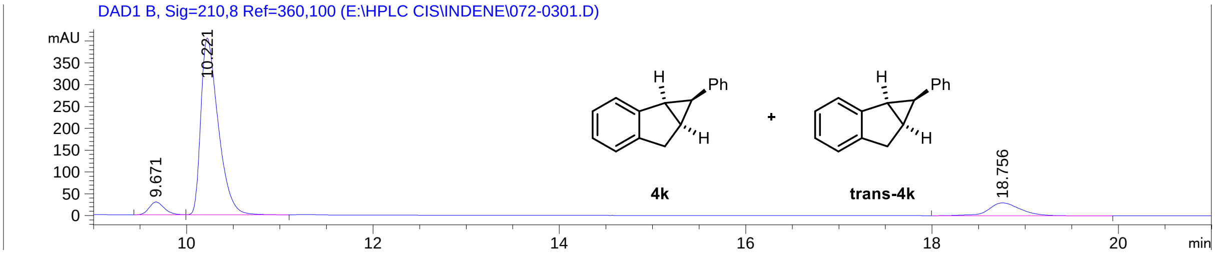

Signal 2: DAD1 B, Sig=210,8 Ref=360,100

Peak #	RetTime [min]	Type	Width [min]	Area [mAU*s]	Height [mAU]	Area %
1	9.671	BV	0.1750	327.84125	29.20081	5.2367
2	10.221	VB	0.1948	5236.61768	405.03387	83.6455
3	18.756	BB	0.3567	696.02710	29.55742	11.1178
Totals :				6260.48602	463.79210	

Analysis Conditions: Daicel Chiralpak-IB column (0.46 cm internal diameter x 25cm), temperature 25°C, eluent Hexane, flow rate 1 mL/min, λ: 210 nm.

HPLC trace for 4l:

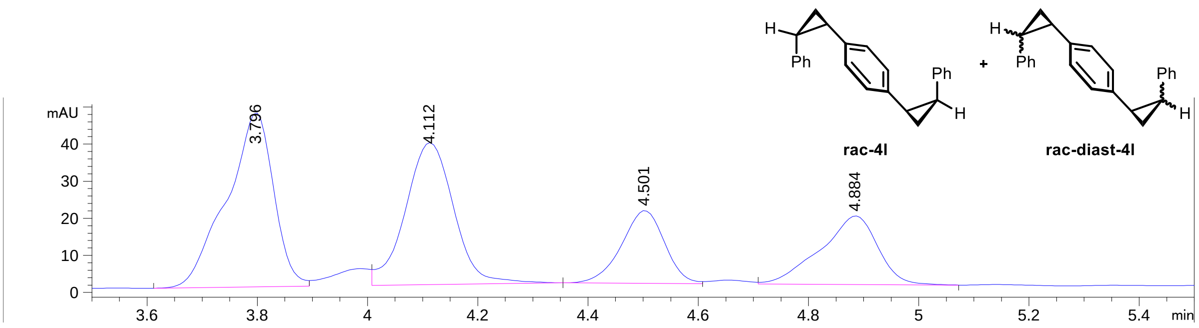

Signal 2: DAD1 B, Sig=210,8 Ref=360,100

Peak #	RetTime [min]	Type	Width [min]	Area [mAU*s]	Height [mAU]	Area %
1	3.796	BV	0.0850	277.99921	47.02260	37.0025
2	4.112	VB	0.0931	233.39400	38.29786	31.0654
3	4.501	BV	0.0834	106.61781	19.62731	14.1911
4	4.884	VB	0.1040	133.28755	18.51572	17.7410

Totals : 751.29857 123.46349

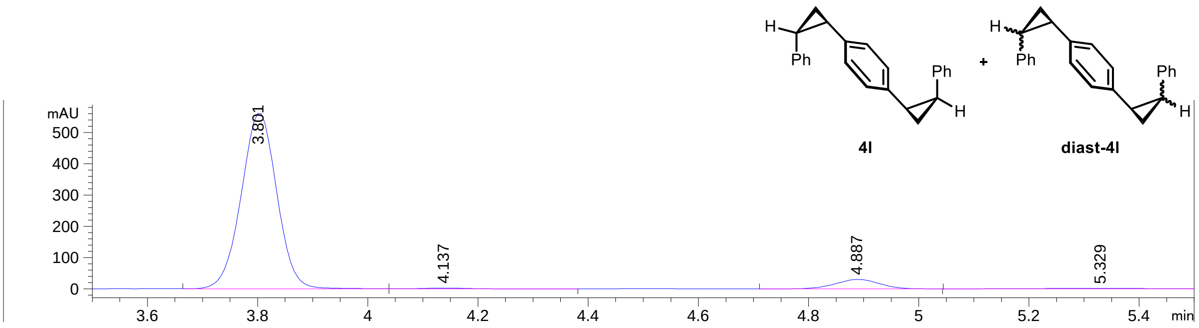

Signal 2: DAD1 B, Sig=210,8 Ref=360,100

Peak #	RetTime [min]	Type	Width [min]	Area [mAU*s]	Height [mAU]	Area %
1	3.801	VV	0.0683	2528.83276	564.05518	92.4006
2	4.137	VB	0.0955	16.89190	2.60926	0.6172
3	4.887	BB	0.0843	166.23207	30.14796	6.0739
4	5.329	BB	0.2248	24.85790	1.45875	0.9083

Totals : 2736.81464 598.27114

Analysis Conditions: Daicel Chiralpak-IA column (0.46 cm internal diameter x 25cm), temperature 25°C, eluent 90:10 Hexane:*i*-PrOH, flow rate 1 mL/min, λ: 210 nm.

HPLC trace for 4m:

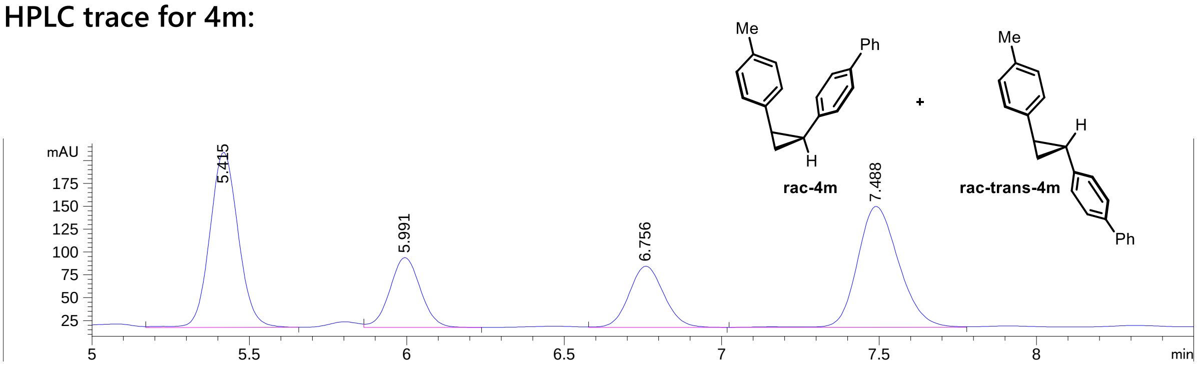

Signal 2: DAD1 B, Sig=210,8 Ref=360,100

Peak #	RetTime [min]	Type	Width [min]	Area [mAU*s]	Height [mAU]	Area %
1	5.415	VB	0.0971	1201.89075	191.85324	35.0822
2	5.991	VB	0.1045	514.61041	76.51008	15.0211
3	6.756	VB	0.1190	511.37463	67.00491	14.9266
4	7.488	BV	0.1397	1198.04895	132.22762	34.9701

Totals : 3425.92474 467.59585

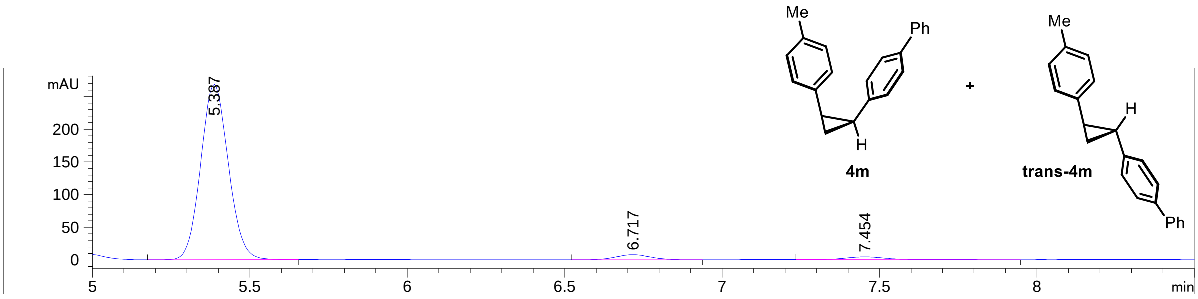

Signal 2: DAD1 B, Sig=210,8 Ref=360,100

Peak #	RetTime [min]	Type	Width [min]	Area [mAU*s]	Height [mAU]	Area %
1	5.387	BB	0.0953	1639.16699	268.19604	94.4506
2	6.717	BB	0.1161	58.97489	7.81170	3.3982
3	7.454	BB	0.1380	37.33273	4.18808	2.1512

Totals : 1735.47461 280.19582

Analysis Conditions: Daicel Chiralpak-IB column (0.46 cm internal diameter x 25cm), temperature 25°C, eluent 95:5 Hexane:*i*-PrOH, flow rate 1 mL/min, λ: 210 nm.

HPLC trace for 4n:

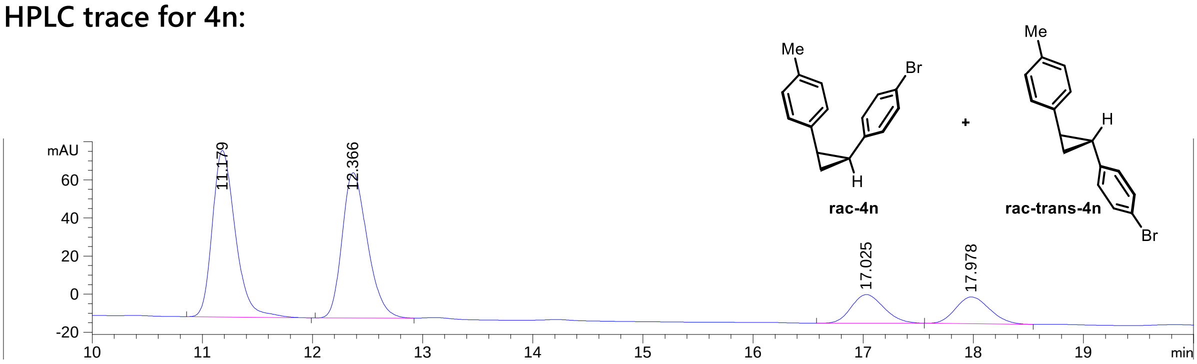

Signal 2: DAD1 B, Sig=210,8 Ref=360,100

Peak #	RetTime [min]	Type	Width [min]	Area [mAU*s]	Height [mAU]	Area %
1	11.179	BB	0.2214	1270.63184	87.50924	41.0237
2	12.366	BB	0.2456	1214.66565	76.36729	39.2168
3	17.025	BB	0.3217	309.43738	15.05813	9.9905
4	17.978	BB	0.3407	302.57663	14.07816	9.7690

Totals : 3097.31149 193.01282

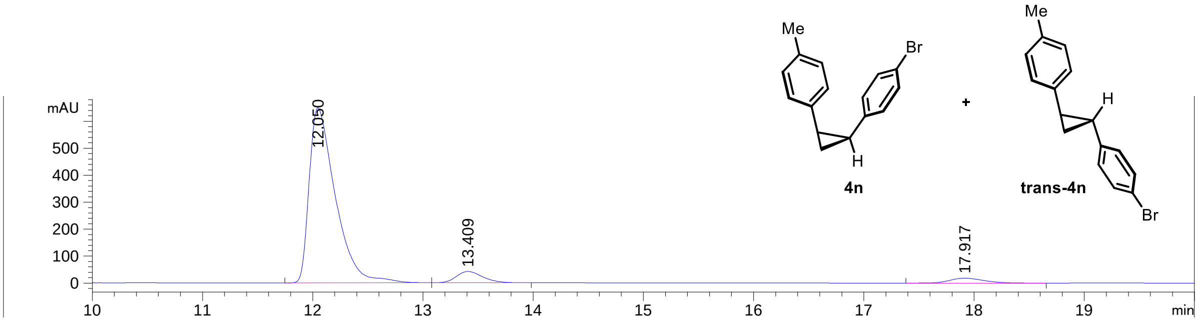

Signal 2: DAD1 B, Sig=210,8 Ref=360,100

Peak #	RetTime [min]	Type	Width [min]	Area [mAU*s]	Height [mAU]	Area %
1	12.050	BV	0.2460	1.06779e4	648.82959	90.7419
2	13.409	VB	0.2556	698.75366	42.10201	5.9381
3	17.917	BB	0.3301	390.68661	18.22361	3.3201

Totals : 1.17674e4 709.15520

Analysis Conditions: Daicel Chiralpak-IB column (0.46 cm internal diameter x 25cm), temperature 25°C, eluent Hexane, flow rate 1 mL/min, λ: 210 nm.

HPLC trace for 4o:

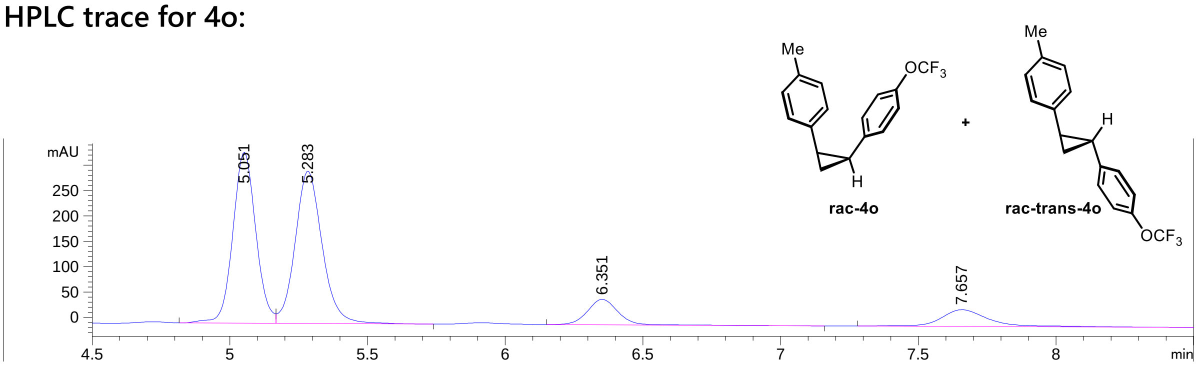

Signal 2: DAD1 B, Sig=210,8 Ref=360,100

Peak #	RetTime [min]	Type	Width [min]	Area [mAU*s]	Height [mAU]	Area %
1	5.051	BV	0.0898	1960.97986	337.58185	40.6457
2	5.283	VB	0.1037	2054.20459	300.80112	42.5780
3	6.351	VB	0.1202	398.91483	50.46457	8.2684
4	7.657	BB	0.1830	410.47025	33.01555	8.5079

Totals : 4824.56952 721.86308

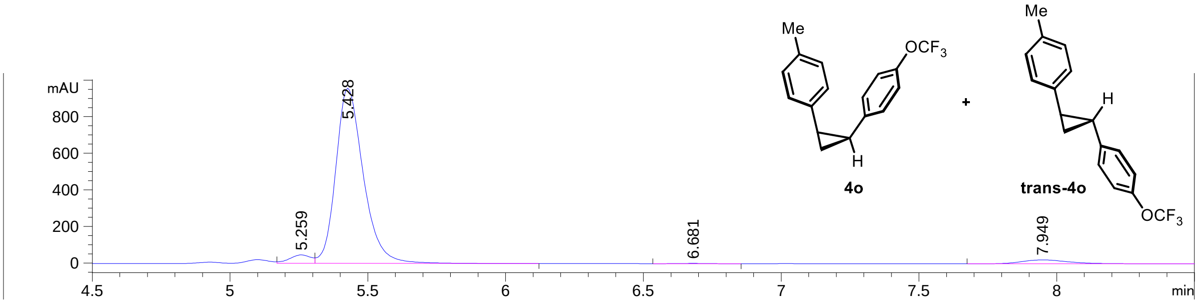

Signal 2: DAD1 B, Sig=210,8 Ref=360,100

Peak #	RetTime [min]	Type	Width [min]	Area [mAU*s]	Height [mAU]	Area %
1	5.259	VV	0.0806	247.70273	46.17984	3.4515
2	5.428	VB	0.1051	6661.02637	958.05084	92.8148
3	6.681	BB	0.1186	12.57915	1.65730	0.1753
4	7.949	BB	0.1809	255.37949	21.44932	3.5585

Totals : 7176.68774 1027.33729

Analysis Conditions: Daicel Chiralpak-IA column (0.46 cm internal diameter x 25cm), temperature 25°C, eluent Hexane 100%, flow rate 1 mL/min, λ: 210 nm.

SFC trace for 4p:

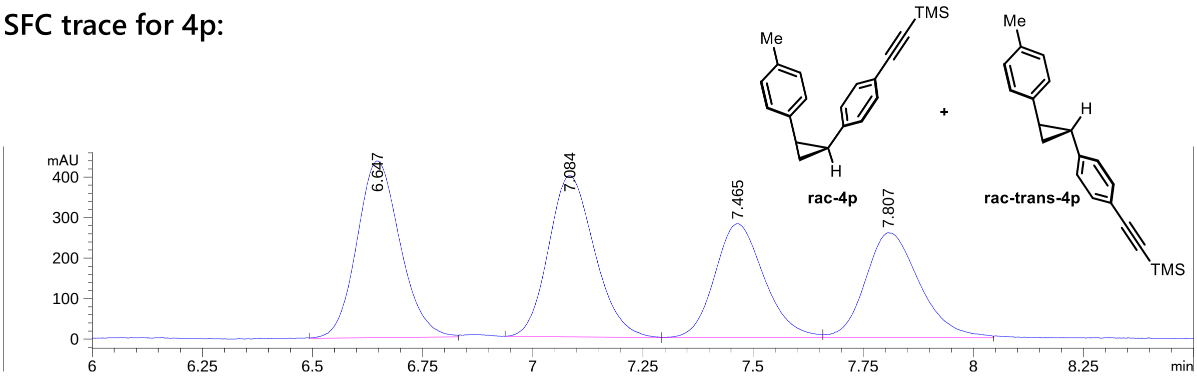

Signal 3: DAD1 C, Sig=210,4 Ref=off

Peak #	RetTime [min]	Type	Width [min]	Area [mAU*s]	Height [mAU]	Area %
1	6.647	BV	0.1024	2967.69312	435.90277	28.9479
2	7.084	BB	0.0974	2882.77100	398.46722	28.1196
3	7.465	BV	0.1108	2188.15210	281.18994	21.3440
4	7.807	VV	0.1126	2213.21460	259.30954	21.5885
Totals :				1.02518e4	1374.86948	

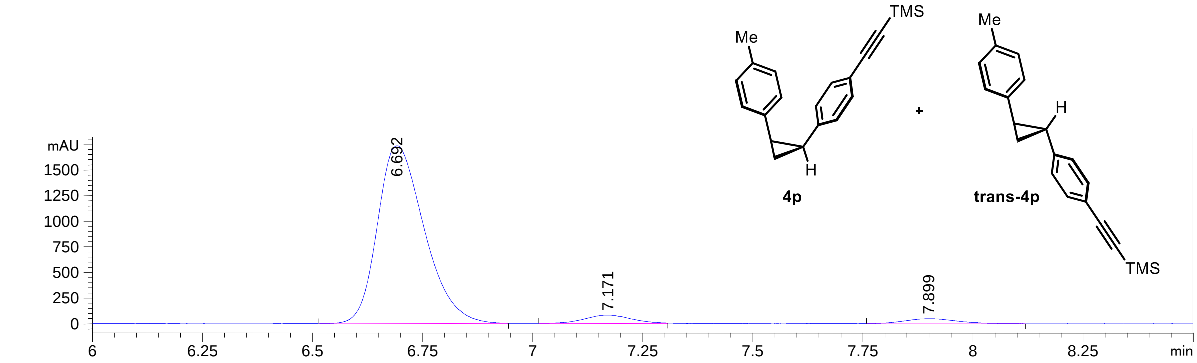

Signal 3: DAD1 C, Sig=210,4 Ref=off

Peak #	RetTime [min]	Type	Width [min]	Area [mAU*s]	Height [mAU]	Area %
1	6.692	BV	0.1114	1.28325e4	1733.61304	92.6458
2	7.171	VV	0.0969	602.37274	81.32830	4.3489
3	7.899	VV	0.1010	416.26407	49.57407	3.0053
Totals :				1.38512e4	1864.51541	

Analysis Conditions: Daicel Chiralpak-IB column (0.46 cm internal diameter x 25cm), temperature 25°C, eluent MeOH 5%, flow rate 1 mL/min, λ: 210 nm.

SFC trace for 4q:

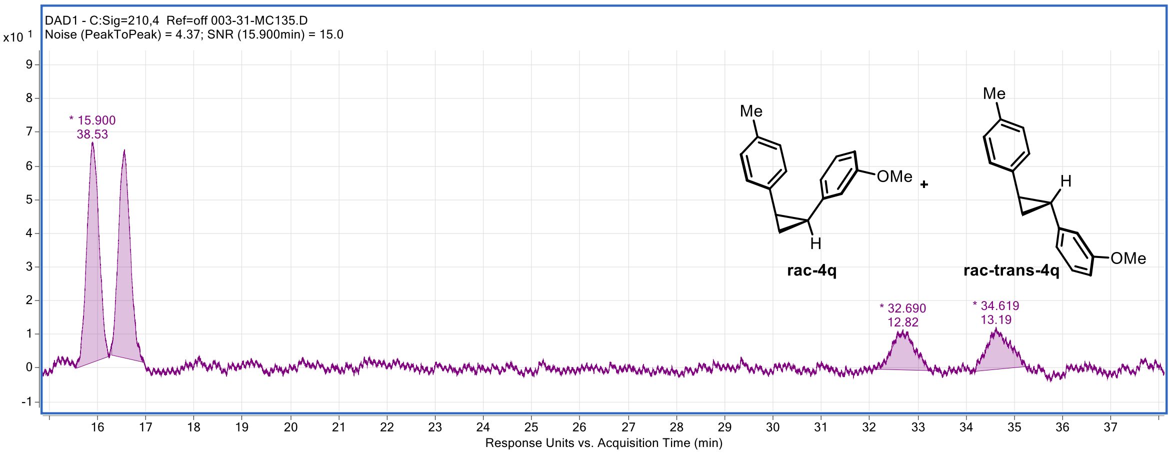

Integration Peak List						
Peak	RT	Width	Height	FWHM	Area	Area Sum %
1	15.900	0.675	65.3	0.278	1132.87	38.53
2	16.561	0.675	61.85	0.267	1042.59	35.46
3	32.690	1.430	11.69	0.541	376.95	12.82
4	34.619	1.107	12.28	0.597	387.96	13.19

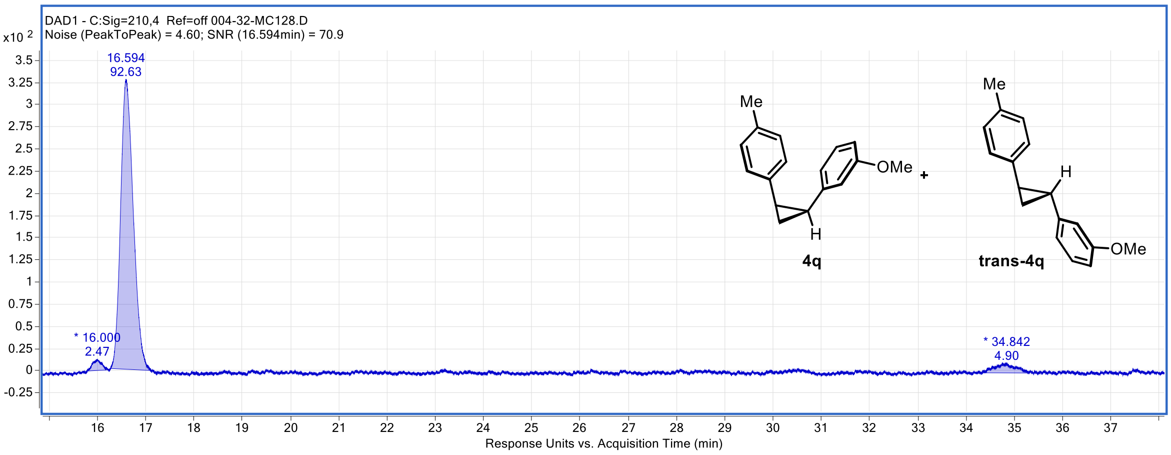

Integration Peak List						
Peak	RT	Width	Height	FWHM	Area	Area Sum %
1	16.000	0.465	12.07	0.236	159.08	2.47
2	16.594	0.832	325.9	0.284	5959.09	92.63
3	34.842	1.022	10.08	0.626	34.842	4.90

Analysis Conditions: Daicel Chiralpak-OJ-H column (0.46 cm internal diameter x 25cm), temperature 25°C, eluent MeOH 5%, flow rate 1 mL/min, λ: 210 nm.

HPLC trace for 4r:

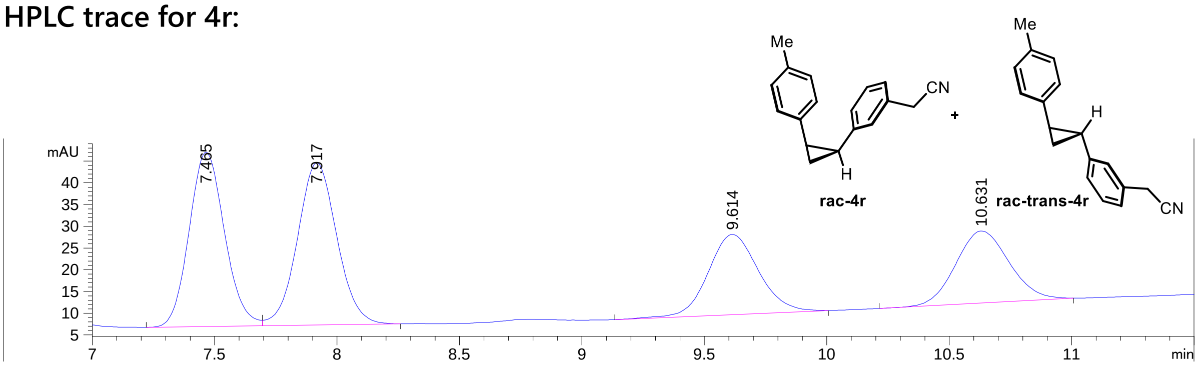

Signal 2: DAD1 B, Sig=210,8 Ref=360,100

Peak #	RetTime [min]	Type	Width [min]	Area [mAU*s]	Height [mAU]	Area %
1	7.465	BV	0.1554	403.82526	40.14590	30.1821
2	7.917	VB	0.1706	418.00073	37.35253	31.2416
3	9.614	BB	0.2211	264.07584	18.43509	19.7371
4	10.631	BB	0.2331	252.06219	16.60843	18.8392

Totals : 1337.96402 112.54195

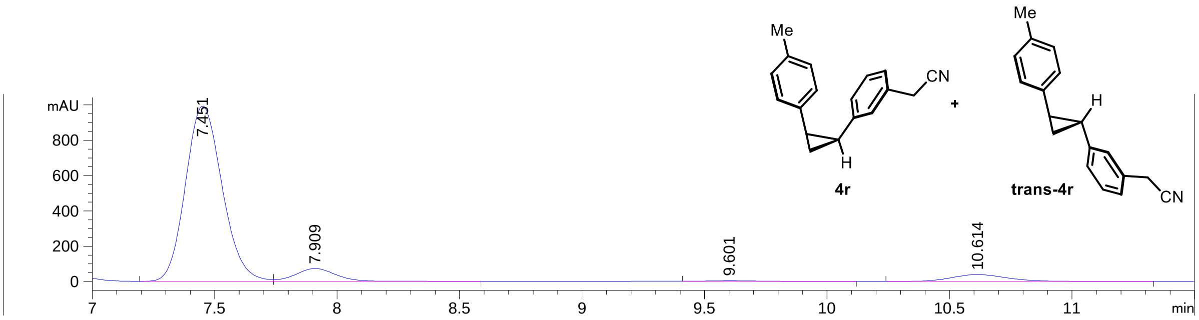

Signal 2: DAD1 B, Sig=210,8 Ref=360,100

Peak #	RetTime [min]	Type	Width [min]	Area [mAU*s]	Height [mAU]	Area %
1	7.451	VV	0.1613	1.03255e4	992.97083	87.4303
2	7.909	VB	0.1765	836.46582	72.58260	7.0827
3	9.601	VB	0.2324	56.28412	3.56058	0.4766
4	10.614	BB	0.2384	591.72717	38.26799	5.0104

Totals : 1.18100e4 1107.38198

Analysis Conditions: Daicel Chiralpak-AS_H column (0.46 cm internal diameter x 25cm), temperature 25°C, eluent 80:20 Hexane:*i*-PrOH, flow rate 1 mL/min, λ: 210 nm.

SFC trace for 4s:

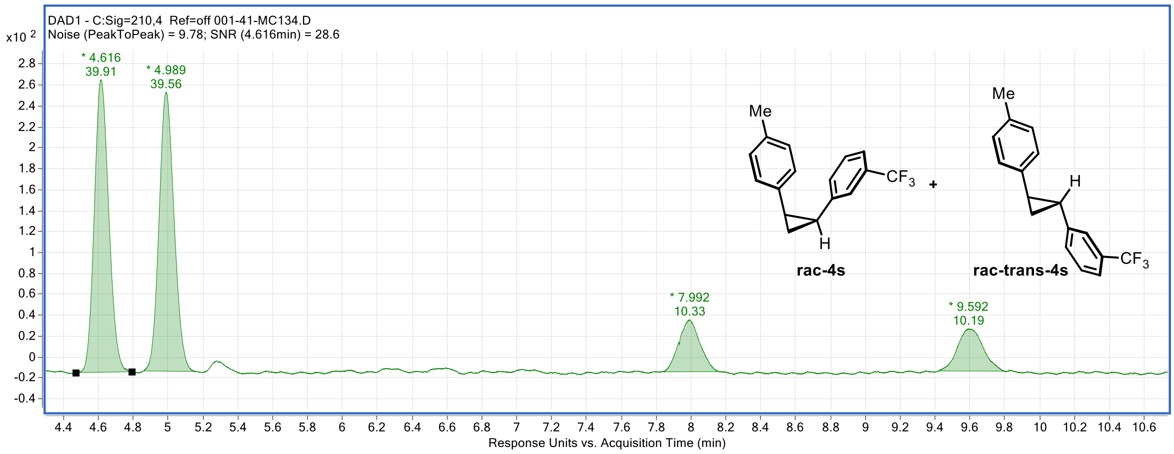

Integration Peak List						
Peak	RT	Width	Height	FWHM	Area	Area Sum %
1	4.616	0.321	279.54	0.090	1615.61	39.91
2	4.989	0.292	266.72	0.093	1601.45	39.56
3	7.992	0.350	49.86	0.134	418.26	10.33
4	9.52	0.379	40.40	0.161	412.40	10.19

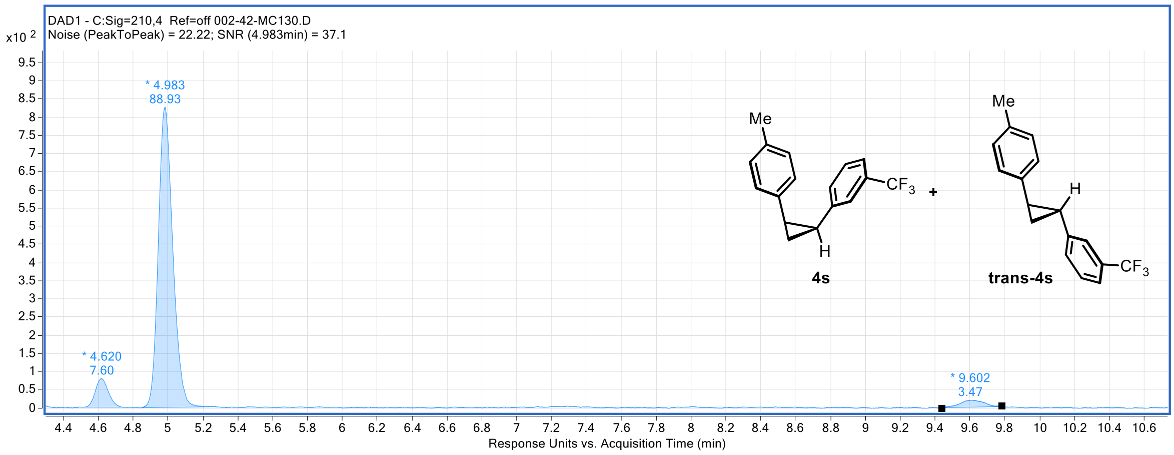

Integration Peak List						
Peak	RT	Width	Height	FWHM	Area	Area Sum %
1	4.620	0.224	78.59	0.080	407.36	7.60
2	4.983	0.395	825.58	0.088	4766.97	88.93
3	9.602	0.346	19.43	0.153	186.19	3.47

Analysis Conditions: Daicel Chiralpak-OJ-H column (0.46 cm internal diameter x 25cm), temperature 25°C, eluent MeOH 5%, flow rate 1 mL/min, λ: 210 nm.

HPLC trace for 4t:

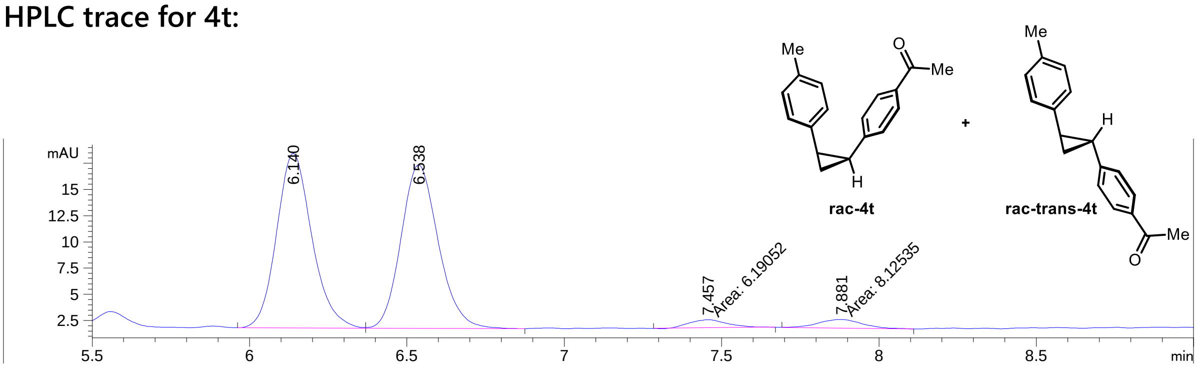

Signal 2: DAD1 B, Sig=210,8 Ref=360,100

Peak #	RetTime [min]	Type	Width [min]	Area [mAU*s]	Height [mAU]	Area %
1	6.140	BV	0.1190	129.95894	16.66458	47.6548
2	6.538	VB	0.1280	128.43434	15.60536	47.0957
3	7.457	MM	0.1354	6.19052	7.62058e-1	2.2700
4	7.881	MM	0.1624	8.12535	8.33941e-1	2.9795

Totals : 272.70915 33.86594

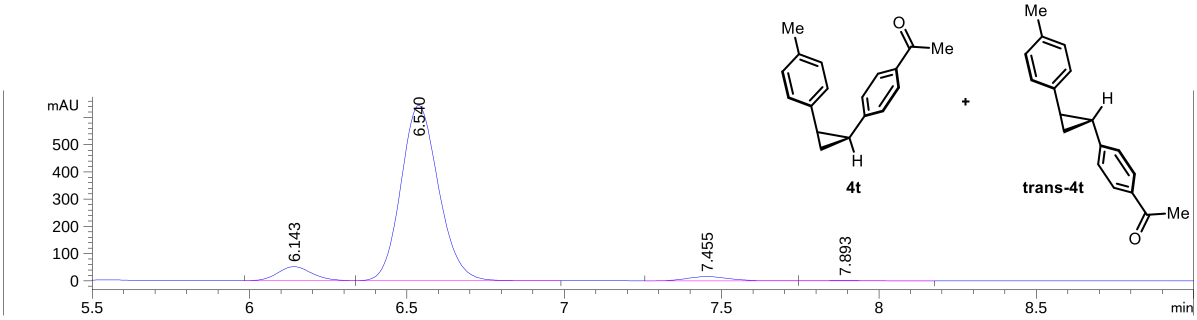

Signal 2: DAD1 B, Sig=210,8 Ref=360,100

Peak #	RetTime [min]	Type	Width [min]	Area [mAU*s]	Height [mAU]	Area %
1	6.143	VV	0.1179	388.94882	51.59669	6.6098
2	6.540	VB	0.1286	5336.97656	644.61127	90.6970
3	7.455	BV	0.1413	142.09523	15.45053	2.4148
4	7.893	VB	0.1641	16.38303	1.51549	0.2784

Totals : 5884.40365 713.17398

Analysis Conditions: Daicel Chiralpak-OZ-H column (0.46 cm internal diameter x 25cm), temperature 25°C, eluent Hexane:iPrOH 90:10, 1 mL/min, flow rate 1 mL/min, λ: 210 nm.

HPLC trace for 4v:

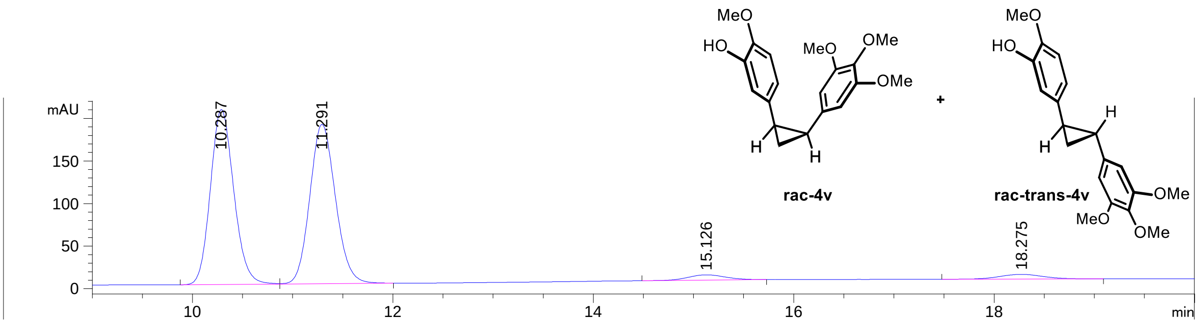

Signal 2: DAD1 B, Sig=210,8 Ref=360,100

Peak #	RetTime [min]	Type	Width [min]	Area [mAU*s]	Height [mAU]	Area %
1	10.287	BV	0.2511	3329.15649	205.37886	47.6235
2	11.291	VB	0.2775	3326.64697	187.00842	47.5876
3	15.126	BB	0.3594	163.80701	6.25755	2.3433
4	18.275	BB	0.4074	170.96663	5.51837	2.4457
Totals :				6990.57710	404.16320	

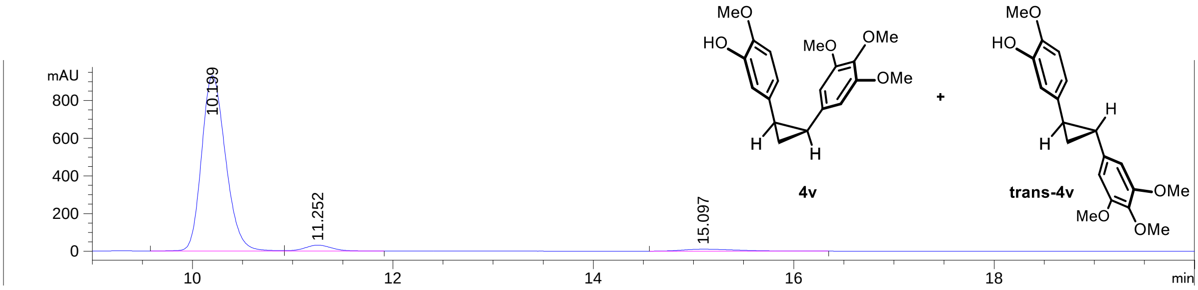

Signal 2: DAD1 B, Sig=210,8 Ref=360,100

Peak #	RetTime [min]	Type	Width [min]	Area [mAU*s]	Height [mAU]	Area %
1	10.199	BV	0.2599	1.54662e4	930.59155	94.0580
2	11.252	VB	0.2831	583.93207	31.38230	3.5512
3	15.097	BB	0.5397	393.12741	10.62463	2.3908
Totals :				1.64433e4	972.59848	

Analysis Conditions: Daicel Chiralpak-IB column (0.46 cm internal diameter x 25cm), temperature 25°C, eluent 70:30 Hexane:*i*-PrOH, flow rate 1 mL/min, λ: 210 nm.
